# Linear Stability of the Slowly-Rotating Kerr-de Sitter Family

**DOI:** 10.1007/s40818-026-00236-4

**Published:** 2026-04-30

**Authors:** Allen Juntao Fang

**Affiliations:** Laboratoire Jacques-Louis Lions, Sorbonne Université, 4 Place Jussieu, Paris, 75005 Île-de-France France

**Keywords:** General relativity, Black hole stability, Harmonic coordinates, Wave equation, 35L52, 58J45, 83C05, 83C57

## Abstract

In this paper, we prove that the slowly-rotating Kerr-de Sitter family of black holes is linearly stable as a family of solutions to the Einstein vacuum equations with $\Lambda >0$ in harmonic (wave) gauge. This article is part of a series that provides a novel proof of the full nonlinear stability of the slowly-rotating Kerr-de Sitter family. This paper and its follow-up offer a self-contained alternative approach to nonlinear stability of the Kerr-de Sitter family from the original work of Hintz, Vasy Hintz and Vasy (Acta Math. **220**(1), 1–206 (2018a). 10.4310/ACTA.2018.v220.n1.a1) by interpreting quasinormal modes as $H^k$ eigenvalues of an operator on a Hilbert space, and using integrated local energy decay estimates to prove the existence of a spectral gap. We also do not compactify the spacetime, thus avoiding the use of *b*-calculus and instead only use standard pseudo-differential arguments in a neighborhood of the trapped set; and avoid constraint damping altogether. The methods in the current paper offer an explicit example of how to use the vectorfield method to achieve resolvent estimates on a trapping background.

## Introduction

The aim of this paper is to offer the linear theory needed for a novel proof of the global nonlinear stability of the slowly-rotating Kerr-de Sitter family of black hole solutions to Einstein’s vacuum equations with a positive cosmological constant. In the process of doing so, we also hope to contribute a deeper understanding of quasinormal modes of wave equations using a vectorfield approach.

### The Black Hole Stability Problem

The Einstein vacuum equations (EVE), which govern Einstein’s theory of relativity under the assumption of vacuum, are given by

1 $$\begin{aligned} {\text {Ric}}(g) - \Lambda g = 0, \end{aligned}$$

where *g* is a Lorentzian metric with signature $(-,+,+,+)$ on a manifold $\mathcal {M}$ (which for the sake of this paper, we will assume is $3+1$ dimensional), ${\text {Ric}}$ denotes its Ricci tensor, and $\Lambda$ is the cosmological constant.

Expanding the Ricci tensor ${\text {Ric}}(g)$ as a differential operator acting on the metric tensor *g*, equation (1) is a fully nonlinear second-order partial differential equation (PDE) for the components of the metric tensor *g*, which is invariant under diffeomorphism. A well-posedness theory for EVE was shown by Choquet-Bruhat [7] and extended in Choquet-Bruhat-Geroch [8] by setting EVE in harmonic gauge (also known as wave gauge in the literature), showing the existence of a maximal globally hyperbolic development for a sufficiently smooth initial data triplet $(\Sigma , \underline{g}, k)$. We discuss the initial value problem, along with wave coordinates, in detail in Sect. 3. For a detailed classical treatment, we refer to the reader to Chapter 10 in [61], and for a more modern treatment, to [53].

Among the most interesting solutions to EVE are the families of black hole solutions. An explicit two-parameter family of black hole solutions to EVE with $\Lambda >0$ is given by the Kerr-de Sitter family of spacetimes $(\mathcal {M}, g_{M, a})$. The fixed four-dimensional manifold is taken to be isomorphic to $\mathbb {R}^+_{t_*}\times (0,\infty )_r\times \mathbb {S}^2$, and the black hole parameters (*M*, *a*) denote the mass parameter and the angular momentum of the black hole respectively. We will use $b=(M, a)$ to denote the black hole parameters. See Sect. 2.2 for a more detailed presentation of the Kerr-de Sitter family.

The Kerr-de Sitter family black holes, like their asymptotically flat cousin the Kerr family of black hole solutions to Einstein’s equations with vanishing cosmological constant, represent a family of rotating, uncharged black hole solutions. In the particular case where $a=0$, the black hole is a static, non-rotating, uncharged black hole, and the Kerr-de Sitter family reduces to the Schwarzschild-de Sitter sub-family of black hole solutions (mirroring the $a=0$ Schwarzschild sub-family of the Kerr family of solutions in the asymptotically flat case).

The classical black hole stability problem is concerned with whether the Kerr family of black hole solutions is stable as a family^1^ in the sense that initial data triplets sufficiently close to the initial data triplet of a given Kerr solution have a maximal development with a domain of outer communication which globally approaches a nearby Kerr solution. An equivalent question of stability can be formulated for the Kerr-de Sitter family, which retains some of the crucial geometric difficulties as Kerr (superradiance and trapping in particular), while featuring substantially easier analysis overall due to exponential decay at the linear level.

This paper and its sequel in [22] seek to address exactly the question of stability for the case of the Kerr-de Sitter family.

#### Theorem 1.1

(Nonlinear stability of the slowly-rotating Kerr-de Sitter family, informal statement) Suppose $(\underline{g},k)$ are smooth initial data on some 3-dimensional hypersurface $\Sigma _0$ satisfying the constraint conditions, which are close to the initial data $(\underline{g}_{b^0}, k_{b^0})$ of a slowly-rotating Kerr-de Sitter spacetime $g_{b^0}$ in some initial data norm. Then there exists a solution *g* to EVE with a positive cosmological constant, (1), such that *g* attains the initial data at $\Sigma _0$ and there exist black hole parameters $b_{\infty }=(M_\infty , a_\infty )$ such that

$$\begin{aligned} g - g_{b_\infty } = O(e^{-{\boldsymbol{\alpha }}t_*}),\quad t_*\rightarrow \infty \end{aligned}$$

for some real ${\boldsymbol{\alpha }}>0$ constant that is independent of the initial data.

This result was in fact first proven by Hintz and Vasy in their seminal work [31]. Their proof uses a modification of the harmonic gauge, to treat Einstein’s equations as a system of quasilinear wave equations for the metric

$$\begin{aligned} g^{\alpha \beta }\partial _\alpha \partial _\beta g_{\mu \nu } = \mathcal {N}_{\mu \nu }(g, \partial g). \end{aligned}$$

An important first step to analyzing the nonlinear stability of the slowly-rotating Kerr-de Sitter family is to consider the linear stability of the gauge-fixed Einstein’s equations linearized around a member of the slowly-rotating Kerr-de Sitter family. In the case of Kerr-de Sitter, the linearized system is expected to exhibit exponential decay, from which the full nonlinear stability quickly follows. Hintz and Vasy in [31] approach proving exponential decay for the linearized system by building on the numerous works on the theory of resonances on black hole spacetimes (see for instance [5, 17, 18]), in particular relying on a series of works by themselves and Dyatlov on applying the scattering resolvent method to the study of wave-type equations on asymptotically (Kerr-)de Sitter backgrounds [19, 26, 27, 30, 32, 58].

### The Theory of Scattering Resonances

The theory of scattering resonances has proven particularly powerful in analyzing asymptotic behavior of wave-like equations on asymptotically (Kerr-)de Sitter spacetimes as they are well-adapted to spectral methods, although recently, significant progress has also been made adapting the method to asymptotically flat spacetimes [25, 28, 59, 60].

The theory of resonances on black hole spacetimes is founded on finding an appropriate notion of characteristic frequencies, and using them to analyze the asymptotic behavior of the system at hand (for an in-depth explanation of the scattering resonance method, see [20]). For a stationary linear operator $L$, these characteristic frequencies (resonances) are typically defined as the poles of a meromorphic continuation of the resolvent $\widehat{L}(\sigma )^{-1}$ into the lower half-space of the complex plane $\mathbb {C}$^2^. The location of the resonances within the complex plane then determine the asymptotic behavior of solutions $\psi$ to $L\psi =0$. In particular, if it is possible to show that all the resonances are located in the lower half space, then a simple contour deformation argument proves exponential decay (see for example the analysis of the scalar wave equation on a Schwarzschild-de Sitter background in [5]). For the Einstein system in harmonic gauge linearized around a slowly-rotating Kerr-de Sitter background, it turns out that it is not true that the resonances are all located in the exponentially decaying half-space of $\mathbb {C}$. However, by proving a high-frequency resolvent estimate, it remains possible to derive the existence of a high-frequency spectral gap^3^. In the case of wave-type equations on Kerr-de Sitter, this relies on a detailed understanding of the nature of the instability of the trapped set in Kerr-de Sitter [5, 18, 19, 29]. Following a contour deformation argument, this allows a decomposition of any solution into a finite sum of linear obstacles to decay which grow at a bounded exponential rate, and an exponentially decaying remainder.

For a general nonlinear wave equation, this is as far as the linear analysis could go. Such a result, of course is entirely unsatisfactory for nonlinear decay. After all, growth at the linear level heuristically leads to even more growth at the nonlinear level, and exponential linear growth would destroy any hopes for nonlinear decay. What turns out to save nonlinear stability in the case of the Einstein equations is the geometric structure of Einstein’s equations themselves. Indeed, at the level of the linearized Einstein equations linearized around Schwarzschild-de Sitter, it is known that the non-decaying resonances are in fact unphysical [37, 42, 43] (see Sect. 10 for an detailed discussion, and Sects. 4.1 and 7 of [31] for comparison). Such a result however, is unknown for Einstein’s equations linearized around even slowly-rotating Kerr-de Sitter backgrounds. To overcome this, Hintz and Vasy introduce constraint damping to the gauge-fixed linearized Einstein equations and use perturbation theory on the constraint-damped gauge-fixed linearized Einstein equations to deduce that the remaining finite linear obstacles to decay are in fact unphysical, thus allowing them to conclude exponential decay at the linear level. Finally, to extend the linear stability results to nonlinear stability, Hintz and Vasy make use of a Nash-Moser argument [26, 31].

### The Vectorfield Method

On the other hand, much recent progress in understanding stability of black hole spacetimes (and asymptotic behavior of linear fields on black hole backgrounds more generally) has followed from the development of the vectorfield method. First used to analyze scalar wave equations, the vectorfield method has also been fruitful as a part of the study of the question of black hole stability, beginning with its role as a critical component of the breakthrough proof of nonlinear stability of Minkowski spacetime as a solution to EVE with $\Lambda =0$ [9]. Over the years, many alternative proofs for the stability of Minkowski space using variants of the vectorfield method have been developed [33, 39, 44]. The vectorfield method has also proven useful beyond the study of just Minkowski space, having played an important role in developments in the study of linear waves on black hole backgrounds [12, 14, 15, 36, 46, 54, 56]. More directly relevant to the subject of this paper, the vectorfield method has also led to numerous developments in the understanding of stability of black hole spacetimes. This includes linearized stability of the Schwarzschild family [16] (see also [38] and [35]), the nonlinear stability of the Schwarzschild family [11, 40], linearized stability of the Kerr family [3], as well as the nonlinear stability of slowly-rotating Kerr [41].

The vectorfield method, as originally conceived, works by exploiting Killing, conformal Killing, and almost-conformal Killing symmetries to define vectorfield multipliers and commutators that can be used to derive various energy estimates. Exploiting symmetries though can only be as strong as one has symmetries to exploit, and in the case of Schwarzschild(-de Sitter) and Kerr(-de Sitter) spacetimes, there are frequently not enough (conformal) Killing vectorfields to exploit directly. To compensate for this lack of symmetries, extensions of the classical vectorfield method have constructed new vectorfields that have coercive deformation tensors on different regions of spacetime, and have extended the method to consider more general Killing tensors [2]. The starting point, and typically the most involved portion of the method is the proof of an *integrated local energy decay* (ILED) estimate, also commonly referred to in the literature as a *Morawetz estimate* [4].

Despite the differences in the origins of the scattering resonance method and the vectorfield method, Morawetz estimates and resolvent estimates are known to be intrinsically tied. On non-trapping asymptotically flat space-times, Metcalfe, Sterbenz, and Tataru have shown an equivalence between resolvent estimates and Morawetz estimates (modulo certain conditions on the real axis) for symmetric wave-type operators [50]. This suggests that despite the initial differences between the approaches to black-hole stability by the scattering resonance and the vectorfield communities, there are actually deep similarities between the two methods. This is further suggested by recent work done by Warnick and Gajic [23, 62] using ideas developed in the new vectorfield method to reinterpret the resonances of the scattering resonance method as true eigenvalues of an operator on a Hilbert space.

Finally, we mention that there has recently been work done by Mavrogiannis using the vectorfield method to prove exponential decay for scalar quasilinear waves on Schwarzschild-de Sitter without reference to spectral theory [48, 49].

### Statement of the Main Result

Motivated by these insights, in this paper and its sequel, we attempt to bridge the gaps between these two methods in a novel proof of the full nonlinear stability of the slowly-rotating Kerr-de Sitter family. Our result aims to use ideas and results from the vectorfield method in order to recover the necessary exponential decay at the linear level to be applied in a full proof of nonlinear stability. The statement that we wish to prove is then as follows (for the more formal statement, see Theorem 5.1).

#### Theorem 1.2

(Main Theorem, version 1) Given initial data satisfying the linearized harmonic gauge constraint, there exists a solution to the linearized Einstein vacuum equations in harmonic gauge that is exponentially decaying to an infinitesimal diffeomorphism of some nearby linearized slowly-rotating Kerr-de Sitter metric $g_{b}'(b')$.

We highlight the main differences between our proof and the original proof for linearized stability of Hintz and Vasy (see Theorem 10.5 of [31]) below.

1. We only use classical pseudo-differential arguments in a neighborhood of trapping in a single part of the derivation of the Morawetz estimate (in Sect. 8.4) to overcome the frequency-dependent nature of the trapped set in Kerr-de Sitter, avoiding all other microlocal arguments and techniques in the rest of the proof. This is in line with the idea that for slowly-rotating Kerr-de Sitter spacetimes, trapping is the only frequency-dependent behavior^4^. It may in fact be possible to remove even this frequency-based argument by following similar methods as [2] to produce a purely physical argument at least in the slowly-rotating case, but this is not pursued further here.
2. By using the vectorfield method, we are able to adapt Warnick’s work on anti-de Sitter spaces [62] to Kerr-de Sitter, resulting in a characterization of quasinormal modes as eigenvalues of an operator on a Hilbert space, thus avoiding the need to construct a meromorphic continuation of the resolvent.
3. We ascertain the “spectral gap” explicitly for the gauge-fixed linearized Einstein equation using a Morawetz estimate, following the approach taken by Tataru and Tohaneanu in [56] to prove a similar Morawetz estimate for scalar waves on Kerr. Doing so allows our proof to remain self-contained while also providing an explicit example of the equivalence between Morawetz estimates and resolvent estimates on a trapping background.
4. We obtain the desired mode stability result (a statement of the non-physical nature of the non-decaying quasinormal mode solutions of the gauge-fixed linearized Einstein operator) by perturbing a geometric mode stability result for Schwarzschild-de Sitter (see Theorem 10.1). This is morally similar to the approach taken by by Hintz and Vasy in [31]. However, we do not introduce constraint damping. Instead, we classify the non-decaying quasinormal modes by a precise analysis of the linearized harmonic coordinate condition and the linearized harmonic-gauge propagation equation induced by the Einstein vacuum equations.

These goals at the linear level will allow us in [22] to give a novel self-contained proof of the full nonlinear stability of the slowly-rotating Kerr-de Sitter family that uses a bootstrapping argument, rather than a Nash-Moser argument to close nonlinear stability.

We provide a brief discussion of the main difficulties involved in achieving the goals of the paper. It is well known that on black hole spacetimes, there tend to be two underlying geometric difficulties standing in the way of a proof of linear stability^5^. The first is the issue of superradiance and the loss of a timelike Killing vector at the horizons. In fact on Kerr-de Sitter spacetimes, much like on its Kerr cousin, there is no global timelike Killing vectorfield on the domain of exterior communication^6^. What has arisen as a powerful solution in the new vectorfield method is defining a new vectorfield that captures exactly within a neighborhood of the horizon, the exponential decay corresponding to the *redshift effect* [13, 14]. The second, and more problematic difficulty is the existence of trapped null geodesics on the interior of the black hole exterior region. Fortunately, on Kerr(-de Sitter), these trapped null geodesics are unstable, in the sense that locally, energy disperses away from the trapped set. This was first observed by in [63] and has played an important role in many subsequent works on wave operators on curved backgrounds [2, 15, 19, 29, 46, 56]. This is the also main idea which we will rely on to prove the desired Morawetz estimates.

Finally, there is the difficulty of proving a mode stability statement. The difficulty in linearized Einstein’s equations, there generally are non-decaying mode solutions. Thus, it is not generally possible to prove complete mode stability (that there are no non-decaying mode solutions). Instead, one wishes to show a geometric mode stability statement: that all non-decaying mode solutions are nonphysical in the sense that they arise out of linearized diffeomorphisms or linearized changes of the black hole parameters. While there is a powerful geometric mode stability (GMS) statement on the non-gauge-fixed linearized Einstein equations linearized around a member of the Schwarzschild-de Sitter family [37, 42, 43], perturbation theory does not directly yield a geometric mode stability statement for even nearby Kerr-de Sitter black holes. This is treated in [31] by introducing constraint damping at the level of the linearized gauge-fixed Einstein’s equations, and then applying perturbation theory. The main idea is that for the linearized gauge-fixed Einstein’s equations, apart from just the geometric non-decaying mode solutions, one also has non-decaying mode solutions arising from a violation of the linearized harmonic coordinate condition. Constraint damping then precisely kills these non-decaying mode solutions so one only has the remaining geometric mode solutions. Rather than introduce constraint damping, we track the non-decaying geometric mode solutions by applying perturbation theory at the level of the gauge-fixed Einstein equations and the harmonic-coordinate constraint propagation equation, which are principally wave and more amenable to perturbative methods. This takes advantage of a bijection between the non-decaying modes of the harmonic-coordinate constraint propagation equation and the geometric non-decaying modes of the linearized gauge-fixed Einstein equations.

### Outline of the Paper

In Sect. 2, we set up the main geometric aspects of the problem. In particular, we construct various regular coordinate systems on the Kerr-de Sitter family, and show the instability of the trapped set. We also identify the vectorfield multipliers that will be used subsequently to prove Killing and redshift energy estimates respectively. In Sect. 3, we then give a brief overview of Einstein’s vacuum equations and harmonic gauge, defining the gauge-fixed linearized Einstein operator that will be the focus of the rest of the paper. We also compute the important properties of the subprincipal symbol at the trapped set and the horizons that will be crucial to the rest of the proof. In Sect. 4, we define the solution semigroup associated to a strongly hyperbolic operator, and use these $C^0$-semigroups to define the quasinormal spectrum. We also in this section define the Laplace-transformed operator which will prove as a useful intermediary in studying the quasinormal spectrum.

Having established the important definitions, we state the main theorem in Sect. 5. We also provide a breakdown of the main intermediary results: exponential decay up to a finite-dimensional perturbation, and geometric mode stability. Section 6 consists of the estimates which are blind to the presence of the trapped set, namely, the standard Killing energy estimate, and (enhanced) redshift estimate, which will serve as the technical basis for proving a Fredholm alternative for the spectrum.

In Sect. 7, we set up the necessary tools for the frequency analysis in Sect. 8. The Morawetz estimate in Sect. 8 is both the most difficult and the most important step to proving exponential decay up to a finite-dimensional perturbation. It requires analyzing both the physical and frequency space behavior of trapping and defining energies that are well-adapted to capturing the behavior at the trapped set. These estimates are the key to proving a spectral gap for the spectrum, and form the core of the paper. In Sect. 9, we show how to use the energy estimates that we have derived in the previous sections to deduce information about the spectrum, in particular, showing that there are only a finite number of spectral obstacles to exponential decay of solutions to the linearized equations.

The mode stability of the gauge-fixed linearized Einstein operator is dealt with in Sect. 10. This is another key component to the proof. We begin with a review of mode stability in Schwarzschild-de Sitter, for which strong results are already known, before showing that the unphysical nature of quasinormal mode solutions is conserved under small perturbations to nearby linearizations of Einstein’s equations around Kerr-de Sitter.

Finally, in Sect. 11, we prove the main theorem, using the tools developed in all the preceding sections.

## Geometric Set Up

In this section, we define key geometric objects that we will make use of later.

### Notational Conventions

Many of the inequalities in this paper feature implicit constants. We use the following notation. Generically, $\epsilon , \delta$, are auxiliary small constants. *C* is used to denote large auxiliary constants. $C(\epsilon )$ (or equivalently $C(\delta )$) indicates a large constant depending monotonically on $\epsilon$ such that $\lim _{\epsilon \rightarrow 0}C(\epsilon ) = \infty$. In particular, inequalities with $C(\epsilon )$ still hold if $C(\epsilon )$ is replaced with a larger constant. Subscripts in constants are then used to denote additional dependencies of the constants.

Throughout the paper, Greek indices will be used to indicate the spacetime indices $\{0,1,2,3\}$, lower-case Latin indices will be used to represent the spatial indices $\{1,2,3\}$, and upper-case Latin indices will be used to represent angular indices. We also denote $\mathbbm {i}{:=} \sqrt{-1}$ to avoid confusion with the index *i*.

We will use the standard musical isomorphism notation to denote $X^\flat$ the canonical one-form associated to a vectorfield *X*, and $\omega ^\sharp$ the canonical vectorfield associated to a one-form $\omega$.

We will use $T^*\mathcal {U}$ and $S^2T^*\mathcal {U}$ to refer to the cotangent bundle and the bundle of symmetric two tensors on $\mathcal {U}$ respectively.

We will use $\nabla$ to denote the full spacetime covariant derivative, and $D = \mathbbm {i}\partial$.

If *M* and *N* are matrices, we write

$$\begin{aligned} M\cdot N = M^TN, \end{aligned}$$

where $M^T$ denotes the transpose of *M*.

### The Kerr-de Sitter Family

The Kerr-de Sitter family of black holes, which will be presented explicitly in what follows, is a family of stationary black hole solutions to the Einstein vacuum equations (EVE) with a positive cosmological constant $\Lambda >0$. The two-parameter family is parameterized by

1. the mass of the black hole *M* and,
2. the angular momentum of the black hole $a = \left\lvert \textbf{a}\right\rvert$, where $\frac{\textbf{a}}{\left\lvert \textbf{a}\right\rvert }$ is the axis of symmetry of the black hole.

We will denote by *B* the set of black-hole parameters (*M*, *a*). In this paper, we will not deal with the full Kerr-de Sitter family, but instead are primarily concerned with only a subfamily characterized by two features: first, that the mass of the black hole is subextremal and satisfies $1-9\Lambda M^2>0$; and that the black hole is slowly rotating, $\frac{\left\lvert a\right\rvert }{M}\sqrt{\Lambda M^2}\ll 1$. We emphasize that the mass of the black hole and the cosmological constant are fixed, whereas *a* is to be interpreted as a smallness constant. The subextremality of the mass ensures that the event horizon and the cosmological horizon remain physically separated, and the slow rotation ensures that the trapped set remains physically separated from both the cosmological and the black-hole ergoregions.

#### The Schwarzschild-de Sitter Metric

Given a cosmological constant $\Lambda$, and a black hole mass $M>0$ such that

2 $$\begin{aligned} 1 - 9\Lambda M^2>0, \end{aligned}$$

we denote by

$$\begin{aligned} b_0 = (M, \textbf{0}) \end{aligned}$$

the black hole parameters for a mass-subextremal Schwarzschild-de Sitter black hole. The Schwarzschild-de Sitter family represents a family of spherical, non-rotating black hole solutions to Einstein’s equations with positive cosmological constant. On the domain of outer communication (also known in the literature as the static region),

3 $$\begin{aligned} \mathcal {M}^\circ {:=} \mathbb {R}_t\times \widetilde{\Sigma }_{t_*}^\circ , \end{aligned}$$

where

4 $$\begin{aligned} \widetilde{\Sigma }_{t_*}{:=} [r_{b,\mathcal {H}^+}, r_{b,\overline{\mathcal {H}}^+}]\times \mathbb {S}^2, \end{aligned}$$

with $r_{b_0,\mathcal {H}^+}$, $r_{b_0, \overline{\mathcal {H}}^+}$ defined below, the Schwarzschild-de Sitter metric can be expressed in standard Boyer-Lindquist coordinates by5where

$$\begin{aligned} \mu _{b_0}(r) = 1 - \frac{2M}{r}- \frac{\Lambda r^2}{3}, \end{aligned}$$

and  denotes the standard metric on $\mathbb {S}^2$. The subextremal mass restriction in (2) guarantees that $\mu _{b_0}(r)$ has three roots: two positive simple roots, and one negative simple root,

$$\begin{aligned} r_{b_0,-}<0<r_{b_0,\mathcal {H}^+}<r_{b_0,\overline{\mathcal {H}}^+}<\infty . \end{aligned}$$

The *r*-constant hypersurfaces defined by $\{r = r_{b_0, \mathcal {H}^+}\}, \{r = r_{b_0, \overline{\mathcal {H}}^+}\}$ are respectively the *(future) event horizon* and the *(future) cosmological horizon*, and bound the domain of outer communications, $\mathbb {R}_t^+\times (r_{b_0, \mathcal {H}^+}, r_{b_0, \overline{\mathcal {H}}^+})\times \mathbb {S}^2$, on which the form of the metric in (5) is valid.

The form of the metric $g_{b_0}$ in Boyer-Lindquist coordinates has one major short-coming, namely, its apparent singularity when $\mu _{b_0}=0$, which occurs exactly at the event horizon and the cosmological horizon. Fortunately, this is only a coordinate singularity, and can be resolved by a change of coordinates. We present two candidates in the following section.

#### Regular Coordinates on Schwarzschild-de Sitter

We now construct some coordinate systems on Schwarzschild-de Sitter that are regular at the horizons, and will be used in calculations throughout the rest of the paper. To fix this, we introduce the change of coordinates

$$\begin{aligned} t_*= t - F_{b_0}(r), \end{aligned}$$

where $F_{b_0}$ is a smooth function of *r*. In the coordinates $(t_*, r, \omega )$, the Schwarzschild-de Sitter metric, $g_{b_0}$, and the inverse metric, $G_{b_0}$, take the form6We will require two main conditions be satisfied by the new $(t_*, r, \omega )$ coordinate system: that the $t_*$-constant hypersurfaces are uniformly spacelike even slightly beyond the horizons, and that in a neighborhood of the trapped set, the new coordinate system is identical to the Boyer-Lindquist coordinate system $(t,r,\omega )$.

##### Lemma 2.1

Fix some interval $\mathcal {I}_{b_0}\subset (r_{b_0,\mathcal {H}^+}, r_{b_0,\overline{\mathcal {H}}^+})$. Then there exists a choice of $F_{b_0}(r)$ such that

1. the $t_*$-constant hypersurfaces are space-like. That is, that
  $$\begin{aligned} -\frac{1}{\mu _{b_0}} + F_{b_0}'(r)^2\mu _{b_0} < 0; \end{aligned}$$
2. $$F_{b_0}(r)$$ satisfies that
  $$\begin{aligned} F_{b_0}(r) \ge 0,\qquad r\in (r_{b_0,\mathcal {H}^+},r_{b_0,\overline{\mathcal {H}}^+}), \end{aligned}$$
  with equality for $r\in \mathcal {I}_{b_0}$.

##### Proof

See Appendix B.1. $$\square $$

Crucially, we can extend $F_{b_0}$ in an arbitrary manner smoothly beyond the horizons $\mathcal {H}^+$, $\overline{\mathcal {H}}^+$. This allows us to consider the extended domain

7 $$\begin{aligned} \mathcal {M}&{:=} \mathbb {R}_{t_*} \times (r_{b_0, \mathcal {H}^+} - \varepsilon _{\mathcal {M}}, r_{b_0, \overline{\mathcal {H}}^+} + \varepsilon _{\mathcal {M}}) \times \mathbb {S}^2, \end{aligned}$$

8 $$\begin{aligned} \Sigma&{:=} (r_{b_0, \mathcal {H}^+} - \varepsilon _{\mathcal {M}}, r_{b_0, \overline{\mathcal {H}}^+} + \varepsilon _{\mathcal {M}}) , \end{aligned}$$

for some $\varepsilon _{\mathcal {M}}>0$ small, on which $g_{b_0}$ as defined by the expression in (6) is a smooth Lorentzian metric satisfying Einstein’s equations. We also define

9 $$\begin{aligned} \mathcal {H}^+_-{:=}\left\{ r=r_{b_0, \mathcal {H}^+} - \varepsilon _{\mathcal {M}}\right\} ,\qquad \overline{\mathcal {H}}^+_+{:=}\left\{ r=r_{b_0, \overline{\mathcal {H}}^+} + \varepsilon _{\mathcal {M}}\right\} . \end{aligned}$$

**Fig. 1:**
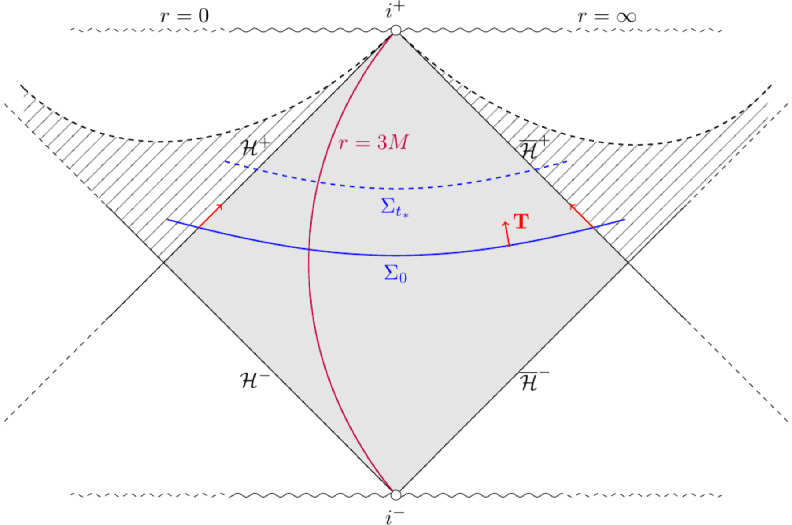
Penrose diagram of Schwarzschild-de Sitter space. The solid gray region represents the domain of outer communication, in which the metric in (5) is valid. The gray dashed regions beyond the horizons represent the extent to which we can smoothly extend the metric in (5) by the regular coordinates in Lemma 2.1. Here, $\mathcal {H}^{\pm }$ and $\overline{\mathcal {H}}^{\pm }$ denote the future/past event and cosmological horizons respectively, and $i^\pm$ denotes future/past timelike infinity. We have also highlighted two level sets of the $t_*$ coordinate, the photon sphere $r=3M$, and the Killing vectorfield $\textbf{T}$, which is future-oriented timelike on the domain of outer communication, and null on the horizons

It will often be useful to consider the outgoing and ingoing Eddington-Finkelstein coordinates given by the case where $F_{b_0}'(r) = \pm \mu _{b_0}(r)^{-1}$ respectively for specific calculations (Fig. 1). In this case, the Schwarzschild-de Sitter metric and inverse metric are10

#### The Kerr-de Sitter Metric

**Fig. 2:**
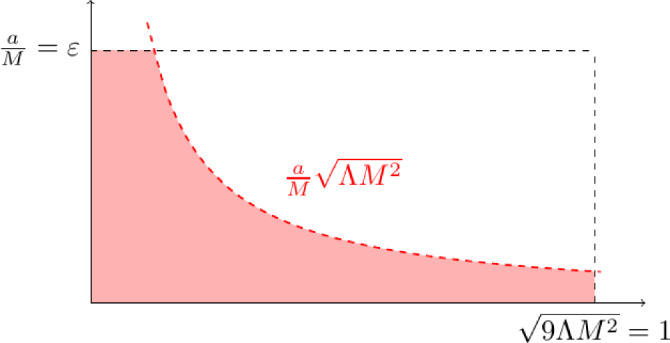
A graphical depiction of the slowly rotating regime. Here $\varepsilon \ll 1$. The most convenient way to think about the slowly-rotating regime in this paper is to consider $M, \Lambda$ fixed, and to think of the slowly-rotating regime as when $\frac{a}{M}$ is sufficiently small, depending on $\Lambda M^2$

The Schwarzschild-de Sitter family represents a stationary, spherically symmetric family of black-hole solutions to Einstein’s equations. On the other hand, the Kerr-de Sitter family represents a stationary, *axi-symmetric*, family of black-hole solutions to Einstein’s equations, of which the Schwarzschild-de Sitter family is a sub-family. In this section, we detail various useful coordinate systems that we use subsequently. Throughout the paper, we are mainly interested in Kerr-de Sitter metrics that are slowly-rotating, i.e. that^7^$a = \left\lvert \textbf{a}\right\rvert \ll \frac{1}{\sqrt{\Lambda }} = \frac{M}{\sqrt{\Lambda }M^2}, M$, and thus close to a Schwarzschild-de Sitter relative (Fig. 2).

##### Definition 1

In the Boyer-Lindquist coordinates $(t, r, \theta , \varphi )\in \mathbb {R}\times (r_{b, \mathcal {H}^+}, r_{b, \overline{\mathcal {H}}^+})\times (0,\pi )\times \mathbb {S}^1_\varphi$ (with $r_{b, \mathcal {H}^+}$, and $r_{b, \overline{\mathcal {H}}^+}$ defined below), the Kerr-de Sitter metric $g_b{:=}g(M,\textbf{a})$, and inverse metric $G_b{:=} G(M, \textbf{a})$ take the form:

11 $$\begin{aligned} g_b ={}&\rho _b^2\left( \frac{dr^2}{\Delta _b} + \frac{d\theta ^2}{\varkappa _b}\right) + \frac{\varkappa _b\sin ^2\theta }{(1+\lambda _b)^2\rho _b^2 }\left( a\,dt- (r^2+a^2)\,d\varphi \right) ^2\nonumber \\&- \frac{\Delta _b}{(1+\lambda _b)^2\rho _b^2}(dt-a\sin ^2\theta \,d\varphi )^2,\nonumber \\ G_{b} ={}&\frac{1}{\rho _b^2}\left( \Delta _{b}\partial _r^2 + \varkappa _b\partial _\theta ^2\right) + \frac{(1+\lambda _b)^2}{\rho _b^2\varkappa _b\sin ^2\theta }\left( a\sin ^2\theta \partial _{t} + \partial _\varphi \right) ^2\nonumber \\&- \frac{(1+\lambda _b)^2}{\Delta _b\rho _b^2}\left( \left( r^2+a^2\right) \partial _t + a\partial _\varphi \right) ^2, \end{aligned}$$

where

$$\begin{aligned} \Delta _b {:=} (r^2+a^2)\left( 1-\frac{1}{3}\Lambda r^2\right) - 2M r,\qquad \rho _b^2 {:=} r^2+a^2\cos ^2\theta , \\ \lambda _b {:=}\frac{1}{3}\Lambda a^2,\qquad \varkappa _b{:=}1+\lambda _b \cos ^2\theta . \end{aligned}$$

##### Remark 1

It is easy to observe that the metric reduces to the Schwarzschild-de Sitter metric $g_{b_0}$ expressed in Boyer-Lindquist coordinates in (5) when $b=b_0$ ($a=0$). When $a\ne 0$, the spherical coordinates $(\theta , \varphi )$ are chosen so that $\frac{\textbf{a}}{\left\lvert \textbf{a}\right\rvert }\in \mathbb {S}^2$ is defined by $\theta =0$, and $\partial _{\varphi }$ generates counter-clockwise rotation around the axis of rotation.

##### Definition 2

As in the Schwarzschild-de Sitter case, we define the *event horizon* and the *cosmological horizon* of $g_{b}$, denoted by $\mathcal {H}^+$, $\overline{\mathcal {H}}^+$ to be the *r*-constant hypersurfaces

$$\begin{aligned} \mathcal {H}^+{:=}\{r=r_{b, \mathcal {H}^+}\},\qquad \overline{\mathcal {H}}^+{:=}\{r=r_{b, \overline{\mathcal {H}}^+}\}, \end{aligned}$$

respectively, where $r_{b, \mathcal {H}^+} < r_{b,\overline{\mathcal {H}}^+}$ are the two largest distinct positive roots of $\Delta _b$.

A consequence of the implicit function theorem is that these roots depend smoothly on the black hole parameters $b=(M, a)$. Since these two horizons are null, the domain of exterior communications, bounded by $r_{b, \mathcal {H}^+}, r_{b, \overline{\mathcal {H}}^+}$ is a causal domain that is fo+liated by compact space-like hypersurfaces. This point is used in a crucial way throughout what follows.

Much like the case in Schwarzschild-de Sitter, the Boyer-Lindquist form of the Kerr-de Sitter metric $g_b$ in (11) has a singularity at both the event horizon and the cosmological horizon. As we have already discussed for the Schwarzschild-de Sitter case, this is merely a coordinate singularity, and it is possible to construct a smooth coordinate system that extends beyond the horizons.

We construct such a new, Kerr-star, coordinate system explicitly (see similar constructions in [13, Section 5.5], [56, Section 4], [31, Section 3.2]). First define the new variables

12 $$\begin{aligned} t_*= t - F_b(r),\quad {\varphi _*}= \varphi - \Phi _b(r), \end{aligned}$$

where $F_b$ and $\Phi _b$ are smooth functions on $(r_{b_0, \mathcal {H}^+} + \varepsilon _{\mathcal {M}}, r_{\overline{\mathcal {H}}^+} - \varepsilon _{\mathcal {M}})$. We can then compute that the metric takes the form

13 $$\begin{aligned} g_b =&{} \frac{\varkappa _b \sin ^2\theta }{(1+\lambda _b)^2\rho _b^2} \left( a(dt_*+ F_b'\,dr) - (r^2+a^2)(d{\varphi _*}+ \Phi _b'\,dr) \right) ^2\nonumber \\&- \frac{\Delta _b}{(1+\lambda _b)\rho _b^2}\left( dt_*+ F_b'\,dr - a \sin ^2\theta (d{\varphi _*}+ \Phi _b'\,dr) - \frac{(1+\lambda _b)\rho _b^2}{\Delta _b} \,dr \right) ^2\nonumber \\&+ \frac{2}{1+\lambda _b}\left( dt_*+ F_b'\,dr - a \sin ^2\theta (d{\varphi _*}+ \Phi _b'\,dr) - \frac{(1+\lambda _b)\rho _b^2}{\Delta _b} \,dr \right) dr \nonumber \\&\quad +\frac{\rho _b^2}{\varkappa _b}\,d\theta ^2. \end{aligned}$$

We pick the $F_b, \Phi _b$ so that the $(t_*,r,\theta ,{\varphi _*})$ coordinate system extends smoothly beyond the horizons, is identical to the Boyer-Lindquist coordinates on a small neighborhood of $r=3M$, and such that the $t_*$-constant hypersurfaces are spacelike.

##### Lemma 2.2

Fix an interval $\mathcal {I}_b{:=} (r_1, r_2)$ such that $r_{b_0,\mathcal {H}^+}+\epsilon _{\mathcal {M}}<r_1<r_2<r_{b_0,\overline{\mathcal {H}}^+} - \epsilon _{\mathcal {M}}$. Then we can pick $F_b, \Psi _b$ so that

1. the choice extends the choice of regular coordinates for Schwarzschild-de Sitter in (6) in the sense that when $b=b_0$,
  $$\begin{aligned} F_{b} = F_{b_0},\qquad \Phi _b = 0, \end{aligned}$$
  and moreover $F_b$, $\Phi _b$ are smooth in *a*;
2. $F_b(r) \ge 0$ for $r\in (r_{b, \mathcal {H}^+}, r_{b, \overline{\mathcal {H}}^+})$ with equality for $r\in \mathcal {I}_b$;
3. the $t_*$-constant hypersurfaces are space-like, and in particular, defining
  14 $$\begin{aligned} A_b{:=} - \frac{1}{G_b(dt_*, dt_*)}, \end{aligned}$$
  we have that
  15 $$\begin{aligned} 1\lesssim A_b \lesssim 1 \end{aligned}$$
  uniformly on $$\mathcal {M}$$;
4. the metric $g_b$ is smooth on $\mathcal {M}$.

##### Proof

See Appendix B.2. $$\square $$

It will often be convenient to perform calculations on Kerr-de Sitter with the choice of

$$\begin{aligned} F_{b}'(r) = \pm \frac{(1+\lambda _b)(r^2+a^2)}{\Delta _b}, \qquad \Phi _b'(r) = \pm \frac{(1+\lambda _b)a}{\Delta _b}, \end{aligned}$$

which correspond to the outgoing and ingoing Eddington-Finkelstein coordinates, in which case the Kerr-de Sitter metric and inverse metric can be expressed in the coordinates $(t_0, r, \theta , \varphi _0)$ by

16 $$\begin{aligned} g_b\!=\!{}&\frac{\varkappa _b\sin ^2\theta }{(1\!+\!\lambda _b)^2\rho _b^2}(ad{t_0}\!-\!(r^2\!+\!a^2)d{\varphi _0})^2 \!-\! \frac{\Delta _b}{(1\!+\!\lambda _b)^2\rho _b^2}\left( d{t_0}\!-\!a(\sin ^2\theta )d{\varphi _0}\right) ^2 \nonumber \\&\pm \frac{2}{(1+\lambda _b)}(d{t_0}- a(\sin ^2\theta )d{\varphi _0})\,dr + \frac{\rho _b^2}{\varkappa _b}d\theta ^2, \end{aligned}$$

17 $$\begin{aligned} \rho _b^2G_b ={}&\Delta _b\left( \partial _r \mp \frac{1+\lambda _b}{\Delta _b}\left( (r^2+a^2)\partial _{t_0}+a\partial _{\varphi _0}\right) \right) ^2\nonumber \\&-\frac{(1+\lambda _b)^2}{\Delta _b}\left( (r^2+a^2)\partial _{t_0} + a\partial _{\varphi _0}\right) ^2 + \rho _b^2\mathcal {O}, \\ \rho _b^2\mathcal {O}\!=\!{}&\varkappa _b\partial _{\theta }^2\!+\!\frac{(1\!+\!\lambda _b)^2}{\varkappa _b\sin ^2\theta }\left( a\sin ^2\theta \partial _{t_0}\!+\!\partial _{\varphi _0}\right) ^2 \nonumber . \end{aligned}$$

In the Eddington-Finkelstein coordinates, the Kerr-de Sitter metric can be written in a more condensed format.

##### Definition 3

Define the vectorfields

18 $$\begin{aligned} \begin{aligned} \widehat{R}= {\left\{ \begin{array}{ll} \partial _r - \frac{1}{\Delta _b}\widehat{T}, &  r> 3M,\\ \partial _r + \frac{1}{\Delta _b}\widehat{T}, &  r< 3M, \end{array}\right. }&\qquad \widehat{T}= (1+\lambda _b)\left( (r^2+a^2)\partial _{t_0} + a\partial _{\varphi _0}\right) ,\\ \widehat{R}' = {\left\{ \begin{array}{ll} \Delta _b^{-2}\partial _r\Delta _b\widehat{T}-\frac{1}{\Delta _b}\widehat{T}', &  r>3M,\\ -\Delta _b^{-2}\partial _r\Delta _b\widehat{T}+\frac{1}{\Delta _b}\widehat{T}', &  r<3M, \end{array}\right. }&\qquad \widehat{T}' = 2(1+\lambda _b)r\partial _{t_0}. \end{aligned} \end{aligned}$$

Notice that we can then write,

19 $$\begin{aligned} \rho _b^2 G_b = -\frac{1}{\Delta _b}\widehat{T}^2 + \Delta _b\widehat{R}^2 + \rho _b^2\mathcal {O}. \end{aligned}$$

We will also define the operator

20 $$\begin{aligned} \rho \widehat{\mathcal {O}}h {:=} \left( \varkappa ^2\left\lvert \partial _\theta h\right\rvert ^2 + \frac{(1+\lambda )^2}{\varkappa \sin ^2\theta }\left\lvert \left( a\sin ^2\theta \partial _{t_0} + \partial _{\varphi _0}\right) h\right\rvert ^2\right) ^{\frac{1}{2}}, \end{aligned}$$

defined so that

$$\begin{aligned} \rho ^2\left\lvert \widehat{\mathcal {O}}h\right\rvert ^2 = \mathcal {O}^{\alpha \beta }\partial _\alpha h \partial _\beta h. \end{aligned}$$

##### Remark 2

Observe that $\widehat{R}$ and $\widehat{R}'$ as defined in (18) are not smooth vectorfields, since they have a singularity at $r=3M$. This is fortunately not an issue for us, since everywhere that we use $\widehat{R}$ and $\widehat{R}'$, we will be restricted to a subset of $\mathcal {M}$ localized away from $r=3M$.

### The Killing Vectorfields $\textbf{T}$, ${\Phi }$

#### Definition 4

Define the vectorfields $\textbf{T}= \partial _{t_*}$, ${\Phi }=\partial _{{\varphi _*}}$ using the Kerr-star coordinates in (13). From the fact that the expression of $g_b$ in the Kerr-star coordinates is independent of $t_*$ and ${\varphi _*}$, we immediately have that $\textbf{T}, {\Phi }$ are Killing vectorfields.

#### Definition 5

On Kerr-de Sitter spacetimes, the *ergoregion* is defined by

$$\begin{aligned} \mathcal {E}{:=} \{(t,x): g_b(\textbf{T},\textbf{T})(t,x) > 0\}. \end{aligned}$$

We define the boundary of the ergoregion, the set of points where $\textbf{T}$ is null, as the *ergosphere*.

#### Remark 3

Observe that for the Schwarzschild-de Sitter sub-family, the ergosphere is exactly the event horizon and the cosmological horizon, and $\textbf{T}$ is timelike on the whole of the interior of the domain of outer communication.

The following lemma shows that on slowly-rotating Kerr-de Sitter spacetimes, the ergoregion consists of two components, $\mathcal {E}_{\mathcal {H}^+}$, which contains $\mathcal {H}^+$, and $\overline{\mathcal {H}}^+$, which contains $\mathcal {E}_{\overline{\mathcal {H}}^+}$.

#### Lemma 2.3

For any fixed $\delta _{\mathcal {H}}\ll 1$, let $\mathcal {B}_{\delta _{\mathcal {H}}}$ be the set of Kerr-de Sitter black hole parameters such that

$$\begin{aligned} \left\lvert \sup _{\mathcal {E}_{\mathcal {H}^+}}r - r_{b_0,\mathcal {H}^+}\right\rvert + \left\lvert \inf _{\mathcal {E}_{\overline{\mathcal {H}}^+}}r - r_{b_0, \overline{\mathcal {H}}^+}\right\rvert \le \delta _{\mathcal {H}}, \end{aligned}$$

so that for $b\in \mathcal {B}_{\delta _{\mathcal {H}}}$, the two components of the ergoregion $\mathcal {E}_{\mathcal {H}^+}$ and $\mathcal {E}_{\overline{\mathcal {H}}^+}$ are physically separated and lie within a small neighborhood of the event and cosmological horizons respectively. Then, for $\left\lvert a\right\rvert \ll \frac{1}{\sqrt{\Lambda }}, M$ and $1-9\Lambda M^2 > 0$, $b\in \mathcal {B}_{\delta _{\mathcal {H}}}$.

#### Proof

In view of Remark 3, we have that

$$\begin{aligned} \sup _{\mathcal {E}_{\mathcal {H}^+}}r = r_{b_0,\mathcal {H}^+}, \quad \inf _{\mathcal {E}_{\overline{\mathcal {H}}^+}}r = r_{b_0, \overline{\mathcal {H}}^+}, \end{aligned}$$

and thus

$$\begin{aligned} \lim _{a\rightarrow 0}\sup _{\mathcal {E}_{\mathcal {H}^+}}r - r_{b_0, \mathcal {H}^+} = \lim _{a\rightarrow 0} \inf _{\mathcal {E}_{\overline{\mathcal {H}}^+}}r - r_{b_0, \overline{\mathcal {H}}^+} = 0, \end{aligned}$$

directly yielding the conclusion. $\square$

### The Horizon Generators $\textbf{K}_{\mathcal {H}^+}, \textbf{K}_{\overline{\mathcal {H}}^+}$

#### Definition 6

In Kerr-de Sitter spacetimes, the horizons are generated by the following Killing *horizon generators*, which are null on their respective horizons, and time-like in a neighborhood of their respective horizons:

$$\begin{aligned} \textbf{K}_{\mathcal {H}^+} {:=} \textbf{T}+ \frac{a}{r_{\mathcal {H}^+}^2 +a^2}{\Phi }, \qquad \textbf{K}_{\overline{\mathcal {H}}^+} {:=} \textbf{T}+ \frac{a}{r_{\overline{\mathcal {H}}^+}^2 + a^2}{\Phi }. \end{aligned}$$

Let us denote also

$$\begin{aligned} \Omega _{\mathcal {H}^+} {:=} \frac{a}{r_{\mathcal {H}^+}^2 + a^2}, \qquad \Omega _{\overline{\mathcal {H}}^+} {:=} \frac{a}{r_{\overline{\mathcal {H}}^+}^2 + a^2}. \end{aligned}$$

In particular, in the Schwarzschild-de Sitter subfamily, $\textbf{K}_{\mathcal {H}^+} = \textbf{K}_{\overline{\mathcal {H}}^+} = \textbf{T}$.

#### Definition 7

Associated to each horizon is a *surface gravity*, $\kappa _{\mathcal {H}}$, defined by

21 $$\begin{aligned} \nabla _{\textbf{K}_{\mathcal {H}}} \textbf{K}= \kappa _{\mathcal {H}} \textbf{K}. \end{aligned}$$

On Schwarzschild-de Sitter, the values for the black hole surface gravity and the cosmological surface gravity respectively are

$$\begin{aligned} \kappa _{b_0,\mathcal {H}^+} = \frac{1}{2}\partial _r\mu _{b_0}(r_{\mathcal {H}^+}),\qquad \kappa _{b_0,\overline{\mathcal {H}}^+} = -\frac{1}{2}\partial _r\mu _{b_0}(r_{\overline{\mathcal {H}}^+}). \end{aligned}$$

Notice that $\kappa _{b_0,\mathcal {H}^+}, \kappa _{b_0,\overline{\mathcal {H}}^+}>0$, and that this positivity persists in the Kerr-de Sitter case, where

$$\begin{aligned} \kappa _{\mathcal {H}^+}\!=\!\frac{r_{\mathcal {H}^+}\left( 1\!-\!\frac{2}{3}\Lambda r_{\mathcal {H}^+}^2\!-\!\frac{\Lambda }{3}a^2 \right) \!-\!M}{r_{\mathcal {H}^+}^2\!+\!a^2},\qquad \!-\!\kappa _{\overline{\mathcal {H}}^+} \!= \!\frac{r_{\overline{\mathcal {H}}^+}\left( 1\!-\!\frac{2}{3}\Lambda r_{\overline{\mathcal {H}}^+}^2\! -\! \frac{\Lambda }{3}a^2 \right) \!-\! M}{r_{\overline{\mathcal {H}}^+}^2 \!+\! a^2}. \end{aligned}$$

### Energy Momentum Tensor and Divergence Formulas

In this section, we define the energy momentum tensor and some basic divergence properties. Given a complex matrix-valued function *h* on $\mathcal {M}$, let us denote its complex conjugate by $\overline{h}$. Moreover, for any 2-tensor $h_{\mu \nu }$, we denote its symmetrization

$$\begin{aligned} h_{(\mu \nu )} = \frac{1}{2}\left( h_{\mu \nu } + h_{\nu \mu } \right) . \end{aligned}$$

#### Definition 8

We define the *energy-momentum tensor* to be the symmetric 2-tensor:

$$\begin{aligned} \mathbb {T}_{\mu \nu }[h] = \nabla _{(\mu }\overline{h}\cdot \nabla _{\nu )}h - \frac{1}{2}g_{\mu \nu }\nabla _\alpha \overline{h}\cdot \nabla ^\alpha h. \end{aligned}$$

The energy-momentum tensor satisfies the following divergence property:22where we denote by

$$\begin{aligned} \Box _{g} = \nabla ^\alpha \partial _\alpha \end{aligned}$$

the scalar wave operator^8^.

This property will be the key to producing the various divergence equations we use to derive the relevant energy estimates in the subsequent sections.

#### Definition 9

Let *X* be a smooth vectorfield on $\mathcal {M}$, *m* be a smooth one-form on $\mathcal {M}$, and *q* be a smooth function on $\mathcal {M}$. We will refer to *X* as the *(vectorfield) multiplier*, to *m* as the *auxiliary zero-order corrector*, and to *q* as the *Lagrangian corrector*. Then define23wheredenotes the *deformation tensor* of *X*.

In proving energy estimates, we will typically apply the divergence formulas in Proposition 2.4 and Corollary 2.6 in the form

24 $$\begin{aligned} \nabla _g\cdot J^{X,q,m}[{h}] = \Re \left[ (X+q)\overline{{h}} \cdot \Box _{g}{h}\right] + K^{X,q,m}[{h}]. \end{aligned}$$

It will also be convenient to define the Laplace-transformed analogues of $J^{X,q,m}[h]$, $K^{X,q,m}[h]$.

#### Definition 10

Let *X* be a smooth vectorfield on $\mathcal {M}$, *m* be a smooth one-form on $\mathcal {M}$, and *q* be a smooth function on $\mathcal {M}$, and let $u = u(x)$. Then we define

25 $$\begin{aligned} \begin{aligned} \widehat{J}(\sigma )^{X,q,m}[u]&= e^{-2\Im \sigma t_*}J^{X,q,m}\left[ e^{-\mathbbm {i}\sigma t_*}u\right] \\ \widehat{K}(\sigma )^{X,q,m}[u]&= e^{-2\Im \sigma t_*}K^{X,q,m}\left[ e^{-\mathbbm {i}\sigma t_*}u\right] , \end{aligned} \end{aligned}$$

which are both $t_*$-independent.

Throughout the paper, we will use the following consequence of the divergence theorem.

#### Proposition 2.4

Let *X* denote a sufficiently regular vectorfield on $\mathcal {M}$, a Kerr-de Sitter black hole spacetime, and $\mathcal {D}$ denote the spacetime region bounded by $\Sigma _{t_1}, \Sigma _{t_2}, \mathcal {H}^+_-$, and $\overline{\mathcal {H}}^+_+$. Moreover, denote

$$\begin{aligned} \mathcal {H}^+_{t_1,t_2} {:=} \mathcal {H}^+_-\bigcap \{t_1\le t_*\le t_2\},\qquad \overline{\mathcal {H}}^+_{t_1,t_2} {:=} \overline{\mathcal {H}}^+_+\bigcap \{t_1\le t_*\le t_2\}. \end{aligned}$$

Then we have the following divergence property:

$$\begin{aligned} -\int _{\mathcal {D}} \nabla _g\cdot X ={}\int _{\Sigma _{t_2}}X\cdot n_{\Sigma _{t_2}} - \int _{\Sigma _{t_1}}X\cdot n_{\Sigma _{t_1}} + \int _{\mathcal {H}^+_{t_1,t_2}} X \cdot n_{\mathcal {H}^+_-} + \int _{\overline{\mathcal {H}}^+_{t_1,t_2}} X\cdot n_{\overline{\mathcal {H}}^+_+}. \end{aligned}$$

Here, $n_{\Sigma _t}$ is the future-directed unit normal on $\Sigma _t$, and $n_{\mathcal {H}}$ denotes the (time-like) future-directed unit normal to $\mathcal {H}$.

It will also be convenient to apply the divergence property to a spacetime domain with boundaries along $\mathcal {H}^+$ and $\overline{\mathcal {H}}^+$.

#### Proposition 2.5

Let *X* denote a sufficiently regular vectorfield on $\mathcal {M}$, a Kerr-de Sitter black hole spacetime, and $\mathcal {D}$ denote the spacetime region bounded by $\widetilde{\Sigma }_{t_1}, \widetilde{\Sigma }_{t_2}, \mathcal {H}^+$, and $\overline{\mathcal {H}}^+$. Moreover, denote

$$\begin{aligned} \mathcal {H}^+_{t_1,t_2} {:=} \mathcal {H}^+\bigcap \{t_1\le t_*\le t_2\},\qquad \overline{\mathcal {H}}^+_{t_1,t_2} {:=} \overline{\mathcal {H}}^+\bigcap \{t_1\le t_*\le t_2\}. \end{aligned}$$

Then we have the following divergence property:

$$\begin{aligned} -\int _{\mathcal {D}} \nabla _g\cdot X ={}\int _{\widetilde{\Sigma }_{t_2}}X\cdot n_{\widetilde{\Sigma }_{t_2}} - \int _{\widetilde{\Sigma }_{t_1}}X\cdot n_{\widetilde{\Sigma }_{t_1}} + \int _{\mathcal {H}^+_{t_1,t_2}} X \cdot \textbf{K}_{\mathcal {H}^+} + \int _{\overline{\mathcal {H}}^+_{t_1,t_2}} X\cdot \textbf{K}_{\overline{\mathcal {H}}^+} . \end{aligned}$$

Here, $n_{\widetilde{\Sigma }_t}$ is the future-directed unit normal on $\widetilde{\Sigma }_t$, and we recall from Sect. 2.4 that $\textbf{K}_{\mathcal {H}}$ are the Killing null generators of $\mathcal {H}$.

#### Remark 4

Observe that the volume form over the horizons can be computed as follows.

$$\begin{aligned} d\mu _{\mathcal {H}^+_{t_1,t_2}}= \sqrt{\det g_{AB}}\,dt_*d\theta d{\varphi _*}, \qquad g_{AB} = \begin{pmatrix} g_{\theta \theta } &  g_{\theta {\varphi _*}}\\ g_{{\varphi _*}\theta } &  g_{{\varphi _*}{\varphi _*}} \end{pmatrix}\Bigg \vert _{\mathcal {H}^+} , \end{aligned}$$

with an analogous formula over the cosmological horizon.

For some of the estimates in this paper, the following analogue of Propositions 2.4 and 2.5 will be more useful.

#### Corollary 2.6

Suppose *X* is a vectorfield on a Kerr-de Sitter spacetime *g*. Then, for sufficiently regular *X*, the following relations hold:

$$\begin{aligned} -\int _{\Sigma _{t_*}}\nabla _g\cdot X\, \sqrt{A}&= \partial _{t_*}\int _{\Sigma _{t_*}}X \cdot n_{\Sigma _{t_*}} + \int _{\overline{\mathcal {H}}^+_+\bigcap \Sigma _{t_*}} X \cdot n_{\overline{\mathcal {H}}^+_+} +\int _{\mathcal {H}^+_-\bigcap \Sigma _{t_*}}X \cdot n_{\mathcal {H}^+_-},\\ -\int _{\widetilde{\Sigma }_{t_*}}\nabla _g\cdot X\, \sqrt{A}&= \partial _{t_*}\int _{\widetilde{\Sigma }_{t_*}}X \cdot n_{\widetilde{\Sigma }_{t_*}} + \int _{\overline{\mathcal {H}}^+\bigcap \widetilde{\Sigma }_{t_*}} X \cdot \textbf{K}_{\overline{\mathcal {H}}^+} +\int _{\mathcal {H}^+\bigcap \widetilde{\Sigma }_{t_*}}X \cdot \textbf{K}_{\mathcal {H}^+}, \end{aligned}$$

where we recall $A$ as defined in (14).

#### Proof

This follows immediately from Proposition 2.4 and the co-area formula. $\square$

By combining Proposition 2.4 and Corollary 2.6 with the divergence relation (24), we will have the divergence relation over a spacetime domain $\mathcal {D}= [t_1,t_2]_{t_*}\times \Sigma$.

26 $$\begin{aligned}&\Re \int _{\mathcal {D}} (X + q)\overline{{h}}\cdot \Box _{g_{b}}{h} + \int _{\mathcal {D}} K^{X,q,m}[{h}]\nonumber \\ ={}&-\int _{\Sigma _{{t}_2}} J^{X,q,m}[{h}]\cdot n_{\Sigma _{{t}_2}} + \int _{\Sigma _{{t}_1}} J^{X,q,m}[{h}]\cdot n_{\Sigma _{{t}_1}}\nonumber \\&- \int _{\mathcal {H}^+_{t_1,t_2}} J^{X,q,m}[{h}]\cdot n_{\mathcal {H}^+} - \int _{\overline{\mathcal {H}}^+_{t_1,t_2}} J^{X,q,m}[{h}]\cdot n_{\overline{\mathcal {H}}^+}; \end{aligned}$$

and its equivalent formulation over a space-like slice

27 $$\begin{aligned}&\Re \int _{\Sigma _{t_*}}(X+q)\overline{{h}}\cdot \Box _{g_{b}}{h} \,\sqrt{A} + \int _{\Sigma _{t_*}}K^{X,q,m}[{h}]\,\sqrt{A}\nonumber \\ ={}&- \partial _{t_*}\int _{\Sigma _{t_*}}J^{X,q,m}[{h}] \cdot n_{\Sigma _{t_*}} - \int _{\mathcal {H}^+\bigcap \Sigma _{t_*}}J^{X,q,m}[{h}]\cdot n_{\mathcal {H}^+}\nonumber \\&- \int _{\overline{\mathcal {H}}^+\bigcap \Sigma _{t_*}} J^{X,q,m}[{h}] \cdot n_{\overline{\mathcal {H}}^+}. \end{aligned}$$

Analogous statements as above hold for the case $\mathcal {D}= [t_1,t_2]_{t_*}\times \widetilde{\Sigma }$.

### The Redshift Vectorfields $\textbf{N}$

In this subsection, we recall the construction of the redshift vectorfield $\textbf{N}$.

#### Proposition 2.7

Let $b=(M, a)$, $\left\lvert a\right\rvert \ll M, \frac{1}{\sqrt{\Lambda }}$ be the black hole parameters for a slowly-rotating Kerr-de Sitter black hole, and let $\Sigma$ be a $t_*$-constant uniformly spacelike hypersurface. Moreover, fix some vectorfield *X* which is tangent to both $\mathcal {H}^+$ and $\overline{\mathcal {H}}^+$, and some $\varepsilon _{\textbf{N}}>0$. Then, there exist a stationary time-like vectorfield $\textbf{N}$, positive constants $c_{\textbf{N}}$ and $C_{\textbf{N}}$, and parameters $r_{\mathcal {H}^+}< r_0<r_1<R_1<R_0< r_{\overline{\mathcal {H}}^+}$ such that the following conditions are fulfilled.

1. On $$r\le r_0$$ or $$r>R_0$$,
  28 $$\begin{aligned} K^{\textbf{N}, 0, 0}[h] \sqrt{A}\ge \left( \kappa _{\mathcal {H}}-\varepsilon _{\textbf{N}}\right) J^{\textbf{N}, 0, 0}[h]\cdot n_{\Sigma } + \left\lvert X h\right\rvert ^2, \end{aligned}$$
  where $\mathcal {H}= \mathcal {H}^+$ for $r\le r_0$, and $\mathcal {H}= \overline{\mathcal {H}}^+$ for $r>R_0$
2. For $$r_0\le r\le R_0$$,
  29 $$\begin{aligned} -K^{\textbf{N}, 0, 0}[{h}] \le C_{\textbf{N}} J^{\textbf{N}, 0, 0}[{h}]\cdot n_{\Sigma }. \end{aligned}$$
3. For $$r_1\le r\le R_1$$,
  30 $$\begin{aligned} \textbf{N}=\textbf{T}. \end{aligned}$$
4. For $\mathcal {H}= \mathcal {H}^+, \overline{\mathcal {H}}^+$, there exists some $c>0$ such that
  31 $$\begin{aligned} {\nabla _{g_b}\cdot \textbf{N}}\big \vert _{\mathcal {H}} < -c. \end{aligned}$$

#### Proof

See Appendix B.3. $$\square $$

#### Remark 5

Observe that the $\textbf{N}$ constructed in Proposition 2.7 depends on the vectorfield *X* and the constant $\varepsilon _{\textbf{N}}$ chosen. In practice, we take $\left\lvert X\right\rvert$ sufficiently large and $\varepsilon _{\textbf{N}}$ sufficiently small so $\textbf{N}$ is fixed throughout the remainder of the paper.

The following technical lemma (see Lemma 3.11 in [62] for the anti-de-Sitter equivalent) constructs the $\mathcal {K}_i$ vectorfields which will be used to define suitable Sobolev spaces in Sect. 2.8.

#### Lemma 2.8

There exists a finite collection of vectorfields $\left\{ \mathcal {K}_i\right\} _{i=1}^N$ with the following properties:

1. $\mathcal {K}_i$ are stationary, smooth vectorfields on $\mathcal {M}$.
2. Near $\mathcal {H}^+$, $\mathcal {K}_1$ is future-oriented null with $g(\mathcal {K}_1,\textbf{K}_{\mathcal {H}^+}) = -1$, and near $\overline{\mathcal {H}}^+$, $\mathcal {K}_1$ is future-oriented null with $g(\mathcal {K}_1,\textbf{K}_{\overline{\mathcal {H}}^+}) = -1$.
3. $\mathcal {K}_i$ are tangent to both $\mathcal {H}^+$ and $\overline{\mathcal {H}}^+$ for $2\le i\le N$.
4. If *X* is any vectorfield supported in $\mathcal {M}$, then there exist smooth functions $x^i$, not necessarily unique, such that
  $$\begin{aligned} X = \sum _i x^i\mathcal {K}_i. \end{aligned}$$
5. We have the following decomposition of the deformation tensor of $\mathcal {K}_i$, 32 for stationary functions $f^{jk}_i = f^{kj}_i\in C^\infty _c(\mathcal {M})$, and on $\mathcal {H}\in \left\{ \mathcal {H}^+, \overline{\mathcal {H}}^+\right\}$,
  $$\begin{aligned} f^{11}_1&= \kappa _{\mathcal {H}},\\ f^{11}_i&=0,\quad i\ne 1. \end{aligned}$$

#### Proof

See Appendix B.4. $$\square $$

### The Almost Killing Timelike Vectorfield $\widetilde{\textbf{T}}$

There are no globally timelike Killing vectorfields on $a\ne 0$ Kerr-de Sitter backgrounds. However, we can define a vectorfield which is Killing outside of two disconnected components that avoid the horizons as well as a neighborhood of $r=3M$, which has an *O*(*a*) deformation tensor and is timelike up to the horizons, where it becomes null.

#### Lemma 2.9

There exists a function $\tilde{\chi }(r) \in C^\infty (\mathcal {M})$ such that

33 $$\begin{aligned} \widetilde{\textbf{T}}= \textbf{T}+ a\tilde{\chi }(r){\Phi }, \end{aligned}$$

satisfies the properties

1. $${\text {supp}}\,\tilde{\chi }(r) \subset [r_{\mathcal {H}^+} - \varepsilon _{\mathcal {M}}, r_0] \bigcup [R_0, r_{\overline{\mathcal {H}}^+}+ \varepsilon _{\mathcal {M}}]$$;
2. is timelike on $\mathcal {M}^\circ \backslash \{\mathcal {H}^+\bigcup \overline{\mathcal {H}}^+\}$, and exactly null on both $\mathcal {H}^+$ and $\overline{\mathcal {H}}^+$; and
3. the deformation tensor of $\widetilde{\textbf{T}}$ is given by 34 where we denote by $X^\flat$ the canonical one-form for any vectorfield *X*.

#### Proof

See Appendix B.5. $$\square $$

### Sobolev Spaces

In this section, we some useful Sobolev spaces that will feature in what follows.

#### Definition 11

Let $h:\mathcal {M}\rightarrow \mathbb {C}^D$. Then denoting by $\mathcal {D}$ a subset of $\mathcal {M}$, we define the regularity spaces

$$\begin{aligned} L^2(\mathcal {D}) {:=} \left\{ h: \int _{\mathcal {D}}\left\lvert h\right\rvert ^2 <\infty \right\} , \qquad H^k(\mathcal {D}) {:=} \left\{ h: \mathcal {K}^\alpha h\in L^2(\mathcal {D}), \left\lvert \alpha \right\rvert \le k\right\} . \end{aligned}$$

Let us also define two $L^2$ inner products on spacelike slices.

#### Definition 12

We define the $\underline{L}^2$ and $L^2$ inner products on the spacelike slice $\Sigma _{t_*}$ by

$$\begin{aligned} \begin{aligned} \left\langle {h}_1, {h}_2\right\rangle _{\underline{L}^2(\Sigma _{t_*})}&= \int _{\Sigma _{t_*}} {h}_1 \cdot \overline{h}_2\, \sqrt{A_b},\\ \left\langle {h}_1, {h}_2\right\rangle _{L^2(\Sigma _{t_*})}&= \int _{\Sigma _{t_*}}{h}_1\cdot \overline{h}_2, \end{aligned} \end{aligned}$$

where $A_b$ is as defined in (14).

#### Remark 6

Observe that due to (15), the two norms are equivalent to each other. Furthermore, despite the dependence on the choice of $g_b$ reflected in the presence of $A_b$ in the definition of $\underline{L}^2(\Sigma )$, the $\underline{L}^2(\Sigma )$ norm defined for differing slowly-rotating Kerr-de Sitter metrics $g_b$ are all equivalent to each other.

We likewise have the following higher-regularity Sobolev norms.

#### Definition 13

For any $u:\Sigma \rightarrow \mathbb {C}^D$, let $\upsilon :\mathcal {M}\rightarrow \mathbb {C}^D$ be the unique lifting satisfying

35 $$\begin{aligned} \upsilon \vert _{\Sigma } = u,\quad \textbf{T}\upsilon = 0. \end{aligned}$$

Then define the regularity spaces $H^k(\Sigma ), \underline{H}^{k}(\Sigma )$ by:

$$\begin{aligned} \begin{aligned} H^k(\Sigma )&{:=} \left\{ u:\left. \mathcal {K}^\alpha \upsilon \right| _{\Sigma } \in L^2(\Sigma ), \left\lvert \alpha \right\rvert \le k\right\} , \\ \underline{H}^{k}(\Sigma )&{:=}\left\{ u:\left. \mathcal {K}^\alpha \upsilon \right| _{\Sigma } \in \underline{L}^2(\Sigma ), \left\lvert \alpha \right\rvert \le k\right\} , \end{aligned} \end{aligned}$$

where $\alpha$ is a multi-index and $\mathcal {K}_i$ are vectorfields satisfying the requirements of Lemma 2.8.

For $h:\mathcal {M}\rightarrow \mathbb {C}^D$, with the same $\mathcal {K}_i$, we abuse notation to define the norm $\overline{H}^{k}(\Sigma _{t_*})$ by:

$$\begin{aligned} \left\Vert h\right\Vert _{\overline{H}^{k}(\Sigma _{t_*})}^2 {:=} \sum _{\left\lvert \alpha \right\rvert \le k} \left\Vert \mathcal {K}^\alpha h\right\Vert _{\underline{L}^2(\Sigma _{t_*})}^2. \end{aligned}$$

#### Remark 7

Observe that different choices of the family $\left\{ \mathcal {K}_i\right\} _{i=1}^N$ will result in different, though equivalent, $H^k(\Sigma ), \underline{H}^{k}(\Sigma ), \overline{H}^{k}(\Sigma )$ norms.

#### Remark 8

At first glance, it may appear that since the construction of the $\{\mathcal {K}_i[g_b]\}$ family of vectorfields in Lemma 2.8 depends on the chosen Kerr-de Sitter metric, the $H^k[g_b](\mathcal {D})$ norms defined in Definition 13 are also dependent on the chosen Kerr-de Sitter metric. However, recall that by its construction in Proposition 2.7, $\textbf{N}$ is uniformly timelike on $\mathcal {M}$ and transverse to both the event horizon and cosmological horizon for all slowly-rotating Kerr-de Sitter black hole backgrounds. Combined with Lemma 2.8, we have that in fact for any slowly-rotating Kerr-de Sitter metric $g_b$, the $H^k[g_b](\mathcal {D})$ norm is equivalent to

$$\begin{aligned} H^k_{\star }(\mathcal {D}){:=}\left\{ u: Z^\alpha u\in L^2(\mathcal {D}), \left\lvert \alpha \right\rvert \le k, Z\in \{\textbf{N}, \left\{ \mathcal {K}_i[b_0]\right\} _{i=2}^{N}\}\right\} . \end{aligned}$$

A similar equivalence holds for the $H^k(\Sigma )$, $\underline{H}^{k}(\Sigma )$, and $\overline{H}^{k}(\Sigma )$ norms.

We also define the following Laplace-transformed Sobolev norms:

#### Definition 14

Let $u:\Sigma \rightarrow \mathbb {C}^D$. Then, we define the *Laplace-transformed Sobolev norms* by:

$$\begin{aligned} \left\Vert u\right\Vert _{\underline{H}^{1}_\sigma (\Sigma )}^2&= \left\Vert u\right\Vert _{\underline{H}^{1}(\Sigma )}^2 + \left\Vert \sigma u\right\Vert _{\underline{L}^2(\Sigma )}^2,\qquad \left\Vert u\right\Vert _{\underline{H}^{k}_\sigma (\Sigma )}^2 = \left\Vert u\right\Vert _{\underline{H}^{k}(\Sigma )}^2 + \left\Vert \sigma u\right\Vert _{\underline{H}^{k-1}_{\sigma }(\Sigma )}^2, \end{aligned}$$

so that $$\underline{H}^{k}_\sigma (\Sigma ) = \underline{H}^{k}(\Sigma ) \bigcap \sigma ^{-1}\underline{H}^{k-1}_\sigma (\Sigma )$$.

#### Remark 9

We define the Laplace-transformed vectorfields $$\widehat{\mathcal {K}}(\sigma )u= e^{\mathbbm {i}\sigma t_*}\mathcal {K}(e^{-\mathbbm {i}\sigma t_*}\upsilon ) $$$$ \vert _{\Sigma _{t_*}}$$, where $$\upsilon $$ is the stationary extension of *u* defined in (35). We can also characterize

$$\begin{aligned} \left\Vert u\right\Vert _{\underline{H}^{k}_\sigma (\Sigma )}^2 = \sum _{\left\lvert \alpha \right\rvert \le k}\left\Vert \widehat{\mathcal {K}}^\alpha (\sigma )u\right\Vert _{\underline{L}^2(\Sigma )}^2. \end{aligned}$$

We use the vectorfields defined in Theorem 2.8 to define the following higher-regularity Sobolev spaces.

#### Definition 15

We define $$\textbf{H}^{k}(\Sigma )$$ to be the space consisting of $$(\psi _0,\psi _1)\in H^k_{{loc}}(\Sigma , \mathbb {C}^D)$$$$\times H^{k-1}_{loc}(\Sigma , \mathbb {C}^D)$$ and denote the norm

$$\begin{aligned} \left\Vert (\psi _0, \psi _1)\right\Vert ^2_{\textbf{H}^{k}(\Sigma )} {:=} \left\Vert \psi _0\right\Vert ^2_{H^{k}(\Sigma )} + \left\Vert \psi _1\right\Vert ^2_{H^{k-1}(\Sigma )}. \end{aligned}$$

Finally, we also define the following weighted Sobolev spaces.

#### Definition 16

For $\alpha \in \mathbb {R}$, $h: \mathcal {M}\rightarrow \mathbb {C}^D$, define the *weighted Sobolev norm*

$$\begin{aligned} \left\Vert h\right\Vert _{H^{k,\alpha }(\mathcal {M})}^2{:=} \int _0^\infty e^{2\alpha t_*} \left\Vert h\right\Vert _{\overline{H}^{k}(\Sigma _{t_*})}^2\,dt_*. \end{aligned}$$

### Strongly Hyperbolic Operators

#### Definition 17

Given a linear second-order differential operator $L$, acting on complex matrix functions

$$\begin{aligned} h: \mathcal {M}\rightarrow \mathbb {C}^D, \end{aligned}$$

we call $L$ a *strongly hyperbolic operator* on a background Lorentzian metric *g* if $L$ can be expressed as

36 $$\begin{aligned} L{h} = \Box _{g}{h} + \textbf{S}[{h}] + \textbf{V}{h}, \end{aligned}$$

where $\Box _{g}$ denotes the scalar geometric wave operator $\nabla ^\alpha \partial _\alpha$,

37 $$\begin{aligned} \textbf{S}= S^\alpha \partial _\alpha \end{aligned}$$

is a smooth vectorfield-valued matrix, and $\textbf{V}$ is a smooth matrix potential. We will often refer to $\textbf{S}$ as the *subprincipal operator* of $L$, and $\textbf{V}$ as the *potential operator* of $L$.

As we will show in Lemma 3.4, the gauge-fixed linearized Einstein operator is an example of a strongly hyperbolic operator.

Given some strongly hyperbolic $L$ on background metric *g*, we define the following quantities,

38 $$\begin{aligned} \begin{aligned} \textbf{s}_{L}[\mathcal {H}^+]&= \sup _{\mathcal {H}^+}\left\{ -g\left( \textbf{K}_{\mathcal {H}^+}, \Re [\overline{\xi }\cdot \textbf{S}\xi ]\right) : \xi \in \mathbb {C}^N,\left\lvert \xi \right\rvert =1\right\} ,\\ \textbf{s}_{L}[\overline{\mathcal {H}}^+]&= \sup _{\overline{\mathcal {H}}^+}\left\{ -g\left( \textbf{K}_{\overline{\mathcal {H}}^+}, \Re [\overline{\xi }\cdot \textbf{S}\xi ]\right) : \xi \in \mathbb {C}^N,\left\lvert \xi \right\rvert =1\right\} ,\\ \textbf{s}_{L}^*[\mathcal {H}^+]&= \inf _{\mathcal {H}^+}\left\{ -g\left( \textbf{K}_{\mathcal {H}^+}, \Re [\overline{\xi }\cdot \textbf{S}\xi ]\right) : \xi \in \mathbb {C}^N,\left\lvert \xi \right\rvert =1\right\} ,\\ \textbf{s}_{L}^*[\overline{\mathcal {H}}^+]&= \inf _{\overline{\mathcal {H}}^+}\left\{ -g\left( \textbf{K}_{\overline{\mathcal {H}}^+}, \Re [\overline{\xi }\cdot \textbf{S}\xi ]\right) : \xi \in \mathbb {C}^N,\left\lvert \xi \right\rvert =1\right\} , \end{aligned} \end{aligned}$$

where we use the shorthand notation

$$\begin{aligned} g\left( \textbf{K}, \Re [\overline{\xi }\cdot \textbf{S}\xi ]\right) {:=} g_{\alpha \beta }\textbf{K}^\alpha \Re [\overline{\xi }\cdot S^\beta \xi ]. \end{aligned}$$

The quantities in (38) will play a critical role in our formulation of quasinormal modes (see Sect. 4).

#### Definition 18

Given a strongly hyperbolic operator $L$ of the form in equation (36), we can define its adjoint^9^$L^*$ as

39 $$\begin{aligned} L^* {h} = \Box _{g}{h} - \textbf{S}^*[{h}] + \textbf{V}^*{h} - (\nabla _{g}\cdot \textbf{S}^*){h}, \end{aligned}$$

where $\textbf{S}^*$ is a vectorfield-valued matrix such that

$$\begin{aligned} \textbf{S}^{*} = (S^\mu )^*\partial _\mu , \end{aligned}$$

with $S^\mu$ as defined in (37), and where we understand the adjoint of a matrix to be its transpose.

It is immediately clear that $L^*$ is itself a strongly hyperbolic operator on $\mathcal {M}$. Moreover, we have the following adjoint relation.

#### Corollary 2.10

Then for $${h}_1, {h}_2\in C^2(\mathcal {M},\mathbb {C}^D)$$:

$$\begin{aligned} \begin{aligned}&\left\langle {h}_1,L{h}_2\right\rangle _{\underline{L}^2(\Sigma _{t_*})} - \left\langle L^* {h}_1, {h}_2\right\rangle _{\underline{L}^2(\Sigma _{t_*})}\\ ={}&\partial _{t_*}\int _{\Sigma _{t_*}} K\cdot n_\Sigma + \int _{\mathcal {H}^+\bigcap \Sigma _{t_*}}K\cdot n_{\mathcal {H}^+} + \int _{\overline{\mathcal {H}}^+\bigcap \Sigma _{t_*}}K\cdot n_{\overline{\mathcal {H}}^+} , \end{aligned} \end{aligned}$$

where

$$\begin{aligned} K_\mu = \overline{{h}}_1\cdot \nabla _\mu {h}_2 - \nabla _\mu \overline{h}_1\cdot {h}_2 - \overline{{h}}_1 \cdot S_\mu {h}_2. \end{aligned}$$

#### Proof

The proof follows directly from applying Corollary 2.6 with the vectorfield $K_\mu$. $\square$

## Einstein’s Equations

In this section, we introduce the generalized harmonic coordinates and the hyperbolic initial value problem formulation of Einstein’s equations as a system of evolution equations. Then we detail some key properties of the linearized Einstein operator that will be crucial in the ensuing stability analysis.

### Harmonic Gauge

Recall that Einstein’s vacuum equations with a cosmological constant $\Lambda$ for *g*, a $(-, +, +, +)$ Lorentzian metric on a smooth manifold $\mathcal {M}$, are

40 $$\begin{aligned} {\text {Ric}}(g) - \Lambda g= 0. \end{aligned}$$

For any globally hyperbolic $(\mathcal {M}, g)$ solution to (40), and spacelike hypersurface $\Sigma _0\subset \mathcal {M}$, the induced Riemannian metric $\underline{g}$ on $\Sigma _0$ and the second fundamental form *k*(*X*, *Y*) of $\Sigma _0$ satisfy the *constraint equations*

41 $$\begin{aligned} \begin{aligned} R(\underline{g}) + ({\text {tr}}_{\underline{g}}k)^2 - \left\lvert k\right\rvert _{\underline{g}}^2&= - 2\Lambda ,\\ \nabla _{\underline{g}} \cdot k - d {\text {tr}}_{\underline{g}} k&= 0, \end{aligned} \end{aligned}$$

where $R(\underline{g})$ is the scalar curvature of $\underline{g}$, and $\nabla _{\underline{g}}\cdot k$ denotes the divergence of *k* with respect to the covariant derivative of $\underline{g}$. The Cauchy problem for Einstein’s equations then asks, given an initial data set consisting of the triple $(\Sigma _0, \underline{g}, k)$, where $\underline{g}$ is a Riemannian metric on the smooth 3-manifold $\Sigma _0$ and *k* is a symmetric 2-tensor on $\Sigma _0$ such that $(\underline{g}, k)$ satisfy (41), for a Lorentzian 4-manifold $(\mathcal {M}, g)$ and an embedding $\Sigma _0\hookrightarrow \mathcal {M}$ such that $\underline{g}$ is the induced metric on $\Sigma _0$, and *k* is the second fundamental form of $\Sigma _0$ in $\mathcal {M}$. We denote initial data triplets with $(\underline{g}, k)$ satisfying the constraint equations (41) to be *admissible* initial data triplets.

It is well-known that (40) is a quasilinear second-order partial differential system of equations for the metric coefficients $g_{\mu \nu }$. In local coordinates, we can write (40) as

$$\begin{aligned} {\text {Ric}}(g)_{\mu \nu } = - \frac{1}{2}g^{\alpha \beta }\partial _{\alpha }\partial _{\beta }g_{\mu \nu } + \nabla _{(\mu }\Gamma (g)_{\nu )} + \mathcal {N}(g, \partial g),\qquad \Gamma (g)^\mu {:=} g^{\alpha \beta } \Gamma (g)^{\mu }_{\alpha \beta }, \end{aligned}$$

where the nonlinear term $\mathcal {N}(g, \partial g)$ involves at most one derivative of *g*. As a result of the presence of the $\nabla _{(\mu }\Gamma (g)_{\nu )}$ term EVE lacks any useful structure. However, as was first demonstrated by Choquet-Bruhat, this problem can be overcome by using the general covariance of Einstein’s equations and choosing *wave coordinates*. With this choice Einstein’s equations become a quasilinear hyperbolic system of equations [7, 8].

#### Definition 19

Define the *constraint operator*

$$\begin{aligned} \mathcal {C}(g, g^0)_\mu = g_{\mu \chi }g^{\nu \lambda }\left( \Gamma (g)^{\chi }_{\nu \lambda } - \Gamma (g^0)^{\chi }_{\nu \lambda } \right) , \end{aligned}$$

where $g^0$ is a fixed background metric which solves Einstein’s equations^10^. Then we say that a Lorentzian metric *g* satisfies the *harmonic coordinate condition* (with respect to $g^0$) if

42 $$\begin{aligned} g_{\mu \chi }g^{\nu \lambda }\left( \Gamma (g)^{\chi }_{\nu \lambda } - \Gamma (g^0)^{\chi }_{\nu \lambda } \right) = 0. \end{aligned}$$

#### Remark 10

Instead of 0 on the right-hand side, if we instead pick some one-form $\mathbb {H}(g)$ depending on *g* but not its derivatives, we would obtain *generalized harmonic coordinates*.

Crucial to the utility of harmonic coordinates is that they are propagated by a hyperbolic operator.

#### Definition 20

Given a smooth one-form $\psi$, we define the *constraint propagation operator*,43where44From (43), $\Box ^{CP}$ is a manifestly hyperbolic operator on $\psi$.

#### Lemma 3.1

Any solution *g* to

45 $$\begin{aligned} {\text {Ric}}(g) - \Lambda g + \nabla _g\otimes \mathcal {C}(g,g^0) =0 \end{aligned}$$

must also satisfy

$$\begin{aligned} \Box ^{CP}_g\mathcal {C}(g,g^0)= 0. \end{aligned}$$

#### Proof

The conclusion follows directly by applying the twice-contracted second Bianchi identity to (45). $\square$

#### Proposition 3.2

If $g\in S^2T^*\mathcal {M}$ satisfies the gauge-fixed Einstein equation

46 $$\begin{aligned} \begin{aligned} {\text {Ric}}(g) - \Lambda g + \nabla _g\otimes \mathcal {C}(g,g^0) ={}&0 \end{aligned} \end{aligned}$$

and moreover, *g* satisfies the gauge constraint on $\Sigma _0$,

$$\begin{aligned} \mathcal {C}(g, g^0)\vert _{\Sigma _0} = 0, \end{aligned}$$

and the initial data $(\underline{g},k)$on $\Sigma _0$ induced by *g* satisfies the constraint equations in (41), then *g* is a solution to the non-gauge-fixed Einstein vacuum equations (40).

#### Proof

The constraint equations in (41) and the assumption that $\mathcal {C}(g, g^0)\vert _{\Sigma _0} = 0$ together imply that in particular, we must also have that

$$\begin{aligned} \mathcal {L}_{n_{\Sigma _0}}\mathcal {C}(g, g^0)\vert _{\Sigma _0} = 0. \end{aligned}$$

Then the result follows from Lemma 3.1 and uniqueness of solutions for hyperbolic PDEs. For a more detailed and complete reference of this argument, we refer the reader to Section 2 of [51]. $\square$

### The Linearized Einstein Equations

We now introduce the linearized Einstein equation, for a more in-depth introduction, we refer the reader to Section 3 of [24]. Directly linearizing (40) around $g_b$ yields the non-gauge-fixed linearized Einstein equation

47 $$\begin{aligned} D_{g_b}({\text {Ric}}- \Lambda )({h}) = 0. \end{aligned}$$

Given admissible initial data $(\Sigma _0, \underline{g}_0, k_0)$ for $g_b$, we define the *linearized constraint equation* as the linearization of (41) around $(\underline{g}_0, k_0)$ in terms of the linearized metric $\underline{g}'$ and the linearized second fundamental form $k'$. An initial data triplet $(\Sigma _0, \underline{g}', k')$ linearized around $(\underline{g}_b, k_b)$ is an *admissible* initial data triplet for Einstein equations linearized around $g_b$ if $(\underline{g}', k')$ satisfy the linearized constraint equations. Linearizing the gauge-fixed Einstein equations in (45), we have the linearized gauge-fixed Einstein equations

48 $$\begin{aligned} D_{g_b}({\text {Ric}}- \Lambda )({h}) - \nabla _{g_b}\otimes D_{g_b}\mathcal {C}(g, g_b)(h) = 0. \end{aligned}$$

#### Definition 21

Define the *linearized gauge constraint*

We have the following linearized equivalent of Lemma 3.1.

#### Lemma 3.3

Let *h* solve (48). Then *h* also satisfies

$$\begin{aligned} \Box ^{CP}_{g_b}(\mathcal {C}_{g_b}{h}) = 0, \qquad \Box ^{CP}_{g_b}\psi = \Box ^{(1)}_{g_{b}}\psi - \Lambda \psi , \end{aligned}$$

where $\Box ^{(1)}_{g_{b}} = \nabla ^\alpha \nabla _\alpha$ denotes the wave operator acting on 1-tensors.

#### Proof

The lemma follows directly by applying the twice-contracted linearized second Bianchi identity to the gauge-fixed linearized Einstein equation. $\square$

#### Remark 11

From Lemma 3.3, it is clear that if $({\mathcal {C}_{g_b}{h}}\big \vert _{\Sigma _0}, {\mathcal {L}_{\textbf{T}}\mathcal {C}_{g_b}{h}}\big \vert _{\Sigma _0}) = (0, 0)$, then $\mathcal {C}_{g_b}{h} = 0$ for all $t_*\ge 0$.

Finally, we remark that any solution *h* to the non-gauge-fixed linearized Einstein’s equations (47) can be put into the linearized gauge $C_{g_b}({h}) = 0$ by finding some infinitesimal diffeomorphism $\nabla _{g_b}\otimes \omega$^11^ such that

49 $$\begin{aligned} \mathcal {C}_{g_b}({h} + \nabla _{g_b}\otimes \omega ) = 0, \end{aligned}$$

as general covariance implies that

$$\begin{aligned} D_{g_b}({\text {Ric}}- \Lambda )(\nabla _{g_b}\otimes \omega ) = 0 \end{aligned}$$

for any one-form $\omega \in C^\infty (\mathcal {M}, T^*\mathcal {M})$. This is equivalent to finding some $\omega$ such that

50 $$\begin{aligned} \Box _{g_b}^\Upsilon \omega = 2\mathcal {C}_{g_b}({h}),\qquad \Box _{g_b}^{\Upsilon } = -2\mathcal {C}_{g_b}\circ \nabla _{g_b}\otimes , \end{aligned}$$

which is principally $\Box ^{(1)}_{g_{b}}$, and in fact, in our case we can calculate that

$$\begin{aligned} \Box _{g_b}^\Upsilon = \Box ^{(1)}_{g_b} - \Lambda . \end{aligned}$$

Solving for $\omega$ satisfying (50) with Cauchy data $\left. (\omega , \mathcal {L}_{\textbf{T}}\omega )\right| _{\Sigma _0}= 0$ then ensures that ${h} +\nabla _{g_b}\otimes \omega$ has the same initial data as *h*.

### Properties of $\mathbb {L}_{g_b}$

Using wave coordinates, we can compute the exact quasilinear structure of Einstein’s equations.

#### Lemma 3.4

Let $g_b + h$ be a solution to the gauge-fixed Einstein vacuum equations in (46) where we choose $g^0=g_b$. Then, *h* solves

$$\begin{aligned} \mathbb {L}_{g_b}h = \mathcal {N}(h,\partial h, \partial \partial h), \end{aligned}$$

where

51 $$\begin{aligned} \mathbb {L}_{g_b}h&{:=} \frac{1}{2}\Box ^{(2)}_{g_{b}}h + \mathcal {R}_g(h), \end{aligned}$$

52 $$\begin{aligned} \mathcal {R}_{g_b}({h})_{\mu \nu }&{:=} {h}^{\alpha \lambda }(R_{g_b})_{\alpha \mu \nu \lambda } - \Lambda {h}_{\mu \nu }, \end{aligned}$$

where $\Box ^{(2)}_{g}{:=}\nabla ^\alpha \nabla _\alpha$ denotes the wave operator acting on 2-tensors, $R_{g_b}$ is the Riemann curvature tensor of $g_b$, and $\mathcal {N}$ is a quasilinear nonlinear term in *h*. In particular, $\mathbb {L}_{g_b}$ is a strongly hyperbolic operator, as defined in Definition 17.

#### Proof

The conclusion follows from equation (2.4) in [24] and the harmonic gauge condition in (42). It should be noted that when writing $\mathbb {L}_{g_b}h$ as a strongly hyperbolic operator,

$$\begin{aligned} \mathbb {L}_{g_b} = \Box _{g_{b}} + \textbf{S}_{b} + \textbf{V}_b, \end{aligned}$$

the exact coefficients in $\textbf{S}_b$ and $\textbf{V}_b$ will depend not only on the coordinate system chosen, but also on the particular frame used to split $S^2T^*\mathcal {M}$. To see that $\mathbb {L}_{g_b}$ can be written as a global system of strongly hyperbolic equations, it suffices to take some global frame on $\mathcal {M}$ (for example, the Cartesian frame). $\square$

#### Definition 22

We refer to $\mathbb {L}= \mathbb {L}_g$ defined in (51) as the *gauge-fixed linearized Einstein operator*.

We can also compute the value of $\textbf{s}_{\mathbb {L}_{g_{b_0}}}[\mathcal {H}]$.

#### Lemma 3.5

Let $g_{b_0}$ be a fixed member of the Schwarzschild-de Sitter family. Then,

$$\begin{aligned} \textbf{s}_{\mathbb {L}_{g_{b_0}}}[\mathcal {H}] = 4\kappa _{b_0,\mathcal {H}},\qquad \mathcal {H}=\mathcal {H}^+,\overline{\mathcal {H}}^+, \end{aligned}$$

where we recall the definition of $\textbf{s}_{L}[\mathcal {H}]$ from (38).

#### Proof

See Appendix C.2. $$\square $$

### Initial Data

In this section, we will construct the mapping $i_{b, \phi }$ between admissible initial data triplets $(\Sigma _0, \underline{g}_0, k_0)$ for the Cauchy problem for the non-gauge-fixed Einstein equations (40) and the admissible initial data $(h_0, h_1)$ for the Cauchy problem for the gauge-fixed Einstein equations. Recall from Sect. 3.1 that an important property of this mapping is that a metric perturbation *h* such that $\gamma _0(h)=(h_0, h_1)$ satisfies the gauge constraint $\mathcal {C}(g_b+h, g^0)\vert _{\Sigma _0} = 0$. To construct $i_{b, \phi }$ we need to specify a choice of $g^0$. In the remainder of this paper, it will be convenient to choose $g^0 = g_b$.

Consider the Kerr-de Sitter initial data triplet $(\Sigma _0, \underline{g}_b, k_b)$ that launches $g_b$, so that in particular, $\underline{g}_b$ and $k_b$ denote the induced metric and second fundamental form on $\Sigma _0$ by $g_b$. As a solution to Einstein’s equations, the particular form of Kerr-de Sitter that we have explored in Sect. 2.2 is not important. Indeed, due to general covariance, if $\phi :\mathcal {M}\rightarrow \mathcal {M}$ is a smooth family of diffeomorphisms that commute with translation in $t_*$, then $\phi ^*g_b$ is another family of smooth Kerr-de Sitter solutions to Einstein’s equations which are not “distinct” from $g_b$. That is, we are only interested in solving Einstein’s equations up to a diffeomorphism. To this end, we will construct $i_{b, \phi }$ mapping $(\Sigma _0, \underline{g}_b, k_b)$ into Cauchy data for (46) launching the Kerr-de Sitter solution $\phi ^*g_b$. That is, we will have that

$$\begin{aligned} i_{b, \phi }(\phi ^*\underline{g}_b, \phi ^*k_b) = (0,0). \end{aligned}$$

The linearization of this mapping will also produce the correctly gauge-fixed initial data for the gauge-fixed linearized Einstein equation linearized around $g_b$.

#### Proposition 3.6

Fix some one-form $\vartheta$ generating the diffeomorphism $\phi :\mathcal {M}\rightarrow \mathcal {M}$. Then there exist neighborhoods of symmetric two-tensors on $\Sigma _0$

$$\begin{aligned} H\subset C^1(\Sigma _0; S^2T^*\Sigma _0),\qquad K\subset C^0(\Sigma _0; S^2T^*\Sigma _0) \end{aligned}$$

of $\underline{g}_{b_0}$ and $k_{b_0}$, respectively, so that $\underline{g}_b\in H$, $k_b\in K$ for all $b\in \mathcal {B}$, where $\mathcal {B}$ is a sufficiently small neighborhood of black-hole parameters of $b_0$; and moreover, for each $b\in \mathcal {B}$, there exists a map

$$\begin{aligned} \begin{aligned} i_{b, \phi }:&\left( H\bigcap C^m(\Sigma _0; S^2T^*\Sigma _0)\right) \times \left( K\bigcap C^{m-1}(\Sigma _0;S^2T^*\Sigma _0)\right) \\&\rightarrow C^m(\Sigma _0; S^2T^*_{\Sigma _0}\mathcal {M})\times C^{m-1}(\Sigma _0;S^2T^*_{\Sigma _0}\mathcal {M}), \end{aligned} \end{aligned}$$

that is smooth for $m\ge 1$ depending smoothly on *b*, such that

1. if *h* is some symmetric two-tensor such that $\gamma _0(h) = i_{b, \phi }(\underline{g}_0, k_0)$, and $g = \phi ^*(g_b+h)$, then
  $$\begin{aligned} (\underline{g}, k) = (\underline{g}_0, k_0), \end{aligned}$$
  where $(\underline{g}, k)$ are the induced metric and the second fundamental form respectively of $\phi (\Sigma _0)$ induced by *g*. Moreover, $g_b+h$ satisfies the gauge constraint
  $$\begin{aligned} \mathcal {C}(g_b+h,g_b)\vert _{\Sigma _0} = 0; \end{aligned}$$
2. if $(\underline{g}_b, k_b)$ is the admissible initial data launching the Kerr-de Sitter metric $g_b$, then
  $$\begin{aligned} i_{b, \phi }(\phi ^*\underline{g}_b, \phi ^*k_b) = (0, 0); \end{aligned}$$
3. $(g_0, g_1) = i_{b, \phi }(\underline{g}, k)$ satisfies the condition
  $$\begin{aligned} \left\Vert (g_0, g_1)\right\Vert _{\textbf{H}^{k}(\Sigma _0)} \lesssim {}&\sum _{0\le \left\lvert I\right\rvert \le k}\left\Vert \partial _{x}^I(\underline{g}- \underline{g}_b)\right\Vert _{L^2(\Sigma _0)} + \sum _{0\le \left\lvert I\right\rvert \le k+1}\left\Vert \partial _x^I\vartheta \right\Vert _{L^2(\Sigma _0)}\\&+ \sum _{0\le \left\lvert I\right\rvert \le k-1}\left\Vert \partial _x^I (k - k_b)\right\Vert _{L^2(\Sigma _0)}, \end{aligned}$$
  where *I* is a multi-index.

#### Proof

See Appendix C.1. $$\square $$

The linearization of $i_{b, \phi }$ constructed above yields the correctly gauge-fixed Cauchy data for the linearized gauge-fixed Einstein equation, just as $i_{b, \phi }$ itself yields the correctly gauge-fixed Cauchy data for the nonlinear gauge-fixed Einstein equation.

#### Corollary 3.7

Fix $b\in \mathcal {B}$ and a one-form $\vartheta$ generating the diffeomorphism $\phi$. Suppose $(\Sigma _0, \underline{g}_b, k_b)$ is the smooth admissible initial data triplet launching $g_b$. Then, let $(\underline{g}', k')$ be smooth solutions of the linearized constraint equations linearized around $(\underline{g}_b, k_b)$, and let

$$\begin{aligned} D_{(\underline{g}_b, k_b)}i_{b, \phi }(\underline{g}', k') = ({h}_0, {h}_1). \end{aligned}$$

Finally, let ${h}\in S^2T^*\mathcal {M}$ be a metric perturbation inducing $({h}_0, {h}_1)$ on $\Sigma _0$ so that $\gamma _0({h}) = ({h}_0, {h}_1)$, where

53 $$\begin{aligned} \gamma _0(h){:=} ({h}\vert _{\Sigma _0}, {\mathcal {L}_{\textbf{n}} h}\Bigg \vert _{\Sigma _0}), \end{aligned}$$

where $\textbf{n}$ is the future-pointing unit normal to $\Sigma _0$.

Then *h* induces the linearized metric $\underline{g}'$ and second fundamental form $k'$ on $\Sigma _0$, and satisfies the linearized gauge constraint on $\Sigma _0$,

$$\begin{aligned} D_{g_b}\mathcal {C}(g_b+{h}, g_b)({h})\vert _{\Sigma _0} = 0. \end{aligned}$$

Moreover, if $({h}_0, {h}_1) = D_{(\underline{g}_b, k_b)}i_{b,\phi }(\underline{g}', k')$, then

$$\begin{aligned}&\left\Vert ({h}_0, {h}_1)\right\Vert _{\textbf{H}^{k}(\Sigma _0)} \lesssim {} \sum _{1\le \left\lvert I\right\rvert \le k}\left\Vert \partial _x^I \underline{g}'\right\Vert _{L^2(\Sigma _0)} + \sum _{1\le \left\lvert I\right\rvert \le k+1}\left\Vert \partial _x^I\vartheta \right\Vert _{L^2(\Sigma _0)}\\&\quad + \sum _{1\le \left\lvert J\right\rvert \le k-1}\left\Vert \partial _x^J k'\right\Vert _{L^2(\Sigma _0)}. \end{aligned}$$

#### Proof

The statement follows directly by linearizing the construction of $i_{b,\phi }$ in Appendix C.1. $\square$

## Quasinormal Spectrum

In this section, we define the quasinormal spectrum and establish the basic theory necessary to use the quasinormal spectrum to analyze the behavior of solutions to initial value problems. The definition of the $\textbf{H}^{k}$-quasinormal modes and their relation to the Laplace-transformed operator follows closely to the original work done in [62] in the anti-de Sitter case. However, we provide a more detailed analysis of the $\textbf{H}^{k}$-quasinormal spectrum and its relation to the initial value problem following an analysis analogous to that in Section 5 of [31]. We remark that Sects. 4.1, 4.2, and 4.3 make no assumptions on the particular choice of strongly hyperbolic operator. Sects. 4.4, 4.5, and 4.6 introduce certain assumptions (see Assumption 4.8) on the operators which in particular will be shown in Sect. 9 to be satisfied for the linearized gauge-fixed Einstein operator.

### Solution Operator Semigroup

We begin with a definition of the solution operator semigroup, which maps initial data to the evolution of a solution to $L\psi =0$ with the given initial data. In this section, we work on a fixed slowly-rotating Kerr-de Sitter background $g_b$ and drop the *b* subscript, denoting $g=g_b$, $A= A_b$ (we recall the definition of $A$ in (14)). We also work with a general strongly hyperbolic operator $L$, although our results will clearly also apply to $\mathbb {L}_g$.

#### Lemma 4.1

Any strongly hyperbolic operator on a fixed slowly-rotating Kerr-de Sitter black hole $g=g_b$

54 $$\begin{aligned} L= \Box _{g} + \textbf{S}+ \textbf{V},\qquad \textbf{S}= S^\mu \partial _\mu , \end{aligned}$$

where $S^\mu$ and $\textbf{V}$ are matrices of smooth functions, and $\Box _{g}$ denotes the Laplace-Beltrami operator of *g*, can be rewritten as

55 $$\begin{aligned} L= P_2+ \frac{1}{\mathbbm {i}} P_1D_{t_*} + \frac{1}{A} D_{t_*}^2, \end{aligned}$$

where $D_{t_*} = \mathbbm {i}\partial _{t_*}$, $A=A_b$ is defined in (14), and $P_i$ are $i^{{\text {th}}}$-order differential operators such that

56 $$\begin{aligned} \begin{aligned} P_1&= g^{it_*}\partial _i + S^{t_*} ,\\ P_2&= g^{ij}\partial _i\partial _j + S^i\partial _i + \textbf{V}. \end{aligned} \end{aligned}$$

Moreover, let $(h_0, h_1)\in \textbf{H}^{1}(\Sigma _0)$. Then we can rewrite the Cauchy problem

57 $$\begin{aligned} \begin{aligned} Lh&= 0,\\ \gamma _0(h)&= (h_0, h_1), \end{aligned} \end{aligned}$$

as a first-order system

58 $$\begin{aligned} \begin{aligned} \textbf{T}\begin{pmatrix} h\\ h' \end{pmatrix} = \begin{pmatrix} 0 &  1\\ AP_2&  AP_1 \end{pmatrix} \begin{pmatrix} h\\ h' \end{pmatrix},\qquad \left. \begin{pmatrix} h\\ h' \end{pmatrix}\right| _{t_*=0} = \begin{pmatrix} h_0\\ h_1 \end{pmatrix}. \end{aligned} \end{aligned}$$

#### Proof

It is a simple computation to verify that (55) and (58) holds for $P_2$ and $P_1$ as defined in (56). $\square$

#### Definition 23

Define the *solution operator* associated to $L$

$$\begin{aligned} \begin{aligned} \mathcal {S}(t_*): \textbf{H}^{1}(\Sigma )&\rightarrow \textbf{H}^{1}(\Sigma ),\\ (h_0, h_1)&\mapsto {(h, \partial _{t_*}h)}\big \vert _{\Sigma _{t_*}}, \end{aligned} \end{aligned}$$

be the solution operator of the Cauchy problem in (57) mapping the initial data to the solution at time $t_*$.

Recall from Lemma 3.4 that $\mathbb {L}_{g_b}$ is a strongly hyperbolic linear operator. In subsequent sections, we use subscripts to denote the specific infinitesimal generator of interest. As noted by Warnick in [62] on anti-de-Sitter spacetimes, $\mathcal {S}$ defines a $C^0$-semigroup (see [21]).

#### Proposition 4.2

Let $\mathcal {S}(t_*)$ be the solution operator for $L\psi = 0$ a strongly hyperbolic operator on a slowly-rotating Kerr-de Sitter background. Then the one-parameter family of operators $\mathcal {S}(t_*)$ defines a $C^0$-semigroup on $\textbf{H}^{k}(\Sigma )$.

#### Proof

See Appendix D.1. $$\square $$

Associated to the $C^0$-semigroup is the closed *infinitesimal generator* of the semigroup.

#### Definition 24

Define

$$\begin{aligned} D^k({\mathcal {A}}) {:=} \left\{ \psi \in \textbf{H}^{k}(\Sigma ):\mathbbm {i}\lim _{t_*\rightarrow 0+}\frac{\mathcal {S}(t_*)\psi -\psi }{t_*} \in \textbf{H}^{k}(\Sigma )\right\} \end{aligned}$$

the domain of

$$\begin{aligned} {\mathcal {A}}\psi {:=} \mathbbm {i}\lim _{t_*\rightarrow 0+}\frac{\mathcal {S}(t_*)\psi -\psi }{t_*}. \end{aligned}$$

The unbounded operator $(D^k(\mathcal {A}), \mathcal {A}),$ is the *infinitesimal generator* of the semigroup $\mathcal {S}(t_*)$ on $H^k(\Sigma )$. For $L$ as in (55), we have that

$$\begin{aligned} \mathcal {A}= \mathbbm {i}\begin{pmatrix} 0 &  1\\ AP_2&  AP_1 \end{pmatrix}. \end{aligned}$$

Observe that $(D^k(\mathcal {A}), \mathcal {A})$ for different values of *k* differ only in their domain. Moreover, $D^k(\mathcal {A})\subset D^{k-1}(\mathcal {A})$, and $(D^k(\mathcal {A}), \mathcal {A})$ and $(D^{k-1}(\mathcal {A}), \mathcal {A})$ agree on $D^k(\mathcal {A})\bigcap D^{k-1}(\mathcal {A})$. We will need the following classical properties of infinitesimal generators (See Corollary II.1.5 in [21]):

#### Proposition 4.3

The operator $(D^k(\mathcal {A}), \mathcal {A})$ satisfies the following properties.

1. The domain $D^k(\mathcal {A})$ is dense in $\textbf{H}^{k}(\Sigma )$.
2. $(D^k(\mathcal {A}), \mathcal {A})$ is a closed operator.
3. There exists some $\textbf{M}$ such that the resolvent $(\mathcal {A}-\sigma )^{-1}$ exists and is a bounded linear transformation of $\textbf{H}^{k}(\Sigma )$ onto $D^k(\mathcal {A})$ for $\Im (\sigma )>\textbf{M}$. In particular,
  $$\begin{aligned} \left\Vert \mathcal {S}(t_*)\textbf{h}\right\Vert _{\textbf{H}^{k}(\Sigma )} \lesssim e^{\textbf{M}t_*}\left\Vert \textbf{h}\right\Vert _{\textbf{H}^{k}(\Sigma )} \end{aligned}$$

#### Proof

These are well-known properties of infinitesimal generators. $\square$

We also have the following relationship between the resolvent $(\mathcal {A}-\sigma )^{-1}$ and the solution operator.

#### Lemma 4.4

Fix $({h}_0, {h}_1)\in \textbf{H}^{k}(\Sigma )$, and $L$ a strongly hyperbolic linear operator on a slowly-rotating Kerr-de Sitter background, and consider a solution *h* to the Cauchy problem

$$\begin{aligned} \begin{aligned} L{h}&= 0\\ \gamma _0({h})&= ({h}_0, {h}_1). \end{aligned} \end{aligned}$$

Moreover, let $(D^k(\mathcal {A}), \mathcal {A})$ be the infinitesimal generator for the $C^0$-solution semigroup $\mathcal {S}(t_*)$. Then for $\Im \sigma > \textbf{M}$ the constant in Proposition 4.3,

$$\begin{aligned} (\mathcal {A}-\sigma )^{-1}\textbf{h}_0 = \mathbbm {i}\int _{\mathbb {R}^+} e^{\mathbbm {i}\sigma {s_*}}\mathcal {S}({s_*})\textbf{h}_0\,d{s_*},\qquad \textbf{h}_0 = \begin{pmatrix} {h}_0\\ {h}_1 \end{pmatrix}. \end{aligned}$$

#### Proof

This is straightforward from applying Lemma A.2 and taking $t_*\rightarrow \infty$. $\square$

### $\textbf{H}^{k}$-Quasinormal Spectrum and Modes

Having established a $C^0$-semigroup, a closed, densely-defined infinitesimal generator $(D^k(\mathcal {A}), \mathcal {A})$, we can now analyze the asymptotic behavior of solutions to $(D_{t_*}-\mathcal {A})\psi = 0$ via the quasinormal spectrum.

#### Definition 25

Let $L$ be a strongly hyperbolic operator on a slowly-rotating Kerr-de Sitter background, and let $(D^k(\mathcal {A}), \mathcal {A})$ be the infinitesimal generator of the associated semigroup on $\textbf{H}^{k}(\Sigma )$. Then $\sigma \in \mathbb {C}$ belongs to the $\textbf{H}^{k}$-*quasinormal spectrum* of $L$, denoted by $\Lambda _{\operatorname {QNF}}^{k}(L)$, if

1. $$\Im \sigma > \frac{1}{2}\max _{\mathcal {H}=\mathcal {H}^+,\overline{\mathcal {H}}^+}\left( \textbf{s}_{L}[\mathcal {H}] - \left( 2k + \frac{1}{2}\right) \kappa _{\mathcal {H}} \right) $$,
2. $\sigma$ is in the spectrum of $(D^k(\mathcal {A}), \mathcal {A})$.

If $\sigma$ is an eigenvalue of $(D^k(\mathcal {A}), \mathcal {A})$, it is called an $\textbf{H}^{k}$-*quasinormal frequency* and its corresponding eigenfunctions, $\textbf{H}^{k}$-*quasinormal mode solutions*.

#### Remark 12

An advantage of this method of Definition 25 is that we do not have to construct a meromorphic extension of the resolvent. In this methodology, the faster the decay of a quasinormal mode, the higher regularity we require in order to study it.

#### Remark 13

The restriction of the $\textbf{H}^{k}$-quasinormal spectrum of $L$ to the half-space

59 $$\begin{aligned} \Im \sigma > \frac{1}{2}\max _{\mathcal {H}=\mathcal {H}^+,\overline{\mathcal {H}}^+}\left( \textbf{s}_{L}[\mathcal {H}] - \left( 2k + \frac{1}{2}\right) \kappa _{\mathcal {H}} \right) \end{aligned}$$

is not sharp. In fact, using the methods in this paper, for any fixed $\varepsilon _{\Omega }>0$, the $\textbf{H}^{k}$-quasinormal spectrum for a strongly hyperbolic operator on a sufficiently slowly-rotating Kerr-de Sitter background can be shown to be well-defined on the half-space

60 $$\begin{aligned} \Im \sigma >\frac{1}{2} \max _{\mathcal {H}=\mathcal {H}^+,\overline{\mathcal {H}}^+}\left( \textbf{s}_{L}[\mathcal {H}] - \left( 2k+1\right) \kappa _{\mathcal {H}} + \varepsilon _{\Omega }\right) . \end{aligned}$$

The loss of a $\varepsilon _{\Omega }>0$ when compared to the regularity levels in [62] is due to the fact that Kerr-de Sitter is not a globally stationary spacetime, and is instead only locally stationary (for a more in-depth discussion, see Sect. 5 of [62]).

While the restriction to the half-space in (59) is not optimal, it is nevertheless consistent with the application of the linear theory developed in the current paper to the context of proving nonlinear stability of Kerr-de Sitter in [22]. In particular, with the restriction in (59), the threshold regularity level $k_0$ in (79) for the gauge-fixed linearized Einstein operator $\mathbb {L}_{g_{b}}$ is $\frac{5}{2}+O(a)$.

### Laplace-Transformed Operator

In this section, we define the Laplace-transformed operator and see how it relates to the infinitesimal generator $(D^k(\mathcal {A}), \mathcal {A})$. We derive resolvent estimates for the Laplace-transformed operator using the vectorfield method in Sects. 6 and 8.

#### Definition 26

Given a linear operator *L*, we construct the *Laplace-transformed operator* of *L* by:

$$\begin{aligned} \widehat{L}(\sigma )u = \left. e^{\mathbbm {i}\sigma t_*}L(e^{-\mathbbm {i}\sigma t_*}u)\right| _{\Sigma _{t_*}}. \end{aligned}$$

Thus, the Laplace transform of a strongly hyperbolic linear operator $L= P_2 -\mathbbm {i}P_1 D_{t_*} + A^{-1} D_{t_*}^2$ is

$$\begin{aligned} \widehat{L}(\sigma ) = P_2 - \mathbbm {i}\sigma P_1 + \sigma ^2 A^{-1}. \end{aligned}$$

We then define a family of domains for $\widehat{L}(\sigma )$, $D^k(\widehat{L}(\sigma ))$ for $k\in \mathbb {N}$ to be the closure of $C^\infty _0(\Sigma , \mathbb {C}^D)$ with respect to the graph norm $\left\Vert u\right\Vert _{\underline{H}^{k-1}(\Sigma )}^2 + \left\Vert \widehat{L}(\sigma )u\right\Vert _{\underline{H}^{k-1}(\Sigma )}^2$, and $D^k_\sigma (\widehat{L}(\sigma ))$ for $k\in \mathbb {N}$ to be the closure of $C^\infty _0(\Sigma , \mathbb {C}^D)$ with respect to the graph norm $\left\Vert u\right\Vert _{\underline{H}^{k-1}_\sigma (\Sigma )}^2 + \left\Vert \widehat{L}(\sigma )u\right\Vert _{\underline{H}^{k-1}_\sigma (\Sigma )}^2$.

#### Definition 27

Given $u\in \mathcal {D}'(\Sigma , \mathbb {C}^D)$, define the *adjoint Laplace-transformed operator* $\widehat{L}^*(\sigma )$ by

$$\begin{aligned} \widehat{L}^*(\sigma ) u = \left. e^{\mathbbm {i}\overline{\sigma }t_*}L^*(e^{-\mathbbm {i}\overline{\sigma }t_*}u)\right| _{\Sigma _{t_*}}, \end{aligned}$$

where $L^*$ is as defined in (39).

Define the domain of $\widehat{L}^*(\sigma )$, $D^k(\widehat{L}^*(\sigma ))$, to be the closure of $\mathcal {D}'(\Sigma ; \mathbb {C}^D)$ with respect to the graph norm $\left\Vert u\right\Vert _{\underline{H}^{k-1}(\Sigma )}^2 + \left\Vert \widehat{L}^*(\sigma )u\right\Vert _{\underline{H}^{k-1}(\Sigma )}^2$. With this domain, $\widehat{L}^*(\sigma )$ is a closed, densely defined operator.

#### Lemma 4.5

The operator $\widehat{L}^*(\sigma )$ defined above is the adjoint of $\widehat{L}(\sigma )$ with respect to the inner product

$$\begin{aligned} \left\langle u_1, u_2\right\rangle _{L^2(\Sigma )} = \int _{\Sigma } u_1\cdot \overline{u_2}. \end{aligned}$$

#### Proof

This follows easily from Corollary 2.10 applied to ${h}_1 = e^{-\mathbbm {i}\overline{\sigma }t_*}\upsilon _1$, ${h}_2 = e^{\mathbbm {i}\overline{\sigma }t_*}\upsilon _2$, where $\upsilon _1, \upsilon _2$ are the unique stationary lifts of $u_1, u_2 \in C^\infty _0(\Sigma ; \mathbb {C}^D)$ respectively, and that $\Sigma$ is a compact interval. $\square$

The main motivation for considering the Laplace-transformed operator $\widehat{L}(\sigma )$ comes from the following lemma.

#### Lemma 4.6

Let $L$ be a strongly hyperbolic operator such that

$$\begin{aligned} L= \Box _{g} + \textbf{S}+ \textbf{V}=P_2 - \mathbbm {i}P_1D_{t_*} + \frac{1}{A} D_{t_*}^2, \end{aligned}$$

with $P_i$ as defined in (56), and let $\mathcal {A}$ be the infinitesimal generator of $L$. Then the resolvent $(\mathcal {A}-\sigma )^{-1}$ is a bounded linear operator from $\textbf{H}^{k}(\Sigma )\rightarrow D^k(\mathcal {A})$ if and only if $\widehat{L}(\sigma )^{-1}:\underline{H}^{k-1}(\Sigma )\rightarrow D^k(\widehat{L}(\sigma ))$ exists as a bounded operator with $D^k(\widehat{L}(\sigma ))\subset \underline{H}^{k}(\Sigma )$. In particular,

61 $$\begin{aligned} (\mathcal {A}- \sigma )^{-1}= -\mathbbm {i}\begin{pmatrix} 1& 0\\ -\mathbbm {i}\sigma &  -1 \end{pmatrix} \begin{pmatrix} A^{-1}\widehat{L}(\sigma )^{-1}& 0\\ 0&  -1 \end{pmatrix} \begin{pmatrix} -AP_1-\mathbbm {i}\sigma & 1\\ 1&  0 \end{pmatrix}. \end{aligned}$$

#### Proof

We can directly calculate that

$$\begin{aligned} (\mathcal {A}-\sigma ) = \mathbbm {i}\begin{pmatrix} 0&  1 \\ 1& AP_1+ \mathbbm {i}\sigma \end{pmatrix} \begin{pmatrix} A\widehat{L}(\sigma ) &  0\\ 0 &  -1 \end{pmatrix} \begin{pmatrix} 1& 0\\ -\mathbbm {i}\sigma &  -1 \end{pmatrix}. \end{aligned}$$

The first and third matrices are both invertible, with

$$\begin{aligned} \begin{pmatrix} 1& 0\\ -\mathbbm {i}\sigma &  -1 \end{pmatrix} = \begin{pmatrix} 1& 0\\ -\mathbbm {i}\sigma &  -1 \end{pmatrix}^{-1},\qquad \begin{pmatrix} -AP_1-\mathbbm {i}\sigma & 1\\ 1&  0 \end{pmatrix} = \begin{pmatrix} 0&  1 \\ 1&  AP_1+ \mathbbm {i}\sigma \end{pmatrix}^{-1}. \end{aligned}$$

Checking the domains of definition of all the operators, we see that if $\widehat{L}(\sigma )^{-1}:\underline{H}^{k-1}(\Sigma )\rightarrow D^k(\widehat{L}(\sigma ))$ is well-defined,

$$\begin{aligned} (\mathcal {A}- \sigma )^{-1}\circ (\mathcal {A}-\sigma )=\operatorname {Id}_{D^k(\mathcal {A})}, \qquad (\mathcal {A}-\sigma )\circ (\mathcal {A}-\sigma )^{-1}=\operatorname {Id}_{\textbf{H}^{k}(\Sigma )}, \end{aligned}$$

and thus, (61) holds.

To show that $(\mathcal {A}- \sigma )^{-1}$ is bounded, we first recall that $P_1$ is a bounded map from $\underline{H}^{1}(\Sigma )$ to $L^2(\Sigma )$. Now observe that the right-hand side of (61) is bounded if $\widehat{L}(\sigma )^{-1}$ is a bounded operator from $\underline{H}^{k-1}(\Sigma )$ to $\underline{H}^{k}(\Sigma )$. $\square$

Lemma 4.6 allows us to analyze the family of operators $\widehat{L}(\sigma )$ in place of $(\mathcal {A}- \sigma )$. We have a similar corollary using $\underline{H}^{k}_\sigma (\Sigma )$ norms.

#### Corollary 4.7

Fix a (not necessarily bounded) subset $\Omega \subset \mathbb {C}$. The family of resolvents $(\mathcal {A}-\sigma )^{-1}$ exists and is a bounded linear transformation of $\textbf{H}^{k}(\Sigma )$ onto $D^k(\mathcal {A})$ for all $\sigma \in \Omega$ if and only if $\widehat{L}(\sigma )^{-1}:\underline{H}^{k-1}_\sigma (\Sigma ) \rightarrow D^k_\sigma (\widehat{L}(\sigma ))$ exists as a uniformly bounded operator with $D^k_\sigma (\widehat{L}(\sigma ))\subset \underline{H}^{k}_\sigma (\Sigma )$ for all $\sigma \in \Omega$.

#### Proof

The proof follows from the expression for $(\mathcal {A}-\sigma )^{-1}$ in equation (61). $\square$

### $\textbf{H}^{k}$-Quasinormal Mode Solutions and Orthogonality

In this subsection, we define the $\textbf{H}^{k}$-quasinormal mode solutions (also known as resonant states), and establish an orthogonality condition. We then show how to relate information about the $\textbf{H}^{k}$-quasinormal spectrum back to solutions of initial value problems.

#### Assumption 4.8

Throughout this section and the remainder of Sect. 4, we assume that the linear operator $L$ and the infinitesimal generator $\mathcal {A}$ of the solution semigroup of $L$ satisfy the following properties:

1. $L$ is a strongly hyperbolic linear operator on a slowly-rotating Kerr-de Sitter background, $g_b$.
2. For any non-negative integer $k\ge 0$, the resolvent
  $$\begin{aligned} (\mathcal {A}-\sigma )^{-1}:\textbf{H}^{k}(\Sigma )\rightarrow D^k(\mathcal {A}) \end{aligned}$$
  and the dual resolvent
  $$\begin{aligned} (\mathcal {A}^{*}-\overline{\sigma })^{-1}:\textbf{H}^{k}(\Sigma )\rightarrow D^k(\mathcal {A}), \end{aligned}$$
  are finite-meromorphic operators on the half-space
  $$\begin{aligned} \Im \sigma > \frac{1}{2}\max _{\mathcal {H}= \mathcal {H}^+, \overline{\mathcal {H}}^+} \left( \textbf{s}_{L}[\mathcal {H}] - \left( 2k+\frac{1}{2}\right) \kappa _{\mathcal {H}} \right) , \end{aligned}$$
3. Moreover, there exists some ${\boldsymbol{\alpha }}>0$, and some $C>0$ such that the resolvent $(\mathcal {A}-\sigma )^{-1}:\textbf{H}^{k}(\Sigma )\rightarrow D^k(\mathcal {A})$ is a uniformly bounded operator for $\Im \sigma \ge -{\boldsymbol{\alpha }}$ and $\left\lvert \sigma \right\rvert >C$.

#### Remark 14

Since the $\textbf{H}^{k}$-quasinormal spectrum coincides with the poles of the resolvent $(\mathcal {A}-\sigma )^{-1}$ in the half-plane

$$\begin{aligned} \Im \sigma > \frac{1}{2}\max _{\mathcal {H}= \mathcal {H}^+, \overline{\mathcal {H}}^+} \left( \textbf{s}_{L}[\mathcal {H}] - \left( 2k+\frac{1}{2}\right) \kappa _{\mathcal {H}} \right) , \end{aligned}$$

and since the poles of a meromorphic function are discrete, the second assumption in Assumption 4.8 ensures that the $\textbf{H}^{k}$-quasinormal spectrum of $L$ is discrete.

#### Remark 15

As will be shown later in Sect. 5.2, the linearized gauge-fixed Einstein operator $\mathbb {L}_{g_b}$ does indeed satisfy the above assumptions. See Theorems 5.3 and 5.4.

We define the $\textbf{H}^{k}$-*resonant states*, or $\textbf{H}^{k}$-*quasinormal mode solutions*, of $L$. Consider some ${h} = ({h}_0, {h}_1)$ a $\sigma _0$-frequency $\textbf{H}^{k}$-quasinormal mode of $L$ for some $\sigma _0\in \Lambda _{\operatorname {QNF}}^{k}(L)$. Using Lemma 4.6, we see that $({h}_0, {h}_1)$ must satisfy

$$\begin{aligned} \widehat{L}(\sigma _0){h}_0 = 0,\qquad -\mathbbm {i}\sigma _0 {h}_0 = {h}_1. \end{aligned}$$

#### Definition 28

Given some $\sigma _0\in \Lambda _{\operatorname {QNF}}^{k}(L)$, we define the space of $\textbf{H}^{k}$-*quasinormal mode solutions* of $L$ with frequency $\sigma _0$ to be the set

$$\begin{aligned} \Lambda _{\operatorname {QNM}}^{k}(L, \sigma _0)\! =\! \left\{ \textbf{v}\!=\!\sum _{k=0}^n e^{\!-\!\mathbbm {i}\sigma _0t_*}t_*^k \textbf{u}_k(x): n\in \mathbb {N}_0, (D_{t_*} \!-\!\mathcal {A})\textbf{v} \!=\! 0, \textbf{u}_k\in C^\infty (\Sigma )\right\} . \end{aligned}$$

Furthermore for a subset $\Xi \subset \left\{ \sigma \in \mathbb {C}: \Im \sigma > \max _{\mathcal {H}=\mathcal {H}^+,\overline{\mathcal {H}}^+}\left( \frac{1}{2}\textbf{s}_{L}[\mathcal {H}] + (\frac{1}{2}-k)\right. \right\}$ ${\left. \kappa _{\mathcal {H}}\right) }$, which contains only finitely many resonances of $L$, we can define the set of $\textbf{H}^{k}$-quasinormal mode solutions with frequencies in $\Xi$ by

$$\begin{aligned} \Lambda _{\operatorname {QNM}}^{k}(L, \Xi ) {:=} \bigoplus _{\sigma \in \Xi \bigcap \Lambda _{\operatorname {QNF}}^{k}(L)}\Lambda _{\operatorname {QNM}}^{k}(L, \sigma ). \end{aligned}$$

It will be useful to have a frequency-space characterization of the space of $\textbf{H}^{k}$-quasinormal mode solutions of $L$.

#### Proposition 4.9

Fix $$\sigma _0\in \Lambda _{\operatorname {QNF}}^{k}(L)$$. Then an equivalent characterization of $$\Lambda _{\operatorname {QNM}}^{k}$$$$(L, \sigma _0)$$ is the set

62 $$\begin{aligned} \Lambda _{\operatorname {QNM}}^{k}(L, \sigma _0) = \left\{ {\text {res}}_{\sigma =\sigma _0}\left( e^{-\mathbbm {i}\sigma t_*}(\mathcal {A}- \sigma )^{-1}p(\sigma )\right) : p(\sigma )\in P\left( \sigma , C^\infty (\Sigma )\right) \right\} , \end{aligned}$$

where we denote by $P(\sigma , C^{\infty }(\Sigma ))$ the set of all polynomials in $\sigma$ with coefficients in $C^{\infty }(\Sigma )$.

#### Proof

See Appendix D.2. $$\square $$

We can likewise characterize the dual $\textbf{H}^{k}$-quasinormal mode solutions of $L$ using the $\left\langle \cdot ,\cdot \right\rangle _{L^2(\Sigma )}$ inner product,

$$\begin{aligned} \begin{aligned} \Lambda _{\operatorname {QNM}}^{k*}(L, \sigma ) = \left\{ \textbf{v} = \sum _{k=0}^{n}e^{-\mathbbm {i}\overline{\sigma }t_*}t_*^k\textbf{u}_k(x): n\in \mathbb {N}_0, (D_{t_*} - \mathcal {A}^*)\textbf{v} = 0, \textbf{u}_k\in \mathcal {E}'(\Sigma ) \right\} . \end{aligned} \end{aligned}$$

Following the same reasoning as above, we also have the frequency-space characterization of the dual $\textbf{H}^{k}$-quasinormal modes

$$\begin{aligned} \begin{aligned} \Lambda _{\operatorname {QNM}}^{k*}(L, \sigma ) ={}&\left\{ {\text {res}}_{\zeta = \overline{\sigma }}\left( e^{-\mathbbm {i}\zeta t_*}(\mathcal {A}^*-\zeta )^{-1}p(\zeta )\right) : p(\zeta )\in P(\zeta , \mathcal {E}'(\Sigma )) \right\} , \end{aligned} \end{aligned}$$

where $\mathcal {A}^*$ is the infinitesimal generator of the dual solution semigroup of $L$.

We can then establish an orthogonality condition to a finite set of $\textbf{H}^{k}$-quasinormal frequencies (see the similar Proposition 5.7, Corollary 5.8 in [31] formulated at the level of the Laplace-transformed operator).

#### Proposition 4.10

Let $\Xi =\left\{ \sigma _j\right\} _{j=1}^{N_\Xi }\subset \Lambda _{\operatorname {QNF}}^{k}(L)$ be a finite set of $\textbf{H}^{k}$-quasinormal frequencies, and fix $\beta > \max \left\{ -\Im \sigma _j: \sigma _j\in \Xi \right\}$. Then define the continuous linear map^12^

$$\begin{aligned} \begin{aligned} \lambda : H^{-1, \beta }(\mathbb {R}_+, \textbf{H}^{k-1}(\Sigma ))&\rightarrow \mathcal {L}(\Lambda _{\operatorname {QNM}}^{k*}(L, \Xi ), \overline{\mathbb {C}}),\\ F&\mapsto \left\langle F, \cdot \right\rangle _{L^2(\mathcal {M})}, \end{aligned} \end{aligned}$$

mapping *F* to a $\mathbb {C}$-anti-linear function on $\mathcal {L}(\Lambda _{\operatorname {QNM}}^{k*}(L, \Xi ))$.

Then $\lambda (F) = 0$ if and only if $(\mathcal {A}-\sigma )^{-1}\widehat{F}(\sigma )$ is holomorphic in a neighborhood of $\Xi$.

#### Proof

See Appendix D.3. $$\square $$

### Quasinormal Spectrum and the Initial Value Problem

We are now ready to use the $\textbf{H}^{k}$-quasinormal spectrum to analyze the Cauchy problem given by

63 $$\begin{aligned} {\left\{ \begin{array}{ll} L{h} & = f, \\ \gamma _0({h}) & = ({h}_0,{h}_1). \end{array}\right. } \end{aligned}$$

We will require that the forcing term and initial data of the Cauchy problem in (63) have certain decay and regularity properties.

#### Definition 29

Let $k,\alpha \in \mathbb {R}$. Then we define the space of data with regularity *k*,and decay $\alpha$ to be

$$\begin{aligned} D^{k,\alpha }(\mathcal {M}){:=} H^{k-1,\alpha }(\mathcal {M}) \oplus \textbf{H}^{k}(\Sigma _0). \end{aligned}$$

We then define the norm

$$\begin{aligned} \left\Vert (f, {h}_0, {h}_1)\right\Vert _{D^{k,\alpha }(\mathcal {M})} {:=} \left\Vert f\right\Vert _{H^{k-1,\alpha }(\mathcal {M})} + \left\Vert ({h}_0, {h}_1)\right\Vert _{\textbf{H}^{k}(\Sigma _0)}. \end{aligned}$$

We have the first preliminary expression of ${h}(t_*)$ in terms of the resolvent $(\mathcal {A}-\sigma )^{-1}$.

#### Lemma 4.11

Let $L$ be a strongly hyperbolic linear operator on a slowly-rotating Kerr-de Sitter background *g*, and let $(f, {h}_0, {h}_1)\in D^{k,\alpha }(\mathcal {M})$ for some $k, \alpha >0$

. Then if *h* is a solution to the Cauchy problem (63), there exists some $\textbf{M}>0$ such that

64 $$\begin{aligned} \textbf{h}(t_*) = \int _{\Im \sigma =\textbf{M}}e^{-\mathbbm {i}\sigma t_*}(\mathcal {A}- \sigma )^{-1}\widehat{F}(\sigma )\,d\sigma , \end{aligned}$$

where

65 $$\begin{aligned} \textbf{h} = \begin{pmatrix} {h}\\ \textbf{T}{h} \end{pmatrix},\qquad F(t_*) = \frac{1}{\mathbbm {i}} \begin{pmatrix} \delta _0(t_*){h}_0\\ - Af(t_*) + \delta _0(t_*){h}_1 \end{pmatrix}, \end{aligned}$$

where $\delta _0$ is the Dirac delta, and

$$\begin{aligned} \widehat{F}(\sigma ) = \int _0^{\infty } F(t_*)e^{\mathbbm {i}\sigma t_*}\,dt_*\end{aligned}$$

denotes the Laplace transform of *F*.

#### Proof

From Duhamel’s principle we have that

66 $$\begin{aligned} \textbf{h}(t_*, \cdot ) = \int _{0}^{t_*} \mathcal {S}(t_*-{s_*}) \begin{pmatrix} \delta _0({s_*}){h}_0(\cdot )\\ - Af({s_*}, \cdot ) + \delta _0({s_*}){h}_1(\cdot ) \end{pmatrix}\,d{s_*}\end{aligned}$$

is a solution to the Cauchy problem in (63). Using Lemma A.2 and letting the limits of integration tend to $+\infty$, we have that for $\Im \sigma >\textbf{M}$,

$$\begin{aligned} (\mathcal {A}-\sigma )^{-1}\widehat{F}(\sigma ) = \mathbbm {i}\int _{\mathbb {R}^+}e^{\mathbbm {i}\sigma {s_*}}\mathcal {S}({s_*})\widehat{F}(\sigma )\,d{s_*}. \end{aligned}$$

As a result, Laplace-transforming both sides of (66), we have that for $\Im \sigma > \textbf{M}$,

$$\begin{aligned} \widehat{\textbf{h}}(\sigma ) = (\mathcal {A}- \sigma )^{-1}\widehat{F}(\sigma ). \end{aligned}$$

As a result at the cost of slightly increasing $\textbf{M}$, we can take the inverse Laplace transform over the contour $\Im \sigma =\textbf{M}$, and

67 $$\begin{aligned} \textbf{h}(t_*) = \int _{\Im \sigma =\textbf{M}}e^{-\mathbbm {i}\sigma t_*}(\mathcal {A}- \sigma )^{-1}\widehat{F}(\sigma )\,d\sigma , \end{aligned}$$

as desired. $$\square $$

Next, we show that under certain conditions on the resolvent $(\mathcal {A}-\sigma )^{-1}$, solutions to the Cauchy problem decay exponentially up to a finite number of non-decaying linear obstacles.

#### Proposition 4.12

Let $L$ be some strongly hyperbolic linear operator satisfying the assumptions in Assumption 4.8. Also fix $k_0>0$ such that

$$\begin{aligned} -{\boldsymbol{\alpha }}>\frac{1}{2}\max _{\mathcal {H}=\mathcal {H}^+,\overline{\mathcal {H}}^+}\left( \textbf{s}_{\mathbb {L}}[\mathcal {H}] - \left( 2k_0 + \frac{1}{2} \right) \kappa _{\mathcal {H}}\right) \end{aligned}$$

where ${\boldsymbol{\alpha }}$ is specified as in Assumption 4.8.

Furthermore for $k\ge k_0$, let $\Xi = \left\{ \sigma _j\right\} _{j=1}^{N_{L}}$ denote the set of all $\textbf{H}^{k}$-quasinormal frequencies of $L$ with $\Im \sigma >-{\boldsymbol{\alpha }}$. Let $(f, h_0, h_1)\in D^{k+1,{\boldsymbol{\alpha }}}(\mathcal {M})$, and let *h* be the solution to the Cauchy problem given by

$$\begin{aligned} \begin{aligned} Lh&= f \\ \gamma _0(h)&= (h_0, h_1). \end{aligned} \end{aligned}$$

For $$\textbf{v} = \begin{pmatrix} h\\ \textbf{T}h \end{pmatrix}$$, we have that

68 $$\begin{aligned} \textbf{v} = \tilde{\textbf{v}} + \sum _{j=1}^{N_{L}}\sum _{\ell =0}^{d_j-1}e^{-\mathbbm {i}\sigma _jt_*}t_*^{\ell } \textbf{u}_{j\ell }(x), \end{aligned}$$

where $d_j$ is the multiplicity of $\sigma _j$ (i.e. $\dim \Lambda _{\operatorname {QNM}}^{k}(L, \sigma _j)$), $\displaystyle \sum _{j=1}^{N_{L}}\sum _{\ell =0}^{d_j-1}e^{-\mathbbm {i}\sigma _jt_*}t_*^\ell \textbf{u}_{j\ell }(x) \in \Lambda _{\operatorname {QNM}}^{k}(L, \Xi )$, and where $\tilde{\textbf{v}}$ satisfies the decay bound

$$\begin{aligned} \begin{aligned} \left\Vert \tilde{\textbf{v}}\right\Vert _{\textbf{H}^{k}(\Sigma _{t_*})} \lesssim e^{-{\boldsymbol{\alpha }}t_*} \left\Vert (f, h_0, h_1)\right\Vert _{D^{k+1, {\boldsymbol{\alpha }}}(\mathcal {M})}, \end{aligned} \end{aligned}$$

for $k>k_0$, and where there is a continuous mapping

$$\begin{aligned} (f, h_0, h_1)\mapsto \sum _{j=1}^{N_{L}}\sum _{\ell =0}^{d_j-1}e^{-\mathbbm {i}\sigma _jt_*}t_*^\ell \textbf{u}_{j\ell }(x). \end{aligned}$$

#### Remark 16

Observe that as written, the individual summands $e^{-\mathbbm {i}\sigma _jt_*}t_*^\ell \textbf{u}_{j\ell }$ in (68) are not necessarily $\textbf{H}^{k}$-quasinormal modes. However, we can rewrite

$$\begin{aligned} \sum _{j=1}^{N_{L}}\sum _{\ell =0}^{d_j-1}e^{-\mathbbm {i}\sigma _jt_*}t_*^\ell \textbf{u}_{j\ell }(x) = \sum _{j=1}^{N_{L}}\sum _{\ell =0}^{d_j-1}a_{j\ell }\left( \sum _{k=0}^{n_{j\ell }}e^{-\mathbbm {i}\sigma _jt_*}t_*^k\tilde{\textbf{u}}_{j\ell k}\right) , \end{aligned}$$

where $a\in \mathbb {R}$, and $\sum _{k=0}^{n_{j\ell }}e^{-\mathbbm {i}\sigma _jt_*}t_*^k\tilde{\textbf{u}}_{j\ell k}$ are indeed $\textbf{H}^{k}$-quasinormal mode solutions.

#### Proof

Recall from Lemma 4.11 that denoting by $\mathcal {A}$ the infinitesimal generator of the solution semigroup associated to $L$, we can write

$$\begin{aligned} \textbf{h}(t_*){:=} \begin{pmatrix} {h}(t_*)\\ \textbf{T}{h}(t_*) \end{pmatrix} = \int _{\Im \sigma =\textbf{M}}e^{-\mathbbm {i}\sigma t_*}(\mathcal {A}- \sigma )^{-1}\widehat{F}(\sigma )\,d\sigma , \end{aligned}$$

where

$$\begin{aligned} F(t_*, x) =\frac{1}{\mathbbm {i}} \begin{pmatrix} \delta _0(t_*){h}_0\\ - Af(t_*) + \delta _0(t_*){h}_1 \end{pmatrix}. \end{aligned}$$

Recall from Lemma 4.11 that $(\mathcal {A}- \sigma )^{-1}$ is a holomorphic family of operators for $\Im \sigma >\textbf{M}$. We have that $(\mathcal {A}-\sigma )^{-1}\widehat{F}(\sigma )$ is meromorphic with only finitely many poles on the half-plane $\Im \sigma > -{\boldsymbol{\alpha }}$, we now deform the contour of the inverse Fourier transform in (64) from $\Im \sigma = \textbf{M}$ to $\Im \sigma = -{\boldsymbol{\alpha }}$. To this end, consider the contours

$$\begin{aligned} \gamma _{\pm C} = \{\pm C + \mathbbm {i}s: -{\boldsymbol{\alpha }}\le s\le \textbf{M}\}. \end{aligned}$$

Using the construction of the Laplace-transformed norms

69 $$\begin{aligned} \left\Vert (\mathcal {A}- \sigma )^{-1}\widehat{F}(\sigma )\right\Vert _{\textbf{H}^{k}_\sigma (\Sigma )} \lesssim \frac{1}{(1+\left\lvert \sigma \right\rvert )} \left\Vert (\mathcal {A}- \sigma )^{-1}\widehat{F}(\sigma )\right\Vert _{\textbf{H}^{k+1}_\sigma (\Sigma )}. \end{aligned}$$

By assumption $(\mathcal {A}-\sigma )^{-1}$ is a uniformly bounded operator for $\Im \sigma >-{\boldsymbol{\alpha }}$, $\left\lvert \sigma \right\rvert >C$. Thus we know that for *C* and *k* sufficiently large,

70 $$\begin{aligned} \left\Vert (\mathcal {A}- \sigma )^{-1}\widehat{F}(\sigma )\right\Vert _{\textbf{H}^{k}_{\sigma }(\Sigma )} \lesssim \left\Vert \widehat{F}(\sigma )\right\Vert _{\textbf{H}^{k}_{\sigma }(\Sigma )} \lesssim \left\Vert (f, h_0, h_1)\right\Vert _{D^{k, -\Im \sigma }(\mathcal {M})}. \end{aligned}$$

As a result,

$$\begin{aligned} \left\Vert \int _{\gamma _{\pm C}}e^{-\mathbbm {i}\sigma t_*}(\mathcal {A}- \sigma )^{-1}\widehat{F}(\sigma )\right\Vert _{\textbf{H}^{k}_{\sigma }(\Sigma )}\,d\sigma \lesssim \frac{e^{\textbf{M}t_*}}{1+C}\left\Vert (f, h_0, h_1)\right\Vert _{D^{k+1, {\boldsymbol{\alpha }}}(\mathcal {M})}, \end{aligned}$$

and for *k* sufficient large, it is clear that for all $t_*>0$,

$$\begin{aligned} \lim _{C\rightarrow \infty }\int _{\gamma _{\pm C}}e^{-\mathbbm {i}\sigma t_*}(\mathcal {A}- \sigma )^{-1}\widehat{F}(\sigma )\,d\sigma = 0. \end{aligned}$$

Then using Cauchy’s integral formula we can perturb the contour of integration to obtain that

71 $$\begin{aligned} \textbf{h}(t_*, \cdot ) \!=\!{}\!\!\int _{\Im \sigma = -{\boldsymbol{\alpha }}} e^{-\mathbbm {i}\sigma t_*}(\mathcal {A}\!-\!\sigma )^{-1}\widehat{F}(\sigma )d\sigma \!\!+\!\! \sum _{1\le j\le N_{L}} {\text {res}}_{\zeta = \sigma _j}\left( e^{-\mathbbm {i}\zeta t_*}(\mathcal {A}\!-\!\!\zeta )^{-1}\widehat{F}(\zeta ) \right) . \end{aligned}$$

From (70), we see that

$$\begin{aligned} \left\Vert \int _{\Im \sigma = -{\boldsymbol{\alpha }}} e^{-\mathbbm {i}\sigma t_*}(\mathcal {A}-\sigma )^{-1}\widehat{F}(\sigma )\,d\sigma \right\Vert _{\textbf{H}^{k}_{\sigma }(\Sigma )} \lesssim e^{-{\boldsymbol{\alpha }}t_*}\left\Vert (f, h_0, h_1)\right\Vert _{D^{k+1, {\boldsymbol{\alpha }}}(\mathcal {M})}, \end{aligned}$$

while we can recall from Proposition 4.9 that

$$\begin{aligned} \sum _{1\le j\le N_{L}} {\text {res}}_{\zeta = \sigma _j}\left( e^{-\mathbbm {i}\zeta t_*}(\mathcal {A}-\zeta )^{-1}\widehat{F}(\zeta ) \right) \in \Lambda _{\operatorname {QNF}}^{k}(L, \Xi ) \end{aligned}$$

where $\Xi$ is some open subset containing all the non-decaying $\textbf{H}^{k}$-quasinormal modes of $L$. Thus, *h* has exactly the desired form in (68). $\square$

Next we show that we can produce exponentially decaying solutions to the Cauchy problem provided we can modify the forcing term and the initial data within some finite-dimensional space of modifications (compare with Corollary 5.8 in [31]).

#### Corollary 4.13

Let $L$ be some strongly hyperbolic linear operator satisfying Assumption 4.8, and fix $k_0>0$ such that

$$\begin{aligned} -{\boldsymbol{\alpha }}>\frac{1}{2}\max _{\mathcal {H}=\mathcal {H}^+,\overline{\mathcal {H}}^+}\left( \textbf{s}_{\mathbb {L}}[\mathcal {H}] - \left( 2k_0 + \frac{1}{2} \right) \kappa _{\mathcal {H}}\right) \end{aligned}$$

with ${\boldsymbol{\alpha }}$ as in Assumption 4.8.

Then, for $k> k_0$, let $\Xi = \left\{ \sigma _j\right\} _{j=1}^{N_{L}}$ denote the set of all $\textbf{H}^{k}$-quasinormal frequencies of $L$ with $\Im \sigma >-{\boldsymbol{\alpha }}$, and let $\mathcal {Z}\subset D^{k,{\boldsymbol{\alpha }}}(\mathcal {M})$ be a finite-dimensional linear subspace. We define the map

72 $$\begin{aligned} \begin{aligned} \lambda _{IVP}: D^{k,{\boldsymbol{\alpha }}}(\mathcal {M})&\rightarrow \mathcal {L}(\Lambda _{\operatorname {QNM}}^{k*}(L, \Xi ), \overline{\mathbb {C}})\\ \lambda _{IVP}(f, {h}_0, {h}_1)&{:=} \lambda \left( \frac{1}{\mathbbm {i}} \begin{pmatrix} \delta _0 h_0\\ -Af+\delta _0{h}_1 \end{pmatrix}\right) , \end{aligned} \end{aligned}$$

where $\lambda$ is as constructed in Proposition 4.10, $\lambda _{\mathcal {Z}}$ denotes its restriction to $\mathcal {Z}$, and $\delta _0 = \delta _0(t_*)$ denotes the Dirac delta,

$$\begin{aligned} \lambda _{\mathcal {Z}}: \mathcal {Z}\rightarrow \mathcal {L}(\Lambda _{\operatorname {QNM}}^{k*}(L, \Xi ), \overline{\mathbb {C}}), \qquad \lambda _{\mathcal {Z}}{:=}\lambda _{IVP}\vert _{\mathcal {Z}}. \end{aligned}$$

Then, if $\lambda _{\mathcal {Z}}$ is surjective, for any choice of $(f, {h}_0, {h}_1)\in D^{k, {\boldsymbol{\alpha }}}(\mathcal {M})$, there exists an element $z = (\tilde{f}, \tilde{{h}}_0, \tilde{{h}}_1) \in \mathcal {Z}$ such that the initial value problem

$$\begin{aligned} \begin{aligned} L{h}&= f + \tilde{f},\\ \gamma _0({h})&= ({h}_0 + \tilde{{h}}_0, {h}_1 + \tilde{{h}}_1) \end{aligned} \end{aligned}$$

has an exponentially decaying solution *h* that satisfies the estimate

73 $$\begin{aligned} \left\Vert h\right\Vert _{\overline{H}^{k}(\Sigma _{t_*})} \lesssim e^{-{\boldsymbol{\alpha }}t_*} \left\Vert (f, {h}_0, {h}_1)\right\Vert _{D^{k+1,{\boldsymbol{\alpha }}}(\mathcal {M})}. \end{aligned}$$

If moreover, $\lambda _{\mathcal {Z}}$ is bijective, then *z* is unique and the map $(f, {h}_0, {h}_1) \rightarrow z$ is continuous.

#### Proof

As in Proposition 4.12, we perturb the contour of integration in (64) to write that for $$\textbf{h} = \begin{pmatrix} {h}\\ \textbf{T}{h} \end{pmatrix}$$, where *h* is a solution to (63),

74 $$\begin{aligned} \textbf{h}(t_*, \cdot ) \!=\!\int _{\Im \sigma = -{\boldsymbol{\alpha }}} e^{-\mathbbm {i}\sigma t_*}(\mathcal {A}\!\!-\!\!\sigma )^{-1}\widehat{F}(\sigma )\,d\sigma \!+\!\! \sum _{1\le j\le N_{L}} {\text {res}}_{\zeta = \sigma _j}\left( e^{-\mathbbm {i}\zeta t_*}(\mathcal {A}\!-\!\zeta )^{-1}\widehat{F}(\zeta ) \right) , \end{aligned}$$

where $F(t_*, x)$ is as defined in (65).

Now we consider finding some $z\in \mathcal {Z}$ for which

$$\begin{aligned} \lambda _{\mathcal {Z}}(z) = - \lambda _{IVP}(f, {h}_0, {h}_1)\in \mathcal {L}(\Lambda _{\operatorname {QNM}}^{k*}(L, \Xi ), \overline{\mathbb {C}}). \end{aligned}$$

This is clearly possible if $\lambda _{\mathcal {Z}}$ is surjective. If in addition, $\lambda _{\mathcal {Z}}$ is bijective, then we can consider the mapping $(f, {h}_0, {h}_1)\mapsto z$ defined by

$$\begin{aligned} \lambda _{\mathcal {Z}}^{-1}\circ \lambda _{IVP}(f, {h}_0, {h}_1) = z \end{aligned}$$

which is clearly a linear and continuous, and therefore bounded, map. Moreover, if

$$\begin{aligned} z = \lambda _{\mathcal {Z}}^{-1}\circ \lambda _{IVP}(f, {h}_0, {h}_1) = (\tilde{f}, \tilde{{h}}_0, \tilde{{h}}_1), \end{aligned}$$

then by construction

$$\begin{aligned} \tilde{F} = \frac{1}{\mathbbm {i}}\begin{pmatrix} \delta _0({h}_0+\tilde{{h}}_0)\\ -A(f + \tilde{f}) + \delta _0({h}_1 + \tilde{{h}}_1) \end{pmatrix}, \end{aligned}$$

satisfies

$$\begin{aligned} \sum _{1\le j\le N_{L}} {\text {res}}_{\zeta = \sigma _j}\left( e^{-\mathbbm {i}\zeta t_*}(\mathcal {A}-\zeta )^{-1}\widehat{\tilde{F}}(\zeta ) \right) = 0. \end{aligned}$$

As a result, using (74), we have that

$$\begin{aligned} \textbf{h}(t_*, \cdot ) = \int _{\Im \sigma = -{\boldsymbol{\alpha }}} e^{-\mathbbm {i}\sigma t_*}(\mathcal {A}-\sigma )^{-1}\widehat{\tilde{F}}(\sigma )\,d\sigma , \end{aligned}$$

and the bound in (73) immediately follows from Plancherel. $\square$

### Perturbation Theory of the Quasinormal Spectrum

In this section, we explore the perturbation theory for the $\textbf{H}^{k}$-quasinormal frequencies and modes. To this end, let us consider a family of stationary strongly hyperbolic linear operators $\{L_w\}_{w\in W}$ on a family of slowly-rotating Kerr-de Sitter backgrounds *b*(*w*), where $W\subset \mathbb {R}^{N_W}$ is a finite dimensional open neighborhood of some fixed $w_0\in \mathbb {R}^{N_W}$.

Let $\mathcal {A}_w$ be the infinitesimal generator associated to the operator $L_w$. In what follows, we will apply the results of this section to both the gauge-fixed linearized Einstein operator $\mathbb {L}_{g_b}$, and the constraint propagation operator $\Box ^{CP}_{g_b}$, perturbing results obtained on a fixed Schwarzschild-de Sitter background to a nearby Kerr-de Sitter background (see Sect. 10.3).

The following proposition is a collection of the main basic perturbation theory we will use, and is analogous to Proposition 5.11 in [31]. We have restated the result below in terms of the infinitesimal generators $\mathcal {A}_w$.

#### Proposition 4.14

Let $k>0$ for some fixed $k_0$ such that

75 $$\begin{aligned} \sup _{w\in W; \mathcal {H}= \mathcal {H}^+, \overline{\mathcal {H}}^+}\left( \frac{1}{2}\textbf{s}_{L_{b(w)}}[\mathcal {H}] -\left( k_0 - \frac{1}{4}\right) \kappa _{b(w), \mathcal {H}}\right) < 0, \end{aligned}$$

and let

$$\begin{aligned} \Omega \subset \left\{ \sigma \in \mathbb {C}: \Im \sigma >\max _{\mathcal {H}=\mathcal {H}^+, \overline{\mathcal {H}}^+} \frac{1}{2}\textbf{s}_{L_{w_0}}[\mathcal {H}] - \left( k-\frac{1}{4}\right) \kappa _{b_0,\mathcal {H}} \right\} \end{aligned}$$

be a non-empty pre-compact set such that

$$\begin{aligned} \Lambda _{\operatorname {QNF}}^{k}(L_{w_0}) \cap \partial \Omega = \emptyset . \end{aligned}$$

Then the following hold.

1. The set $I{:=}\left\{ (w,\sigma )\in W\times \Omega : (\mathcal {A}_w - \sigma )^{-1}{\text { is bounded}}\right\}$ is open.
2. Let $\mathcal {L}_{weak}(X_1, X_2)$ denote the space of bounded linear operators mapping $X_1\rightarrow X_2$ equipped with the weak operator topology, and $\mathcal {L}_{op}(X_1, X_2)$ the space of bounded linear operators mapping $X_1 \rightarrow X_2$ equipped with the norm topology. Then the map
  $$\begin{aligned} I\ni (w,\sigma )\mapsto (\mathcal {A}_w - \sigma )^{-1} \in \mathcal {L}_{weak}(\textbf{H}^{k}(\Sigma ), D^{k}(\mathcal {A})) \end{aligned}$$
  is continuous for all $k>k_0$ and also as a map into $\mathcal {L}_{op}(\textbf{H}^{k+\epsilon }(\Sigma ), D^{k-\epsilon }(\mathcal {A}))$.
3. The set $\Lambda _{\operatorname {QNF}}^{k}(L_w)\bigcap \Omega$ depends continuously on *w* in the Hausdorff distance sense, and the total rank
  $$\begin{aligned} d{:=} \sum _{\sigma \in \Lambda _{\operatorname {QNF}}^{k}(L_w)\bigcap \Omega }\operatorname {rank}_{\zeta =\sigma } (\mathcal {A}_w-\zeta )^{-1} \end{aligned}$$
  is constant for *w* near $w_0$, where
  $$\begin{aligned} \operatorname {rank}_{\zeta =\sigma } (\mathcal {A}_w-\zeta )^{-1} = \dim \Lambda _{\operatorname {QNM}}^{k}(L, \sigma ). \end{aligned}$$
4. The total space of quasinormal modes $\Lambda _{\operatorname {QNM}}^{k}(L_w, \Omega )\subset C^\infty (\mathcal {M})$ depends continuously on *w* in the sense that there exists a continuous map
  $$\begin{aligned} W\times \mathbb {C}^d\rightarrow C^\infty (\mathcal {M}) \end{aligned}$$
  such that $\Lambda _{\operatorname {QNF}}^{k}(L_w, \Omega )$ is the image of $\{w\}\times \mathbb {C}^d$.
5. Likewise, the total space of dual states $\Lambda _{\operatorname {QNM}}^{k*}(L_w, \Omega )\subset H_{loc}^{1-k_0}(\mathcal {M})$ depends continuously on *w*.

#### Proof

See Appendix D.4. $$\square $$

## Main Theorem

### Statement of the Main Theorem

We are now ready to state the main theorem.

#### Theorem 5.1

(Main Theorem, version 2) Fix $k>k_0$, where $k_0$ is as defined in (79). Then, let $(\underline{g}', k')\in H^{k+1}(\Sigma _0; S^2T^*\Sigma _0)\oplus H^{k}(\Sigma _0; S^2T^*\Sigma _0)$ be solutions of the linearized constraint equations linearized around the initial data $(\underline{g}_{b}, k_{b})$ of a slowly rotating Kerr-de Sitter background $(\mathcal {M}, g_b)$. Then there exists a solution *h* to the initial value problem

$$\begin{aligned} {\left\{ \begin{array}{ll} \mathbb {L}_{g_b} {h} =0 & {\text {in }}\mathcal {M},\\ \gamma _0({h}) = D_{(\underline{g}_b, k_b)}i_{b,\operatorname {Id}}(\underline{g}', k')& {\text {on }}\Sigma _0, \end{array}\right. } \end{aligned}$$

with $i_{b, \operatorname {Id}}$ defined in Proposition 3.6. Moreover, there exists some ${\boldsymbol{\alpha }}>0$, some finite-dimensional family of 1-forms $\Theta \in C^\infty (\mathcal {M})$ parametrized by $\vartheta : \mathbb {R}^{N_\Theta }\rightarrow \Theta$, and some $b'\in T_bB, \omega \in \mathbb {R}^{N_{\Theta }}$ such that

$$\begin{aligned} {h} = g_b'(b') + \nabla _{g_b}\otimes \vartheta (\omega ) + \tilde{{h}}, \qquad g_b'(b'){:=} \frac{\partial g_b}{\partial b}b', \end{aligned}$$

where $\tilde{{h}}$ satisfies the bounds

$$\begin{aligned} \sup _{t_*}e^{{\boldsymbol{\alpha }}t_*}\left\Vert \tilde{h}\right\Vert _{\overline{H}^{k}(\Sigma _{t_*})} \lesssim \left\Vert \underline{g}'\right\Vert _{H^{k+1}(\Sigma _0)} + \left\Vert k'\right\Vert _{H^{k}(\Sigma _0)}, \end{aligned}$$

and $b'$ and $\omega$ are small in the sense that

$$\begin{aligned} \left\lvert b'\right\rvert + \left\lvert \omega \right\rvert \lesssim \left\Vert \underline{g}'\right\Vert _{H^{k+1}(\Sigma _0)} + \left\Vert k'\right\Vert _{H^{k}(\Sigma _0)}. \end{aligned}$$

The main theorem in the case of $g_{b}=g_{b_0}$ a Schwarzschild-de Sitter metric will be proven in Sect. 10.2, and the general slowly-rotating case in Sect. 11.

#### Corollary 5.2

Fix *k* as in Theorem 5.1, and $(f, h_0, h_1)\in D^{k,{\boldsymbol{\alpha }}}(\mathcal {M})$, where ${\boldsymbol{\alpha }}$ is as in Theorem 5.1. Moreover, assume that there exists some ${T_*}>0$ such that for all $t_*>{T_*}$,

$$\begin{aligned} f(t_*, \cdot ) = 0 ,\qquad \left. \mathcal {C}_{g_b}(h)\right| _{\Sigma _{t_*}}=0, \end{aligned}$$

and let *h* be the solution to the Cauchy problem

76 $$\begin{aligned} \begin{aligned} \mathbb {L}_{g_b}h&= f,\\ \gamma _0(h)&= (h_0, h_1). \end{aligned} \end{aligned}$$

Then, there exists some ${\boldsymbol{\alpha }}>0$, some finite-dimensional family of 1-forms $\Theta \in C^\infty (\mathcal {M})$ parametrized by $\vartheta : \mathbb {R}^{N_\Theta }\rightarrow \Theta$, and some $b'\in T_bB, \omega \in \mathbb {R}^{N_{\Theta }}$ such that

$$\begin{aligned} {h} = g_b'(b') + \nabla _{g_b}\otimes \vartheta (\omega ) + \tilde{{h}}, \qquad g_b'(b'){:=} \frac{\partial g_b}{\partial b}b', \end{aligned}$$

where $\tilde{{h}}$ satisfies the bounds

$$\begin{aligned} \sup _{t_*}e^{{\boldsymbol{\alpha }}t_*}\left\Vert \tilde{h}\right\Vert _{\overline{H}^{k}(\Sigma _{t_*})} \lesssim \left\Vert \underline{g}'\right\Vert _{H^{k+1}(\Sigma _0)} + \left\Vert k'\right\Vert _{H^{k}(\Sigma _0)}, \end{aligned}$$

and $b'$ and $\omega$ are small in the sense that

77 $$\begin{aligned} \left\lvert b'\right\rvert + \left\lvert \omega \right\rvert \lesssim \left\Vert \underline{g}'\right\Vert _{H^{k+1}(\Sigma _0)} + \left\Vert k'\right\Vert _{H^{k}(\Sigma _0)}. \end{aligned}$$

### Main Intermediary Results

In this section, we describe the main intermediary steps taken in proving Theorem 5.1.

1. A Fredholm alternative result on the $\textbf{H}^{k}$-quasinormal modes for the gauge-fixed linearized Einstein operator $\mathbb {L}_{g_b}$, which gives us the discreteness of the $\textbf{H}^{k}$-quasinormal spectrum, and the finite-meromorphy of the resolvent $(\mathcal {A}-\sigma )^{-1}$. See Theorem 5.3.
2. A high-frequency estimate for the resolvent $(\mathcal {A}-\sigma )^{-1}$ on a region of the complex plane, proving the existence of a spectral gap underneath the real axis, and $\textbf{H}^{k}$-quasinormal-mode-free regions of $\mathbb {C}$. See Theorem 5.4.
3. A mode stability statement for the non-gauge-fixed linearized Einstein operator, which allows us to characterize the non-decaying modes of the gauge-fixed linearized Einstein operator as unphysical. See Theorem 5.6.

#### Remark 17

We remark that the first two steps are the latter two assumptions in Assumption 4.8.

Recall that we denote by $\mathbb {L}_{g_b}$ the gauge-fixed linearized Einstein operator around $g_b$,

$$\begin{aligned} \mathbb {L}_{g_b} = D_{g_b}({\text {Ric}}-\Lambda ) - \nabla _{g_b}\otimes \mathcal {C}_{g_b}, \end{aligned}$$

which by Lemma 3.4 is a strongly hyperbolic operator. To $\mathbb {L}_{g_b}$, we can associate the solution operator $\mathcal {S}_{g_b}(t_*)$, and the infinitesimal generator of the solution semigroup $\mathcal {A}_{g_b}$. The first result we will need is a Fredholm alternative for $\mathcal {A}_{g_b}$.

#### Theorem 5.3

For $g=g_b$ a sufficiently slowly-rotating Kerr-de Sitter background, let $\mathbb {L}_{g_b} = \mathbb {L}$ be the gauge-fixed linearized Einstein operator linearized around $g_b$. Let $(D^k(\mathcal {A}_{g_b}), \mathcal {A}_{g_b}) = (D^k(\mathcal {A}), \mathcal {A})$ be the infinitesimal generator of the solution semigroup of $\mathbb {L}_{g_b}$ on $\textbf{H}^{k}(\Sigma )$. Then, for

$$\begin{aligned} 2\Im (\sigma )> \max _{\mathcal {H}=\mathcal {H}^+,\overline{\mathcal {H}}^+} \left( \textbf{s}_{\mathbb {L}}[\mathcal {H}] - \left( 2k + \frac{1}{2} \right) \kappa _{\mathcal {H}} \right) , \end{aligned}$$

one of the following must be true:

1. $\sigma$ is in the resolvent set of $(D^k(\mathcal {A}), \mathcal {A}))$,
2. $\sigma$ is an eigenvalue of $(D^k(\mathcal {A}), \mathcal {A}))$ with finite multiplicity.

In particular, the latter possibility is true only for isolated values of $\sigma$. This implies that the resolvent is meromorphic on the specified half plane, and that the residues at the poles are finite rank operators.

#### Remark 18

When compared to the approach of proving the stability of Kerr-de Sitter in [31], and the preceding works in [26, 27, 30, 58], this theorem is equivalent to the idea of proving a *meromorphic continuation*. Also, note that like Warnick [23, 62], we construct the quasinormal modes as true eigenvalues of an operator on a Hilbert space, rather than as the poles of a cutoff resolvent.

#### Proof

See Sect. 9.1. $$\square $$

To prove asymptotic stability, we will need to locate the aforementioned eigenvalues. To this end, we first show that there are only a finite number of non-decaying $\textbf{H}^{k}$-quasinormal modes, and then we show that those finite non-decaying quasinormal modes are in some sense unphysical.

To show that there are only a finite number of non-decaying $\textbf{H}^{k}$-quasinormal modes, we prove the existence of a high-frequency spectral gap.

#### Theorem 5.4

Let $g_b$ be a slowly-rotating Kerr-de Sitter metric, and let $\mathbb {L}$, $\mathcal {A}$ denote the gauge-fixed linearized Einstein operator, and the infinitesimal generator of the solution semigroup associated to $\mathbb {L}$ respectively. Then there exists some ${\boldsymbol{\alpha }}>0$ and $C>0$ such that the resolvent $(\mathcal {A}-\sigma )^{-1}$ exists and is a uniformly bounded linear transformation of $\textbf{H}^{k}(\Sigma )$ onto $D^k(\mathcal {A})$, satisfying the bound

78 $$\begin{aligned} \left\Vert (\mathcal {A}-\sigma )^{-1}\textbf{h}_0\right\Vert _{D^k(\mathcal {A})} \lesssim \left\Vert \textbf{h}_0\right\Vert _{\textbf{H}^{k}(\Sigma )},\qquad \textbf{h}_0{:=} \begin{pmatrix} h_0\\ h_1 \end{pmatrix} \end{aligned}$$

for all $\Im \sigma \ge -{\boldsymbol{\alpha }}$, $\left\lvert \sigma \right\rvert \ge C$, $k> k_0$, where

79 $$\begin{aligned} \max _{\mathcal {H}= \mathcal {H}^+, \overline{\mathcal {H}}^+}\frac{1}{2}\textbf{s}_{L}[\mathcal {H}] + \left( \frac{1}{4} - k_0 \right) \kappa _{\mathcal {H}} < - {\boldsymbol{\alpha }}, \end{aligned}$$

#### Remark 19

Observe that in particular, using the computation of $2\textbf{s}_{\mathbb {L}}[\mathcal {H}]$ in Lemma 3.5, for a sufficiently slowly-rotating Kerr-de Sitter metric $g_b$, the choice $k_0>2$ satisfies the condition in (79). In the proof of nonlinear stability of the slowly-rotating Kerr-de Sitter family presented in [22], we will effectively take $k_0=3$ to avoid the use of fractional functional spaces.

#### Remark 20

Theorem 5.4 plays the role of Theorem 4.3 in [31]. Both statements show the existence of a high-frequency spectral gap. The main difference comes from the method of proof. We provide a self-contained proof that circumvents the use of frequency-space methods outside of a neighborhood of the trapped set.

#### Proof

See Sect. 9.2. $$\square $$

We will refer to $k_0$ as the threshold regularity level. The reliance of the spectral gap on sufficiently large $k_0$ reflects that we need the control of the resolvent $\mathcal {A}$ extends to a half-space containing the closed upper-half plane (see Sect. 9.1 for a more precise discussion).

Due to Theorem 5.4, we know that any non-decaying quasinormal mode must lie in a compact region of the complex plane. Moreover, since the eigenvalues of $(D^k(\mathcal {A}), \mathcal {A})$ are isolated, there can only be a finite number of them in any compact region of $\mathbb {C}$. Thus, Theorems 5.3 and 5.4 will together show that solutions to the gauge-fixed linearized Einstein equations decay exponentially up to a finite-dimensional perturbation, and will be proven in Sect. 9.

The results on the $\textbf{H}^{k}$-quasinormal spectrum in Theorems 5.3 and 5.4 allow us to prove an asymptotic expansion for the gauge-fixed linearized Einstein system.

#### Corollary 5.5

Fix $b\in \mathcal {B}$ a set of black-hole parameters for a slowly-rotating Kerr-de Sitter black-hole, and let $k>k_0$, where $k_0$ is the threshold regularity level defined in (79),

Let $(h_0, h_1)\in \textbf{H}^{k}$, and $f\in H^{k-1}_0(\mathcal {M})$. Then if *h* is a solution of the initial value problem

$$\begin{aligned} {\left\{ \begin{array}{ll} \mathbb {L}_{g_b}{h} & = f\\ \gamma _0({h}) & = (h_0, h_1), \end{array}\right. } \end{aligned}$$

then, there exists ${\boldsymbol{\alpha }}>0$ such that *h* has an asymptotic expansion

80 $$\begin{aligned} {h} = \tilde{h} + \sum _{j=1}^{N_{\mathbb {L}_{g_b}}}\sum _{\ell =0}^{d_j-1}e^{-\mathbbm {i}\sigma _jt_*}t_*^\ell {u}_{j\ell }(x), \end{aligned}$$

where $\tilde{h}$ satisfies the decay bounds

$$\begin{aligned} \begin{aligned} \left\Vert \tilde{h}\right\Vert _{\overline{H}^{k}(\Sigma _{t_*})}&\lesssim e^{-{\boldsymbol{\alpha }}t_*} \left\Vert (f, h_0, h_1)\right\Vert _{D^{k+1, {\boldsymbol{\alpha }}}(\mathcal {M})}. \end{aligned} \end{aligned}$$

#### Proof

Theorems 5.3 and 5.4 confirm that $\mathbb {L}_{g_b}$ is a strongly hyperbolic linear operator satisfying the assumptions in Proposition 4.12. The result then follows directly. $\square$

In the asymptotic expansion for *h* in Corollary 5.5, there are a finite number of non-decaying $\textbf{H}^{k}$-quasinormal mode solutions. Thus, to show the desired exponential decay, it remains to show that these non-decaying $\textbf{H}^{k}$-quasinormal mode solutions are unphysical.

#### Theorem 5.6

(Mode stability of $\mathbb {L}_{g_b}$, version 1) There exists a basis $\{h_\ell \}$ of the non-decaying $\textbf{H}^{k}$-quasinormal mode solution of $\mathbb {L}_{g_b}$, such that $h_{\ell }$ satisfy one of the following two conditions:

1. there exists some linearized Kerr-de Sitter metric $b'$, and some one-form $\omega$ such that
  81 $$\begin{aligned} h = g_{b}'(b') + \nabla _{g_b}\otimes \omega , \end{aligned}$$
  or;
2. *h* does not satisfy the linearized gauge constraint conditions. That is, that
  $$\begin{aligned} \mathcal {C}_{g_b}{h} \ne 0. \end{aligned}$$

As a result if *h* is a non-decaying $\textbf{H}^{k}$-quasinormal mode solution, then *h* is not a physical mode solution. These notions are expanded upon in Sect. 10, and a more precise statement of mode stability of $\mathbb {L}_{g_b}$ is given in Proposition 10.5.

#### Remark 21

At the level of mode stability, the main difference between the present work and [31] is the role of constraint damping. In [31], the authors prove linear stability using knowledge about the quasinormal modes of slowly-rotating Kerr-de Sitter, which comes from applying perturbation theory and constraint damping to the quasinormal modes of Schwarzschild-de Sitter. We instead do not introduce constraint damping, keeping more careful track of the exponentially growing modes via an identification of the non-decaying modes which comes from linearized diffeomorphisms and linearized Kerr-de Sitter metric with the non-decaying modes of the propagation equation for the harmonic coordinate condition. This will allow us to separate which exponentially growing modes arise from the general covariance of the nonlinear problem (linearized Kerr-de Sitter metrics and linearized diffeomorphisms), and which exponentially growing modes arise from violations of the linearized harmonic gauge constraint. The latter, those non-decaying quasinormal modes corresponding to violations of the linearized harmonic gauge constraints, are precisely those that one would hope to eliminate by introducing constraint damping.

### Structure of the Remainder of the Paper

We outline the remaining parts of the paper. Section 6 sets up the necessary estimates to prove Theorem 5.4, all of which are insensitive to the issue of trapping.

Section 8 set up the estimates necessary to prove Theorem 5.4. These estimates are organized so that the majority of them avoid the trapped set. The estimate taking place in a neighborhood of the trapped set is reserved for Sect. 8.4.2, which is the most technically difficult part of the paper. In particular, the estimate in Sect. 8.4.2 necessitates a detailed, frequency-space analysis of the trapped set of Kerr-de Sitter.

Section 9 then contains the proofs for the main intermediate results Theorem 5.3 and Theorem 5.4, which are direct applications of the estimates of Sects. 6 and 8.

Section 10 is of a wholly different flavor than the previous sections, dealing with the mode stability of the linearized gauge-fixed Einstein operator $\mathbb {L}_{g_b}$, and is a straightforward application of a geometric mode stability statement originally proven by Kodama and Ishibashi [37, 42], and stated in Theorem 10.1 in the form presented in [31], and the perturbation theory in Proposition 4.14.

### Choice of Constants

In what follows, the proof involves the choice of several constants. We review these constants and their relationship to each other.

1. $C_{\textbf{N}}(\epsilon )$ and $C_{\widetilde{\textbf{T}}}(\delta )$ are (large) implicit constants that are introduced in Sect. 6, chosen so that
  $$\begin{aligned} C_{\textbf{N}}(\epsilon ) \gg \max \left( 1, \frac{1}{\epsilon }\right) , \qquad C_{\widetilde{\textbf{T}}}(\delta ) \gg \max \left( 1, \frac{1}{\delta }\right) . \end{aligned}$$
2. $\gamma$ is the size of a lower-order correction term added to the $\widetilde{\textbf{T}}$-energy estimate and the redshift estimate in Sect. 6. When applied in Sect. 9.1, $\delta _0$, $\epsilon _0$ will be some fixed
  $$\begin{aligned} \delta _0, \epsilon _0 \ll 1, \end{aligned}$$
  and $\gamma$ will be chosen such that
  $$\begin{aligned} \gamma \gg C_{\textbf{N}}(\epsilon _0)C_{\widetilde{\textbf{T}}}(\delta _0). \end{aligned}$$
3. $\delta _{\mathcal {H}}$ is a smallness constant that measures the amount that we extend $\Sigma$ beyond the horizons. In Sect. 8.5, we choose $\delta _{\mathcal {H}}$ such that
  $$\begin{aligned} \delta _{\mathcal {H}}^2\ll \max \left( \frac{r_0}{r_{\mathcal {H}^+}}, \frac{r_{\overline{\mathcal {H}}^+}}{R_0} \right) . \end{aligned}$$
4. $\delta _r$ and $\delta _\zeta$ are smallness constants measuring the size of the localization around the trapped set we take in Sect. 8.
5. $\varepsilon _{{\Gamma }}$ is a smallness constant that measures the smallness of the skew-adjoint component of the subprincipal symbol at the trapped set, and we will take
  $$\begin{aligned} \varepsilon _{{\Gamma }} \ll 1 \end{aligned}$$
  in Sect. 8.4.
6. $\textbf{M}$ will denote the maximal exponential growth rate of solutions to the linearized Einstein equations.
7. $\breve{C}$ and $\check{C}$ are large constants that produces large bulk terms in the Morawetz estimates in Sects. 8.2 and 8.3 which will be chosen so that
  $$\begin{aligned} \breve{C}\gg \frac{1}{\delta _r^2}, \qquad \check{C}\gg \frac{1}{\delta _\zeta }. \end{aligned}$$
8. We will use $C_0$ to denote the large lower-order error that is incurred in all of our high-frequency estimates. It is the largest constant in the proofs for high-frequency resolvent estimates, and is chosen so that
  $$\begin{aligned} \textbf{M}\ll \breve{C}, \check{C}\ll C_0. \end{aligned}$$
9. $\mathring{c}$, $\check{c}$, and $\breve{c}$ are constants introduced in Sect. 8.5 to help glue the Morawetz estimates on the various regions of phase space together. For a more precise description of their relationship with the other constants here, see Sect. 8.5.
10. $b=(M,a)$ and $b_0 = (M_0,0)$ denote the Kerr-de Sitter and Schwarzschild-de Sitter black hole parameters we will consider. We treat $\Lambda$ as a fixed constant, and *M* satisfying the mass-subextremal condition in (2), and *a* such that
  $$\begin{aligned} 0\le a \ll \min \left( \varepsilon _{\textbf{N}}, \delta _r, \delta _\zeta , \delta _{\mathcal {H}}, \frac{\epsilon _0}{C_{\textbf{N}}(\epsilon _0)C_{\widetilde{\textbf{T}}}(\delta _0)}, \frac{r_0}{r_{\mathcal {H}^+}} - 1, \frac{r_{\overline{\mathcal {H}}^+}}{R_0} - 1 \right) . \end{aligned}$$

## Energy Estimates

In this section, we prove a variety of energy estimates that will be used throughout the paper. In particular, we will prove a Killing energy estimate, a redshift energy estimate, and an enhanced redshift energy estimate. The Morawetz estimate, or its resolvent estimate equivalent, is the subject of Sect. 8, due to the different approach and difficulties involved in the proof. All the estimates in this section are proven relying purely on the physical space vectorfield method, using vectorfields as multipliers and commutators.

We prove the estimates in this section for any strongly hyperbolic linear operator on a slowly-rotating Kerr-de Sitter background of the form

82 $$\begin{aligned} L{h} = \Box _{g_{b}} {h} + \textbf{S}[{h}] + \textbf{V}{h}, \end{aligned}$$

where $\textbf{S}$ is a matrix-valued vectorfield, and $\textbf{V}$ is a smooth matrix valued potential. Recall from Lemma 3.4 that the gauge-fixed linearized Einstein operator is itself a strongly hyperbolic linear operator. As we are only working with a general strongly hyperbolic linear operator, we do not need any additional structural assumptions from Einstein’s equations. In particular, the estimates proven in this section are blind to the presence of the trapped set. This should be contrasted with the derivations of the Morawetz estimates in Sect. 8, where trapping plays a crucial role and we require a precise structure in Einstein’s equations at the trapped set to close the argument.

### $\widetilde{\textbf{T}}$-energy Estimates

The Killing estimate will be derived via using the almost Killing vectorfield $\widetilde{\textbf{T}}$ of Lemma 2.9 as a multiplier. We define the $\widetilde{\textbf{T}}$-energy by

83 $$\begin{aligned} E(t_*)[{h}] = \int _{\widetilde{\Sigma }_{t_*}} J^{\widetilde{\textbf{T}}, 0, 0} [{h}]\cdot n_{\Sigma }, \end{aligned}$$

where $J^{X,q,m}[h]$ is as defined in (23), with $(X,q,m) = (\widetilde{\textbf{T}}, 0, 0)$, and we recall the definition of $\widetilde{\Sigma }$ in (4).

#### Theorem 6.1

We have the following estimates.

1. There exists a $C>0$ such that
  84 $$\begin{aligned} E(t_*)[{h}] \lesssim \left\Vert \nabla {h}\right\Vert _{L^2(\widetilde{\Sigma }_{t_*})}^2 \end{aligned}$$
  for all $${h}\in C^\infty (\mathcal {M},\mathbb {C}^N)$$.
2. For any vectorfield *X* tangent to both $\overline{\mathcal {H}}^+$ and $\mathcal {H}^+$, there exists $C_X$ depending on *X* such that for all *h*,
  85 $$\begin{aligned} \left\Vert X{h}\right\Vert ^2_{L^2(\widetilde{\Sigma }_{t_*})} \le C_X E(t_*)[{h}]. \end{aligned}$$
3. For any $\epsilon >0$, there exists a constant $C(\epsilon )$ such that
  86 $$\begin{aligned} \begin{aligned} \partial _{t_*} E(t_*)[{h}] \le {}&\epsilon \left( \left\Vert {h}\right\Vert ^2_{\underline{H}^{1}(\widetilde{\Sigma }_{t_*})} + \left\Vert L{h}\right\Vert ^2_{\underline{L}^2(\widetilde{\Sigma }_{t_*})} \right) \\&+ C(\epsilon )\left\Vert \textbf{T}{h}\right\Vert _{\underline{L}^2(\widetilde{\Sigma }_{t_*})}^2 + a C(\epsilon ) \left\Vert h\right\Vert ^2_{\underline{H}^{1}(\widetilde{\Sigma }_{t_*})}. \end{aligned} \end{aligned}$$
4. If *h* is additionally assumed to vanish on the horizons, there exists a constant $C(\epsilon )$ independent of $\gamma$ such that for any $\epsilon >0$, the following estimate holds
  87 $$\begin{aligned} \begin{aligned} -\partial _{t_*} E(t_*)[{h}] \le {}&\epsilon \left( \left\Vert {h}\right\Vert ^2_{\underline{H}^{1}(\widetilde{\Sigma }_{t_*})} + \left\Vert L{h}\right\Vert ^2_{\underline{L}^2(\widetilde{\Sigma }_{t_*})} \right) \\&+ C(\epsilon )\left\Vert \textbf{T}{h}\right\Vert _{\underline{L}^2(\widetilde{\Sigma }_{t_*})}^2 + a C(\epsilon ) \left\Vert h\right\Vert ^2_{\underline{H}^{1}(\widetilde{\Sigma }_{t_*})}. \end{aligned} \end{aligned}$$

#### Remark 22

Upon initial inspection, it appears alarming that $\left\Vert \textbf{T}h\right\Vert _{\underline{L}^2(\Sigma )}$ terms show up on the right-hand side of the estimates in Theorem 6.1, especially since $C(\epsilon )$ is not limited to being a small constant. In fact, it could be very large! Thankfully, the estimates in Theorem 6.1 are not used to prove decay. Rather, they will only be used to prove that the resolvent $\widehat{L}(\sigma )^{-1}$ is bounded on some upper-half space, i.e. that solutions to $Lh = 0$ have a maximum exponential rate at which they grow. In these settings, the $\left\Vert \textbf{T}h\right\Vert _{\underline{L}^2(\Sigma )}$ term can generally be absorbed (via some Gronwall estimate with a large exponent).

#### Proof

To prove 1 and 2, it is sufficient to observe that $\widetilde{\textbf{T}}$ by definition is time-like in $\mathcal {M}^\circ$ as defined in (3), and null along both the event horizon and the cosmological horizon.

To establish 3, we apply the divergence theorem of Corollary 2.6 on $\Sigma _{t_*}$ with the vectorfield $J^{\widetilde{\textbf{T}}, 0, 0}[{h}]$,

88 $$\begin{aligned} \begin{aligned} - \int _{\widetilde{\Sigma }_{t_*}}\nabla \cdot J^{\widetilde{\textbf{T}}, 0, 0}[{h}]\, \sqrt{A} ={}&\partial _{t_*}E(t_*)[h] + \int _{\overline{\mathcal {H}}^+\bigcap \widetilde{\Sigma }_{t_*}} J^{\widetilde{\textbf{T}}, 0, 0}[{h}] \cdot \textbf{K}_{\overline{\mathcal {H}}^+}\\&+\int _{\mathcal {H}^+\bigcap \widetilde{\Sigma }_{t_*}} J^{\widetilde{\textbf{T}}, 0, 0}[{h}] \cdot \textbf{K}_{\mathcal {H}^+}, \end{aligned} \end{aligned}$$

and handle the terms individually. The first term on the right hand side is left alone as it gives rise to the derivative of the energy. Consider surface integrals at the horizons. Since $\widetilde{\textbf{T}}$ is null, future-directed, and tangent to both $\mathcal {H}^+$ and $\overline{\mathcal {H}}^+$, the surface integrals at the horizons are positive. That is,

$$\begin{aligned} \int _{\mathcal {H}^+\bigcap \widetilde{\Sigma }_{t_*}}J^{\widetilde{\textbf{T}}, 0, 0}[{h}]\cdot \textbf{K}_{\mathcal {H}^+} \ge 0,\qquad \int _{\overline{\mathcal {H}}^+\bigcap \widetilde{\Sigma }_{t_*}} J^{\widetilde{\textbf{T}}, 0, 0}[{h}]\cdot \textbf{K}_{\overline{\mathcal {H}}^+} \ge 0. \end{aligned}$$

We now deal with the divergence term on the left-hand side of (88). Using (23) and the divergence property of the energy-momentum tensor in (24), we have that

$$\begin{aligned} \nabla \cdot J^{\widetilde{\textbf{T}}, 0, 0}[{h}]&= \Re \left[ \widetilde{\textbf{T}}\overline{{h}}\cdot \Box _{g}{h} \right] + K^{\widetilde{\textbf{T}}, 0, 0}[{h}]\\&= \Re \left[ \widetilde{\textbf{T}}\overline{{h}}\cdot \left( L{h} -\textbf{S}[{h}] -\textbf{V}[h] \right) \right] + K^{\widetilde{\textbf{T}}, 0, 0}[{h}]. \end{aligned}$$

We first consider

89 $$\begin{aligned} \Re \left[ \widetilde{\textbf{T}}\overline{h} \cdot \left( L{h} -\textbf{S}[{h}] -\textbf{V}[h] \right) \right] . \end{aligned}$$

These terms can each be directly controlled by Cauchy-Schwarz, using the fact that $\widetilde{\textbf{T}}= \textbf{T}+ a\,\tilde{\chi }(r){\Phi }$,

$$\begin{aligned} \begin{aligned}&\int _{\widetilde{\Sigma }_{t_*}} \Re \left[ \widetilde{\textbf{T}}\overline{{h}}\cdot \left( L{h} -\textbf{S}[{h}] -\textbf{V}[h] \right) \right] \,\sqrt{A}\\ \le {}&\epsilon \left( \left\Vert {h}\right\Vert ^2_{\underline{H}^{1}(\widetilde{\Sigma }_{t_*})} + \left\Vert L{h}\right\Vert ^2_{\underline{L}^2(\widetilde{\Sigma }_{t_*})} \right) + C(\epsilon )\left\Vert \textbf{T}{h}\right\Vert _{\underline{L}^2(\widetilde{\Sigma }_{t_*})}^2 + a C(\epsilon ) \left\Vert h\right\Vert ^2_{\underline{H}^{1}(\widetilde{\Sigma }_{t_*})}. \end{aligned} \end{aligned}$$

We now consider the deformation tensor term. Using Eq. (34), we see that

90 $$\begin{aligned} \int _{\widetilde{\Sigma }_{t_*}}K^{\widetilde{\textbf{T}}, 0, 0}[h]\,\sqrt{A} \lesssim {} a\left\Vert {h}\right\Vert ^2_{\underline{H}^{1}(\widetilde{\Sigma }_{t_*})} + a \left\Vert \textbf{T}h\right\Vert ^2_{\underline{L}^2(\widetilde{\Sigma }_{t_*})}. \end{aligned}$$

We conclude the proof of 3 by taking $C(\epsilon )$ sufficiently large.

The final statement, 4, is proven in the same way as 3. Since we have the additional assumption that *h* vanishes on $\mathcal {H}^+$ as well as $\overline{\mathcal {H}}^+$, we can neglect the surface integrals at the horizons, and only need to estimate the divergence term. This can be done using Cauchy-Schwarz in the same manner as (90). $\square$

The presence of *O*(*a*) terms on the right hand side of our estimates arise from the issue of superradiance. Since we are working in the slowly rotating case, these superradiant terms will be handled by an appropriate redshift argument, which is the subject of Theorem 6.3.

We also prove the following corollary of Theorem 6.1 which will be useful in Sect. 9 for proving Theorem 5.3.

#### Corollary 6.2

Define the energy

91 $$\begin{aligned} E_\gamma (t_*)[{h}] = \int _{\widetilde{\Sigma }_{t_*}} J^{\widetilde{\textbf{T}}, 0, -\gamma \widetilde{\textbf{T}}^\flat } [{h}]\cdot n_{\widetilde{\Sigma }_{t_*}}, \end{aligned}$$

where we recall the definition of $J^{X,q,m}[h]$ from (23).

We have the following estimates.

1. For all $${h}\in C^\infty (\mathcal {M},\mathbb {C}^D)$$,
  92 $$\begin{aligned} E_\gamma (t_*)[{h}] \lesssim \left\Vert h\right\Vert _{H^1(\widetilde{\Sigma }_{t_*})}^2 + \left\Vert \textbf{T}h\right\Vert _{L^2(\widetilde{\Sigma }_{t_*})}^2 + \gamma \left\Vert h\right\Vert _{L^2(\widetilde{\Sigma }_{t_*})}^2. \end{aligned}$$
2. For any $\epsilon >0$, there exists a constant $C(\epsilon )$ independent of $\gamma$, and a constant *C* independent of both $\epsilon$ and $\gamma$ such that
  93 $$\begin{aligned} \begin{aligned} \partial _{t_*} E_{\gamma }(t_*)[{h}] \le {}&\epsilon \left( \left\Vert {h}\right\Vert ^2_{\underline{H}^{1}(\widetilde{\Sigma }_{t_*})} + \left\Vert (L- \gamma ){h}\right\Vert ^2_{\underline{L}^2(\widetilde{\Sigma }_{t_*})} \right) \\&+ C(\epsilon )\left\Vert \textbf{T}{h}\right\Vert _{\underline{L}^2(\widetilde{\Sigma }_{t_*})}^2 + a C(\epsilon ) \left\Vert h\right\Vert _{\underline{H}^{1}(\widetilde{\Sigma }_{t_*})}^2\\&+ aC \gamma \left\Vert h\right\Vert ^2_{\underline{L}^2(\Sigma _{t_*})}. \end{aligned} \end{aligned}$$
3. If *h* is additionally assumed to vanish on the horizon, there exists a constant $C(\epsilon )$ independent of $\gamma$, and a constant *C* independent of both $\gamma$ and $\epsilon$ such that for any $\epsilon >0$, the following estimate holds
  94 $$\begin{aligned} \begin{aligned} -\partial _{t_*} E_\gamma (t_*)[{h}] \le {}&\epsilon \left( \left\Vert {h}\right\Vert ^2_{\underline{H}^{1}(\widetilde{\Sigma }_{t_*})} + \left\Vert (L- \gamma ){h}\right\Vert ^2_{\underline{L}^2(\widetilde{\Sigma }_{t_*})} \right) \\&+ C(\epsilon )\left\Vert \textbf{T}{h}\right\Vert _{\underline{L}^2(\widetilde{\Sigma }_{t_*})}^2 + a C(\epsilon ) \left\Vert h\right\Vert _{\underline{H}^{1}(\widetilde{\Sigma }_{t_*})}^2\\&+ a C\gamma \left\Vert h\right\Vert ^2_{\underline{L}^2(\Sigma _{t_*})}. \end{aligned} \end{aligned}$$

#### Proof

The first conclusion, follows directly from (84) and the definition of $J^{\widetilde{\textbf{T}}, 0, -\gamma \widetilde{\textbf{T}}^{\flat }}$[*h*].

To prove (93), observe that for $\mathcal {H}=\mathcal {H}^+,\overline{\mathcal {H}}^+$,

$$\begin{aligned} \int _{\mathcal {H}\bigcap \widetilde{\Sigma }_{t_*}} J^{\widetilde{\textbf{T}}, 0, -\gamma \widetilde{\textbf{T}}^\flat }[h]\cdot \textbf{K}_{\mathcal {H}} = \int _{\mathcal {H}\bigcap \widetilde{\Sigma }_{t_*}} J^{\widetilde{\textbf{T}}, 0, 0}[h]\cdot \textbf{K}_{\mathcal {H}} . \end{aligned}$$

Moreover, for $\gamma >0$, the flux terms vanish, since for $\mathcal {H}= \mathcal {H}^+, \overline{\mathcal {H}}^+$,

$$\begin{aligned} \int _{\mathcal {H}\bigcap \widetilde{\Sigma }_{t_*}} \gamma \left\lvert h\right\rvert ^2g\left( \textbf{K}_{\mathcal {H}}, \textbf{K}_{\mathcal {H}}\right) = 0. \end{aligned}$$

As a result, following the proof of (86), we see that we again have

$$\begin{aligned} \partial _{t_*}E(t_*)[h] \le \left\lvert \int _{\widetilde{\Sigma }_{t_*}}\nabla \cdot J^{\widetilde{\textbf{T}}, 0, -\gamma \widetilde{\textbf{T}}^\flat }[h]\sqrt{A}\right\rvert . \end{aligned}$$

Using the divergence property (24), we have that

$$\begin{aligned} \nabla \cdot J^{\widetilde{\textbf{T}}, 0, -\gamma \widetilde{\textbf{T}}^\flat }[h]\!=\!\Re \left[ \widetilde{\textbf{T}}\overline{h}\cdot \left( (L\!-\!\gamma )h\!-\! \textbf{S}[h]\! -\! \textbf{V}[h]\right) \right] \!+\! K^{\widetilde{\textbf{T}}, 0,\! -\!\gamma \widetilde{\textbf{T}}^\flat }[h] \!+\! \Re \left[ \widetilde{\textbf{T}}\overline{h}\cdot \gamma h\right] , \end{aligned}$$

where we now have using the construction of $\widetilde{\textbf{T}}$ in Lemma 2.9 that for some $C>0$ independent of $\gamma$,

$$\begin{aligned} \begin{aligned}&\left\lvert \int _{\widetilde{\Sigma }_{t_*}}K^{\widetilde{\textbf{T}}, 0, -\gamma \widetilde{\textbf{T}}^\flat }[h]\,\sqrt{A} + \Re \left\langle \widetilde{\textbf{T}}h, \gamma h\right\rangle _{\underline{L}^2(\widetilde{\Sigma }_{t_*})}\right\rvert \\ \le {}&C\left( a\left\Vert h\right\Vert _{\underline{H}^{1}(\widetilde{\Sigma }_{t_*})}^2 + \left\Vert \textbf{T}h\right\Vert _{\underline{L}^2(\widetilde{\Sigma }_{t_*})}^2 + a\gamma \left\Vert h\right\Vert _{\underline{L}^2(\widetilde{\Sigma }_{t_*})}^2\right) . \end{aligned} \end{aligned}$$

Repeating the proof of (86) as in Theorem 6.1 yields the conclusions of the corollary. The proof of (87) follow similarly. $\square$

### Redshift Estimates

The redshift estimates will allow us to extend energy estimates to the horizons. This will be useful in proving both the meromorphic continuation and the resolvent estimates. We will use the vectorfield $\textbf{N}$ as a multiplier to derive the redshift estimates. We define the redshift energy on $\Sigma _{t_*}$,

95 $$\begin{aligned} \mathcal {E}(t_*)[{h}] = \int _{\Sigma _{t_*}}J^{\textbf{N}, 0, 0}[{h}]\cdot n_{\Sigma _{t_*}}, \end{aligned}$$

and the redshift region

96 $$\begin{aligned} \dot{\Sigma }{:=}[r_{\mathcal {H}^+}-\varepsilon _{\mathcal {M}}, r_0)\bigcup (R_0, r_{\overline{\mathcal {H}}^+}+\varepsilon _{\mathcal {M}}]. \end{aligned}$$

#### Theorem 6.3

Let $\varepsilon _{\textbf{N}}>0$ be fixed.

1. We have that
  97 $$\begin{aligned} \mathcal {E}(t_*)[{h}] \lesssim \left\Vert \nabla {h}\right\Vert ^2_{L^2(\Sigma _{t_*})} \lesssim \mathcal {E}(t_*)[{h}]. \end{aligned}$$
2. For any $\epsilon >0$, there exists some constant $C_{\textbf{N}}(\epsilon )>0$ such that
  98 $$\begin{aligned} \partial _{t_*} \mathcal {E}(t_*)[{h}] \le {}&\max _{\mathcal {H}=\mathcal {H}^+, \overline{\mathcal {H}}^+}\left( \textbf{s}_{L}[\mathcal {H}]-\kappa _{\mathcal {H}} +\varepsilon _{\textbf{N}} + \epsilon \right) \mathcal {E}(t_*)[{h}] \nonumber \\&+ C_{\textbf{N}}(\epsilon )\left( \left\Vert L{ h}\right\Vert _{\underline{L}^2(\Sigma _{t_*})}^2 + E(t_*)[{h}] + \left\Vert h\right\Vert _{\underline{L}^2(\Sigma _{t_*})}^2 \right) . \end{aligned}$$
3. If in addition, *h* is assumed to vanish on the event horizon and the cosmological horizon, then for any $\epsilon >0$, there exists $C(\epsilon )$ such that
  99 $$\begin{aligned} -\partial _{t_*} \mathcal {E}(t_*)[{h}] \le {}&\max _{\mathcal {H}=\mathcal {H}^+, \overline{\mathcal {H}}^+}\left( -\textbf{s}_{L}^*[\mathcal {H}] + \kappa _{\mathcal {H}} + \varepsilon _{\textbf{N}} +\epsilon \right) \mathcal {E}(t_*)[{h}]\nonumber \\&+C_{\textbf{N}}\left( \left\Vert L{h}\right\Vert _{\underline{L}^2(\Sigma _{t_*})}^2 + E(t_*)[\chi _\bullet h] + \left\Vert h\right\Vert _{\underline{L}^2(\Sigma _{t_*})}^2 \right) , \end{aligned}$$
  for some $\chi _\bullet (r)$ such that
  $$\begin{aligned} \chi _\bullet (r)= {\left\{ \begin{array}{ll} 0& r< \frac{1}{2}\left( r_{\mathcal {H}^+}+ r_0\right) ,\\ 1&  r>r_0. \end{array}\right. } \end{aligned}$$
4. If instead, *h* is supported on $\dot{\Sigma }$ as defined in (96), then for any $\epsilon >0$, there exists $C(\epsilon )$ such that
  100 $$\begin{aligned} \begin{aligned} \partial _{t_*}\mathcal {E}(t_*)[{h}] \le {}&\max _{\mathcal {H}=\mathcal {H}^+, \overline{\mathcal {H}}^+}\left( \textbf{s}_{L}[\mathcal {H}] - \kappa _{\mathcal {H}} + \varepsilon _{\textbf{N}} + \epsilon \right) \mathcal {E}(t_*)[{h}]\\&+ C(\epsilon )\left\Vert L{h}\right\Vert _{\underline{L}^2(\Sigma _{t_*})}^2 + C(\epsilon )\left\Vert h\right\Vert _{\underline{L}^2(\Sigma _{t_*})}^2. \end{aligned} \end{aligned}$$

#### Proof

Using the fact that

$$\begin{aligned} g\left( \frac{n_{\Sigma }}{\sqrt{A}}, \textbf{K}\right) = -1, \end{aligned}$$

we decompose $\textbf{S}[h]$ into

101 $$\begin{aligned} \textbf{S}= \frac{1}{\sqrt{A}}s n_{\Sigma } + \textbf{S}', \end{aligned}$$

where $\textbf{S}'$ is tangent to the horizons, and $n_{\Sigma }$ denotes the unit normal to $\Sigma$. We now fix $\textbf{N}$ to be as constructed in Proposition 2.7 with $X=\textbf{S}'$ and $\varepsilon _{\textbf{N}}$.

To prove 1, a similar argument as in the case of the $\widetilde{\textbf{T}}$-energy shows that the redshift energy $\mathcal {E}(t_*)[{h}]$ is positive. Then, since $\textbf{N}$ is everywhere timelike, $\mathcal {E}(t_*)[{h}]$ controls all derivatives, including those transverse to the horizons.

To prove 2, we apply the divergence theorem in Corollary 2.6 with $X=J^{\textbf{N}, 0, 0}[{h}]$,

102 $$\begin{aligned}&\partial _{t_*}\int _{\Sigma _{t_*}}J^{\textbf{N}, 0, 0}[h]\cdot n_{\Sigma _{t_*}} + \int _{\mathcal {H}^+_-\bigcap \Sigma _{t_*}}J^{\textbf{N}, 0, 0}[{h}]\cdot n_{\mathcal {H}^+_-} + \int _{\overline{\mathcal {H}}^+_+\bigcap \Sigma _{t_*}}J^{\textbf{N}, 0, 0}[{h}]\cdot n_{\overline{\mathcal {H}}^+_+}\nonumber \\ ={}&- \int _{\Sigma _{t_*}}\nabla \cdot J^{\textbf{N}, 0, 0}[{h}]\sqrt{A}. \end{aligned}$$

By construction, $\textbf{N}$ is timelike and future-directed everywhere. As a result,

$$\begin{aligned} \int _{\mathcal {H}^+_-}J^{\textbf{N}, 0, 0}[{h}]\cdot n_{\mathcal {H}^+_-} \ge 0, \quad \int _{\overline{\mathcal {H}}^+_+}J^{\textbf{N}, 0, 0}[{h}]\cdot n_{\overline{\mathcal {H}}^+_+}\ge 0. \end{aligned}$$

It thus remains to estimate the divergence term on the right-hand side of (102). Using (22),

103 $$\begin{aligned} \nabla \cdot J^{\textbf{N}, 0, 0}[{h}] = \Re \left[ \textbf{N}\overline{{h}}\cdot \Box _{g}{h} \right] +K^{\textbf{N}, 0, 0}[h]. \end{aligned}$$

We consider the terms in (103) individually. First, using the definition of $L$,

$$\begin{aligned} \begin{aligned} \Re \left[ \textbf{N}\overline{{h}}\cdot \Box _{g} {h}\right]&= \Re \left[ \textbf{N}\overline{{h}}\cdot \left( L{h}-\textbf{S}[{h}]- \textbf{V}{h}\right) \right] . \end{aligned} \end{aligned}$$

Then, using Cauchy-Schwartz and (97),

104 $$\begin{aligned} \left\langle \textbf{N}h, Lh - \textbf{V}h \right\rangle _{\underline{L}^2(\Sigma )} \le {} \epsilon \mathcal {E}(t_*)[{h}] + C(\epsilon )\left( \left\Vert L{h}\right\Vert _{\underline{L}^2(\Sigma )}^2 + \left\Vert h\right\Vert _{\underline{L}^2(\Sigma )}^2 \right) . \end{aligned}$$

We now control the principal bulk term, $K^{\textbf{N},0,0}[{h}]-\Re \left[ \textbf{N}\overline{{h}}\cdot \textbf{S}[{h}]\right]$. Observe that

$$\begin{aligned} \textbf{s}_{L}^*[\mathcal {H}]\left\lvert \xi \right\rvert ^2 \le \left. \Re [\xi ^*\cdot s\xi ]\right| _{\mathcal {H}} \le \textbf{s}_{L}[\mathcal {H}]\left\lvert \xi \right\rvert ^2, \end{aligned}$$

where *s* is as defined in (101). Then, from (28), we have that

$$\begin{aligned} \Re \left\langle \textbf{N}h, \textbf{S}[h]\right\rangle _{\underline{L}^2(\dot{\Sigma })} \!\!-\! \int _{\dot{\Sigma }} K^{\textbf{N}, 0, 0}[h]\,\sqrt{A} \!\le \! \max _{\mathcal {H}=\mathcal {H}^+, \overline{\mathcal {H}}^+}\left( \textbf{s}_{L}[\mathcal {H}] \!-\! \kappa _{\mathcal {H}}\! +\! \varepsilon _{\textbf{N}} \right) \int _{\dot{\Sigma }}J^{\textbf{N},0, 0}[h]\cdot n_{\dot{\Sigma }}, \end{aligned}$$

where we recall the definition of $\dot{\Sigma }$ in (96), and we have used the fact that the redshift bulk $K^{\textbf{N}, 0, 0}[h]$ controls a sufficiently large amount of the derivatives tangential to the horizons. It remains then to control $K^{\textbf{N},0,0}[{h}]-\Re \left[ \textbf{N}\overline{{h}}\cdot \textbf{S}[{h}]\right]$ on $\Sigma \backslash \dot{\Sigma }$. But on $\Sigma \backslash \dot{\Sigma }$, we recall from (85) that $E(t_*)[h]$ controls the full $L^2$ norm of $\nabla h$. Thus, we have that for some $C>0$,

105 $$\begin{aligned} \begin{aligned}&\Re \left\langle \textbf{N}h, \textbf{S}[h]\right\rangle _{\underline{L}^2(\Sigma )} - \int _{\Sigma } K^{\textbf{N}, 0, 0}[h]\,\sqrt{A}\\ \le {}&\max _{\mathcal {H}=\mathcal {H}^+, \overline{\mathcal {H}}^+}\left( \textbf{s}_{L}[\mathcal {H}] - \kappa _{\mathcal {H}} + \varepsilon _{\textbf{N}} \right) \mathcal {E}(t_*)[{h}] + CE(t_*)[{h}]. \end{aligned} \end{aligned}$$

Combining the estimates in (104) and (105) and taking $C(\epsilon )$ sufficiently large yields the conclusion.

To prove 3, we repeat the same estimates but using $-\textbf{N}$ as a multiplier. The only change that we need to make to the argument is the analysis on the boundary flux terms, which are no longer positive. However, this is easily handled since we assumed that *h* vanishes exactly at $\mathcal {H}^+_-$ and $\overline{\mathcal {H}}^+_+$.

Finally, we prove 4. This can be done repeating the proof above, but on $\dot{\Sigma }$ instead of $\Sigma$. We then observe that the only place we needed to use $E(t_*)[{h}]$ to control derivatives was on $\Sigma \backslash \dot{\Sigma }$. On $\dot{\Sigma }$, we can use (28) to achieve arbitrary control of tangential derivatives. $\square$

An important first corollary of the redshift energy estimates is an application of Gronwall’s Lemma:

#### Corollary 6.4

Fix ${T_*}>0$ and let $\psi \in L^2(\mathbb {R}^+, H^{1}(\Sigma ))$ with $\textbf{T}\psi \in L^2(\mathbb {R}^+, L^2(\Sigma ))$ be a weak solution to the Cauchy problem

106 $$\begin{aligned} \begin{aligned} L\psi&= f,\\ \gamma _0(\psi )&=(\psi _0,\psi _1) \end{aligned} \end{aligned}$$

where $L$ is the gauge-fixed linearized Einstein operator above, $\psi _0\in H^{1}(\Sigma ), \psi _1\in L^2(\Sigma )$. Then $\psi \in C^0(\mathbb {R}^+,H^{1}(\Sigma ))$ with $\textbf{T}\psi \in C^0(\mathbb {R}^+, L^2(\Sigma ))$, and there exists some constant $\textbf{M}$, depending on the black-hole background *g*, such that

107 $$\begin{aligned} \sup _{t_*\le {T_*}}e^{-\textbf{M}t_*}\left\Vert \psi \right\Vert _{H^{1}(\Sigma _{t_*})} \lesssim \left( \left\Vert \psi _0\right\Vert _{H^{1}(\Sigma )} + \left\Vert \psi _1\right\Vert _{L^2(\Sigma )} + \int _0^{T_*}e^{-\textbf{M}t_*}\left\Vert f\right\Vert _{\underline{L}^2(\Sigma _{t_*})}\,dt_*\right) . \end{aligned}$$

In particular, there exists some $\textbf{M}$ such that for

$$\Im \sigma > \textbf{M}$$,

108 $$\begin{aligned} \left\Vert u\right\Vert _{\underline{H}^{1}_\sigma (\Sigma )} \lesssim \left\Vert \widehat{L}(\sigma )u\right\Vert _{\underline{L}^2(\Sigma )}. \end{aligned}$$

#### Proof

To prove the first statement, suppose $\psi _0,\psi _1$ are in fact smooth, and induce a smooth solution of (106). Then for $\gamma$ fixed and sufficiently large, there exists some $\textbf{M}$ sufficiently large such that

$$\begin{aligned} \begin{aligned}&\max _{\mathcal {H}=\mathcal {H}^+, \overline{\mathcal {H}}^+}\left( \textbf{s}_{L}[\mathcal {H}]-\kappa _{\mathcal {H}} +\varepsilon _{\textbf{N}} + \epsilon \right) \mathcal {E}(t_*)[{h}] \\&+ C_{\textbf{N}}(\epsilon )\left( \left\Vert L{ h}\right\Vert _{\underline{L}^2(\Sigma _{t_*})}^2 + E(t_*)[{h}] + \left\Vert h\right\Vert _{\underline{L}^2(\Sigma _{t_*})}^2 \right) \\ \le {}&\textbf{M}\mathcal {E}_{\gamma }(t_*)[h] + C_{\textbf{N}}(\epsilon )\left\Vert L{ h}\right\Vert _{\underline{L}^2(\Sigma _{t_*})}^2. \end{aligned} \end{aligned}$$

Thus, using (98), we have that

109 $$\begin{aligned} \partial _{t_*} \mathcal {E}(t_*)[{h}]\le {}\textbf{M}\mathcal {E}_{\gamma }(t_*)[h] + C_{\textbf{N}}(\epsilon )\left\Vert L{ h}\right\Vert _{\underline{L}^2(\Sigma _{t_*})}^2. \end{aligned}$$

An application of Gronwall’s inequality then immediately yields (107). An approximation argument then allows us to loosen the smoothness requirement since $C^0(\mathbb {R}^+,H^{1}(\Sigma ))$ and $C^0(\mathbb {R}^+,L^2(\Sigma ))$ are complete.

To prove the Laplace-transformed statement in (108), we rewrite (109),

110 $$\begin{aligned} \partial _{t_*}\mathcal {E}(t_*)[h] - \textbf{M}\mathcal {E}(t_*)[h] \lesssim \left\Vert L{h}\right\Vert _{\underline{L}^2(\mathcal {D})}^2. \end{aligned}$$

Considering (110) in the specific case where $h(t_*, x) = e^{-\mathbbm {i}\sigma t_*} u(x)$, we have that

$$\begin{aligned} \left( \Im \sigma - \textbf{M}\right) \mathcal {E}(t_*)[e^{-\mathbbm {i}\sigma t_*}u] \lesssim C \left\Vert Le^{-\mathbbm {i}\sigma t_*}u\right\Vert _{\underline{L}^2(\Sigma _{t_*})}. \end{aligned}$$

For $\Im \sigma >\textbf{M}$, we see that the left-hand side is positive. Multiplying both sides by $e^{-2\Im \sigma t_*}$ to remove any $t_*$-dependency, we then have that

$$\begin{aligned} \left( \Im \sigma - \textbf{M}\right) \left( \left\Vert u\right\Vert _{H^1(\Sigma _{t_*})} + \left\Vert \sigma u\right\Vert _{L^2(\Sigma _{t_*})} \right) \lesssim \left\Vert \widehat{L}(\sigma )u\right\Vert _{\underline{L}^2(\Sigma _{t_*})} \end{aligned}$$

as desired. $$\square $$

#### Remark 23

We remark that the second corollary above is exactly the statement that the resolvent of the operator $L$ is bounded in some upper-half space. Indeed, any estimate of the form

$$\begin{aligned} \partial _{t_*}E[\psi ] \lesssim E[\psi ] + \left\Vert L\psi \right\Vert ^2, \end{aligned}$$

for a coercive energy *E* would give a similar quasinormal mode-free region. The redshift energy used in the corollary is just one such example.

#### Corollary 6.5

Fix some $\varepsilon _{\textbf{N}}>0$, and define

111 $$\begin{aligned} \mathcal {E}_\gamma (t_*)[h] = \int _{\Sigma _{t_*}}J^{\textbf{N},0, -\gamma \textbf{N}^\flat }[h]\cdot n_{\Sigma _{t_*}}, \end{aligned}$$

where we recall the definition of $J^{X,q,m}[h]$ in (24). Then we have the following estimates.

1. We have that
  112 $$\begin{aligned} \mathcal {E}_\gamma (t_*)[{h}] \lesssim \left\Vert h\right\Vert ^2_{H^1(\Sigma _{t_*})} + \left\Vert \textbf{T}h\right\Vert _{L^2(\Sigma _{t_*})}^2 + \gamma \left\Vert h\right\Vert ^2_{L^2(\Sigma _{t_*})} \lesssim \mathcal {E}_\gamma (t_*)[{h}]. \end{aligned}$$
2. Then for any $\epsilon >0$, there exists some constant $C_{\textbf{N}}(\epsilon )>0$ such that
  113 $$\begin{aligned} \partial _{t_*} \mathcal {E}_\gamma (t_*)[{h}] \le {}&\max _{\mathcal {H}=\mathcal {H}^+, \overline{\mathcal {H}}^+}\left( \textbf{s}_{L}[\mathcal {H}] -\kappa _{\mathcal {H}} +\varepsilon _{\textbf{N}} +\epsilon \right) \mathcal {E}_\gamma (t_*)[{h}] \nonumber \\&+ C_{\textbf{N}}(\epsilon )\left( \left\Vert (L- \gamma ){ h}\right\Vert _{\underline{L}^2(\Sigma _{t_*})}^2 + E_\gamma (t_*)[{h}] + \left\Vert h\right\Vert _{\underline{L}^2(\Sigma _{t_*})}^2 \right) . \end{aligned}$$
3. If in addition, *h* is assumed to vanish on $\mathcal {H}^+_-$ and $\overline{\mathcal {H}}^+_+$, then for any $\epsilon >0$, there exists some constant $C_{\textbf{N}}(\epsilon )>0$ such that
  114 $$\begin{aligned} -\partial _{t_*} \mathcal {E}_\gamma (t_*)[{h}] \le {}&\max _{\mathcal {H}=\mathcal {H}^+, \overline{\mathcal {H}}^+}\left( -\textbf{s}_{L}^*[\mathcal {H}] + \kappa _{\mathcal {H}} + \varepsilon _{\textbf{N}} + \epsilon \right) \mathcal {E}_\gamma (t_*)[{h}]\nonumber \\&+C_{\textbf{N}}(\epsilon )\left( \left\Vert (L- \gamma ){h}\right\Vert _{\underline{L}^2(\Sigma _{t_*})}^2 + E_\gamma (t_*)[h] + \left\Vert h\right\Vert _{\underline{L}^2(\Sigma _{t_*})}^2 \right) . \end{aligned}$$

#### Proof

We again fix $\textbf{N}$ as constructed in Proposition 2.7 with $\varepsilon _{\textbf{N}}$, and $X = \textbf{S}'$. The first conclusion in (112) follows directly from (111) and (97).

To prove (113), observe that for $\mathcal {H}= \mathcal {H}^+_-, \overline{\mathcal {H}}^+_+$, we have that

$$\begin{aligned} \int _{\mathcal {H}}J^{\textbf{N}, 0, -\gamma \textbf{N}^\flat }[h]\cdot n_{\mathcal {H}} = \int _{\mathcal {H}}J^{\textbf{N}, 0, 0}[h]\cdot n_{\mathcal {H}} - \int _{\mathcal {H}}\gamma \left\lvert h\right\rvert ^2g(\textbf{N}, n_{\mathcal {H}}). \end{aligned}$$

Moreover, for $\gamma >0$, we have that for $\mathcal {H}= \mathcal {H}^+_-, \overline{\mathcal {H}}^+_+$,

$$\begin{aligned} \int _{\mathcal {H}}\gamma \left\lvert h\right\rvert ^2g(\textbf{N}, n_{\mathcal {H}})< 0. \end{aligned}$$

As a result, following the proof of (98), we have that

$$\begin{aligned} \partial _{t_*}\mathcal {E}_\gamma (t_*)[h]\le \int _{\Sigma _{t_*}}\nabla \cdot J^{\textbf{N}, 0, -\gamma \textbf{N}^\flat }[h]\,\sqrt{A}. \end{aligned}$$

Using the divergence property (24), we have that

$$\begin{aligned} \nabla \cdot J^{\textbf{N}, 0, \!-\!\gamma \textbf{N}^\flat }[h] \!= \!\Re \left[ \textbf{N}\overline{h}\cdot \left( (L\!- \!\gamma )h \!-\! \textbf{S}[h] \!-\! \textbf{V}[h]\right) \right] \!\!+\! K^{\textbf{N}, 0, \!-\!\gamma \textbf{N}^\flat }[h] \!+\! \Re \left[ \textbf{N}\overline{h}\cdot \gamma h \right] , \end{aligned}$$

where we recall that

$$\begin{aligned} K^{\textbf{N}, 0, -\gamma \textbf{N}^\flat }[h] + \Re \left[ \textbf{N}\overline{h}\cdot \gamma h \right] = K^{\textbf{N}, 0, 0}[h] - \frac{1}{2}\gamma \nabla \cdot \textbf{N}\left\lvert h\right\rvert ^2. \end{aligned}$$

From Proposition 2.7, we have that $\nabla \cdot \textbf{N}<0$ in a small neighborhood of the horizons. Thus, we have that there exists some $C>0$ such that

$$\begin{aligned} \int _{\Sigma _{t_*}}K^{\textbf{N}, 0, -\gamma \textbf{N}^\flat }[h]\,\sqrt{A} + \Re \left\langle \textbf{N}h,\gamma h\right\rangle _{\underline{L}^2(\Sigma _{t_*})} > \int _{\Sigma _{t_*}}K^{\textbf{N}, 0, 0}[h]\,\sqrt{A} - C E_{\gamma }(t_*)[h] \end{aligned}$$

Then, repeating the proof of (98) yields (113) directly. $\square$

### $\textbf{N}$ as a Commutator

We use the redshift vectorfield $\textbf{N}$ not only as a multiplier but also as a commutator in order to derive higher-order energy estimates. This will improve the domain on which we define quasinormal modes.

#### Theorem 6.6

Consider some ${h}\in C^\infty _0(\mathcal {M}, \mathbb {C}^D)$. Define *f* as

115 $$\begin{aligned} L{h} =f. \end{aligned}$$

Then,

1. There exists a finite set of vectorfields $\left\{ \mathcal {K}_i\right\} _{i=1}^N$, which span the set of smooth vectorfields over $\mathcal {M}$, such that for $\textbf{h} = ({h}, \mathcal {K}_1{h}, \cdots , \mathcal {K}_N{h})$, $\textbf{h}$ satisfies
  116 $$\begin{aligned} \textbf{L}\textbf{h} = \textbf{f}\end{aligned}$$
  where $\textbf{L}$ is a strongly hyperbolic operator constructed from *L*, which acts on vectors in $\mathbb {C}^{D'}$, $D' = D(N+1)$, and $f'$ is defined by
  117 $$\begin{aligned} \textbf{f}_0 = f,\quad \textbf{f}_i=\mathcal {K}_if. \end{aligned}$$
  We also have
  118 $$\begin{aligned} \textbf{s}_{\textbf{L}}[\mathcal {H}] = \textbf{s}_{L}[\mathcal {H}] - 2\kappa _{\mathcal {H}}, \end{aligned}$$
  where $\mathcal {H}$ is either $\mathcal {H}^+$ or $\overline{\mathcal {H}}^+$.
2. Conversely, suppose that $\textbf{h}\in C^\infty _0(\mathcal {M}; \mathbb {C}^{D'})$ solves (116) with $\textbf{f}$ having the form (117) for some *f*. Then, defining ${h}{:=} \textbf{h}_0$, and $\delta \textbf{h}= (\textbf{h}_i - \mathcal {K}_i{h})_{i=1,\cdots N}$, we have that $\delta \textbf{h}$ satisfies
  $$\begin{aligned} \textbf{L}'\delta \textbf{h} = 0 \end{aligned}$$
  for a strongly hyperbolic operator $\textbf{L}'$ acting on vectors of dimension *DN* such that $\textbf{s}_{\textbf{L}'}= \textbf{s}_{\textbf{L}}$. If the initial conditions imply that $\gamma _0(\delta \textbf{h})=0$, then actually we have that *h* solves $L{h} = f$.

It is a simple calculation to verify the following commutation lemma.

#### Lemma 6.7

Let *K* be a smooth vectorfield on $\mathcal {M}$. Then, for sufficiently smooth *h*,

We now have the tools necessary to prove the main theorem of this section.

#### Proof of Theorem 6.6

Let us first prove the first part of the theorem. The main idea here will be to commute the equation

119 $$\begin{aligned} L{h} = f \end{aligned}$$

with the set of vectorfields $\mathcal {K}_i$ constructed in Lemma 2.8. Then, by rewriting the resulting equation as a system, we will analyze the subprincipal operator at $\mathcal {H}^+$ and $\overline{\mathcal {H}}^+$ to verify that it satisfies (118).

**Step 1: Commuting the equation.** Commuting Eq. (119) with the $\mathcal {K}_i$ vectorfields, we have120Using (32), we can write thatwhere repeated *j*, *k* indices are summed over $1, \cdots , N$. Applying (32) again,

$$\begin{aligned} {\text {tr}}_g ^{({\mathcal {K}_i})}\pi = \nabla \cdot \mathcal {K}_i = f^{jk}_ig(\mathcal {K}_j, \mathcal {K}_k). \end{aligned}$$

Thus, we can rewrite

$$\begin{aligned} \mathcal {K}_if = \Box _{g}\mathcal {K}_i{h} +\textbf{S}[\mathcal {K}_i{h}] + \textbf{V}\mathcal {K}_i{h} - {\textsf{S}_i}^j \mathcal {K}_j{h} - \textsf{S}_i {h} + [\mathcal {K}_i, \textbf{V}]{h} , \end{aligned}$$

where  and $\textsf{S}_i$ are smooth vectorfields on $\mathcal {M}$ given byTo verify the improvement that we gain from commuting, i.e. that $\textbf{s}_{\textbf{L}}[\mathcal {H}] =\textbf{s}_{L}[\mathcal {H}]-2\kappa _{\mathcal {H}}$, it is necessary to analyze the first-order terms  and $\textsf{S}_i$ at the horizons. Since we are working in a system after a commutation, it will turn out that despite containing first-order derivatives of *h*, $\textsf{S}_i$ will turn out to be part of the zero-order potential operator in the new system, while  will contribute be the main subprincipal term of the new system. To this end, let us first consider the term . Define $\widetilde{\kappa }{:=} \widetilde{\kappa }(r)$ to be a smooth cutoff function function such that

121 $$\begin{aligned} \widetilde{\kappa } = {\left\{ \begin{array}{ll} \kappa _{\mathcal {H}^+} & {\text { near }} \mathcal {H}^+, \\ 0 & {\text { away from }}\mathcal {H}^+, \overline{\mathcal {H}}^+,\\ \kappa _{\overline{\mathcal {H}}^+} & {\text { near }}\overline{\mathcal {H}}^+. \end{array}\right. } \end{aligned}$$

Adding and subtracting $2\widetilde{\kappa }\mathcal {K}_1\mathcal {K}_i{h}$,We then use $[\mathcal {K}_i,\mathcal {K}_j] = \mathcal {K}_i\mathcal {K}_j - \mathcal {K}_j\mathcal {K}_i$ to writefor any . We then define  such that on $\mathcal {H}^+$ and $\overline{\mathcal {H}}^+$,Next, we definewhich has the crucial property that  vanishes on both $\mathcal {H}^+$ and $\overline{\mathcal {H}}^+$, and  so thatWe can now rewrite (120) in the following manner:122which can be written as a system of equations for $\textbf{h}_i$, where $\textbf{h}_i = \mathcal {K}_i{h}$ for $i>0$, and $\textbf{h}_0={h}$.where the Einstein summation notation denotes summation over $j=1, \cdots , N$, andWe now have equations for $\textbf{h}_i, i\ne 0$. It remains to derive an equation for $\textbf{h}_0={h}$. In particular, we cannot use the original equation $\textbf{L}\textbf{h}_0 = L\textbf{h}_0$ since we want to derive an equation consistent with $\textbf{s}_{\textbf{L}}[\mathcal {H}] =\textbf{s}_{L}[\mathcal {H}] - 2\kappa _{\mathcal {H}}$. Instead, let us rewrite the main Eq. (119) using the fact that $\textbf{h}_1 = \mathcal {K}_1\textbf{h}_0$,

123 $$\begin{aligned} f = \Box _{g}\textbf{h}_0 + (\textbf{S}- 2\widetilde{\kappa } \mathcal {K}_1)\textbf{h}_0 + \textbf{V}\textbf{h}_0 + 2\widetilde{\kappa } \textbf{h}_1 . \end{aligned}$$

We then defineWith these definitions, we see that we can combine the equations (122) and (123) to writeThen, since we have that  vanishes on the horizons, we see thatThis directly implies that

$$\begin{aligned} \begin{aligned} \textbf{s}_{\textbf{L}}[\mathcal {H}^+]&= \textbf{s}_{L}[\mathcal {H}^+] - 2\kappa _{\mathcal {H}^+},\\ \textbf{s}_{\textbf{L}}[\overline{\mathcal {H}}^+]&= \textbf{s}_{L}[\overline{\mathcal {H}}^+] - 2\kappa _{\overline{\mathcal {H}}^+}. \end{aligned} \end{aligned}$$

**Step 2: Verifying the propagation of the constraint.** We now prove the second part of the theorem. The goal in this part of the theorem will be to show that the extended system of equations propagates the constraints $\mathcal {K}_i\textbf{h}_0 - \textbf{h}_i$.

Define

$$\begin{aligned} \delta \textbf{h}_i = \mathcal {K}_i\textbf{h}_0 - \textbf{h}_i. \end{aligned}$$

Then we can rewrite the equation for $\textbf{h}_0$ in equation (123), as

$$\begin{aligned} L\textbf{h}_0 - 2\widetilde{\kappa } (\delta \textbf{h}_1) = \textbf{f}_0. \end{aligned}$$

Commuting this equation with $\mathcal {K}_i$ and repeating the algebra above leading to Eq. (122) we recoverIf we have that

$$\begin{aligned} 0 =\mathcal {K}_i\textbf{f}_0 - \textbf{f}_i,\quad 1\le i\le N, \end{aligned}$$

then,This can be rewritten aswhere $\textbf{L}'$ is a strongly hyperbolic operator withFrom the hyperbolic nature of this system, it is clear that having initial data $\delta \textbf{h}\vert _{\Sigma _0}=0$ implies that $\delta \textbf{h}=0$ identically and thus the extended system (116) reduces back to the original uncommuted equation (115). It remains to check that $\textbf{s}_{\textbf{L}'}[\mathcal {H}] = \textbf{s}_{\textbf{L}}[\mathcal {H}]$. To this end, we can evaluateso $\textbf{s}_{\textbf{L}'}[\mathcal {H}] = \textbf{s}_{\textbf{L}}[\mathcal {H}].$ $\square$

We thus have the following higher-regularity equivalent of Corollary 6.4:

#### Corollary 6.8

Let $L$ be a strongly hyperbolic operator on a slowly-rotating Kerr-de Sitter background, and suppose that $\psi \in L^2(\mathbb {R}_+,H^{1}(\Sigma ))$ satisfies $\textbf{T}\psi \in L^2(\mathbb {R}_+, L^2(\Sigma ))$ and is a weak solution of the Cauchy problem

$$\begin{aligned} \begin{aligned} L\psi&= f,\\ \gamma _0(\psi )&=(\psi _0, \psi _1), \end{aligned} \end{aligned}$$

where $(\psi _0, \psi _1)\in \textbf{H}^{k}(\Sigma )$, and $f\in H^k(\mathcal {M})$. Then $\psi \in C^0(\mathbb {R}^+, H^1(\Sigma ))$ with $\textbf{T}\psi \in C^0(\mathbb {R}^+,L^2(\Sigma ))$ and moreover, the following holds.

1. For any ${T_*}>0$ and $k\ge 1$, $\psi$ satisfies the energy estimate
  $$\begin{aligned} \sup _{t_*\le {T_*}}e^{-\textbf{M}t_*}\left\Vert \psi \right\Vert _{\overline{H}^{k}(\Sigma )} \le C \left\Vert (\psi _0, \psi _1)\right\Vert _{\textbf{H}^{k}(\Sigma _0)} + \int _{0}^{{T_*}}e^{-\textbf{M}t_*}\left\Vert f\right\Vert _{\overline{H}^{k-1}(\Sigma _{t_*})}\,dt_*\end{aligned}$$
  for some constants $C, \textbf{M}$ depending on *g*, $L$, and *k*.
2. On the Laplace-transformed side, there exists $\textbf{M}$ and *C* such that for $\Im \sigma > \textbf{M}$,
  $$\begin{aligned} \left\Vert u\right\Vert _{\underline{H}^{k}_\sigma (\Sigma )}\lesssim \left\Vert \widehat{L}(\sigma )u\right\Vert _{\underline{H}^{k-1}_\sigma (\Sigma )}. \end{aligned}$$

#### Proof

We prove the result by commuting the equation with the vectorfields $\mathcal {K}_i$ a sufficient number of times and then applying Corollary 6.4. $\square$

## Tools for Frequency Analysis

In this section we introduce tools used in the frequency analysis of Sect. 8.

### Pseudodifferential Analysis

In this section, we introduce the basics of the classical pseudo-differential analysis we will be using. We first introduce the necessary pseudo-differential calculus on $\mathbb {R}^n$ before defining pseudo-differential operators on manifolds, which is what we will actually use in the pseudo-differential arguments involved in Sect. 8. For an in-depth reference, we refer the reader to Chapter 1 of [1], Chapter 18 of [34], or Chapters 1-4 of [57].

#### Definition 30

For $m\in \mathbb {R}$, we define $S^{m}(\mathbb {R}^d)$ to be the class of order-*m* symbols on $\mathbb {R}^d$, consisting of $C^\infty$ functions $a(\textbf{x},\zeta )$ such that

$$\begin{aligned} \left\lvert D_{\textbf{x}}^\beta D_\zeta ^\alpha a(\textbf{x}, \zeta ) \right\rvert \le C_{\alpha \beta }\left\langle \zeta \right\rangle ^{m-\left\lvert \alpha \right\rvert } \end{aligned}$$

for all multi-indexes $\alpha$, where $\left\langle \zeta \right\rangle = (1+\left\lvert \zeta \right\rvert ^2)^{\frac{1}{2}}$. We also define the symbol class

$$\begin{aligned} S^{-\infty }(\mathbb {R}^d) {:=} \bigcap _{m}S^m(\mathbb {R}^d). \end{aligned}$$

To each symbol is its associated *pseudo-differential operator* acting on Schwartz functions $\phi$,

$$\begin{aligned} a(\textbf{x}, D)\phi (\textbf{x}) = \operatorname {Op}(a)\phi (\textbf{x}) {:=} (2\pi )^{-d}\int _{\mathbb {R}^d}e^{\mathbbm {i}\textbf{x}\cdot \zeta }a(\textbf{x}, \zeta )\widehat{\phi }(\zeta )\,d\zeta , \end{aligned}$$

where $\widehat{\phi }$ is the Fourier transform of $\phi$.

#### Remark 24

By abuse of notation, we will understand symbols *a* homogeneous of degree *m* on $\left\lvert \zeta \right\rvert >1$ to also be symbols in $S^m(\mathbb {R}^d)$, since they can be corrected to be proper symbols by some cutoff in $S^{-\infty }(\mathbb {R}^d)$.

We review the basic properties of the pseudo-differential symbol calculus (see for example Theorem I.3.2.3, Theorem I.4.1, and Corollary I.4.1 in [1]).

#### Proposition 7.1

For $m, m_1, m_2\in \mathbb {R}$, let $a\in S^m(\mathbb {R}^d)$, $a_1\in S^{m_1}(\mathbb {R}^d)$, and $a_2\in S^{m_2}(\mathbb {R}^d)$.

1. We have that $a(\textbf{x},D)^* = a^*(\textbf{x},D)$, where
  $$\begin{aligned} a^*(\textbf{x},\zeta ) \sim \sum _{\alpha }\frac{1}{\alpha !}\partial _\zeta ^\alpha D_{\textbf{x}}\overline{a}(\textbf{x},\zeta ), \end{aligned}$$
  where we say that $a\sim \sum a_j$ ($\sum a_j$ is an asymptotic expansion for *a*) if
  $$\begin{aligned} \forall k\ge 0, \qquad a - \sum _{j=0}^ka_j \in S^{m_{k+1}}(\mathbb {R}^d). \end{aligned}$$
2. We have that
  $$\begin{aligned} a_1(\textbf{x},D)\circ a_2(\textbf{x},D) = b(\textbf{x},D), \end{aligned}$$
  where
  $$\begin{aligned} b = a_1\# a_2 \sim \sum _{\alpha }\frac{1}{\alpha !}\partial _\zeta ^\alpha a_1 D_\textbf{x}^\alpha a_2. \end{aligned}$$
3. We have that
  $$\begin{aligned} \left[ a_1(\textbf{x},D), a_2(\textbf{x},D)\right] = b(\textbf{x}, D), \end{aligned}$$
  where,
  $$\begin{aligned} b = \left\{ a_1,a_2\right\} + S^{m_1 + m_2 - 2}(\mathbb {R}^d), \end{aligned}$$
  where
  $$\begin{aligned} \left\{ f,g\right\} = \sum \left( \frac{\partial f}{\partial \zeta ^i}\frac{\partial g}{\partial \textbf{x}^i} - \frac{\partial g}{\partial \zeta ^i}\frac{\partial f}{\partial \textbf{x}^i}\right) \end{aligned}$$
  denotes the *Poisson bracket* of *f* and *g*.

#### Definition 31

We call a symbol $a(\textbf{x}, \zeta )\in S^m(\mathbb {R}^d)$ and its corresponding operator $a(\textbf{x}, D)$ an *elliptic symbol* and an *elliptic operator* respectively if there exists some *c*, *C* such that for $\left\langle \zeta \right\rangle >C$,

$$\begin{aligned} \left\lvert a(\textbf{x}, \zeta )\right\rvert \ge c \left\langle \zeta \right\rangle ^m. \end{aligned}$$

Elliptic operators are particularly convenient objects to work with as they are invertible in a pseudo-differential sense.

#### Proposition 7.2

If $a(\textbf{x}, \zeta )\in S^m(\mathbb {R}^d)$ is elliptic, then it has a *parametrix* $b(\textbf{x}, \zeta )\in S^{-m}(\mathbb {R}^d)$ such that

$$\begin{aligned} a(\textbf{x}, D)b(\textbf{x}, D) - \operatorname {Id}\in \Psi ^{-\infty }, \qquad b(\textbf{x}, D)a(\textbf{x}, D) - \operatorname {Id}\in \Psi ^{-\infty }. \end{aligned}$$

We also state a key proposition regarding the behavior of symbols under coordinate transformations (see Proposition I.7.1 of [1]).

#### Proposition 7.3

Let $\phi :\Omega \rightarrow \Omega '$ be a smooth diffeomorphism between two open subsets of $\mathbb {R}^d$. Moreover, let $a\in S^m(\mathbb {R}^d)$ be an order *m* symbol such that the operator $a(\textbf{x}, D)$ has kernel with compact support in $\Omega \times \Omega$.

Then the following hold.

1. The function $a'(y, \zeta )$ defined by
  $$\begin{aligned} a'(\phi (\textbf{x}), \zeta ) = e^{-\mathbbm {i}\phi (\textbf{x})\cdot \zeta } a(\textbf{x},D) e^{\mathbbm {i}\phi (\textbf{x})\cdot \zeta }, \qquad a' = 0 {\text { for }} y\not \in \Omega ', \end{aligned}$$
  is also a member of $S^m$.
2. The kernel of $a'(\textbf{x},D)$ has compact support in $\Omega '\times \Omega '$,
3. For $$u\in \mathcal {S}'(\mathbb {R}^d)$$,
  $$\begin{aligned} a,( D)(u\circ \phi ) = (a'(\textbf{x}, D)u)\circ \phi . \end{aligned}$$
4. If *a* has the form
  124 $$\begin{aligned} a= a_m \mod S^{m-1}(\mathbb {R}^d), \end{aligned}$$
  where $a_m$ is a homogeneous symbol of order *m*, then the same is true for $a'$. That is, there is a homogeneous symbol $a_m'$ of order *m* such that
  $$\begin{aligned} a'= a_m' \mod S^{m-1}(\mathbb {R}^d), \end{aligned}$$
  and in fact 125

In particular, (125) shows that the principal symbol of a pseudodifferential operator on a manifold $\mathcal {U}$ is a member of $T^*\mathcal {U}$, and is invariant under coordinate transformations. These pseudo-differential operators have well-behaved mapping properties based on their symbol.

#### Proposition 7.4

If $a\in S^m(\mathbb {R}^d)$, then the operator *a*(*x*, *D*) is a well-defined mapping from $H^s(\mathbb {R}^d)$ to $H^{s-m}(\mathbb {R}^d)$ for any $s\in \mathbb {R}$.

We recall below the Coifman-Meyer commutator estimate.

#### Proposition 7.5

(See [10]) For $$f\in C^\infty $$, $$P\in \Psi ^1$$,

$$\begin{aligned} \left\Vert [P,f]u\right\Vert _{L^2} \le C \left\Vert f\right\Vert _{C^1} \left\Vert u\right\Vert _{L^2}. \end{aligned}$$

Two standard pseudo-differential objects that will feature heavily in what follows are the principal symbol of the Laplace-Beltrami operator associated to a Kerr-de Sitter metric and the Hamiltonian vectorfield it generates.

#### Definition 32

For a fixed Kerr-de Sitter metric $g_b$, define $p_b$ to be the *principal symbol* of the Laplace-Beltrami operator associated to $g_b$,

$$\begin{aligned} p_b {:=} g^{\mu \nu }\zeta _\mu \zeta _\nu . \end{aligned}$$

Moreover, for a fixed Kerr-de Sitter metric $g_b$, denote by

$$\begin{aligned} H_{p_b}{:=} \sum _{\mu }\frac{\partial p_b}{\partial x_\mu }\frac{\partial }{\partial \zeta _\mu } - \frac{\partial p_b}{\partial \zeta _\mu } \frac{\partial }{\partial x_\mu },\qquad (x;\zeta ){:=} (t,r,\omega ; \sigma , \xi , \eta ), \end{aligned}$$

the *Hamiltonian vectorfield* associated to $g_b$.

We also have the following inequality which serves as a generalization of Gärding’s inequality which we will make repeated use of in Sect. 8.

#### Theorem 7.6

(Corollary II.8 [55]) Let $\left\{ a_j\right\} _{j=1}^k, b\in C^{1,1}S^1$ be a finite set of real symbols with $\left\lvert b\right\rvert \le \sum \left\lvert a_j\right\rvert$, where $C^{1,1}S^1$ denotes the class of first-order symbols with $C^{1,1}$ coefficients. Then,

$$\begin{aligned} \left\Vert B(x, D)u\right\Vert _{L^2}\lesssim \sum _{j=1}^k\left\Vert A_j(x, D)u\right\Vert _{L^2} + \left\Vert u\right\Vert _{L^2}. \end{aligned}$$

In our application on slowly-rotating Kerr-de Sitter backgrounds, it will be convenient to perform all the calculations involving pseudo-differential calculus in this paper using the Boyer-Lindquist coordinates $(t,r,\omega )$, with $(\sigma , \xi , \eta )$ representing the respective frequency variables, with covectors written as

$$\begin{aligned} \zeta = \sigma \,dt + \xi \,dr + \eta ,\quad \eta \in T^*\mathbb {S}^2, \end{aligned}$$

where we recall that by its construction, $t_*=t$ on a small neighborhood of $r=3M$.

We define two specific classes of symbols, which will come up in our subsequent analysis.

#### Definition 33

Let $S^n(\mathcal {M}) = S^n(t_*,r,\omega ;\sigma ,\xi ,\eta )$ denote the class of order-*n* symbols on $\mathcal {M}$, and $S_{{\operatorname {tan}}}^n(\Sigma ) = S_{{\operatorname {tan}}}^n(r,\theta ;\xi ,\eta )$ denote the sub-class of stationary, axi-symmetric order-*n* symbols independent of $\sigma$ and $t_*$.

Throughout this paper, we will work on a restricted class of pseudo-differential operators of order *n* of the form

$$\begin{aligned} \sum _{i=0}^{n+1}P^{n-m}D_{t_*}^m,\qquad P^{n-m}\in \Psi _{{\operatorname {tan}}}^{n-m}(\Sigma ), \end{aligned}$$

which are differential in $t_*$ and pseudo-differential in the spatial variables. This restricted class was used to prove Morawetz estimates for the scalar wave operator by Tataru and Tohaneanu in [56] and has recently also been used on perturbations of the Schwarzschild wave operator by Tohaneanu and Lindblad [45].

We also introduce the following definition of negative Sobolev spaces.

#### Definition 34

We define the negative Sobolev spaces

$$\begin{aligned} \left\Vert h\right\Vert _{H^{-k}(\mathbb {R}^3)} {:=} \left\Vert \left\langle D_x\right\rangle ^{-k}h\right\Vert _{L^2(\mathbb {R}^3)}. \end{aligned}$$

We remark that since we will only need to use pseudo-differential methods in a neighborhood of the trapped set, all of our symbols will be compactly supported in a neighborhood of $r=3M$.

### Trapping Behavior in Kerr-de Sitter

In this section, we discuss the well-known properties of *trapped null geodesics* in Kerr-de Sitter which remain in a compact spatial region for all time (see for example Proposition 3.1 of [18] and Section 6.4 of [58]). These null geodesics represent a fundamental high-frequency geometric obstacle to decay. To analyze the dynamics of the trapped set ${\Gamma }_b$ in frequency space, we consider null-bicharacteristics rather than null-geodesics, as null-geodesics are just the physical projection of the integral curves of null-bicharacteristics.

It is instructive to first consider the trapped null geodesics in Schwarzschild-de Sitter, where we can write out the trapped set explicitly, and make some fundamental observations. The trapped null geodesics in Kerr-de Sitter are in a sense perturbations of the trapped null geodesics in Schwarzschild-de Sitter. The descriptions of the trapped sets that follow are well known from [17]. However, we have included proofs in the appendix for the descriptions of the trapped sets for the sake of completeness. We will make this notion more rigorous in what follows.

On Schwarzschild-de Sitter, the trapped set can be located entirely in physical space.

#### Lemma 7.7

For $g_{b_0}$ a Schwarzschild-de Sitter background, the trapped set is given by

$$\begin{aligned} {\Gamma }_{b_0} = \left\{ (t, r, \omega ;\sigma , \xi , \eta ): r=3M, \xi =0, p_{b_0}=0\right\} , \end{aligned}$$

where $p_{b_0}$ is the principal symbol of the scalar wave operator $\Box _{g_{b_{0}}}$. Moreover, the trapped set is unstable in the sense that

$$\begin{aligned} \mu _{b_0}> 0,\quad p_{b_0} =0,\quad \pm (r-3M)> 0, \quad H_{p_{b_0}}r = 0 \implies \pm H_{p_{b_0}}^2r > 0, \end{aligned}$$

where we recall from Definition 32 that we denote by $p_{b_0}$ the principal symbol of $\Box _{g_{b_{0}}}$, and by $H_{p_{b_0}}$ the Hamiltonian vectorfield of $g_{b_0}$.

#### Proof

See Appendix E.1. $$\square $$

#### Remark 25

The physical projection of ${\Gamma }_{b_0}$ is exactly the photon sphere, $r=3M$.

We now move onto the trapped set in the case of Kerr-de Sitter. In this case, the trapped set exhibits frequency-dependent behavior.

#### Lemma 7.8

For $g_b$ a Kerr-de Sitter background, the trapped set is

126 $$\begin{aligned} {\Gamma }_{b} = \left\{ (t, r, \omega ; \sigma , \xi , \eta ): r=\mathring{r}_b(\sigma , \eta ), \xi =0, p_{b}=0\right\} , \end{aligned}$$

where $p_{b}$ is the principal symbol of the scalar wave operator $\Box _{g_{b}}$, and $\mathring{r}_b(\sigma , \eta )$ is a function satisfying the following properties.

1. $\mathring{r}_b(\sigma ,\eta )$ lies in an *O*(*a*) neighborhood of $r=3M$ for all $\sigma , \eta$.
2. $\mathring{r}_b(\sigma , \eta )$ is smooth in $\sigma , \eta$, as well as the black hole parameters *b*.

Moreover, the trapped set is unstable in the sense that

127 $$\begin{aligned} \Delta _b>0, \quad p_b=0, \quad \pm (r-\mathring{r}_b)>0, \quad H_{p_b} r=0 \implies \pm H^2_{p_b} r > 0, \end{aligned}$$

where we denote by $H_{p_b}$ the Hamiltonian vectorfield of $g_b$.

#### Proof

See Appendix E.2. $$\square $$

We conclude this section by defining two important cutoff functions that we will subsequently make use of.

#### Lemma 7.9

There exist frequency cutoffs

$$\begin{aligned} \breve{\chi }_\zeta {:=} \breve{\chi }_\zeta (r, \theta ;\xi ,\eta ),\qquad \mathring{\chi }_\zeta {:=} \mathring{\chi }_\zeta (r, \theta ;\xi ,\eta ), \end{aligned}$$

where $(t,r,\theta , \varphi ;\sigma ,\xi , \eta _{\theta }, \eta _{\varphi })$ are the Boyer-Lindquist coordinates, defined so that

128 $$\begin{aligned} \breve{\chi }_{\zeta } = {\left\{ \begin{array}{ll} 1 &  \frac{\left\lvert \xi \right\rvert ^2}{\left\lvert \eta \right\rvert ^2} \ge \delta _\zeta ,\\ 0 &  \frac{\left\lvert \xi \right\rvert ^2}{\left\lvert \eta \right\rvert ^2} < \frac{1}{2} \delta _{\zeta },\\ \end{array}\right. }\qquad \mathring{\chi }_{\zeta } = {\left\{ \begin{array}{ll} 1 &  \frac{\left\lvert \xi \right\rvert ^2}{\left\lvert \eta \right\rvert ^2} \le \delta _\zeta ,\\ 0 &  \frac{\left\lvert \xi \right\rvert ^2}{\left\lvert \eta \right\rvert ^2} > 2 \delta _\zeta , \end{array}\right. } \end{aligned}$$

and some constant $C_{\xi }>0$ sufficiently large such that for $r\in [\mathring{r}_-, \mathring{R}_+]$,

$$\begin{aligned} C_\xi (H_{p_b}r)^2 -p_b \end{aligned}$$

is elliptic on the support of $\breve{\chi }_{\zeta }$ for all $g_b$ sufficiently slowly-rotating Kerr-de Sitter black holes.

#### Proof

First, we observe that $H_{p_b}r = 2\rho _b^{-2}\Delta _b\xi$ in Boyer-Lindquist coordinates. We write

$$\begin{aligned} \rho _b^2p_b = \Delta _b\xi ^2 + \varkappa _b\eta _{\theta }^2 - (1+\lambda _b)^2 \left( \Delta _b^{-1}\left( (r^2+a^2)\sigma + a\eta _{\varphi }\right) ^2 - \varkappa _b^{-1}\sin ^{-2}\theta \left( a\sin ^2\theta \sigma +\eta _{\varphi }\right) ^2 \right) . \end{aligned}$$

Observe that for $a\ll M, \Lambda$ and $r\in [\mathring{r}_-, \mathring{R}_+]$, we have that

$$\begin{aligned} G_b(dt,dt) = -(1+\lambda _b)^2\left( \Delta _b^{-1}(r^2+a^2)^2 - a^2 \varkappa _b^{-1}\sin ^{2}\theta \right) < 0. \end{aligned}$$

As a result, we have that

$$\begin{aligned} \rho _b^2p_b + \frac{1}{2}G_b(dt,dt)\sigma ^2 \lesssim \xi ^2 + \left\lvert \eta \right\rvert ^2. \end{aligned}$$

By the construction of $\breve{\chi }_{\zeta }$ and $\mathring{\chi }_\zeta$ in (128), we have that $(H_{p_b}r)^2 \gtrsim \xi ^2 + \left\lvert \eta \right\rvert ^2$ on the support of $\breve{\chi }_{\zeta }$. As a result, we have that for some $C_\xi$ sufficiently large,

$$\begin{aligned} C_\xi (H_{p_b}r)^2 - \rho _b^2p_b \gtrsim -\frac{1}{2}G_b(dt,dt)\sigma ^2 + \xi ^2 + \left\lvert \eta \right\rvert ^2. \end{aligned}$$

Since $G_b(dt,dt)<0$ and $\rho _b>0$ for $r\in [\mathring{r}_-, \mathring{R}_+]$ we then have that $C_\xi (H_{p_b}r)^2 - p_b$ is elliptic as desired. $\square$

### Pseudo-Differential Modified Divergence Theorem

In this section, we introduce a pseudo-differential modification of the main divergence property presented in equation (22). This modification allows us to handle the frequency-dependent nature of trapping in the Kerr-de Sitter family, and uses small pseudo-differential perturbations of vectorfield multipliers and Lagrangian correctors. We emphasize that this perturbation is only used in Sect. 8.4.2 to prove Theorem 8.9.

We first prove a convenient lemma connecting the frequency analysis with the unperturbed divergence property in equation (24).

#### Lemma 7.10

Let *h* be a complex-valued matrix function $h:\mathcal {M}\rightarrow \mathbb {C}^D$, *g* be a fixed Kerr-de Sitter metric and $p$ the principal symbol of $\Box _{g}$. Then we can rewrite $K^{X,q,0}[h]$ as defined in (23) as

129 $$\begin{aligned} K^{X,q, 0}[{h}] = (\textbf{k}^{X,q}_{(2)})^{\alpha \beta }\nabla _{(\alpha }{h}\cdot \nabla _{\beta )}\overline{{h}} + \textbf{k}^{X,q}_{(0)}\left\lvert {h}\right\rvert ^2, \end{aligned}$$

where $(\textbf{k}^{X,q}_{(2)})^{\alpha \beta }\zeta _\alpha \zeta _\beta$ and $\textbf{k}^{X,q}_{(0)}$ are given by

$$\begin{aligned} (\textbf{k}^{X,q}_{(2)})^{\alpha \beta }\zeta _\alpha \zeta _\beta = \frac{1}{2}H_{p}X + \left( q-\frac{1}{2}\nabla _g\cdot X\right) p,\qquad \textbf{k}^{X,q}_{(0)} = -\frac{1}{2}\nabla ^\alpha \partial _\alpha q. \end{aligned}$$

#### Proof

Observe that

$$\begin{aligned} 2^{({X})}\pi \cdot \mathbb {T}[h] = \mathcal {L}_Xg^{\alpha \beta }\nabla _{(\alpha } h\cdot \nabla _{\beta )}\overline{ h} - (\nabla _g\cdot X) g^{\alpha \beta }\nabla _{\alpha } h\cdot \nabla _{\beta }\overline{ h}. \end{aligned}$$

By the definition of the Hamiltonian vectorfield in Definition 32,

$$\begin{aligned} \mathcal {L}_Xg^{\alpha \beta }\zeta _\alpha \zeta _\beta = H_{p}X. \end{aligned}$$

Equation (129) then follows from the definition of the principal symbol of $\Box _{g}$ in Definition 32 and the definition of $K^{X,q,m}[h]$ in (23). $\square$

Due to the frequency-dependent nature of trapping in the Kerr-de Sitter family, we are not able to use the divergence property in equation (24) directly to prove the desired Morawetz estimates near the trapped set ${\Gamma }_b$. Instead, we use an integration-by-parts variant of the divergence property that uses a pseudo-differential perturbation of the vectorfield multipliers.

First, we observe that

130 $$\begin{aligned} \begin{aligned} \overline{\mathbb {L}}_{g_b}&{:=} Q\mathbb {L}_{g_b}Q^- = \Box _{g_{b}} + \overline{\textbf{S}}_b + \overline{\textbf{V}}_b, \\ \overline{\textbf{S}}_b&{:=} Q\textbf{S}_bQ^{-} + Q\left[ \Box _{g_{b}}, Q^-\right] , \\ \overline{\textbf{V}}_b&{:=} Q\textbf{V}_b Q^-, \end{aligned} \end{aligned}$$

where $Q\in S^0 + S^{-1}D_{t_*}$ is as constructed in (E23), and $Q^-$ denotes its parametrix. In what follows, it will also be convenient to split $\overline{\textbf{S}}_b$ into its Hermitian and skew-Hermitian components, given by

131 $$\begin{aligned} \overline{\textbf{S}}_{b,a} {:=} \frac{1}{2} \left( \overline{\textbf{S}}_b - \overline{\textbf{S}}_b^*\right) ,\qquad \overline{\textbf{S}}_{b,s} {:=} \frac{1}{2}\left( \overline{\textbf{S}}_b + \overline{\textbf{S}}_b^*\right) , \end{aligned}$$

where the adjoint is taken with respect to the $L^2(\mathcal {D})$ norm. Observe that both $\overline{\textbf{S}}_b, \overline{\textbf{S}}_{b,a}$ and $\overline{\textbf{S}}_{b,s}$ belong to $\operatorname {Op}S^{1} + \operatorname {Op}S^0\partial _{t_*}$, and moreover we can write

132 $$\begin{aligned} \begin{aligned} \overline{\textbf{S}}_b = \overline{\textbf{S}}_{0}\partial _{t_*} + \overline{\textbf{S}}_{1},\qquad \overline{\textbf{S}}_{b,a} = \overline{\textbf{S}}_{0,a}\partial _{t_*} + \overline{\textbf{S}}_{1,a},\qquad \overline{\textbf{S}}_{b,s} = \overline{\textbf{S}}_{0,s}\partial _{t_*} + \overline{\textbf{S}}_{1,s}. \end{aligned} \end{aligned}$$

We will similarly define

$$\begin{aligned} \overline{\textbf{V}}_{b,a} {:=} \frac{1}{2} \left( \overline{\textbf{V}}_b - \overline{\textbf{V}}_b^*\right) ,\qquad \overline{\textbf{V}}_{b,s} {:=} \frac{1}{2}\left( \overline{\textbf{V}}_b + \overline{\textbf{V}}_b^*\right) . \end{aligned}$$

#### Proposition 7.11

Let us consider

133 $$\begin{aligned} X_b {:=} X_{b_0} + \widetilde{X},\qquad q_b{:=} q_{b_0} + a\tilde{q}, \end{aligned}$$

where $X_{b_0}$ and $q_{b_0}$ are a smooth vectorfield and a smooth function respectively, and

134 $$\begin{aligned} \begin{aligned} \widetilde{X}&= \widetilde{X}_0\partial _t + \widetilde{X}_1, \\ \tilde{q}&= \tilde{q}_0 + \tilde{q}_{-1}\partial _t, \end{aligned} \end{aligned}$$

where $\widetilde{X}_i, \tilde{q}_i \in \operatorname {Op}S^i(\Sigma )$, and have Schwartz kernels supported in $\mathring{\mathcal {U}}\times \mathring{\mathcal {U}}$, where $\mathring{\mathcal {U}}$ is a compact subset of $(\mathring{r},\mathring{R})\times \mathbb {S}^2$ that contains $(\mathring{r}_-, \mathring{R}_+)$, which in particular includes the base projection of the trapped set ${\Gamma }_b$, and $\widetilde{X}_0$ and $\tilde{q}_0$ are self-adjoint with respect to the $L^2(\Sigma )$ inner product, and $\widetilde{X}_1$ and $\tilde{q}_{-1}$ are skew-adjoint with respect to the $L^2(\Sigma )$ inner product. Moreover, let

$$\begin{aligned} \mathcal {D}{:=}[0,{T_*}]\times \mathring{\Sigma },\qquad \mathring{\Sigma }{:=}[\mathring{r}_-, \mathring{R}_+]\times \mathbb {S}^2. \end{aligned}$$

and let *h* be a function such that for all $t_*$, $h(t_*,\cdot )$ is compactly supported on $\mathring{\Sigma }$. Then

135 $$\begin{aligned}&-\Re \left\langle \overline{\mathbb {L}}_{g_b}{h}, (X_b+q_b){h}\right\rangle _{L^2(\mathcal {D})}\nonumber \\ ={}&\int _{\mathcal {D}}K^{X_{b_0},q_{b_0},0}[{h}] - \Re \left\langle \overline{\textbf{S}}_b[h], (X_{b_0}+q_{b_0}){h}\right\rangle _{L^2(\mathcal {D})} - \Re \left\langle \overline{\textbf{V}}_b{h}, (X_{b_0}+q_{b_0}){h}\right\rangle _{L^2(\mathcal {D})} + a\Re \mathbb {K}^{\widetilde{X}, \tilde{q}}[{h}] \nonumber \\&+ \left. \int _{\Sigma _{t_*}}J^{X_{b_0}, q_{b_0},0}[{h}]\cdot n_{\Sigma }\right| _{t_*=0}^{t_*= {T_*}} + {\Re \left\langle g_b(\textbf{T}, n_{\mathring{\Sigma }})\overline{\textbf{S}}_{0}h, X_{b_0}h\right\rangle _{L^2(\mathring{\Sigma })}}\Bigg \vert _{t_*=0}^{t_*={T_*}} + a \Re \left. \mathbb {J}^{\widetilde{X}, \tilde{q}}(t_*)[{h}]\right| _{t_*=0}^{t_*= {T_*}} . \end{aligned}$$

where

136 $$\begin{aligned} 2 \mathbb {K}^{\widetilde{X}, \tilde{q}}[{h}] ={}&\left\langle \left[ \widetilde{X}, \Box _{g_{b}}\right] {h},{h}\right\rangle _{L^2(\mathcal {D})} + \left\langle \left[ \widetilde{X}, \overline{\textbf{S}}_{b,s}\right] h, h\right\rangle _{L^2(\mathcal {D})} + \left\langle \left( \widetilde{X} \overline{\textbf{S}}_{b,a} + \overline{\textbf{S}}_{b,a} \widetilde{X}\right) {h},{h}\right\rangle _{L^2(\mathcal {D})}\nonumber \\&- \left\langle \left( \tilde{q}\Box _{g_{b}}+ \Box _{g_{b}}\tilde{q}\right) {h}, {h}\right\rangle _{L^2(\mathcal {D})} - 2\left\langle \overline{\textbf{S}}_{b}{h}, \tilde{q} {h}\right\rangle _{L^2(\mathcal {D})} - 2\left\langle \overline{\textbf{V}}_{b}{h},\left( \widetilde{X}+\tilde{q}\right) {h}\right\rangle _{L^2(\mathcal {D})},\nonumber \\ \mathbb {J}^{\widetilde{X}, \tilde{q}}(t_*)[{h}] ={}&\left\langle n_{\mathring{\Sigma }}{h}, \widetilde{X}{h}\right\rangle _{L^2(\mathring{\Sigma }_{t_*})} + \left\langle \overline{\textbf{S}}_b{h}, \widetilde{X}_0 g_b(\textbf{T}, n_{\mathring{\Sigma }}){h}\right\rangle _{L^2(\mathring{\Sigma }_{t_*})} + \left\langle n_{\mathring{\Sigma }}{h}, \tilde{q}{h}\right\rangle _{L^2(\mathring{\Sigma }_{t_*})} \nonumber \\&+ \left\langle g_{b}(\textbf{T}, n_{\mathring{\Sigma }})\overline{\textbf{S}}_{0}h, \widetilde{X} h \right\rangle _{L^2(\mathring{\Sigma }_{t_*})} . \end{aligned}$$

#### Proof

See Appendix E.7. $$\square $$

We can decompose $\mathbb {K}^{\widetilde{X}, \tilde{q}}[{h}]$ into its principal, subprincipal, and zeroth order components as follows

137 $$\begin{aligned} \mathbb {K}^{\widetilde{X}, \tilde{q}}[{h}] = \mathbb {K}^{\widetilde{X}, \tilde{q}}_{(2)}[{h}] + \mathbb {K}^{\widetilde{X}, \tilde{q}}_{(1)}[{h}] + \mathbb {K}^{\widetilde{X}, \tilde{q}}_{(0)}[{h}], \end{aligned}$$

where

138 $$\begin{aligned} \begin{aligned} \mathbb {K}^{\widetilde{X}, \tilde{q}}_{(2)}[{h}] ={}&\frac{1}{2}\left\langle \left( \left[ \widetilde{X}, \Box _{g_{b}}\right] + \left( \widetilde{X} \overline{\textbf{S}}_{b,a} + \overline{\textbf{S}}_{b,a}\widetilde{X} \right) - \left( \Box _{g_{b}}\tilde{q} + \tilde{q}\Box _{g_{b}} \right) \right) {h}, {h}\right\rangle _{L^2(\mathcal {D})}\\ \mathbb {K}^{\widetilde{X}, \tilde{q}}_{(1)}[{h}] ={}&-\frac{1}{2}\left\langle \left[ \overline{\textbf{S}}_{b,s}, \widetilde{X}\right] {h},{h}\right\rangle _{L^2(\mathcal {D})} - \left\langle \overline{\textbf{S}}_{b} {h}, \tilde{q} {h}\right\rangle _{L^2(\mathcal {D})} - \left\langle \overline{\textbf{V}}_b {h}, \widetilde{X} {h}\right\rangle _{L^2(\mathcal {D})},\\ \mathbb {K}^{\widetilde{X}, \tilde{q}}_{(0)}[{h}] ={}&-\left\langle \overline{\textbf{V}}_b {h}, \tilde{q} {h}\right\rangle _{L^2(\mathcal {D})}. \end{aligned} \end{aligned}$$

We have a similar decomposition of the boundary terms

$$\begin{aligned} \mathbb {J}^{\widetilde{X}, \tilde{q}}(t_*)[{h}] = \mathbb {J}^{\widetilde{X}, \tilde{q}}_{(2)}(t_*)[{h}] + \mathbb {J}^{\widetilde{X}, \tilde{q}}_{(1)}(t_*)[{h}], \end{aligned}$$

where

139 $$\begin{aligned} \begin{aligned} \mathbb {J}^{\widetilde{X}, \tilde{q}}_{(2)}(t_*)[{h}] {:=}{}&\left\langle n_{\mathring{\Sigma }}{h},\widetilde{X}{h}\right\rangle _{L^2(\mathring{\Sigma }_{t_*})},\\ \mathbb {J}^{\widetilde{X}, \tilde{q}}_{(1)}(t_*)[{h}] {:=}{}&\left\langle \overline{\textbf{S}}_b{h}, \widetilde{X}_{0}g_b(\textbf{T}, n_{\mathring{\Sigma }}){h}\right\rangle _{L^2(\mathring{\Sigma }_{t_*})} + \left\langle n_{\mathring{\Sigma }}{h}, \tilde{q}{h}\right\rangle _{L^2(\mathring{\Sigma }_{t_*})} \\&+ \left\langle g_{b}(\textbf{T}, n_{\mathring{\Sigma }})\overline{\textbf{S}}_{0,a}h, \widetilde{X} h \right\rangle _{L^2(\mathring{\Sigma }_{t_*})}. \end{aligned} \end{aligned}$$

We observe that similar to Lemma 7.10, we have the following symbolic representation of the principal bulk term $\mathbb {K}^{\widetilde{X}, \tilde{q}}_{(2)}$.

#### Lemma 7.12

Let *h* be a complex-valued matrix function $h:\mathcal {M}\rightarrow \mathbb {C}^D$, $g_b$ be a fixed slowly-rotating Kerr-de Sitter metric and $p_b$ be the principal symbol of $\Box _{g}$. Furthermore, let $X_b$, $q_b$ be as defined in Proposition 7.11, and $X_{b_0}$, $q_{b_0}$ be as defined in Lemma 8.8. Then

$$\begin{aligned} K^{X_{b_0}, q_{b_0},0}[h] + a\mathbb {K}^{\widetilde{X}, \tilde{q}}[h] \end{aligned}$$

has principal symbol given by

$$\begin{aligned} \frac{1}{2}H_{p}(\varkappa _{b_0} + a \tilde{\varkappa }) - \overline{\textbf{s}}_b(\varkappa _{b_0} + a \tilde{\varkappa }) + p_b(\mathfrak {q}_{b_0}+a\tilde{\mathfrak {q}}), \end{aligned}$$

where

$$\begin{aligned} \varkappa _{b_0} = \mathbbm {i}f_{b_0}\xi ,\qquad \tilde{\varkappa } = \tilde{\varkappa }_0\sigma + \tilde{\varkappa }_1, \mathfrak {q}_{b_0}= q_{b_0} - \frac{1}{2}\nabla _{g_{b_0}}\cdot X_{b_0}, \qquad \tilde{q} = \tilde{\mathfrak {q}}_{-1}\sigma + \tilde{\mathfrak {q}}_0. \end{aligned}$$

#### Proof

The conclusion follows from the form of $\mathbb {K}^{\widetilde{X}, \tilde{q}}_{(2)}[h]$ in (138) and Lemma 7.10. $\square$

### Subprincipal Symbol of $\mathbb {L}_{g_b}$ at Trapping

The presence of a nontrivial non-signed subprincipal operator in $\mathbb {L}_{g_b}$ poses a considerable obstacle in proving the desired high-frequency Morawetz estimate. Fortunately, the subprincipal operator of $\mathbb {L}_{g_b}$ possesses an appropriate microlocal smallness at ${\Gamma }_b$ that is enough to close the desired high-frequency Morawetz estimate in Sect. 8.4. This smallness was also critical to the proof in [31] (See Theorem 4.4 in [31]). We will first specify what we mean when we refer to the subprincipal operator^13^, and then uncover the desired smallness at the trapped set.

#### The Invariant Subprincipal Operator

Let $\mathcal {E}$ be a tensor bundle over a manifold *X*. The main case of interest in this paper will be when $\mathcal {E}$ is the cotangent bundle $T^*X$ or the bundle of symmetric two-tensors $S^2T^*X$. The main property we are interested in is the norm of the skew-adjoint component of the subprincipal operator. For convenience, we remove the dependence of adjoints on a volume density by tensoring all bundles with the half-density bundle $\Omega ^{\frac{1}{2}}$ over *X*.

It is well-known that if $P\in S^m(X,\mathcal {E}\otimes \Omega ^{\frac{1}{2}})$ is a sum of homogeneous symbols

$$\begin{aligned} p \sim \sum p_m,\qquad p_j \in S^j_{hom}(T^*X\backslash 0, \mathbb {C}^{N\times N}) \end{aligned}$$

with $p_j$ being a homogeneous symbol of degree *j* valued in complex $N\times N$ matrices, that the subprincipal symbol

140 $$\begin{aligned} \sigma _{sub}(P) {:=} p_{m-1} - \frac{1}{2\mathbbm {i}}\sum _j \partial _{x_j\xi _j}p_m(x,\xi ) \in S^{m-1}_{hom}(T^*X\backslash 0, \mathbb {C}^{N\times N}) \end{aligned}$$

is well-defined under changes of coordinates (see [34] Theorem 18.1.33). However, the subprincipal symbol as defined above does still depend on the choice of local trivialization of $\mathcal {E}$. We would like a frame-independent notion of the subprincipal symbol since this would allow us to choose convenient local frames in explicit computations. Fortunately, as shown in [27], there exists a modification of (140) which is independent both of the choice of local trivialization and of local coordinates on $\mathcal {M}$. We review the basics of the construction here as well as the key features of the invariant subprincipal symbol that we will use. For a more thorough discussion, we refer the reader to Sects. 3.3 and 4 of [27]. The results here on the invariant subprincipal symbol are specialized cases of more general results in the literature. For the subsequent results, we list both a reference for the general result, and provide a proof in the appendix for completeness.

##### Definition 35

Consider $P\in \Psi ^m(X, \mathcal {E}\otimes \Omega ^{\frac{1}{2}})$ with scalar principal symbol *p*. Moreover, let $\left\{ e_k(x)\right\} _{k=1}^N$ be a local frame of $\mathcal {E}$, and define the operators $P_{jk}\in \Psi ^m (X, \Omega ^{\frac{1}{2}})$ by

$$\begin{aligned} P_{jk}\left( \sum _k u_k(x)e_k(x)\right) = \sum _{jk}P_{jk}(u_k)e_j(x), u_k\in C^\infty (X, \Omega ^{\frac{1}{2}}). \end{aligned}$$

Then we define the *invariant subprincipal operator* $S_{sub}(P)\in \textit{Diff}^1(T^*X\backslash 0, \pi ^*\mathcal {E})$ by

141 $$\begin{aligned} S_{sub}(P)\left( \sum _k q_k(x,\zeta )e_k(x) \right) {:=} \sum _{jk}(\sigma _{sub}(P_{jk})q_k)e_j - \mathbbm {i}\sum _{k}H_{p}q_k e_k. \end{aligned}$$

Observe that $S_{sub}(P)$ is homogeneous of degree $m-1$ with respect to dilations in the fibers of $T^*X\backslash 0$, and that in a local frame, can be understood as a matrix of first-order differential operators.

The main property of the invariant subprincipal symbol, and indeed its very nomenclature comes from the fact that it is invariant under both changes of coordinates and changes of frame.

##### Lemma 7.13

Let $P\in \Psi ^m(X, \mathcal {E}\otimes \Omega ^{\frac{1}{2}})$ with scalar principal symbol *p*, and let $\{e_k(x)\}_{k=1}^N$ and $\{e'_k\}_{k=1}^N$ be two local frames of $\mathcal {E}$ such that

$$\begin{aligned} e_j(x) = C(x)e_j'(x), \qquad C\in C^\infty (U,{\text {End}}(\mathcal {E})). \end{aligned}$$

Then, we have that

$$\begin{aligned} S_{sub}^e(P) = S_{sub}^{e'}(P). \end{aligned}$$

##### Proof

We can directly compute

$$\begin{aligned} \sigma _{sub}(C^{-1}PC) = (C^{e'})^{-1}\sigma _{sub}^{e'}C^{e'} - \mathbbm {i}(C^{e'})^{-1}H_{p}(C^{e'}), \end{aligned}$$

where $C^{e'}$ is the matrix of *C* in the frame $e'$. Then observe that

$$\begin{aligned} (C^{-1}PC)^{e'} = P^e,\qquad (C^{e'})^{-1}H_p(C^{e'}) = (C^{e'})^{-1}H_p C^{e'} - H_p. \end{aligned}$$

As a result, we have that

$$\begin{aligned} \sigma _{sub}^e(P) - \mathbbm {i}H_p = (C^{e'})^{-1}(\sigma ^{e'}_{sub}(P)-\mathbbm {i}H_p)C^{e}, \end{aligned}$$

which is exactly the desired invariance, where we remark that since the principal symbol *p* of *P* is a scalar^14^ and is well-defined independently of the choice of frame. $\square$

The main application in this paper will be to calculate $S_{sub}(\nabla \cdot \nabla )$ the invariant subprincipal operator of the Laplace-Beltrami operator acting on sections of the tensor bundle at particular regions in phase space when conjugated by an appropriate a zero-order operator. To this end, we consider the following lemma (see Proposition 3.11 in [27] for a more general statement).

##### Lemma 7.14

(Proposition 3.11 in [27]) Let $P\in \Psi ^2(X, \mathcal {E}\otimes \Omega ^{\frac{1}{2}})$ be a pseudo-differential operator with real scalar principal symbol. Suppose that $Q\in \Psi ^{0}(X, \mathcal {E}\otimes \Omega ^{\frac{1}{2}})$ is an operator acting on $\mathcal {E}$-valued half-densities with principal symbol *q*. Then

$$\begin{aligned} \sigma ^{1}(\left[ P,Q\right] ) = \left[ S_{sub}(P), Q\right] , \end{aligned}$$

and if *Q* is elliptic with parametric $Q^-$, then

$$\begin{aligned} S_{sub}(QPQ^-) = q S_{sub}(P)q^{-1}. \end{aligned}$$

In addition,

$$\begin{aligned} \sigma ^{1}(P - P^*) = \frac{1}{2\mathbbm {i}}\left( S_{sub}(P) - S_{sub}(P)^*\right) . \end{aligned}$$

In particular then,

$$\begin{aligned} \sigma ^{1}\left( QPQ^- - (QPQ^-)^*\right) = \frac{1}{2\mathbbm {i}}\left( q S_{sub}(P)q^{-1} - (q S_{sub}(P)q^{-1})^*\right) . \end{aligned}$$

##### Proof

See Appendix E.3. $$\square $$

As the main application of interest, the invariant subprincipal symbol of the Laplace-Beltrami operator acting on the bundle $T_k\mathcal {M}{:=} \otimes ^k T^*\mathcal {M}$ of covariant tensors of rank *k* has a particularly nice form.

##### Lemma 7.15

(Proposition 4.1 in [27]) Let $(\mathcal {M}, g)$ be a smooth manifold equipped with a metric tensor^15^*g*. Let $\bigtriangleup ^{(k)} = {\text {tr}}\nabla ^2 \in \textit{Diff}^2(\mathcal {M}, T_k\mathcal {M})$ be the Laplace-Beltrami operator on $\mathcal {M}$ acting on the bundle $T_k\mathcal {M}$. Then

142 $$\begin{aligned} S_{sub}(\bigtriangleup ^{(k)}) = -\mathbbm {i}\nabla ^{\pi ^*T_k\mathcal {M}}_{H_G}, \end{aligned}$$

where $\pi : T^*\mathcal {M}\backslash 0\rightarrow \mathcal {M}$ is the bundle projection, and $H_{G}$ is the Hamiltonian vectorfield of $G = g^{-1}$.

##### Proof

See Appendix E.4. $$\square $$

This in particular gives us a convenient computation regarding the invariant subprincipal symbol of  the Laplace-Beltrami operator acting on $T_k\mathbb {S}^2$.

##### Lemma 7.16

(Proposition 9.1 in [31]) Let $\pi =\pi _{\mathbb {S}^2}$. Away from the zero section, we split

$$\begin{aligned} \pi ^*T^*\mathbb {S}^2 = E\oplus F, \qquad E(y,\eta ) = {\text {Span}}\,(\eta ),\qquad F(y, \eta ) = \eta ^\perp . \end{aligned}$$

Then $\nabla ^{\pi ^*T_k\mathbb {S}^2}_{H_{\left\lvert \eta \right\rvert ^2}}$ is diagonal in this splitting in the sense that it preserves both the space of sections of *E* and the space of sections of *F*.

##### Proof

See Appendix E.5. $$\square $$

#### The Subprincipal Operator of $\mathbb {L}_{g_b}$

We are now ready to define the microlocal smallness we require at the trapped set in proving a Morawetz estimate near trapping in Sect. 8.

##### Lemma 7.17

Fix $b_0$ black hole parameters of a subextremal member of the Schwarzschild-de Sitter family and some $\varepsilon _{{\Gamma }_{b_0}} > 0$. Then, there exists a stationary, elliptic^16^$Q\in \Psi ^0$ with Schwartz kernel supported near ${\Gamma }_{b_0}$, with parametrix $Q^-$ such that in the $(t, r, \omega ;\sigma ,\xi , \eta )$ Boyer-Lindquist coordinates for Schwarzschild-de Sitter,

143 $$\begin{aligned} \left. \frac{1}{2}\left\lvert \eta \right\rvert ^{-1} \overline{\textbf{s}}_{b_0, a}\right| _{{\Gamma }_{b_0}} < \varepsilon _{{\Gamma }_{b_0}}, \end{aligned}$$

where the operators

144 $$\begin{aligned} \overline{\textbf{S}}_{b_0, a} {:=} \frac{1}{2}\left( \overline{\textbf{S}}_{b_0} -\overline{\textbf{S}}_{b_0}^*\right) ,\qquad \overline{\textbf{S}}_{b_0}{:=}Q\textbf{S}_{b_0}Q^- + Q\left[ \Box _{g_{b_{0}}}, Q^-\right] , \end{aligned}$$

have principal symbols $\overline{\textbf{s}}_{b_0,a}$ and $\overline{\textbf{s}}_{b_0}$ respectively, and the adjoint in (144) is taken with respect to the $L^2([0,{T_*}]\times \Sigma )$ Hermitian inner product.

##### Proof

See Appendix E.6. $$\square $$

##### Remark 26

In practice we will choose $\varepsilon _{{\Gamma }_{b_0}} < {\boldsymbol{\alpha }}_{b_0}$, so that the spectral gap is strong enough to overcome any potentially harmful contribution arising from the subprincipal component of the operator.

This smallness at the trapped set in particular implies the following convenient property of the subprincipal operator in a microlocal neighborhood of the trapped set ${\Gamma }_b$. We start with a decomposition lemma that will prove critical in what follows.

##### Lemma 7.18

Fix some $\delta _0>0$, and let $\overline{\textbf{s}}_b$ denote the principal symbol of the subprincipal operator of $\mathbb {L}_{g_b}$ conjugated by $Q$, the operator in Lemma 7.17. Then, there exists a choice of *a*, $\varepsilon _{{\Gamma }_{b_0}}$, $\delta _r$ and $\delta _\zeta$ sufficiently small so that

on $\mathring{\Sigma }$ and the support of $\mathring{\chi }_{\zeta }$ as defined in (128),

$$\begin{aligned} \overline{\textbf{s}}_{b,a} \in \delta _0\left( S_{{\operatorname {tan}}}^1(\Sigma ) + \sigma S_{{\operatorname {tan}}}^0(\Sigma )\right) . \end{aligned}$$

##### Proof

This follows directly by perturbing $\overline{\textbf{s}}_b$ and the smoothness of the trapped set on *a*. $\square$

## Morawetz Estimates

Recall from the discussion in Sect. 4 that the quasinormal modes (resonances) represent linear obstacles to decay. As a first step in eliminating these linear obstacles to decay, we will prove the existence of large, quasinormal-mode-free regions using a resolvent estimate.

Resolvent estimates are deeply connected to integrated local energy decay estimates, and the primary geometric obstruction to proving either in the current setting is the presence of *trapped null geodesics*. Recall that we proved the energy estimates of Sect. 6 for general strongly hyperbolic operators without relying on any particular structure on the trapped set. However, in this section, to deal with the trapped null geodesics, the structure of $\mathbb {L}_{g_b}$ at the trapped set will play a critical role. It is well known that due to the presence of trapped null geodesics, one does not expect to derive a full Morawetz estimate, but instead one that loses derivatives at the trapped set (see [2, 12, 15, 29, 40, 52]).

To capture the loss of derivatives at the trapped set, we need to define a new energy norm. This new energy norm will reflect that there are three different regions of $\Sigma$: the redshift region, the non-trapping region, and the trapping region. This division of $\Sigma$ corresponds to the fact that the geometric difficulties of trapping and superradiance are separated in physical space. On both the redshift and the non-trapping region, we will prove the desired Morawetz estimate using only physical space methods. On the trapping region, the frequency-dependent nature of trapping will lead us to use a pseudo-differentially modified divergence theorem in order to prove the desired Morawetz estimate (compare with similar work in the slowly-rotating Kerr case [56]).

**Fig. 3:**
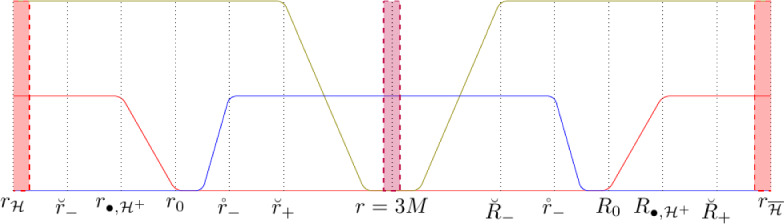
We give a rough figure depicting the cutoffs and the regions of the spacetime where geometric obstacles to decay are located. The red curve is $\dot{\chi }$, the green curve is $\breve{\chi }$, and the blue curve is $\mathring{\chi }$. The purple region is the region (in *r*) containing the trapped set ${\Gamma }_b$. The red rectangular regions on either side of the diagram denote the regions (in *r*) containing the ergoregions of the slowly-rotating Kerr-de Sitter black hole. The heights of the cutoffs in the figure are not indicative of their relative value, since all the cutoffs have values in [0, 1]. Rather, the fact that $\breve{\chi }$ is relatively large compared to $\dot{\chi }$ and $\mathring{\chi }$ reflects that when combining the various estimates, we will only add a (relatively) small amount of the redshift and trapping estimates to a (relatively) large amount of the non-trapping estimate. Observe that critically, the supports of $\dot{\chi }$ and $\mathring{\chi }$ do not intersect, but the support of $\breve{\chi }$ has a non-empty intersection with the support of both $\dot{\chi }$ and $\mathring{\chi }$

We begin by defining some auxiliary cutoff functions (Fig. 3). To this end, for fixed $b\in \mathcal {B}$, let us define

$$\begin{aligned} r_{\mathcal {H}^+}< \breve{r}_{-}< r_{\bullet , \mathcal {H}^+}<r_{0}< \mathring{r}_{-}< \breve{r}_{+}<3M< \breve{R}_{-}< \mathring{R}_{+}< R_{0}< R_{\bullet ,\overline{\mathcal {H}}^+}< \breve{R}_{+} < r_{\overline{\mathcal {H}}^+}. \end{aligned}$$

We now define the smooth physical cut-off functions

145 $$\begin{aligned} \dot{\chi }(r) {:=} \chi _{\mathcal {H}^+}(r) + \chi _{\overline{\mathcal {H}}^+}(r),\quad \breve{\chi }(r) {:=} \breve{\chi }_-(r) + \breve{\chi }_+(r), \quad \mathring{\chi }(r), \end{aligned}$$

such that

146 $$\begin{aligned} \chi _{\mathcal {H}^+}(r)= &   {\left\{ \begin{array}{ll} 1& r\in [r_{\mathcal {H}^+}, r_{\bullet , \mathcal {H}^+}]\\ 0& r\in [r_{0}, r_{\overline{\mathcal {H}}^+}] \end{array}\right. },\nonumber \\ \breve{\chi }_{ -}(r)= &   {\left\{ \begin{array}{ll} 1& r\in [r_{\mathcal {H}^+},\breve{r}_+]\\ 0& r\in (3M-\epsilon ,r_{\overline{\mathcal {H}}^+}] \end{array}\right. },\nonumber \\ \mathring{\chi }(r)= &   {\left\{ \begin{array}{ll} 1& r\in [\mathring{r}_{-}, \mathring{R}_{+}]\\ 0& r\not \in [\mathring{r}_{ -} - \epsilon , \mathring{R}_{ +}+\epsilon ] \end{array}\right. },\nonumber \\ \breve{\chi }_{+}(r)= &   {\left\{ \begin{array}{ll} 1& r\in [\breve{R}_{-}, r_{\overline{\mathcal {H}}^+}]\\ 0& r\in [r_{\mathcal {H}^+}, 3M+\epsilon ) \end{array}\right. },\nonumber \\ \chi _{\overline{\mathcal {H}}^+}(r)= &   {\left\{ \begin{array}{ll} 1& r\in [R_{\bullet , \overline{\mathcal {H}}^+}, r_{\overline{\mathcal {H}}^+}]\\ 0& r\in [r_{\mathcal {H}^+}, R_{0}] \end{array}\right. }. \end{aligned}$$

In what follows, we will denote $[r_{\mathcal {H}^+}, r_{\bullet , \mathcal {H}^+})\bigcup (R_{\bullet , \overline{\mathcal {H}}^+}, r_{\overline{\mathcal {H}}^+}]$ the redshift region, $(\breve{r}_-, \breve{r}_+)\bigcup (\breve{R}_-, \breve{R}_+)$ the nontrapping region, and $(\mathring{r}_{-}, \mathring{R}_{+})$ the trapping region.

### Definition 36

We define the *Morawetz energy norms* $Mor^k(\mathcal {D})$ and $\underline{Mor}^k(\Sigma )$ subsequently in Definition 38 as $H^k$ norms with a degeneracy at the top level of derivatives.

We define the *local energy norm* by

$$\begin{aligned} \left\Vert h\right\Vert _{LE^1(\mathcal {D})}^2 {:=}{}\left\Vert \dot{\chi }(r)h\right\Vert _{H^1(\mathcal {D})}^2 + \left\Vert \breve{\chi }(r)h\right\Vert _{H^1(\mathcal {D})}^2 + \left\Vert \mathring{\chi }(r)h\right\Vert _{Mor(\mathcal {D})}^2. \end{aligned}$$

We also have its Laplace-transformed equivalent for a function *u* on the spacelike slice $\Sigma$:

$$\begin{aligned} \begin{aligned} \left\Vert u\right\Vert _{\underline{LE}^{1}(\Sigma )}^2{:=}{}&\left\Vert \dot{\chi }(r)u\right\Vert _{\underline{H}^{1}_\sigma (\Sigma )}^2 + \left\Vert \breve{\chi }(r)u\right\Vert _{\underline{H}^{1}_\sigma (\Sigma )}^2 + \left\Vert \mathring{\chi }(r)u\right\Vert _{\underline{Mor}(\Sigma )}^2. \end{aligned} \end{aligned}$$

We can likewise define the higher-order $LE^k$ spaces:

$$\begin{aligned} LE^k(\mathcal {D})&{:=}\left\{ h: \mathcal {K}^\alpha h\in LE^1(\mathcal {D}), \left\lvert \alpha \right\rvert \le k-1\right\} ,\\ \underline{LE}^{k}(\Sigma )&{:=}\left\{ h: \mathcal {K}^\alpha h\in \underline{LE}^{1}(\Sigma ), \left\lvert \alpha \right\rvert \le k-1\right\} . \end{aligned}$$

Using this combined local energy norm, we are now ready to state the main theorem of this section.

### Theorem 8.1

Let $g_b$ be a fixed slowly-rotating Kerr-de Sitter background, and $u\in H^k(\Sigma )$. Then, for $k_0$ as defined in (79), there exists some ${\boldsymbol{\alpha }}>0$, $C_0>0$ such that for $k>k_0$,

147 $$\begin{aligned} \left\Vert u\right\Vert _{\underline{LE}^{k}(\Sigma )} \lesssim \left\Vert \widehat{\mathbb {L}}_{g_b}(\sigma )u\right\Vert _{\underline{H}^{k-1}_\sigma (\Sigma )}, \qquad {\text {if }} \Im \sigma =-{\boldsymbol{\alpha }},{\text { or }} \Im \sigma >-{\boldsymbol{\alpha }}, \left\lvert \sigma \right\rvert \ge C_0, \end{aligned}$$

for all *u* where the norms on both sides are finite.

Moreover, by adjusting the value of ${\boldsymbol{\alpha }}$ as necessary, the only poles of $\widehat{\mathbb {L}}_{g_b}(\sigma )^{-1}$ which satisfy $\Im \sigma > -{\boldsymbol{\alpha }}$ in fact also satisfy $\Im \sigma \ge 0$.

### Remark 27

The proof of Theorem 8.1 relies heavily on the fact that $\mathbb {L}_{g_b}$ is strongly hyperbolic, and the fact that we have good estimates for the scalar wave operator on the Schwarzschild-de Sitter background $g_{b_0}$. Indeed, the proofs in the ensuing section should be thought of as perturbations of the arguments that can be used in the $b = b_0 = (M, 0)$ case.

As alluded to by the construction of the cutoff functions at the beginning of the section, we divide the proof of Theorem 8.1 into three parts, corresponding to the redshift region, the nontrapping region, and the trapping region. We prove a resolvent estimate separately for solutions supported in each of these regions in Sects. 8.1, 8.2, 8.3, and 8.4 before showing that they can be appropriately combined to yield a resolvent estimate on the whole spacetime in Sect. 8.5.

The main idea behind proving Theorem 8.1 will be to first prove a Morawetz estimate for functions *h* with appropriate support, using well-chosen vectorfield multipliers (*X*, *q*, *m*) and the divergence theorem in (24). We then pass from the Morawetz estimate to a resolvent estimate using the following basic outline. Let us assume that we have chosen (*X*, *q*, *m*) appropriately so as to arrive at the following inequality^17^

$$\begin{aligned} {\int _{\breve{\Sigma }_{t_*}}J^{X,q,m}[h]\cdot n_{\breve{\Sigma }_{t_*}}}\Bigg \vert _{t_*=0}^{t_*={T_*}} + \int _{\mathcal {D}}K^{X,q,m}[h] \lesssim \left\Vert \mathbb {L}_{g_b}h\right\Vert _{L^2(\mathcal {D})}^2 + \left\Vert h\right\Vert _{L^2(\mathcal {D})}^2, \end{aligned}$$

where we will impose conditions on $J^{X,q,m}[h]$ and $K^{X,q,m}[h]$ subsequently. Differentiating by $\partial _{t_*}$, we then have that

148 $$\begin{aligned} \partial _{t_*}\int _{\breve{\Sigma }_{t_*}}J^{X,q,m}[h]\cdot n_{\breve{\Sigma }_{t_*}} + \int _{\breve{\Sigma }_{t_*}}K^{X,q,m}[h]\,\sqrt{A} \lesssim \left\Vert \mathbb {L}_{g_b}h\right\Vert _{\underline{L}^2(\breve{\Sigma }_{t_*})}^2 + \left\Vert h\right\Vert _{\underline{L}^2(\breve{\Sigma }_{t_*})}^2. \end{aligned}$$

Substituting in $h=e^{-\mathbbm {i}\sigma t_*}u(x)$, we see that (148) reduces to

$$\begin{aligned}&2\Im \sigma \int _{\breve{\Sigma }_{t_*}}J^{X,q,m}\left[ e^{-\mathbbm {i}\sigma t_*}u\right] \cdot n_{\breve{\Sigma }_{t_*}} + \int _{\breve{\Sigma }_{t_*}}K^{X,q,m}\left[ e^{-\mathbbm {i}\sigma t_*}u\right] \,\sqrt{A}\\ \lesssim {}&\left\Vert \mathbb {L}_{g_b}e^{-\mathbbm {i}\sigma t_*}u\right\Vert _{\underline{L}^2(\breve{\Sigma }_{t_*})}^2 + \left\Vert e^{-\mathbbm {i}\sigma t_*}u\right\Vert _{\underline{L}^2(\breve{\Sigma }_{t_*})}^2. \end{aligned}$$

Multiplying both sides by $\left\lvert e^{\mathbbm {i}\sigma t_*}\right\rvert$ eliminates any $t_*$-dependency in the equation, so that

149 $$\begin{aligned} \begin{aligned}&2\Im \sigma \int _{\breve{\Sigma }_{t_*}}\widehat{J}(\sigma )^{X,q,m}\left[ u\right] \cdot n_{\breve{\Sigma }_{t_*}} + \int _{\breve{\Sigma }_{t_*}}\widehat{K}(\sigma )^{X,q,m}\left[ u\right] \,\sqrt{A}\\ \lesssim {}&\left\Vert \widehat{\mathbb {L}}_{g_b}(\sigma )u\right\Vert _{\underline{L}^2(\breve{\Sigma }_{t_*})}^2 + \left\Vert u\right\Vert _{\underline{L}^2(\breve{\Sigma }_{t_*})}^2. \end{aligned} \end{aligned}$$

To reduce to a desired resolvent estimate, we proceed in two steps.

1. We will choose (*X*, *q*, *m*) so that
  $$\begin{aligned} K^{X,q,m}[h] \ge 0, \end{aligned}$$
  which implies that
  150 $$\begin{aligned} \widehat{K}(\sigma )^{X,q,m}\left[ u\right] \ge 0, \end{aligned}$$
  and such that for $\textbf{M}\ge \Im \sigma \ge -{\boldsymbol{\alpha }}$,
  151 $$\begin{aligned} 4\left\lvert \Im \sigma \widehat{J}(\sigma )^{X,q,m}\left[ u\right] \cdot n_{\breve{\Sigma }_{t_*}}\right\rvert < \widehat{K}(\sigma )^{X,q,m}\left[ u\right] \,\sqrt{A} + \left\lvert u\right\rvert ^2 \end{aligned}$$
  Equations (150) and (151) then imply that the left-hand side of (149) is positive (up to a lower-order term), and we have
  152 $$\begin{aligned} \int _{\breve{\Sigma }_{t_*}}\widehat{K}(\sigma )^{X,q,m}\left[ u\right] \,\sqrt{A} \lesssim \left\Vert \widehat{\mathbb {L}}_{g_b}(\sigma )u\right\Vert _{\underline{L}^2(\breve{\Sigma }_{t_*})}^2 + \left\Vert u\right\Vert _{\underline{L}^2(\breve{\Sigma }_{t_*})}^2. \end{aligned}$$
2. To absorb the lower-order term $\left\Vert u\right\Vert _{L^2(\breve{\Sigma }_{t_*})}$ on the right-hand side of (149), we will use the high-frequency condition in the resolvent estimates $\left\lvert \sigma \right\rvert \ge C_0$ for some $C_0$ sufficiently large. If (*X*, *q*, *m*) are chosen in such a manner that
  $$\begin{aligned} \left\lvert \sigma u\right\rvert ^2 \lesssim \widehat{K}(\sigma )^{X,q,m}[u], \end{aligned}$$
  then for sufficiently large $C_0$, we have that the $L^2$ term on the right-hand side of (152) will be absorbed into the left-hand side, and we are left with exactly the desired resolvent estimate subject to the conditions $\textbf{M}\ge \Im \sigma \ge -{\boldsymbol{\alpha }}$ and $\left\lvert \sigma \right\rvert \ge C_0$ for some $C_0$ sufficiently large.

### Remark 28

In the trapping case of Sect. 8.4.2, it is not as direct to prove the desired resolvent estimate, due to the degeneracy at the top level of derivatives in $K^{X,q,m}[h]$, and we will in fact have to prove two separate resolvent estimates, one on $\left\lvert \Im \sigma \right\rvert \le {\boldsymbol{\alpha }}$, and one on $\Im \sigma > \frac{{\boldsymbol{\alpha }}}{2}$, but the core ideas remain the same.

### Remark 29

The application of Morawetz estimates to functions ${h} =e^{-\mathbbm {i}\sigma t_*}u$ is the main tool for deriving the desired resolvent estimates. The choice ${h} =e^{-\mathbbm {i}\sigma t_*}u$ allows us to compare the integrals over the final and initial time, $2\Im \sigma \int _{\breve{\Sigma }_{t_*}}\widehat{J}(\sigma )^{X,q,m}\left[ u\right] \cdot n_{\Sigma }$, with the bulk term $\int _{\breve{\Sigma }_{t_*}}\widehat{K}(\sigma )^{X,q,m}\left[ u\right] \,\sqrt{A}$. Provided the bulk term is sufficiently large with respect to the boundary term (either by a good choice of multiplier, or by some restrictions on $\Im \sigma$), the boundary terms can then be directly absorbed into the bulk (recall the discussion before (152)). This should be contrasted with the typical approach in the vectorfield method, which is to use a Killing energy estimate to control the boundary terms.

### Redshift Region

#### Theorem 8.2

Let *g* be a slowly rotating Kerr-de Sitter metric, and define

153 $$\begin{aligned} \dot{\Sigma }{:=} \Sigma \left( [r_{\mathcal {H}^+},r_{\bullet ,\mathcal {H}^+}]\right) \bigcup \Sigma \left( [r_{\bullet ,\overline{\mathcal {H}}^+},r_{\overline{\mathcal {H}}^+}]\right) . \end{aligned}$$

Then there exists a choice of $r_{\bullet , \mathcal {H}^+}, r_{\bullet , \overline{\mathcal {H}}^+}$

and some ${\boldsymbol{\alpha }}, C_0 >0$ such that for $u:\Sigma \rightarrow \mathbb {C}$ compactly supported in $\dot{\Sigma }$ and for $k>k_0$, where $k_0$ is as defined in (79),

154 $$\begin{aligned} \left\Vert u\right\Vert _{\underline{H}^{k}_\sigma (\dot{\Sigma })} \lesssim \left\Vert \widehat{\mathbb {L}}_{g_b}(\sigma )u\right\Vert _{\underline{H}^{k-1}_\sigma (\dot{\Sigma })}, \qquad {\text {if }}\Im \sigma \ge -{\boldsymbol{\alpha }},{\text { and }} \left\lvert \sigma \right\rvert \ge C_0. \end{aligned}$$

#### Proof

We consider (100) with $\varepsilon _{\textbf{N}}$ and $\epsilon$ fixed such that

$$\begin{aligned} \varepsilon _{\textbf{N}} < \frac{1}{4}\max _{\mathcal {H}= \mathcal {H}^+, \overline{\mathcal {H}}^+}\kappa _{\mathcal {H}},\qquad \epsilon = \frac{1}{4}\max _{\mathcal {H}= \mathcal {H}^+, \overline{\mathcal {H}}^+}\kappa _{\mathcal {H}}, \end{aligned}$$

we have that for *h* supported on $\mathcal {D}$,

$$\begin{aligned} \partial _{t_*}\mathcal {E}(t_*)[{h}] - \max _{\mathcal {H}=\mathcal {H}^+, \overline{\mathcal {H}}^+}\left( \textbf{s}_{\mathbb {L}}[\mathcal {H}]-\frac{1}{2}\kappa _{\mathcal {H}}\right) \mathcal {E}(t_*)[{h}] \lesssim \left\Vert \mathbb {L}_{g_b}{h}\right\Vert _{\underline{L}^2(\Sigma _{t_*})}^2 + \left\Vert h\right\Vert _{\underline{L}^2(\Sigma _{t_*})}^2. \end{aligned}$$

To proceed from this version of the redshift estimate to a resolvent estimate, we consider $h=e^{-\mathbbm {i}\sigma t_*}u$,

$$\begin{aligned}&\left( 2\Im \sigma - \max _{\mathcal {H}=\mathcal {H}^+, \overline{\mathcal {H}}^+}\left( \textbf{s}_{\mathbb {L}_{g_b}}[\mathcal {H}]-\frac{1}{2}\kappa _{\mathcal {H}}\right) \right) \mathcal {E}(t_*)[e^{-\mathbbm {i}\sigma t_*}u]\\ \lesssim {}&\left\Vert \mathbb {L}_{g_b} e^{-\mathbbm {i}\sigma t_*}u \right\Vert _{\underline{L}^2(\Sigma _{t_*})}^2 + \left\Vert e^{-\mathbbm {i}\sigma t_*}u\right\Vert _{\underline{L}^2(\Sigma _{t_*})}^2. \end{aligned}$$

If

155 $$\begin{aligned} 2\Im \sigma >\max _{\mathcal {H}=\mathcal {H}^+, \overline{\mathcal {H}}^+}\left( \textbf{s}_{\mathbb {L}_{g_b}}[\mathcal {H}]-\frac{1}{2}\kappa _{\mathcal {H}}\right) , \end{aligned}$$

then the left-hand side of the above equation is positive. Multiplying both sides by $e^{-2\Im \sigma t_*}$ to cancel out any $t_*$ dependency, we then have using 1 of Theorem 6.3 that

$$\begin{aligned}&\left( 2\Im \sigma - \max _{\mathcal {H}=\mathcal {H}^+, \overline{\mathcal {H}}^+}\left( \textbf{s}_{\mathbb {L}_{g_b}}[\mathcal {H}]-\frac{1}{2}\kappa _{\mathcal {H}}\right) \right) \left( \left\Vert u\right\Vert _{\underline{H}^{1}(\Sigma )}^2 + \left\Vert \sigma u\right\Vert _{\underline{L}^2(\Sigma )}^2 \right) \\ \lesssim {}&\left\Vert \widehat{\mathbb {L}}_{g_b}(\sigma )u \right\Vert _{\underline{L}^2(\Sigma )}^2 + \left\Vert u\right\Vert _{\underline{L}^2(\Sigma _{t_*})}^2. \end{aligned}$$

Then, if (155) is satisfied, and if $\left\lvert \sigma \right\rvert > C_0$ for some $C_0$ large enough, the $\left\Vert u\right\Vert _{\underline{L}^2(\Sigma _{t_*})}$ term on the right-hand side can be absorbed by the $\left\Vert \sigma u\right\Vert _{\underline{L}^2(\Sigma )}$ term on the left-hand side, yielding the desired resolvent estimate (154) in the $k=1$ case.

To prove the higher-order estimates, we can repeat the derivation of (8.1) for $\textbf{L}^{(k)}_{g_b}$ in place of $\mathbb {L}$, where $\textbf{L}^{(k)}_{g_b}$ is the strongly hyperbolic operator constructed from $\mathbb {L}_{g_b}$ after *k* commutations with the vectorfields $\{\mathcal {K}_i\}_{i=1}^N$, as in Theorem 6.6. Thus, to conclude, we need only show that for *k* sufficiently large, in particular larger than $k_0$, $\max _{\mathcal {H}=\mathcal {H}^+, \overline{\mathcal {H}}^+}\left( \textbf{s}_{\textbf{L}^{(k)}_{g_b}}[\mathcal {H}]-\frac{1}{2}\kappa _{\mathcal {H}}\right) < 0$. But precisely from Theorem 6.6, we know that

$$\begin{aligned} \textbf{s}_{\textbf{L}^{(k)}}[\mathcal {H}] = \textbf{s}_{\mathbb {L}_{g_b}}[\mathcal {H}] - 2k\kappa _{\mathcal {H}}. \end{aligned}$$

Thus, for $$k > k_0$$,

$$\begin{aligned} \max _{\mathcal {H}=\mathcal {H}^+, \overline{\mathcal {H}}^+}\left( \textbf{s}_{\textbf{L}^{(k)}_{g_b}}[\mathcal {H}]-\frac{1}{2}\kappa _{\mathcal {H}}\right) < 0, \end{aligned}$$

as desired. $$\square $$

### Nontrapping Region

The resolvent estimates away from trapping will be proven using the following vectorfield

156 $$\begin{aligned} \breve{X}= \breve{f}(r)\widehat{R}, \qquad \breve{f}(r) = e^{\breve{C}(r-3M)^2}(r-3M)\Delta , \end{aligned}$$

where $\widehat{R}$ is as defined in (18).

#### Theorem 8.3

Let *g* be a fixed slowly-rotating Kerr-de Sitter background.

We recall the notation

157 $$\begin{aligned} \mathcal {D}{:=}\mathbb {R}^+_{t_*}\times \breve{\Sigma },\qquad \breve{\Sigma }{:=}\Sigma \left( (\breve{r}_-,\breve{r}_+)\bigcup (\breve{R}_-, \breve{R}_+)\right) . \end{aligned}$$

Then for $k_0$ the threshold regularity level defined in (79), there exists ${\boldsymbol{\alpha }}>0$, $C_0>0$ such that for $u:\Sigma \rightarrow \mathbb {C}$ compactly supported on $\breve{\Sigma }$ and for $k>k_0$

158 $$\begin{aligned} \left\Vert u\right\Vert _{\underline{H}^{k}_\sigma (\breve{\Sigma })} \lesssim \left\Vert \widehat{\mathbb {L}}(\sigma )u\right\Vert _{\underline{H}^{k-1}_\sigma (\breve{\Sigma })},\qquad {\text {if }}\textbf{M}\ge \Im \sigma \ge -{\boldsymbol{\alpha }},\quad \left\lvert \sigma \right\rvert \ge C_0. \end{aligned}$$

Similar to the approach taken to proving the resolvent estimates in Theorem 8.2 and Corollary 6.4, we will first use an energy estimate and then convert that energy estimate into a resolvent estimate.

There are two components to proving the desired Morawetz estimate. The main difficulty will be the positive bulk term, which will come down to choosing $\breve{C}$ as well as the choice of $\breve{q}$. After making these choices we will have to handle the boundary terms. In the literature, the typical method for handling boundary terms is to add a large amount of the standard $\partial _{t_*}$-energy estimate to the Morawetz estimate to prove an energy-Morawetz estimate.

We will handle the boundary terms in a different way, proving (158) by absorbing the boundary terms directly into positivity of the bulk term. We begin with the bulk term.

#### Lemma 8.4

Fix a constant $C>0$ and a slowly-rotating Kerr-de Sitter metric *g*. Then there exists a choice of $\breve{C}$ and $\breve{q}$ so that for $\breve{X}$ defined as in (156) and for *h* compactly supported on $\breve{\Sigma }$, there exists some $C_1$ such that

159 $$\begin{aligned} K^{\breve{X}, \breve{q}, 0}[{h}] - \Re \left[ \breve{X}{h} \cdot \textbf{S}[\overline{{h}}]\right] > C\left\lvert \nabla {h}\right\rvert ^2 - C_1\left\lvert h\right\rvert ^2. \end{aligned}$$

#### Remark 30

When proving a high-frequency Morawetz estimate for the scalar wave operator, the positivity requirement is merely that

$$\begin{aligned} K^{\breve{X},\breve{q}, 0}[{h}]>c\left\lvert \nabla {h}\right\rvert ^2 - C_1\left\lvert h\right\rvert ^2 \end{aligned}$$

for some $c>0$. Our requirement here that *C* can be any positive real number, and that, in particular, it can be *arbitrarily large* is due to the presence of the subprincipal operator, which does not carry a sign, and for which we do not have any control. In its absence, or in the case that it has a good sign, such strong positivity in the Morawetz estimate would not be necessary. To accommodate the subprincipal term with no sign, we need to prove (159) with $C>\sup \textbf{S}$.

#### Proof

We first calculate the deformation tensor associated to the vectorfield multiplier $\breve{f}(r)\widehat{R}$,

$$\begin{aligned} 2^{\breve{X}}\pi ^{\alpha \beta } = \partial _r\breve{f}(r)g^{r(\beta }\widehat{R}^{\alpha )} + \breve{f}(r)g^{r(\beta }\widehat{R}'^{\alpha )} - \breve{f}(r)\widehat{R}g^{\alpha \beta }, \end{aligned}$$

where we recall the definition of $\widehat{R}$ and $\widehat{R}'$ in (18). Then

$$\begin{aligned} \begin{aligned} K^{\breve{X}, 0, 0}[{h}] ={}&\frac{1}{2}\left( \Re \left( \left( \breve{f}(r)\widehat{R}'{h} + \partial _r\breve{f}(r)\widehat{R}{h}\right) \cdot g^{r\gamma }\partial _\gamma \overline{{h}}\right) - \breve{f}(r)(\widehat{R}g)^{\alpha \beta }\partial _{(\alpha }{h}\cdot \partial _{\beta )}\overline{h}\right) \\&- (\nabla _g\cdot \breve{X})\partial ^\gamma {h}\cdot \partial _\gamma \overline{h}. \end{aligned} \end{aligned}$$

Let us rewrite this as

160 $$\begin{aligned} \begin{aligned} \rho ^2K^{\breve{X}, 0, 0}[{h}] ={}&\frac{1}{2}\Re \left( \breve{f}(r)\widehat{R}'{h}\cdot (\rho ^2g)^{r\gamma }\partial _\gamma \overline{{h}} +\partial _r\breve{f}(r)\widehat{R}{h}\cdot (\rho ^2g)^{r\gamma }\partial _\gamma \overline{{h}}\right) \\&- \frac{1}{2}\left( \breve{f}(r)(\widehat{R}(\rho ^2g))^{\alpha \beta }\partial _{(\alpha }{h}\cdot \partial _{\beta )}\overline{{h}}\right) - \left( \rho ^2\nabla _g\cdot \breve{X}- \rho \breve{f}(r) \widehat{R}\rho \right) \partial ^\gamma {h}\cdot \partial _\gamma \overline{{h}}. \end{aligned} \end{aligned}$$

We first ensure that the first two terms of equation (160) is positive. The third term will be handled by the choice of Lagrangian correction.

Using the definition of $\breve{f}(r)$ in equation (156), we have that

161 $$\begin{aligned}&\Re \left( \breve{f}(r)\widehat{R}'{h}\cdot (\rho ^2g)^{r\gamma }\partial _\gamma \overline{{h}} +\partial _r\breve{f}(r)\widehat{R}{h}\cdot (\rho ^2g)^{r\gamma }\partial _\gamma \overline{{h}}\right) - \breve{f}(r)(\widehat{R}(\rho ^2g))^{\alpha \beta }\partial _{(\alpha }{h}\cdot \partial _{\beta )}\overline{{h}}\nonumber \\ ={}&e^{\breve{C}(r-3M)^2}\left( (2\breve{C}(r-3M)^2+1)\Delta ^2\left\lvert \widehat{R}h\right\rvert ^2 - \Re \left( \frac{r-3M}{\Delta }\widehat{T}{h} \cdot \left( \partial _r\Delta \widehat{T}\overline{{h}} - 2 \Delta \widehat{T}'\overline{{h}} \right) \right) \right) , \end{aligned}$$

where we recall the definition of $\widehat{T}, \widehat{T}'$ from (18).

Since $\left\lvert r-3M\right\rvert$ and $\Delta$ are bounded from below on $\breve{\Sigma }$, there exists some choice of $\breve{C}$ sufficiently large so that on $\breve{\Sigma }$,

$$\begin{aligned} 2(\breve{C}(r-3M)^2 + 1)\Delta ^2 > 0. \end{aligned}$$

Thus the coefficient of $\left\lvert \widehat{R}{h}\right\rvert ^2$ in (161) is positive. We move on to dealing with the last term in (161). Observe that

$$\begin{aligned} r^2\partial _r\Delta _{b_0} - 4r\Delta _{b_0} = -2r^2(r-3M), \qquad \widehat{T}' = \frac{2}{r}\widehat{T}+ O(a)(\partial _{t_0}, \partial _{\varphi _0}). \end{aligned}$$

As a result, we can write

$$\begin{aligned} \begin{aligned}&-(r-3M)\Delta ^{-1}\widehat{T}{h}\cdot \left( \partial _r \Delta \widehat{T}\overline{{h}} - 2\Delta \widehat{T}'\overline{{h}}\right) \\ ={}&2(r-3M)^2\Delta ^{-1}\left\lvert \widehat{T}{h}\right\rvert ^2 + \Delta ^{-1}O(a)\left( \left\lvert \partial _{t_0}{h}\right\rvert ^2 + \left\lvert \partial _{\varphi _0}{h}\right\rvert ^2\right) . \end{aligned} \end{aligned}$$

Now, in order to handle the terms on the second line of equation (160), we define

$$\begin{aligned} \breve{q}_0 = \nabla _g\cdot \breve{X}+ \rho ^{-1} \breve{f}(r) \widehat{R}\rho . \end{aligned}$$

Recall that we have

$$\begin{aligned} K^{0, \breve{q}_0, 0}[{h}] = \breve{q}_0\partial ^\gamma {h}\cdot \partial _\gamma \overline{{h}} - \frac{1}{2}\nabla ^\gamma \partial _\gamma \breve{q}_0\left\lvert {h}\right\rvert ^2. \end{aligned}$$

As a result,

$$\begin{aligned} \begin{aligned} 2K^{\breve{X}, \breve{q}_0, 0}[{h}] \!=\!{}&\rho ^{-2}e^{\breve{C}(r-3M)^2}\left( \left( 2\breve{C}(r\!-\!3M)^2\!+\! 1\right) \left\lvert \Delta \widehat{R}{h}\right\rvert ^2 \!+\!\Delta ^{-1}(r\!-\!3M)^2\left\lvert \widehat{T}{h}\right\rvert ^2\right) \\&\!+\!\rho ^{-2}e^{\breve{C}(r-3M)^2}\left( O(a)\left( \left\lvert \partial _{t_0}{h}\right\rvert ^2 \!+\! \left\lvert \partial _{\varphi _0}{h}\right\rvert ^2\right) \right) \!-\! \nabla ^\alpha \partial _\alpha \breve{q}_{0} \left\lvert {h}\right\rvert ^2. \end{aligned} \end{aligned}$$

By choosing $\breve{C}$ sufficiently large, we can in particular guarantee that there exists some $C_2$ such that

$$\begin{aligned} K^{\breve{X}, \breve{q}_0, 0}[{h}] \ge C\left\lvert \widehat{R}{h}\right\rvert ^2 + C\left\lvert \widehat{T}{h}\right\rvert ^2 + O(a)\left( \left\lvert \partial _{t_0}{h}\right\rvert ^2 + \left\lvert \partial _{\varphi _0}{h}\right\rvert ^2\right) - C_2\left\lvert {h}\right\rvert ^2. \end{aligned}$$

There are two remaining issues. The first is the *O*(*a*) errors. The second is that we do not have control over all the derivatives of *h*. For instance, control over $\widehat{R}{h}$ and $\widehat{T}{h}$ do not yield any control over $\widehat{\mathcal {O}}{h}$. We fix these two problems at the same time by “borrowing” some positivity from $\widehat{R}h$ and $\widehat{T}h$ using the Lagrangian correction. To this end, consider

162 $$\begin{aligned} \breve{q}= \breve{q}_0 + \breve{q}_1,\qquad \breve{q}_1 =\delta _1e^{\breve{C}(r-3M)^2}. \end{aligned}$$

Up to lower order terms,

163 $$\begin{aligned} \begin{aligned}&\rho ^2K^{\breve{X}, \breve{q},0}[h]\\ \!=\!{}&e^{\breve{C}(r\!-\!3M)^2}\left( \delta _1\rho ^2\left\lvert \widehat{\mathcal {O}}{h}\right\rvert ^2 \!+\! \left( 2\breve{C}(r\!-\!3M)^2 + 1\right) \left\lvert \Delta \widehat{R}{h}\right\rvert ^2 \!+\!\frac{(r-3M)^2-\delta _1}{\Delta }\left\lvert \widehat{T}{h}\right\rvert ^2 \right) \\&\!+\! e^{\breve{C}(r-3M)^2}\left( \delta _1\Delta \left\lvert \widehat{R}{h}\right\rvert ^2 \!+\! O(a)\left( \left\lvert \partial _{t_0}{h}\right\rvert ^2 \!+\! \left\lvert \partial _{\varphi _0}{h}\right\rvert ^2\right) \right) , \end{aligned} \end{aligned}$$

where we recall the definition of $\widehat{\mathcal {O}}h$ in (20).

For sufficiently large $\breve{C}$ and sufficiently small $\delta _1$, *a*, we have that $(r-3M)^2-\delta _1>0$ on $\breve{\Sigma }$, and the *O*(*a*) errors are entirely controlled, so there exists some $\delta >0$ and $C>0$ such that

164 $$\begin{aligned} K^{\breve{X}, \breve{q},0}[h] > \delta e^{\breve{C}(r-3M)^2}\left( \breve{C}\left\lvert \widehat{R}{h}\right\rvert ^2 + \left\lvert \widehat{T}h\right\rvert ^2 + \left\lvert \widehat{\mathcal {O}}h\right\rvert ^2\right) - C\left\lvert {h}\right\rvert ^2. \end{aligned}$$

We next consider the contribution of the subprincipal operator. Using Cauchy-Schwarz, we observe that

165 $$\begin{aligned} \left\lvert \textbf{S}[h]\cdot \breve{X}\overline{h}\right\rvert \le e^{\breve{C}(r-3M)^2}\left( \epsilon \left\lvert \nabla h\right\rvert ^2 + \epsilon ^{-1}\left\lvert (r-3M)\widehat{R}h\right\rvert ^2\right) . \end{aligned}$$

Thus for sufficiently small $\epsilon$ and sufficiently large $\breve{C}$, we have that up to lower-order terms,

$$\begin{aligned} \left\lvert \textbf{S}[h]\cdot \breve{X}\overline{h}\right\rvert \le \frac{1}{2}K^{\breve{X}, \breve{q},0}[h]. \end{aligned}$$

Thus, we have that for *h* supported on $\breve{\Sigma }$, up to lower-order terms,

$$\begin{aligned} K^{\breve{X}, \breve{q},0}[h] - \Re \left[ \textbf{S}[{h}]\cdot \breve{X}\overline{{h}}\right] \ge e^{\breve{C}(r-3M)^2}\frac{\delta }{2}\left\lvert \nabla {h}\right\rvert ^2. \ \end{aligned}$$

We conclude the proof of Lemma 8.4 by further increasing $\breve{C}$ as necessary so that $\frac{\delta }{2}e^{\breve{C}\delta _r^2}>C$. $\square$

We now show how to close the proof of Theorem 8.3 in the $k=1$ case given the positivity of the bulk term in Lemma 8.4.

***Proof of Theorem*** 8.3***for*** $k=1$ For *h* supported on $\mathcal {D}$ as defined in (157), we define the Morawetz energy on a $t_*$-constant spacelike slice by

$$\begin{aligned} \breve{\mathfrak {E}}(t_*)[{h}] = \int _{\breve{\Sigma }_{t_*}}J^{\breve{X}, \breve{q}, 0}[{h}] \cdot n_{\breve{\Sigma }}. \end{aligned}$$

It is clear upon inspection that

$$\begin{aligned} \breve{\mathfrak {E}}(t_*)[{h}] \lesssim \left\Vert {h}\right\Vert _{\underline{H}^{1}(\breve{\Sigma })}^2 + \left\Vert \textbf{T}h\right\Vert _{\underline{L}^2(\breve{\Sigma })}^2 , \end{aligned}$$

where we emphasize that unlike $\mathcal {E}(t_*)$, $\mathfrak {E}(t_*)[h]$ does not necessarily have a sign. We now apply (24) to $J^{\breve{X}, \breve{q}, 0}[{h}]$, to see that

$$\begin{aligned} \nabla \cdot J^{\breve{X}, \breve{q}, 0}[{h}] = \Re \left[ \breve{X}\overline{{h}}\cdot \Box _{g}{h} \right] + K^{\breve{X}, \breve{q}, 0}[{h}]. \end{aligned}$$

Since we have assumed *h* with compact support on $\breve{\Sigma }$, *h* vanishes on the horizons, and

$$\begin{aligned} \int _{\mathcal {H}^+\bigcap \Sigma _{t_*}} J^{\breve{X}, \breve{q}, 0}[{h}]\cdot n_{\mathcal {H}^+} = \int _{\overline{\mathcal {H}}^+\bigcap \Sigma _{t_*}} J^{\breve{X}, \breve{q}, 0}[{h}] \cdot n_{\overline{\mathcal {H}}^+} = 0. \end{aligned}$$

Applying the divergence equation (24),

166 $$\begin{aligned}&\left. \breve{\mathfrak {E}}(t_*)[{h}]\right| _{t_*=0}^{t_*= {T_*}} + \int _{\mathcal {D}}K^{\breve{X}, \breve{q}, 0}[{h}] - \Re \left\langle \breve{X}h , \textbf{S}[{h}]\right\rangle _{L^2(\mathcal {D})}\nonumber \\ ={}&-\Re \left\langle (\breve{X}+\breve{q}) {h}, \mathbb {L}{h}\right\rangle _{L^2(\mathcal {D})} + \Re \left\langle (\breve{X}+\breve{q}) {h}, \textbf{V}{h}\right\rangle _{L^2(\mathcal {D})} + \Re \left\langle \breve{q}{h},\textbf{S}[{h}]\right\rangle _{L^2(\mathcal {D})}. \end{aligned}$$

Note that at this point, a Morawetz estimate would follow immediately

from Lemma 8.4 and a Cauchy-Schwarz argument to control the lower-order term on the right-hand

To prove the resolvent estimate in (158), we differentiate both sides of equation (166) by $\partial _{t_*}$.

167 $$\begin{aligned} \partial _{t_*}\breve{\mathfrak {E}}(t_*)[h] + \int _{\breve{\Sigma }_{t_*}}K^{\breve{X}, \breve{q}, 0}[{h}]\,\sqrt{A} - \Re \left\langle \breve{X}h , \textbf{S}[{h}]\right\rangle _{\underline{L}^2(\breve{\Sigma }_{t_*})} \lesssim \left\Vert \mathbb {L}_{g_b}h\right\Vert _{\underline{L}^2(\breve{\Sigma }_{t_*})}^2 + \left\Vert h\right\Vert _{\underline{L}^2(\breve{\Sigma }_{t_*})}^2. \end{aligned}$$

We now show how to absorb the sign-less principal-level boundary term into the bulk term. To this end, let $u\in \underline{H}^{1}(\breve{\Sigma }_{t_*})$, and consider ${h} = e^{-\mathbbm {i}\sigma t_*}u(x)$, so that

$$\begin{aligned} \partial _{t_*}\breve{\mathfrak {E}}(t_*)[h] = 2\Im \sigma \breve{\mathfrak {E}}(t_*)[h]. \end{aligned}$$

Recalling that

$$\begin{aligned} J^{\breve{X}, \breve{q}, 0}[{h}]\cdot n_{\Sigma } = \breve{X}{h} \cdot n_{\Sigma }\overline{{h}} + \breve{q}{h} \cdot n_{\Sigma }\overline{{h}}, \end{aligned}$$

we choose $\breve{C}$ sufficiently large using Lemma 8.4 such thatSubstituting this back into (167), we have that for $h=e^{-\mathbbm {i}\sigma t_*}u$,

$$\begin{aligned} \left\Vert h\right\Vert _{\overline{H}^{1}(\breve{\Sigma }_{t_*})}^2 \lesssim \left\Vert \mathbb {L}_{g_b}h\right\Vert _{\underline{L}^2(\breve{\Sigma }_{t_*})}^2 + \left\Vert h\right\Vert _{\underline{L}^2(\breve{\Sigma }_{t_*})}^2. \end{aligned}$$

Multiplying both sides by $e^{-2\Im \sigma t_*}$ to remove any $t_*$-dependency, we then have that

$$\begin{aligned} \left\Vert u\right\Vert _{\underline{H}^{1}_\sigma (\breve{\Sigma }_{t_*})} \lesssim \left\Vert \widehat{\mathbb {L}}(\sigma )u\right\Vert _{\underline{L}^2(\breve{\Sigma }_{t_*})} + \left\Vert u\right\Vert _{\underline{L}^2(\breve{\Sigma }_{t_*})}. \end{aligned}$$

Recalling from Definition 14 that

$$\begin{aligned} \left\Vert u\right\Vert _{\underline{H}^{1}_\sigma (\breve{\Sigma })}^2 = \left\Vert u\right\Vert _{\underline{H}^{1}(\breve{\Sigma })}^2 + \left\Vert \sigma u\right\Vert _{\underline{L}^2(\breve{\Sigma })}^2, \end{aligned}$$

we see that for $\left\lvert \sigma \right\rvert$ sufficiently large, the $\left\Vert u\right\Vert _{L^2(\breve{\Sigma }_{t_*})}$ term on the right-hand side can be absorbed into the $\left\Vert \sigma u\right\Vert _{L^2(\breve{\Sigma }_{t_*})}$ left-hand side to conclude. $\square$

Now let us prove Theorem 8.3 for higher-order *k*. To do so, we commute derivatives with the gauge-fixed linearized Einstein operator to derive a higher order positive bulk term. The rest of the proof is identical to the $k=0$ version.

***Proof of Theorem*** 8.3***for*** $k>1$ Let us define

168 $$\begin{aligned} \mathbb {L}= \frac{1}{A}D_{t_*}^2 + P_1D_{t_*} + P_2, \end{aligned}$$

where $P_i$ are order-*i* differential operators on $\breve{\Sigma }$.

The main idea will be to use the fact that $\textbf{T}$ commutes with $\mathbb {L}$, and that $P_2$ is elliptic on $\breve{\Sigma }$. We prove (158) for the $k=2$ case. The higher-order cases follow from induction.

First, from (158) with $k=1$ have that for ${h} = e^{-\mathbbm {i}\sigma t_*}u$, spatially supported on $\mathcal {D}$,

169 $$\begin{aligned} \left\Vert \sigma u\right\Vert _{\underline{H}^{1}_\sigma (\breve{\Sigma })} \lesssim \left\Vert \sigma \widehat{\mathbb {L}}(\sigma )u\right\Vert _{\underline{L}^2(\breve{\Sigma })}. \end{aligned}$$

Rewriting (168), we thus have that

$$\begin{aligned} P_2u = \widehat{\mathbb {L}}(\sigma )u - \sigma P_1 u - \sigma ^2 Au. \end{aligned}$$

Since $P_2$ is elliptic on $\breve{\Sigma }$, using standard elliptic estimates and (169), we can conclude that

$$\begin{aligned} \left\Vert u\right\Vert _{\underline{H}^{2}_\sigma (\breve{\Sigma })} \lesssim \left\Vert \widehat{\mathbb {L}}(\sigma )u\right\Vert _{\underline{H}^{1}_\sigma (\breve{\Sigma })}, \end{aligned}$$

yielding the $k=2$ case. Repeating the elliptic estimates and commutations with $\textbf{T}$ as above yields the subsequent higher-order estimates. $\square$

### Nontrapped Near $r=3M$

In the previous section, we proved the resolvent estimates away from $r=3M$, using the vectorfield method to take advantage of the non-trapping nature of the region. In this section, we restrict ourselves to a physical neighborhood of $r=3M$ containing ${\Gamma }_b$, but microlocalize away from ${\Gamma }_b$, so that we are still able to prove a Morawetz estimate despite being in a neighborhood of $r=3M$. As in the previous section, we fix $g=g_b$ and drop the subscripts in what follows.

Recall from Lemma 7.8 that

$$\begin{aligned} {\Gamma }= \left\{ (t, r, \theta , \varphi ;\sigma , \xi , \eta _{\theta }, \eta _{\varphi }): p= 0, H_{p}r = 0, r = \mathring{r}(\sigma ,\eta _{\varphi }) \right\} . \end{aligned}$$

In what follows, we work on a small neighborhood of $r=3M$ where the $(t_*, r, \theta , {\varphi _*})$ coordinates are identical to the standard Boyer-Lindquist coordinates. As such, we use Boyer-Lindquist coordinates in what follows, and denote

$$\begin{aligned} (\textbf{x}, \zeta ) = (t,r,\theta ,\varphi ; \sigma , \xi , \eta _{\theta }, \eta _{\varphi }). \end{aligned}$$

In this section then we will prove a Morawetz estimate for functions supported physically neighborhood with Fourier transform supported in a frequency neighborhood away from the trapped set.

#### Theorem 8.5

Let

$$\begin{aligned} \mathcal {D}{:=}\mathbb {R}^+_{t_*} \times \mathring{\Sigma },\qquad \mathring{\Sigma }{:=} (\mathring{r}_-, \mathring{R}_+)\times \mathbb {S}^2, \end{aligned}$$

where we assume $3M-\mathring{r}_- \le \delta _r, \mathring{R}_+ - 3M \le \delta _r$ for some $\delta _r$ sufficiently small. Then let $u\in H^{k}(\Sigma )$ be compactly supported in $\mathring{\Sigma }$ such that its Fourier transform $\hat{u}(\xi , \eta ), (\xi ,\eta )\in T^{*}\mathbb {R}^3$ is supported on the region $\frac{\left\lvert \xi \right\rvert ^2}{\left\lvert \eta \right\rvert ^2} \ge \delta _\zeta$ for some $\delta _\zeta$ sufficiently small,then there exist constants ${\boldsymbol{\alpha }}, C_0 > 0$ such that we have the following resolvent estimate

170 $$\begin{aligned} \left\Vert u\right\Vert _{\underline{H}^{k}_\sigma (\mathring{\Sigma })} \lesssim \left\Vert \widehat{\mathbb {L}}(\sigma )u\right\Vert _{\underline{H}^{k-1}_\sigma (\mathring{\Sigma })}, \qquad {\text {if }} -{\boldsymbol{\alpha }}\le \Im \sigma \le \textbf{M},\quad {\text {and }} \left\lvert \sigma \right\rvert >C_0. \end{aligned}$$

Due to the introduction of the frequency cutoff away from ${\Gamma }_b$, we will pursue the resolvent estimates here in frequency space instead of using the physical space calculations of Sect. 8.2. To this end, we will show that $K^{X,q, 0}[h]$, which has principal symbol $\frac{1}{2\mathbbm {i}}H_{p}X$ from Lemma 7.10, is elliptic.

#### Lemma 8.6

Let

171 $$\begin{aligned} \check{X}= \check{f}(r)\partial _r,\qquad \check{f}(r) = e^{\check{C}(r-3M)^2}(r-3M)\Delta , \end{aligned}$$

and define

172 $$\begin{aligned} \begin{aligned} \check{\varkappa }&{:=} \check{f}(r)\xi = \frac{1}{2}e^{\check{C}(r-3M)^2}(r-3M)H_{p}r,\\ \check{\mathfrak {q}}&{:=} -e^{\check{C}(r-3M)^2}\frac{1}{C_\xi }\left( 2\check{C}(r-3M)^2+1\right) , \end{aligned} \end{aligned}$$

where $C_\xi$ is the positive constant from Lemma 7.9, and $\check{C}>0$ is some positive constant to be determined.

Fix some $C>0$. Then, there exists some $\check{f}(r)$ and $\check{C}>0$, such that for $\check{X}$ and $\check{\varkappa }$ as defined in (171), for any first order symbol $s \in S^1(\mathcal {M})$ satisfying $\left\lvert s\right\rvert \le C\left\lvert \zeta \right\rvert$, there exists some $C_{\zeta }$ sufficiently large such that

$$\begin{aligned} H_{p}\check{\varkappa }- s\check{\varkappa }+ \check{\mathfrak {q}}p\gtrsim \left\lvert \zeta \right\rvert ^2,\qquad \left\lvert \zeta \right\rvert >C_{\zeta }. \end{aligned}$$

#### Remark 31

Note that the inclusion of the $s \check{\varkappa }$ term with potentially large *s* reflects that we will absorb both the contributions of the subprincipal term and the boundary terms in the Morawetz estimate via the positive bulk term generated by the principal wave component of the operator. This is entirely analogous to the argument taken in the previous section via the vectorfield argument.

#### Proof

We can calculate that

$$\begin{aligned} H_{p}\check{\varkappa }= e^{\check{C}(r-3M)^2}\left( \left( 2\check{C}(r-3M)^2+1\right) (H_{p}r)^2 + (r-3M)(H_{p}^2r) \right) , \end{aligned}$$

and hence,

$$\begin{aligned} H_{p}\check{\varkappa }\!-\! s\check{\varkappa }\!+\! \check{\mathfrak {q}}p\!=\!{}&e^{\check{C}(r-3M)^2}\left( \left( 2\check{C}(r-3M)^2+1\right) (H_{p}r)^2 \!+\! (r-3M)(H_{p}^2r)\right) \\&\!+\!e^{\check{C}(r-3M)^2}\left( \!- \!s (r-3M)H_{p} \!-\! \frac{1}{C_\zeta }\left( 2\check{C}(r-3M)^2 + 1\right) p\right) . \end{aligned}$$

The main idea of the lemma will be to use the fact that on the support of $\breve{\chi }_{\zeta }$, we have already shown in Lemma 7.9 that for some $C_\xi >0$ sufficiently large, $C_\xi (H_{p}r)^2-p$ is elliptic. This will compensate for the fact that we are in a physical neighborhood of ${\Gamma }$.

First observe that by Cauchy-Schwarz,

$$\begin{aligned} \begin{aligned} \left\lvert (r-3M)H_{p}^2r\right\rvert&\le \epsilon (H_{p}^2r) + \frac{(r-3M)^2}{\epsilon }(H_{p}^2r),\\ \left\lvert (r-3M)s H_{p}r\right\rvert&\le \epsilon C\left\lvert \zeta \right\rvert ^2 + \frac{(r-3M)^2}{\epsilon }(H_{p}r)^2. \end{aligned} \end{aligned}$$

Since $(H_{p}r)^2 - C_\xi ^{-1}p$ is elliptic, there exists a choice of $\epsilon$ sufficiently small so that

$$\begin{aligned} \epsilon \left\lvert s\right\rvert ^2 +\epsilon \left\lvert H_{p}^2r\right\rvert \le (H_{p}r)^2- C_\xi ^{-1}p. \end{aligned}$$

Choosing $\epsilon$ sufficiently small as above, and $\check{C}> \frac{2}{\epsilon }$ sufficiently large, we have that

$$\begin{aligned} (2\check{C}(r-3M)^2+1)\left( (H_{p}r)^2-C_\xi ^{-1}p\right) + (r-3M)(H_{p}^2r) - \frac{1}{2}(r-3M)s H_{p}r \end{aligned}$$

is elliptic, as desired. $\square$

We now show how to prove the Morawetz estimate in Theorem 8.5 for the case $k=1$, given the ellipticity of the bulk term proven in Lemma 8.6.

***Proof of Theorem*** 8.5***for*** k=1 We first prove Theorem 8.5 for $k=1$. The main difficulty, as was the case for the previous resolvent estimates investigated, is showing that the boundary term that arises after using the integration-by-parts (or divergence theorem) argument can be absorbed by the bulk term. Define, for $\check{\mathfrak {q}}$ as constructed in (172),

$$\begin{aligned} \check{q}= \frac{1}{2}\nabla _g\cdot \check{X}+ \check{\mathfrak {q}}, \end{aligned}$$

so that using (129),

$$\begin{aligned} K^{\check{X}, \check{q}, 0}[h] = (\textbf{k}^{\check{X}, \check{q}}_{(2)})^{\alpha \beta }\nabla _{(\alpha }h\cdot \nabla _{\beta )}\overline{h} - \frac{1}{2}\nabla ^\alpha \partial _\alpha \check{q}\left\lvert h\right\rvert ^2, \end{aligned}$$

where

$$\begin{aligned} 2(\textbf{k}^{\check{X}, \check{q}}_{(2)})^{\alpha \beta }\zeta _\alpha \zeta _\beta = H_{p}\check{\varkappa }+ \check{\mathfrak {q}}p. \end{aligned}$$

We now also define the relevant Morawetz energy on the $t_*$-constant hypersurfaces for this section

$$\begin{aligned} \check{\mathfrak {E}}(t_*)[{h}] = \int _{\Sigma _{t_*}}J^{\check{X}, \check{q}, 0}[{h}]\cdot n_{\Sigma _{t_*}} . \end{aligned}$$

Applying the divergence relation in Corollary 2.6, we have that

173 $$\begin{aligned} \!-\!\Re \left\langle \mathbb {L}{h}, (\check{X}\!+\!\check{q}){h}\right\rangle _{L^2(\mathcal {D})} \!=\!{}&\int _{\mathcal {D}}K^{\check{X}, \check{q}, 0}[{h}] \!+\! \left. \check{\mathfrak {E}}(t_*)[{h}]\right| _{t_*= 0}^{t_*= {T_*}} \!-\! \Re \left\langle \textbf{S}[{h}], (\check{X}\!+\!\check{q}){h}\right\rangle _{L^2(\mathcal {D})} \nonumber \\&\!-\! \Re \left\langle \textbf{V}{h}, (\check{X}\!+\!\check{q}){h}\right\rangle _{L^2(\mathcal {D})}. \end{aligned}$$

We now show the resolvent estimate (170)^18^. Consider *u*(*x*) such that $\hat{u}(\xi , \eta )$ is supported on the region $\frac{\left\lvert \xi \right\rvert ^2}{\left\lvert \eta \right\rvert ^2} \ge \delta _\zeta$ for some $\delta _\zeta$ sufficiently small, and let $h = e^{-\mathbbm {i}\sigma t_*}u$. Then,

$$\begin{aligned} \partial _{t_*}\check{\mathfrak {E}}(t_*)[{h}] = 2\Im \sigma \check{\mathfrak {E}}(t_*)[{h}]. \end{aligned}$$

Recall that

$$\begin{aligned} J^{\check{X}, \check{q}, 0}[{h}]\cdot n_{\Sigma } = \check{X}{h} \cdot n_{\Sigma }\overline{{h}} + \check{q}{h} \cdot n_{\Sigma }\overline{{h}}. \end{aligned}$$

Then from Lemma 8.6, we can choose $\check{C}$ sufficiently large so that for any $-{\boldsymbol{\alpha }}< \Im \sigma < \textbf{M}$,Plugging this back into (173), and applying Cauchy-Schwarz, we have that for $h = e^{\mathbbm {i}\sigma t_*}u$,

$$\begin{aligned} \left\Vert h\right\Vert _{\overline{H}^{1}(\mathring{\Sigma })}^2 \lesssim \left\Vert \mathbb {L}_{g_b}h\right\Vert _{\underline{L}^2(\mathring{\Sigma })}^2 + \left\Vert h\right\Vert _{\underline{L}^2(\mathring{\Sigma })}^2. \end{aligned}$$

Multiplying both sides of the equation by $e^{-2\Im \sigma t_*}$ to get rid of any $t_*$ dependency, we have that

$$\begin{aligned} \left\Vert u\right\Vert _{\underline{H}^{1}_\sigma (\mathring{\Sigma }_{t_*})} \lesssim \left\Vert \widehat{\mathbb {L}}(\sigma )u\right\Vert _{\underline{L}^2(\mathring{\Sigma }_{t_*})} + \left\Vert u\right\Vert _{\underline{L}^2(\mathring{\Sigma }_{t_*})}. \end{aligned}$$

Recalling from Definition 14 that

$$\begin{aligned} \left\Vert u\right\Vert _{\underline{H}^{1}_\sigma (\mathring{\Sigma })}^2 = \left\Vert u\right\Vert _{\underline{H}^{1}(\mathring{\Sigma })}^2 + \left\Vert \sigma u\right\Vert _{\underline{L}^2(\mathring{\Sigma })}^2, \end{aligned}$$

we see that for $\left\lvert \sigma \right\rvert$ sufficiently large, the $\left\Vert u\right\Vert _{L^2(\mathring{\Sigma }_{t_*})}$ term on the right-hand side can be absorbed into the $\left\Vert \sigma u\right\Vert _{L^2(\mathring{\Sigma }_{t_*})}$ left-hand side to conclude. $\square$

To prove Theorem 8.5 for higher-order *k*, we again rely on a commutation with $\partial _{t_*}$ and an elliptic argument, taking advantage of the fact that the trapped set and the ergoregions in the slowly-rotating cases are physically separated.

***Proof of Theorem*** 8.5***for*** k>1 We prove Theorem 8.5 for $k=2$. The $k>2$ case follows similarly. Reflecting the fact that $\textbf{T}$ is Killing and commutes with $\mathbb {L}$, we have from (170) with $k=1$ that for $u\in \underline{H}^{k}(\mathring{\Sigma })$ supported on $\mathring{\Sigma }$,

174 $$\begin{aligned} \left\Vert \sigma u\right\Vert _{\underline{H}^{1}(\mathring{\Sigma })} \lesssim \left\Vert \sigma \widehat{\mathbb {L}}(\sigma )u\right\Vert _{\underline{L}^2(\mathring{\Sigma })}. \end{aligned}$$

Then, using that $\mathbb {L}= P_2 + P_1D_{t_*} + \frac{1}{A} D^2_{t_*}$, we have that for $u\in \underline{H}^{2}(\mathring{\Sigma })$,

$$\begin{aligned} P_2u = \widehat{\mathbb {L}}(\sigma ) u - \sigma P_1u - \sigma ^2 Au. \end{aligned}$$

Recall that $P_2$ is elliptic away from the ergoregions. Since we are considering a region supported away from the ergoregions, we can apply a standard elliptic estimate to see that

175 $$\begin{aligned} \left\Vert u\right\Vert _{\underline{H}^{2}(\mathring{\Sigma })} \lesssim \left\Vert \widehat{\mathbb {L}}(\sigma )u\right\Vert _{\underline{H}^{1}_{\sigma }(\mathring{\Sigma })}. \end{aligned}$$

Combining equations (174) and (175) allows us to conclude. $\square$

### Trapping Region

We now microlocalize to the trapped set in a neighborhood of $r=3M$. While in the previous sections we were able to prove Morawetz estimates that controlled the full $H^k$ norm of solutions *h*, we will be unable to do so in this section due to the presence of trapping. Instead, we define new norms that account for trapping by degenerating exactly on the trapped set. Also, we use the frequency analysis in Sect. 7 to account for the frequency-dependent nature of the trapped set in Kerr-de Sitter. The pseudo-differential operators introduced should be compared to the very similar pseudo-differential operators used by Tataru and Tohaneanu in [56] to prove a Morawetz estimate for solutions to the scalar wave on a Kerr background.

Throughout this section we will let $g_{b_0}=g(M,0)$ be a fixed Schwarzschild-de Sitter metric and $g_b=g(M,a)$ be a nearby Kerr-de Sitter metric. As we discussed in Sect. 7.2, for any $\delta _r>0$, there exists a neighborhood of $b_0$ of black hole parameters $\mathcal {B}$ such that for all $g_b$ Kerr-de Sitter backgrounds with $b\in \mathcal {B}$, the trapped set ${\Gamma }_b$ lies entirely within $\left\{ \left\lvert r-3M\right\rvert <\delta _r\right\}$. In addition on $\left\{ \left\lvert r-3M\right\rvert <\delta _r\right\}$, the Kerr-star coordinates $(t_*, r, \theta , {\varphi _*})$ reduce to the Boyer-Lindquist coordinates $(t, r, \theta , \varphi )$, and we will use the Boyer-Lindquist coordinates in the remainder of this section.

We begin with a proof of the trapped high-frequency Morawetz estimate on Schwarzschild-de Sitter in Sect. 8.4.1, and show how to use the basic spectral gap for the scalar wave equation on Schwarzschild-de Sitter to prove a spectral gap for the gauge-fixed linearized Einstein operator linearized around a nearby Kerr-de Sitter metric.

Throughout this section, we will denote

176 $$\begin{aligned} \mathcal {D}{:=}\mathbb {R}^+_{t_*} \times \mathring{\Sigma },\qquad \mathring{\Sigma }{:=} \Sigma (\mathring{r}_-\le r\le \mathring{R}_+), \end{aligned}$$

where we assume $3M-\mathring{r}_- \le \delta _r, \mathring{R}_+ - 3M \le \delta _r$, so that for sufficiently slowly-rotating Kerr-de Sitter metrics $g_b$, the trapped set ${\Gamma }_b$ lies entirely within $\mathcal {D}$.

#### Scalar Wave Operator on Schwarzschild-de Sitter

Before we prove the high-frequency Morawetz estimates for the gauge-fixed linearized Einstein equations on Kerr-de Sitter, let us first review the proof of high-frequency Morawetz estimates in a neighborhood of the photon sphere for the scalar wave equation on Schwarzschild-de Sitter. This will serve as the basis upon which we add the pseudo-differential modification of the divergence theorem in Sect. 7.3 to prove a high-frequency Morawetz estimate for the gauge-fixed linearized Einstein operator.

Recall from Lemma 7.7 that for $g_{b_0}$ a Schwarzschild-de Sitter metric, the trapped set is contained exactly at the photon sphere $r=3M$,

$$\begin{aligned} {\Gamma }_{b_0} = \left\{ (t,r,\omega ;\sigma , \xi , \eta ): r=3M, \xi =0, p_{b_0}=0\right\} . \end{aligned}$$

We then define the following norm177where $\mathring{\Sigma }$ is as defined in (176).

##### Remark 32

We remark that compared to the standard Morawetz norm defined on Schwarzschild (see for instance equation (1.11) in [46]), we differ by a power of *r*. This plays no role in our analysis as *r* is both bounded above and below on the static region of Schwarzschild-de Sitter.

We also define the following auxiliary, non-coercive norm that will be used in the subsequent proof of the desired Morawetz estimate,

$$\begin{aligned} \mathring{\mathfrak {E}}_{b_0}(t)[{h}] = \int _{\mathring{\Sigma }_t}J^{X_{b_0}, q_{b_0}, 0}[{h}]\cdot n_{\mathring{\Sigma }_t}, \end{aligned}$$

where $X_{b_0}$ and $q_{b_0}$ will be defined in what follows.

With the desired norm in hand, we now state the desired resolvent estimate for the scalar wave operator in Schwarzschild-de Sitter:

##### Proposition 8.7

Let

$$\begin{aligned} \mathcal {D}{:=}\mathbb {R}^+_t\times \mathring{\Sigma },\qquad \mathring{\Sigma }= (3M-\delta _r, 3M+\delta _r), \end{aligned}$$

for some sufficiently small $\delta _r$. Then if *u* is a sufficiently smooth function with compact support in $\mathring{\Sigma }$, then there exists some ${\boldsymbol{\alpha }}_{0}>0$ such that

178 $$\begin{aligned} \left\Vert u\right\Vert _{Mor_{b_0}(\mathring{\Sigma })} \lesssim \left\Vert \widehat{\Box }_{g_{b_{0}}}(\sigma )u\right\Vert _{\underline{L}^2(\mathring{\Sigma })},\qquad \Im \sigma \ge -{\boldsymbol{\alpha }}_0. \end{aligned}$$

We will prove the result with a purely physical argument, emphasizing that the pseudo-differential nature of the subsequent arguments in Kerr-de Sitter reflects the frequency-dependent nature of trapping in Kerr-de Sitter and the microlocal smallness we need at the level of the subprincipal operator of $\mathbb {L}_{g_b}$.

There are two components to the proof of Proposition 8.7. First, we prove the resolvent estimate (178) for $\left\lvert \Im \sigma \right\rvert \le {\boldsymbol{\alpha }}_0$ for some ${\boldsymbol{\alpha }}_0>0$, and then we prove the resolvent estimate for $\Im \sigma > \frac{{\boldsymbol{\alpha }}_0}{2}$. These two steps correspond to using a Morawetz estimate and a basic energy estimate respectively.

The main lemma is as follows.

##### Lemma 8.8

For $\delta _r$ sufficiently small there exists:

1. a smooth vectorfield
  179 $$\begin{aligned} X_{b_0} = f_{b_0}(r)\partial _r, \end{aligned}$$
  where $f_{b_0}(r)$ is bounded near $r=3M$. In particular, we will choose
  180 $$\begin{aligned} f_{b_0}(r)= \frac{(r-3M)\mu _{b_0}}{r^2}; \end{aligned}$$
2. a smooth function $q_{b_0}$; such that for *h* supported in $\mathcal {D}{:=}\mathbb {R}^+_t\times \mathring{\Sigma }$, $\mathring{\Sigma }= (3M-\delta _r, 3M+\delta _r)$,

##### Proof

We can first calculate that for $X_{b_0} = f_{b_0}(r)\partial _r$ as defined above in (180),181Since $\mu _{b_0} > 0$ on $\mathring{\Sigma }$, the first two terms on the right-hand side of equation (181) are clearly positive. There are two remaining issues. The first is a treatment of the third term in equation (181), which does not have a sign. The second is that we wish for $K^{X_{b_0}, 0, 0}[{h}] = \mathbb {T}[{h}]\cdot ^{({X_{b_0}})}\pi$ to be coercive in all the derivatives of *h*, not just $\partial _r$ and . The key to resolving both of these issues will be to make an appropriate choice of $q_{b_0}$. Consider

$$\begin{aligned} q_0 {:=} \frac{\mu _{b_0}}{2r^2}, \end{aligned}$$

so that

$$\begin{aligned} K^{0, q_0, 0}[h] = \frac{\mu _{b_0}}{2r^2}g_{b_0}^{\gamma \delta }\partial _\gamma {h}\cdot \partial _\delta \overline{h} - \frac{1}{2}\nabla ^\alpha \partial _\alpha q_0 \left\lvert h\right\rvert ^2, \end{aligned}$$

where we can directly calculate that

$$\begin{aligned} -\frac{1}{2}\nabla ^\alpha \partial _\alpha q_0(3M) = \frac{1-9M^2\Lambda }{243M^4}>0. \end{aligned}$$

As a result, for $\delta _r$ sufficiently small,

$$\begin{aligned} -\frac{1}{2}\nabla ^\alpha \partial _\alpha q_0 > 0 \end{aligned}$$

on all of $\mathring{\Sigma }$.

The addition of $K^{0, q_0, 0}[{h}]$ to $K^{X, 0, 0}[{h}]$ will eliminate the third term on the right-hand side of equation (181) and introduce the coerciveness of the $L^2$ norm, that is,To solve the issue of coerciveness of the $\partial _t$ derivatives, we borrow positivity from the angular and radial derivatives in $K^{X_{b_0}, q_0,0}[{h}]$ via the Lagrangian correction. Consider

182 $$\begin{aligned} q_1 {:=} -\delta _1\frac{(r-3M)^2}{r^4}. \end{aligned}$$

ThenCombining the above calculations, we find that defining

183 $$\begin{aligned} q_{b_0}{:=} q_0 + q_1, \end{aligned}$$

we can calculate184where

$$\begin{aligned} \alpha _{b_0}^2&= \mu _{b_0}^{-1}\frac{(r-3M)^2}{r^4},\\ \beta _{b_0}^2&= \mu _{b_0}^2\left( \frac{1}{r^2} - \frac{2(r-3M)}{r^3} - \delta _1\mu _{b_0}^{-1}\frac{(r-3M)^2}{r^4}\right) , \\ \gamma _{b_0}^2&= \nabla ^\alpha \partial _\alpha (q_0+q_1), \end{aligned}$$

where we have used that $q_1 = O(\delta _1)$ to write $\gamma _{b_0}^2$ as a positive function on $\mathcal {D}$. $\square$

Having shown that $K^{X_{b_0}, q_{b_0},0}[{h}]$ generates a non-negative bulk term, we can now move onto the proof of Proposition 8.7.

##### Proof of Proposition 8.7

Using the divergence theorem in Corollary 2.6, we see that for *h* supported in a neighborhood of $r=3M$,

185 $$\begin{aligned} \partial _t\mathring{\mathfrak {E}}(t)[{h}] + \int _{\mathring{\Sigma }}K^{X_{b_0}, q_{b_0},0}[{h}]\,\sqrt{A} = -\Re \int _{\mathring{\Sigma }} \Box _{g_{b_{0}}}{h}\cdot \left( X_{b_0}+q_{b_0}\right) \overline{{h}}\,\sqrt{A}. \end{aligned}$$

Using Lemma 8.8 and the Cauchy-Schwarz inequality, we then have that

$$\begin{aligned} \partial _t\mathring{\mathfrak {E}}(t)[{h}] + \left\Vert {h}\right\Vert _{Mor_{b_0}(\mathring{\Sigma })}^2 \lesssim \left\Vert \Box _{g_{b_{0}}}h\right\Vert _{\underline{L}^2(\mathring{\Sigma })}^2. \end{aligned}$$

To prove the resolvent estimate in (178), we consider ${h} = e^{-\mathbbm {i}\sigma t}u(x)$, where *u* is supported in a neighborhood of $r=3M$. Furthermore, recall that

186 $$\begin{aligned} \mathring{\mathfrak {E}}(t)[{h}] = \int _{\mathring{\Sigma }}J^{X_{b_0}, q_{b_0},0}[h]\cdot n_{\mathring{\Sigma }} = \Re \int _{\mathring{\Sigma }}f_{b_0}(r)\partial _r{h}\cdot n_{\mathring{\Sigma }}\overline{h} + \Re \int _{\mathring{\Sigma }}q_{b_0}{h}\cdot n_{\mathring{\Sigma }}\overline{h}. \end{aligned}$$

Using the fact that $q_{b_0}$ is a smooth stationary function on $\mathring{\Sigma }$, we use integration by parts to write that

$$\begin{aligned} \begin{aligned} \left\langle \partial _r(3M-r){q}_{b_0}{h},n_{\mathring{\Sigma }}{h}\right\rangle _{L^2(\mathring{\Sigma })} ={}&\left\langle (r-3M){q}_{b_0}\partial _r{h},n_{\mathring{\Sigma }}{h}\right\rangle _{L^2(\mathring{\Sigma })} + \left\langle (r-3M){q}_{b_0}{h},n_{\mathring{\Sigma }}\partial _r{h}\right\rangle _{L^2(\mathring{\Sigma })}\\&+ Err[{h}], \end{aligned} \end{aligned}$$

where *Err*[*h*] consists of lower-order terms satisfying the estimate

$$\begin{aligned} \left\lvert Err[{h}]\right\rvert \lesssim \left\Vert (r-3M)\nabla {h}\right\Vert _{L^2(\mathring{\Sigma })}^2 + \left\Vert \partial _r{h}\right\Vert _{L^2(\mathring{\Sigma })}^2 + \left\Vert {h}\right\Vert _{L^2(\mathring{\Sigma })}^2. \end{aligned}$$

Recall that we assumed that *h* could be written as $h=e^{-\mathbbm {i}\sigma t}u$, it is clear that $\partial _th = -\mathbbm {i}\sigma h$. Thus, we can apply Cauchy-Schwarz to control

$$\begin{aligned} \left\lvert \left\langle (r-3M){q}_{b_0}\partial _r{h},n_{\mathring{\Sigma }}{h}\right\rangle _{L^2(\mathring{\Sigma })}\right\rvert + \left\lvert \left\langle (r-3M){q}_{b_0}{h},n_{\mathring{\Sigma }}\partial _r{h}\right\rangle _{L^2(\mathring{\Sigma })}\right\rvert \lesssim \left\Vert h\right\Vert _{Mor_{b_0}(\mathring{\Sigma })}^2. \end{aligned}$$

We can also apply Cauchy-Schwarz to control

$$\begin{aligned} \int _{\mathring{\Sigma }}\left\lvert f_{b_0}(r)\partial _rh\cdot n_{\mathring{\Sigma }}\overline{h}\right\rvert \lesssim \left\Vert h\right\Vert _{Mor_{b_0}(\mathring{\Sigma })}^2. \end{aligned}$$

Having controlled each of the terms in (186), we can write that

$$\begin{aligned} \left\lvert \mathring{\mathfrak {E}}_{b_0}(t)[{h}]\right\rvert \lesssim \left\Vert h\right\Vert _{Mor_{b_0}(\mathring{\Sigma })}^2. \end{aligned}$$

Recalling that for $h=e^{-\mathbbm {i}\sigma t}$, $\partial _th = -\mathbbm {i}\sigma h$, we see that for any $\delta >0$, there exists a choice of ${\boldsymbol{\alpha }}_0>0$ such that for $\left\lvert \Im \sigma \right\rvert <{\boldsymbol{\alpha }}_0$,

$$\begin{aligned} 2\Im \sigma \mathring{\mathfrak {E}}(t)[{h}] + \int _{\mathring{\Sigma }}K^{X_{b_0}, q_{b_0},0}[{h}]\,\sqrt{A} \gtrsim \left\Vert h\right\Vert _{\mathfrak {E}(\mathring{\Sigma })}. \end{aligned}$$

Plugging this back into (185), we have from Cauchy-Schwarz that

$$\begin{aligned} \left\Vert h\right\Vert _{\mathfrak {E}(\mathring{\Sigma })} \lesssim \left\Vert \Box _{g_{b_{0}}}{h}\right\Vert _{\underline{L}^2(\mathring{\Sigma })}^2 + \left\Vert h\right\Vert _{\underline{L}^2(\mathring{\Sigma })}^2. \end{aligned}$$

Multiplying both sides by $e^{2\Im \sigma t}$ to remove any *t*-dependency, and using Cauchy-Schwarz, we have that

187 $$\begin{aligned} \left\Vert u\right\Vert _{Mor_{b_0}(\mathring{\Sigma })} \lesssim \left\Vert \widehat{\Box }_{g_{b_{0}}}(\sigma )u\right\Vert _{\underline{L}^2(\mathring{\Sigma })}, \qquad \left\lvert \Im \sigma \right\rvert \le {\boldsymbol{\alpha }}_{0}. \end{aligned}$$

It then remains to prove the resolvent estimate (178) with $\Im {\sigma }>{\boldsymbol{\alpha }}_{0}$. In the case of the scalar wave operator on Schwarzschild-de Sitter, there is no subprincipal component to consider, and the Killing energy is conserved. Thus, using the equivalent of a naive Gronwall-type energy estimate, we have that for any $\epsilon >0$, there exists a constant $C(\epsilon )>0$ such that

$$\begin{aligned} \partial _{t}E(t)[{h}] \le C(\epsilon )\left\Vert \Box _{g_{b_{0}}}h\right\Vert _{\underline{L}^2(\mathring{\Sigma })}^2 + \epsilon E(t)[h]. \end{aligned}$$

Since *h* is supported near ${\Gamma }_{b_0}$ (in particular, it is supported away from both $\mathcal {H}^+$ and $\overline{\mathcal {H}}^+$), the Killing energy norm of *h* controls all derivatives of *h*. Thus, when we plug in ${h} = e^{-\mathbbm {i}\sigma t}u(x)$ for some *u* that is compactly supported on a small neighborhood of ${\Gamma }_{b_0}$ (and in particular, compactly supported away from the event horizon and the cosmological horizon), we have

$$\begin{aligned} \partial _tE(t)[{h}] \gtrsim \Im \sigma \left\Vert {h}\right\Vert _{\underline{H}^{1}_\sigma (\mathring{\Sigma })}^2. \end{aligned}$$

As a result, for $\Im \sigma >\frac{{\boldsymbol{\alpha }}_0}{2}$, and sufficiently small $\epsilon$,

188 $$\begin{aligned} \left\Vert u\right\Vert _{H^1_{\sigma }(\mathring{\Sigma })} \lesssim \left\Vert \widehat{\Box }_{g_{b_{0}}}(\sigma )u\right\Vert _{\underline{L}^2(\mathring{\Sigma })}, \qquad \Im \sigma >\frac{{\boldsymbol{\alpha }}_0}{2}. \end{aligned}$$

Combining the estimates in (187) and (188) yields a resolvent estimate on the entire half-plane $\Im \sigma \ge -{\boldsymbol{\alpha }}_0$ as desired, concluding the proof of Proposition 8.7. $\square$

#### Morawetz Estimate Near $r=3M$ for the Gauge-Fixed Linearized Einstein Operator

We now turn to the problem of proving resolvent estimates for the gauge-fixed linearized Einstein operator in Kerr-de Sitter. We will first need to define the relevant norms. To capture the idea that trapping is a feature of the characteristic set, for $r\in (3M - \delta _r, 3M+\delta _r)$, we factor

$$\begin{aligned} p_b(r, \theta ; \sigma , \xi , \eta _{\theta }, \eta _{\varphi }) = g^{tt}(\sigma -\sigma _1(r,\theta ; \xi , \eta _{\theta }, \eta _{\varphi }))(\sigma -\sigma _2(r, \theta ; \xi , \eta _{\theta }, \eta _{\varphi })), \end{aligned}$$

where $\sigma _1, \sigma _2\in S_{hom}^1$ are distinct smooth symbols. On the cones $\sigma =\sigma _i$ (i.e. on the characteristic set), the symbol $r-\mathring{r}_b$ is then equal to

$$\begin{aligned} \ell _i = r-\mathring{r}_b(\sigma _i,\eta _{\varphi }) = r-3M - a \tilde{r}_b \left( \sigma _i, \eta _{\varphi }\right) . \end{aligned}$$

To use $\ell _i$, we cut off away from the singularity at frequency 0 and redefine:

$$\begin{aligned} \ell _i = r-\mathring{r}_b(\sigma _i,\eta _{\varphi }) = r-3M - a \chi _{\ge 1}\tilde{r}_b \left( \sigma _i, \eta _{\varphi }\right) \in S_{{\operatorname {tan}}}^0(\Sigma ), \end{aligned}$$

where $\chi _{\ge 1}$ is a smooth symbol such that $\chi _{\ge 1}=1$ for frequencies $\ge 2$, $\chi _{\ge 1}=0$ for frequencies $\le 1$.

The symbols $\ell _i$ can then be used to define microlocally weighted $L^2$ function spaces in a neighborhood of $r=3M$.

##### Definition 37

Let $h:\mathcal {M}\rightarrow \mathbb {C}^D$ such that $h(t_*, \cdot )$ is compactly supported on $\mathring{\Sigma }$ for all $t_*>0$, and let $\mathcal {D}$ be as defined in (176). We define

$$\begin{aligned} \begin{aligned} \left\Vert h\right\Vert _{L^2_{\ell _i}(\mathcal {D})}^2&= \left\Vert \ell _i(D, x){h}\right\Vert _{L^2(\mathcal {D})}^2. \end{aligned} \end{aligned}$$

We also define the corresponding norms over a spacelike slice.

$$\begin{aligned} \left\Vert {h}\right\Vert _{\underline{L}^2_{\ell _i}(\mathring{\Sigma })}^2&= \left\Vert \ell _i(D, x){h}\right\Vert _{\underline{L}^2(\mathring{\Sigma })}^2. \end{aligned}$$

##### Remark 33

The symbols $\ell _i$ are nonzero outside an *O*(*a*) neighborhood of 3*M*, so $L_{\ell _i}^2$ is equivalent to the $L^2$ norm outside an *O*(*a*) neighborhood of $r=3M$.

##### Definition 38

Consider $h:\mathcal {M}\rightarrow \mathbb {C}^D$ such that $h(t_*,\cdot )$ is compactly supported on $\mathring{\Sigma }$ for all $t_*\ge 0$. Then for $\mathcal {D}$ as defined in (176) we define the *local Morawetz norm* by

$$\begin{aligned} \begin{aligned} \left\Vert h\right\Vert ^2_{Mor_b(\mathcal {D})} ={}&\left\Vert \mathring{\chi }(D_t-\sigma _2(D, x))\mathring{\chi } h\right\Vert ^2_{L^2_{\ell _1}(\mathcal {D})} + \left\Vert \mathring{\chi } (D_t-\sigma _1(D, x)) \mathring{\chi }h\right\Vert ^2_{L^2_{\ell _2}(\mathcal {D})}\\&+ \left\Vert \partial _r h\right\Vert ^2_{L^2(\mathcal {D})} + \left\Vert h\right\Vert ^2_{L^2(\mathcal {D})}. \end{aligned} \end{aligned}$$

We also define the higher-order Morawetz norms

$$\begin{aligned} \left\Vert h\right\Vert _{Mor^k_b(\mathcal {D})}^2 = \sum _{\left\lvert \alpha \right\rvert \le {k-1}}\left\Vert \mathcal {K}^{\alpha }h\right\Vert _{Mor_b(\mathcal {D})}^2. \end{aligned}$$

Observe that $Mor^1_b(\mathcal {D}) = Mor_b(\mathcal {D})$. We also have the equivalent norms over the spacelike slice $\mathring{\Sigma }$,

$$\begin{aligned} \left\Vert h\right\Vert _{\underline{Mor}_b(\mathring{\Sigma })}^2&={} \left\Vert \mathring{\chi }(D_t-\sigma _2(D, x))\mathring{\chi } h\right\Vert _{\underline{L}^2_{\ell _1}(\mathring{\Sigma })}^2 + \left\Vert \mathring{\chi }(D_t-\sigma _1(D, x)) \mathring{\chi }h\right\Vert _{\underline{L}^2_{\ell _2}(\mathring{\Sigma })}^2\\&+ \left\Vert \partial _r h\right\Vert _{\underline{L}^2(\mathring{\Sigma })}^2 + \left\Vert h\right\Vert _{\underline{L}^2(\mathring{\Sigma })}^2,\\ \left\Vert h\right\Vert _{\underline{Mor}_b^k(\mathring{\Sigma })}^2&= \sum _{\left\lvert \alpha \right\rvert \le {k-1}}\left\Vert \mathcal {K}^{\alpha }h\right\Vert _{\underline{Mor}_b(\mathring{\Sigma })}^2. \end{aligned}$$

##### Remark 34

Note that for the Schwarzschild-de Sitter metric $g_{b_0}$, the Morawetz norm in Definition 38 reduces towhich agrees with our earlier definition in (177) of the Morawetz norm on Schwarzschild-de Sitter (up to the presence of cutoffs).

##### Remark 35

Observe that the local Morawetz norm is fine-tuned so that all the derivatives taken of *h* have symbols which vanish exactly on the trapped set. This will be exploited heavily in what follows to prove the desired Morawetz estimate.

We state the main theorem of this section.

##### Theorem 8.9

Let $\mathcal {D}$ and $\mathring{\Sigma }$ be as defined in (176), and *u* be a sufficiently smooth function supported in $\mathring{\Sigma }$ such that $\hat{u}(\xi , \eta )$ is supported on the region $\frac{\left\lvert \xi \right\rvert ^2}{\left\lvert \eta \right\rvert ^2} \le 2\delta _\zeta$. Then for $\delta _r, \delta _\zeta$ sufficiently small, there exists ${\boldsymbol{\alpha }}>0$, $C_0>0$, such that for $k\ge k_0$, where $k_0$ is the threshold regularity level defined in (79), such that

189 $$\begin{aligned} \left\Vert u\right\Vert _{Mor^{k}_b(\mathring{\Sigma })} \lesssim \left\Vert \widehat{\mathbb {L}}_{g_b}(\sigma )u\right\Vert _{\underline{H}^{k-1}_\sigma (\mathring{\Sigma })},\qquad {\text {if }}\Im \sigma > -{\boldsymbol{\alpha }},{\text { and }} \left\lvert \sigma \right\rvert \ge C_0; {\text { or }} \Im \sigma = -{\boldsymbol{\alpha }}. \end{aligned}$$

Like in the case for the scalar wave operator on Schwarzschild-de Sitter in Sect. 8.4.1, we will prove the resolvent estimate in (189) in two parts. We first prove the resolvent estimate for $\left\lvert \Im \sigma \right\rvert \le {\boldsymbol{\alpha }}$ for some ${\boldsymbol{\alpha }}>0$, and then we prove the resolvent estimate for $\Im \sigma > \frac{{\boldsymbol{\alpha }}}{2}$. Again like in the case for the scalar wave equation on Schwarzschild-de Sitter, these two estimates correspond to a Morawetz estimate and a $\partial _{t_*}$-energy estimate respectively.

We give a brief outline of the proof:

1. We begin with proving the resolvent estimate for $\left\lvert \Im \sigma \right\rvert \le {\boldsymbol{\alpha }}$. The bulk of the proof will be dedicated, as in the case in Sect. 8.4.1 for the scalar wave operator on Schwarzschild-de Sitter, to handling the bulk terms in the first line of (135). The main outline remains similar to the approach used in the Schwarzschild-de Sitter case and can be viewed as a perturbation of the proof in Lemma 8.8. The key idea is to extract a non-negative bulk term at the principal level that degenerates only at the trapped set. This is done in Lemma 8.11, and relies on finding suitable $X_b$, $q_b$ to extract a sum of squares expression for
  $$\begin{aligned} \frac{1}{2\mathbbm {i}}H_{p_b} \varkappa _b + p_b \mathfrak {q}_b, \end{aligned}$$
  the bulk term that comes out of the principal scalar-wave component of $\mathbb {L}_{g_b}$. To do so, we will take pseudodifferential modifications of the vectorfield multiplier $X_{b_0}$, and the Lagrangian corrector $q_{b_0}$ constructed in Lemma 8.8 that take into account the more complicated (in particular frequency-dependent) nature of trapping in Kerr-de Sitter to guarantee the desired degenerate ellipticity.
2. We will treat the remaining terms in the integration by parts argument as small perturbations of the positive bulk we obtained in the previous step. We first control in Corollary 8.13 the remaining terms at the principal level. These come from the contribution of the subprincipal symbol and the pseudo-differential conjugation, and have symbol $$\overline{\textbf{s}}_b \varkappa _b$$. It is critical here that for $$b=b_0$$, this symbol can be made arbitrarily small by an appropriate choice of $$Q$$. This allows us to continue to treat it as a small perturbation of the scalar wave operator for *b* close to $$ b_0$$.
3. We will also use the degenerate ellipticity to show that the lower-order bulk terms are appropriately controlled in Lemma 8.21. Unlike in the non-trapping regimes, here we cannot simply control these terms via a high-frequency argument. Since the ellipticity that we recover at the principal level is degenerate at ${\Gamma }_b$, we can only control degenerate lower-order terms. We will get around this difficulty by showing that we can suitably modify the lower-order terms so that they respect the aforementioned degeneracy at the cost of a derivative.
4. The final step in proving the resolvent estimate for $\left\lvert \Im \sigma \right\rvert \le {\boldsymbol{\alpha }}$ is to show that when $h=e^{-\mathbbm {i}\sigma t_*}u$, the boundary terms themselves can also be controlled by the degenerate ellipticity of the bulk term for some ${\boldsymbol{\alpha }}>0$ sufficiently small. We emphasize that in this proof, we do not appeal to energy-boundedness. In fact, the only time we do use a $\partial _{t_*}$-energy estimate is to show that the energy grows at most like $e^{\epsilon t_*}$ for $\epsilon \ll 1$. This is done in Lemma 8.22.
5. Finally, we prove a resolvent for $\left\lvert \Im \sigma \right\rvert \le {\boldsymbol{\alpha }}$. This step makes use of a naive Gronwall-based energy estimate which takes advantage of the smallness of the subprincipal symbol when microlocalized near trapping.

We divide the proof into sections as outlined above.

**Principal level bulk terms**

We first handle the bulk term rising from the principal scalar wave component of $\mathbb {L}_{g_b}$. The main degenerate positivity is the following.

##### Lemma 8.10

Let $X_{b_0}$, $q_{b_0}$ be as defined in Lemma 8.8 (specifically, as defined in (179) and (183) respectively).

Then for sufficiently small *a*, there exists some

$$\begin{aligned} \widetilde{X}\in \Psi _{{\operatorname {tan}}}^1 + \Psi _{{\operatorname {tan}}}^0\partial _{t_*},\qquad \tilde{q} \in \Psi _{{\operatorname {tan}}}^0 + \Psi _{{\operatorname {tan}}}^{-1}\partial _{t_*}, \end{aligned}$$

depending smoothly on *a* such that defining

$$\begin{aligned} X_b {:=} X_{b_0} + a\widetilde{X},\qquad q_b{:=} q_{b_0} + a\tilde{q}, \end{aligned}$$

there exists a Hermitian (with respect to the $L^2(\mathcal {D})$ inner product) operator

$$\begin{aligned} \tilde{\mathfrak {q}}_{b_0}\in (a+\delta _r)\Psi _{{\operatorname {tan}}}^0(\Sigma ), \end{aligned}$$

such that

$$\begin{aligned} \left\Vert h\right\Vert _{Mor_b(\mathcal {D})}^2 \lesssim {}&\int _{\mathcal {D}}K^{X_{b_0},q_{b_0},0}[h] + a \mathbb {K}^{\widetilde{X}, \tilde{q}}[h] - 2\Re \left\langle \overline{\textbf{S}}_b h, X_{b} h\right\rangle _{L^2(\mathcal {D})} + 2\Re \left\langle \overline{\textbf{S}}_bh, \tilde{\mathfrak {q}}_{b_0}h\right\rangle _{L^2(\mathcal {D})} \\&+ 2{\Re \left\langle \overline{\textbf{S}}_{0}h, a\widetilde{X}h\right\rangle _{L^2(\mathring{\Sigma })}}\Bigg \vert ^{t_*={T_*}}_{t_*=0} + 2{\Re \left\langle \overline{\textbf{S}}_bh, a\widetilde{X}_0h\right\rangle _{L^2(\mathring{\Sigma })}}\Bigg \vert ^{t_*={T_*}}_{t_*=0}, \end{aligned}$$

The main key to proving Lemma 8.10 will be an appropriate degenerate positivity in principal-order bulk terms for an appropriately chosen multiplier. This stems from the fact that the operator $\mathbb {L}_{g_b}$ is strongly hyperbolic, and the positive bulk term gained from commuting with the scalar wave operator. The proof of the lemma below follows closely that of Tataru and Tohaneanu in the Kerr setting in Lemma 4.3 of [56].

##### Lemma 8.11

Let

$$\begin{aligned} \varkappa _{b_0} = \mathbbm {i}f_{b_0}\xi ,\qquad \mathfrak {q}_{b_0} = q_{b_0} - \frac{1}{2}\nabla _{g_{b_0}}\cdot X_{b_0}, \end{aligned}$$

be symbols corresponding to the choice of vectorfield multiplier and Lagrangian corrector in Lemma 8.8^19^. Then for sufficiently small *a*, there exist smooth homogeneous symbols $\tilde{\varkappa }\in S_{{\operatorname {tan}}}^1(\Sigma ) + \sigma S_{{\operatorname {tan}}}^0(\Sigma )$, $\tilde{\mathfrak {q}}\in S^0+\sigma S^{-1}$ that depend smoothly on *a* such that defining,

$$\begin{aligned} \varkappa _b = \varkappa _{b_0} + a\tilde{\varkappa },\qquad \mathfrak {q}_b = \mathfrak {q}_{b_0}+ a\tilde{\mathfrak {q}}, \end{aligned}$$

for $\left\lvert r-3M\right\rvert < \delta _r$, we have the following sum of squares representation

190 $$\begin{aligned} \rho ^2\left( \frac{1}{2\mathbbm {i}}H_{p_b} \varkappa _{b} + p_b \mathfrak {q}_{b} \right) = \sum _{j=1}^7\mathfrak {a}_j^2 \end{aligned}$$

where $$\mathfrak {a}_j\in S_{{\operatorname {tan}}}^1(\Sigma ) + \sigma S_{{\operatorname {tan}}}^0(\Sigma )$$. Moreover,

1. The decomposition (190) extends the decomposition in (184) in the sense that
  $$\begin{aligned} \begin{aligned}&(\mathfrak {a}_1, \mathfrak {a}_2, \mathfrak {a}_3, \mathfrak {a}_4, \mathfrak {a}_5) \mod a(S^1_{hom} + \sigma S^0_{hom}) \\ ={}&\left( \sqrt{\delta _1}\alpha _{b_0}\sigma , \beta _{b_0}\xi , \sqrt{\frac{\mu _{b_0}(1-\delta _1)}{r^2}}\alpha _{b_0}\eta _{\theta }, \sqrt{\frac{\mu _{b_0}(1-\delta _1)}{r^2}}\alpha _{b_0}\eta _{\varphi }, \sqrt{\frac{\mu _{b_0}(1-\delta _1)}{r^2}}\alpha _{b_0}\xi \right) , \end{aligned} \end{aligned}$$
  and
  $$\begin{aligned} (\mathfrak {a}_6,\mathfrak {a}_7) \in \sqrt{a}(S^1_{hom}+\sigma S^0_{hom}). \end{aligned}$$
2. $\{\mathfrak {a}_j\}_{1\le j\le 7}$ is elliptically equivalent to the family of symbols $(\ell _2(\sigma -\sigma _1),\ell _1(\sigma -\sigma _2),\xi )$ in the sense that there exists a symbol valued matrix $\mathbb {M}\in M^{7\times 3}(S^0)$ with maximum rank 3 everywhere such that
  $$\begin{aligned} \mathfrak {a}= \mathbb {M} \mathfrak {b},\quad \mathfrak {b}= \begin{pmatrix} \ell _2(\sigma -\sigma _1)\\ \ell _1(\sigma -\sigma _2)\\ \xi \end{pmatrix}. \end{aligned}$$

##### Proof

As discussed above, the main idea is to find appropriate pseudo-differential modifications of the vectorfield $X_{b_0}$ and the Lagrangian correction $q_{b_0}$ that are adapted to the perturbed trapping dynamics of Kerr-de Sitter.

For the sake of simplifying some of our ensuing calculations, define

$$\begin{aligned} \mathfrak {q}'_{b_0} = \mathfrak {q}_{b_0} - 2\left\{ \ln \rho _b, \varkappa _{b_0}\right\} , \qquad \tilde{\mathfrak {q}}'_{b_0} = \tilde{\mathfrak {q}}_{b_0} -2\left\{ \ln \rho _b, \tilde{\varkappa }\right\} , \end{aligned}$$

so that

$$\begin{aligned} \rho ^2_b\left( \frac{1}{2\mathbbm {i}}H_p(\varkappa _{b_0}+a\tilde{\varkappa }) +(\mathfrak {q}_{b_0}+a\tilde{\mathfrak {q}})p_b \right) = \frac{1}{2\mathbbm {i}}H_{{\rho _b^2 p}} (\varkappa _{b_0}+a\tilde{\varkappa }) + \left( \tilde{\mathfrak {q}}' +a\tilde{\mathfrak {q}}'\right) ({\rho _b^2 p}_b). \end{aligned}$$

We first choose $\tilde{\varkappa }$ so that $H_{{\rho _b^2 p}_b} (\varkappa _{b_0}+a\tilde{\varkappa })$ vanishes at the trapped set ${\Gamma }_b$. The most immediate Kerr-de Sitter extension of the choice of $\varkappa _{b_0}$ is the symbol

$$\begin{aligned} \varkappa _{b}'{:=}\mathbbm {i}\Delta _{b}\rho _b^{-4}\left( r-\mathring{r}_b(\sigma ,\eta _{\varphi })\right) \xi = \mathbbm {i}\frac{r-\mathring{r}_b(\sigma ,\eta _{\varphi })}{2\rho _b^4}H_{{\rho _b^2 p}_{b}}r. \end{aligned}$$

This symbol would clearly extend our choice in Schwarzschild-de Sitter in the sense that

$$\begin{aligned} \varkappa _{b_0}' = \varkappa _{b_0}, \end{aligned}$$

and moreover, $\varkappa _{b}'$ is well defined and smooth in a neighborhood of the trapped set. We can calculate that on the characteristic set $p_b=0$, we have

$$\begin{aligned} 2 H_{{\rho _b^2 p}}\varkappa _b' = \left( \frac{1}{\rho _b^4} - \frac{4(r-\mathring{r}_b)\partial _r\rho _b}{\rho _b^5}\right) (H_{{\rho _b^2 p}_b} r)^2 + \frac{r-\mathring{r}_b(\sigma ,\eta _{\varphi })}{\rho _b^4}H_{{\rho _b^2 p}_b}^2r. \end{aligned}$$

Recall from (E15) that for $p=0$, near $r=3M$, we have that

$$\begin{aligned} H_{{\rho _b^2 p}_b}^2r = 2\Delta _b\partial _r\left( \frac{(1+\lambda _b)^2}{\Delta _b}\left( (r^2+a^2)\sigma +a\eta _{\varphi }\right) \right) . \end{aligned}$$

Since $\mathring{r}_b$ is the unique minimum of $\frac{(1+\lambda _b)^2}{\Delta _b}\left( (r^2+a^2)\sigma +a\eta _{\varphi }\right)$, and we are in a $\delta _r$ neighborhood of $r=3M$, there exist positive symbols $\alpha ,\beta \in S^0_{\hom }$ such that on $p_b=0$, near $r=3M$,

191 $$\begin{aligned} H_{{\rho _b^2 p}}\varkappa _b' = \alpha ^2(r,\sigma , \eta _{\varphi }) \sigma ^2(r-\mathring{r}_b)^2 + \beta ^2(r,\sigma , \eta _{\varphi })\xi ^2. \end{aligned}$$

Unfortunately, the problem with $\varkappa _b'$ is that it is not a polynomial in $\sigma$, and thus cannot be directly used in conjunction with our integration-by-parts or divergence theorem method to produce a Morawetz estimate. To overcome this difficulty, recall that we defined $\varkappa _b'$ so that it is smooth in *a*, and so that

$$\begin{aligned} \varkappa _b'-\varkappa _{b_0}\in aS^1_{hom}. \end{aligned}$$

Thus using Mather division theorem (Theorem A.3) to divide $\frac{1}{\mathbbm {i}}\left( \varkappa _b' - \varkappa _{b_0}\right)$ by the principal symbol of the wave operator, $p_b$, gives us

192 $$\begin{aligned} \frac{1}{\mathbbm {i}}\left( \varkappa _b'\!-\!\varkappa _{b_0}\right) \!=\! a\left( \tilde{\varkappa }_1(r,\theta ;\xi ,\eta _{\theta }, \eta _{\varphi }) \!+\! \tilde{\varkappa }_0(r,\theta ;\xi , \eta _{\theta }, \eta _{\varphi })\sigma \right) \!+\! a \mathfrak {r}(r,\theta ;\xi ,\eta _{\theta }, \eta _{\varphi })p_b, \end{aligned}$$

where $\tilde{\varkappa }_i\in S^i_{hom}$ and $\mathfrak {r}\in S^{-1}_{hom}$. Now, we define $\tilde{\varkappa }$ so that

193 $$\begin{aligned} \frac{1}{\mathbbm {i}}\tilde{\varkappa } = \tilde{\varkappa }_1+\tilde{\varkappa }_0\sigma , \end{aligned}$$

so that on $p_b=0$,

$$\begin{aligned} \varkappa _b = \varkappa _{b_0} + a\tilde{\varkappa } = \varkappa _b'. \end{aligned}$$

Thus $\varkappa _b$ is a symbol which is a polynomial in $\sigma$ and moreover vanishes at the trapped set ${\Gamma }_{b}$.

*A priori*, $H_{\rho _b^2 p}\varkappa _b$ is a third degree polynomial in $\sigma$. Applying the Mather division theorem (Theorem A.3) again yields that there exist some $\gamma _1\in S_{{\operatorname {tan}}}^1, \gamma _2 \in S_{{\operatorname {tan}}}^2$, $f_0\in S_{{\operatorname {tan}}}^0, f_{-1}\in \sigma S_{{\operatorname {tan}}}^{-1}$ such that

$$\begin{aligned} \frac{1}{2\mathbbm {i}\rho _b^2} H_{{\rho _b^2 p}_b}(\varkappa _{b_0}\!+\!a\tilde{\varkappa }) \!+\! \mathfrak {q}_{b_0}' ({\rho _b^2 p}_b) \!= \!\gamma _2\!+\!\gamma _1\sigma \!+\! \left( e_{b_0} \!+\! a(f_0+f_{-1}\sigma )\right) (\sigma \!-\!\sigma _1)(\sigma \!-\!\sigma _2), \end{aligned}$$

observing that

$$\begin{aligned} e_{b_0} {:=} \delta _1\alpha _{b_0}^2. \end{aligned}$$

is the coefficient for $\sigma ^2$ in the expression for $\frac{1}{2\mathbbm {i}}H_{p_{b_0}}\varkappa _{b_0} + \mathfrak {q}_{b_0}p_{b_0}$ (see (184)). It now remains to demonstrate that $\gamma _2+\gamma _1\sigma +e_{b_0}(\sigma -\sigma _1)(\sigma -\sigma _2)$ can be expressed as a sum of squares up to some error in $a(S_{{\operatorname {tan}}}^0+ S_{{\operatorname {tan}}}^{-1}\sigma )p_b$. If this were true, we could write

194 $$\begin{aligned} \gamma _2+\gamma _1\sigma + e_{b_0}(\sigma -\sigma _1)(\sigma -\sigma _2) = \sum \mathfrak {a}_j^2 + a(g_0+g_{-1}\sigma )(\sigma -\sigma _1)(\sigma -\sigma _2). \end{aligned}$$

We could then define $\tilde{\mathfrak {q}}$ such that

$$\begin{aligned} \tilde{\mathfrak {q}}' = -2\left( f_0+g_0+(f_{-1}+g_{-1})\sigma \right) , \end{aligned}$$

so that the $a(S_{{\operatorname {tan}}}^0+\sigma S_{{\operatorname {tan}}}^{-1})p_b$ terms are all canceled.

We now return to showing (194). Recall that on $p_b=0$,

$$\begin{aligned} H_{{\rho _b^2 p}_b}(\varkappa _{b_0} + a\tilde{\varkappa }) = H_{{\rho _b^2 p}_b}\varkappa _b'. \end{aligned}$$

As a result of (191), we now have that if $\sigma =\sigma _i$, which in particular implies that $p_b=0$,

$$\begin{aligned} \gamma _2+\gamma _1\sigma =\alpha ^2(r, \sigma , \eta _{\varphi })\sigma ^2(r-\mathring{r}_b)^2 + \beta ^2(r,\sigma ,\eta _{\varphi })\xi ^2. \end{aligned}$$

We can solve for $\gamma _2, \gamma _1$ explicitly now by considering the two-dimensional system of equations

$$\begin{aligned} \begin{aligned} \gamma _2+\gamma _1\sigma _i&= \frac{1}{4}\alpha _i^2(\sigma _1-\sigma _2)^2 + \beta _i^2\xi ^2,\\ \alpha _i&= \frac{2\left\lvert \sigma _i\right\rvert }{\sigma _1-\sigma _2}\alpha (r, \sigma _i, \eta _{\varphi })(r-\mathring{r}_b(\sigma _i, \eta _{\varphi }))\in S_{{\operatorname {tan}}}^0,\\ \beta _i&= \beta (r, \sigma _i, \eta _{\varphi }) \in S_{{\operatorname {tan}}}^0. \end{aligned} \end{aligned}$$

Solving the system yields

195 $$\begin{aligned} \begin{aligned} \gamma _2&= \frac{1}{4}(\sigma _1-\sigma _2)(\alpha _2^2\sigma _1 - \alpha _1^2\sigma _2) + \frac{\sigma _1\beta _2^2 - \sigma _2\beta _1^2}{\sigma _1-\sigma _2}\xi ^2, \\ \gamma _1&= \frac{1}{4}(\sigma _1-\sigma _2)(\alpha _1^2-\alpha _2^2)+\frac{\beta _1^2 - \beta _2^2}{\sigma _1-\sigma _2}\xi ^2. \end{aligned} \end{aligned}$$

We first add together the first two terms in $\gamma _i$ to see that

196 $$\begin{aligned} \begin{aligned}&(\sigma _1-\sigma _2)\left( \alpha _2^2\sigma _1 -\alpha _1^2\sigma _2 + \sigma (\alpha _1^2-\alpha _2^2)\right) \\ ={}&(1-{\delta _1})(\alpha _1(\sigma -\sigma _2)-\alpha _2(\sigma -\sigma _1))^2 + {\delta _1}\left( \alpha _1(\sigma -\sigma _2)+\alpha _2(\sigma -\sigma _1)\right) ^2\\&- 4e_b(\sigma -\sigma _1)(\sigma -\sigma _2), \end{aligned} \end{aligned}$$

where

$$\begin{aligned} e_b = \frac{(\alpha _1-\alpha _2)^2}{4}+ {\delta _1}\alpha _1\alpha _2. \end{aligned}$$

Recall that in $g_{b_0}$, $\alpha _1=\alpha _2=\alpha _{b_0}$, $\sigma _2=-\sigma _1$, and that $\mathfrak {q}_{b_0} = \delta _1\alpha _{b_0}^2$. This implies that

197 $$\begin{aligned} e_b-e_{b_0}\in a(S_{{\operatorname {tan}}}^0 + \sigma S_{{\operatorname {tan}}}^{-1}) \end{aligned}$$

as desired. We now add together the second terms in the $\gamma _i$ given in (195)

198 $$\begin{aligned} \begin{aligned} \frac{\sigma _1\beta _2^2-\sigma _2\beta _1^2}{\sigma _1-\sigma _2} + \sigma \frac{\beta _1^2-\beta _2^2}{\sigma _1-\sigma _2} ={}&\frac{1}{2}\left( \beta _1^2+\beta _2^2 -Ca\right) + \frac{(Ca-\beta _2^2+\beta _1^2)(\sigma -\sigma _2)^2}{2(\sigma _1-\sigma _2)^2}\\&+ \frac{(Ca-\beta _1^2+\beta _2^2)(\sigma -\sigma _1)^2}{2(\sigma _1-\sigma _2)^2} + O(a)p_b. \end{aligned} \end{aligned}$$

Summing (196) and (198) together, we have that

$$\begin{aligned} \begin{aligned}&\frac{1}{2\mathbbm {i}}H_{{\rho _b^2 p}_b}( \varkappa _{b_0}+a\tilde{\varkappa }) + ({\rho _b^2 p}_b)\mathfrak {q}'_b\\ ={}&\frac{1-{\delta _1}}{4}\left( \alpha _1(\sigma -\sigma _2)-\alpha _2(\sigma -\sigma _1) \right) ^2 + \frac{{\delta _1}}{4}\left( \alpha _1(\sigma -\sigma _2) + \alpha _2(\sigma -\sigma _1)\right) ^2\\&+ \frac{1}{2}\left( \beta _1^2+\beta _2^2 - Ca\right) \xi ^2 + \frac{(Ca-\beta _2^2+\beta _1^2)(\sigma -\sigma _2)^2}{2(\sigma _1-\sigma _2)^2}\xi ^2\\&+ \frac{(Ca-\beta _1^2+\beta _2^2)(\sigma -\sigma _1)^2}{2(\sigma _1-\sigma _2)^2}\xi ^2 +a(S_{{\operatorname {tan}}}^0+S_{{\operatorname {tan}}}^{-1}\sigma )(\sigma -\sigma _1)(\sigma -\sigma _2). \end{aligned} \end{aligned}$$

We then pick

$$\begin{aligned} \mathfrak {a}_1^2&= \frac{\delta _1}{4}\left( \alpha _1(\sigma -\sigma _2)+\alpha _2(\sigma -\sigma _1)\right) ^2,\\ \mathfrak {a}_2^2&= \frac{1}{2}\left( \beta _1^2 + \beta _2^2 - Ca\right) \xi ^2,\\ \mathfrak {a}_{3}^2&= \frac{\eta _{\theta }^2}{\left\lvert \eta \right\rvert ^2 + \Delta _{b_0}\xi ^2}\frac{(1-\delta _1)}{4} \left( \alpha _1(\sigma -\sigma _2)-\alpha _2(\sigma -\sigma _1)\right) ^2, \\ \mathfrak {a}_{4}^2&= \frac{\eta _{\varphi }^2}{\left\lvert \eta \right\rvert ^2 + \Delta _{b_0}\xi ^2}\frac{(1-\delta _1)}{4} \left( \alpha _1(\sigma -\sigma _2)-\alpha _2(\sigma -\sigma _1)\right) ^2,\\ \mathfrak {a}_{5}^2&= \frac{\Delta _{b_0}\xi ^2}{\left\lvert \eta \right\rvert ^2 + \Delta _{b_0}\xi ^2}\frac{(1-\delta _1)}{4}\left( \alpha _1(\sigma -\sigma _2)-\alpha _2(\sigma -\sigma _1)\right) ^2,\\ \mathfrak {a}_6^2&= \frac{\left( Ca-\beta _2^2 + \beta _1^2\right) \left( \sigma -\sigma _2\right) ^2}{2\left( \sigma _1-\sigma _2\right) ^2}\xi ^2,\\ \mathfrak {a}_7^2&= \frac{\left( Ca-\beta _1^2 + \beta _2^2\right) \left( \sigma -\sigma _1\right) ^2}{2\left( \sigma _1-\sigma _2\right) ^2}\xi ^2, \end{aligned}$$

concluding the proof of Lemma 8.11. $\square$

Before we proceed, we note the following useful rewriting of $\varkappa _b$.

##### Corollary 8.12

Let $\varkappa _b$ be as constructed in the proof of Lemma 8.11. Then we can write that

199 $$\begin{aligned} \varkappa _b = \frac{\Delta _{b}}{\rho _b^4}(r-\mathring{r}_b(\sigma _1, \eta _{\varphi }))\frac{\xi (\sigma -\sigma _2)}{\sigma _1-\sigma _2} + \frac{\Delta _{b}}{\rho _b^4}(r-\mathring{r}_b(\sigma _2, \eta _{\varphi }))\frac{\xi (\sigma -\sigma _1)}{\sigma _2-\sigma _1}. \end{aligned}$$

As a result, if *u* is compactly supported on $\mathring{\Sigma }$, then

200 $$\begin{aligned} \left\Vert X_bu\right\Vert _{L^2(\mathring{\Sigma })} \lesssim \left\Vert u\right\Vert _{Mor_b(\mathring{\Sigma })}. \end{aligned}$$

##### Proof

Using the observation that $\varkappa _b\in \sigma S^0 + S^1$, we can apply the Mather division theorem (Theorem A.3) to write that there exist $\mathfrak {e}_{(i)}$, $\mathfrak {r}_{(i)}$, for $i=1,2$ such that

201 $$\begin{aligned} \begin{aligned} \varkappa _b&= (\sigma -\sigma _1)\mathfrak {e}_{(1)} + \mathfrak {r}_{(1)},\\ \varkappa _b&= (\sigma -\sigma _2)\mathfrak {e}_{(2)} + \mathfrak {r}_{(2)}. \end{aligned} \end{aligned}$$

Moreover, using (192), we have that

$$\begin{aligned} \begin{aligned} (r-\mathring{r}_b(\sigma _1, \eta _{\varphi }))\frac{H_{{\rho _b^2 p}_b}r}{2\rho _b^4} = \mathfrak {r}_1,\\ (r-\mathring{r}_b(\sigma _2, \eta _{\varphi }))\frac{H_{{\rho _b^2 p}_b}r}{2\rho _b^4} = \mathfrak {r}_2. \end{aligned} \end{aligned}$$

Solving the combined system of equations given by (201) and (8.4.2) for $\mathfrak {e}_{(i)}$ and $\mathfrak {r}_{(i)}$, $i=1,2$ then yields (199) using the fact that

$$\begin{aligned} H_{{\rho _b^2 p}_b}r = 2\Delta _{b}\xi . \end{aligned}$$

The bound in (200) is an immediate corollary of Lemma 8.11. $\square$

We now illustrate how to account for the contribution of the subprincipal operator in the principal bulk term.

##### Corollary 8.13

Let

202 $$\begin{aligned} \overline{\textbf{s}}_{b,a} = \sigma _1(\overline{\textbf{S}}_{b,a}) \end{aligned}$$

denote the principal symbol of the skew-adjoint component of the subprincipal operator of $\overline{\mathbb {L}}_{g_b}$. For $a<a_0$ of Lemma 8.11, and the symbols $\tilde{\varkappa }, \tilde{\mathfrak {q}}$ of Lemma 8.11, there exists a choice of $Q$, such that in a small neighborhood of ${\Gamma }_b$, (in both frequency and physical space),

203 $$\begin{aligned} \rho _b^2\mathfrak {k}^{\varkappa _b,\mathfrak {q}_b} {:=} \rho _b^2 \left( \frac{1}{2\mathbbm {i}}H_{p_b}\varkappa _{b} + p_b\mathfrak {q}_b - \overline{\textbf{s}}_{b,a}\varkappa _b \right) \ge \frac{1}{2}\sum _{j=1}^7\mathfrak {a}_{j}^2. \end{aligned}$$

##### Proof

We use the characterization of $\varkappa _b$ in Corollary 8.12 to write

$$\begin{aligned} \overline{\textbf{s}}_b\varkappa _b = \frac{\Delta _b}{\rho _b^4}(r-\mathring{r}_b(\sigma _1, \eta _{\varphi }))\frac{\xi (\sigma -\sigma _2) \overline{\textbf{s}}_b}{\sigma _1-\sigma _2} + \frac{\Delta _b}{\rho _b^4}(r-\mathring{r}_b(\sigma _2, \eta _{\varphi }))\frac{\xi (\sigma -\sigma _1) \overline{\textbf{s}}_b}{\sigma _2-\sigma _1}. \end{aligned}$$

Recall from Lemma 7.18 that for any fixed $\delta _0>0$, there exists a choice of *a*, $\varepsilon _{{\Gamma }_{b_0}}$, $\delta _r$, and $\delta _\zeta$ such that

$$\begin{aligned} \left\lvert \overline{\textbf{s}}_b\right\rvert \lesssim \delta _0\left\lvert \zeta \right\rvert . \end{aligned}$$

On $\mathring{\Sigma }$, $\left\lvert \xi \right\rvert ^2 + \left\lvert \eta \right\rvert ^2 \lesssim \sigma _1^2 + \sigma _2^2$, so it will be sufficient to bound

$$\begin{aligned} \begin{aligned} \left\lvert \frac{\Delta _b}{\rho _b^4}(r-\mathring{r}_b(\sigma _1,\eta _{\varphi }))\frac{\xi (\sigma -\sigma _2)\sigma }{\sigma _1-\sigma _2}\right\rvert ,&\quad \left\lvert \frac{\Delta _b}{\rho _b^4}(r-\mathring{r}_b(\sigma _2,\eta _{\varphi }))\frac{\xi (\sigma -\sigma _1)\sigma }{\sigma _2-\sigma _1}\right\rvert ,\\ \left\lvert \frac{\Delta _b}{\rho _b^4}(r-\mathring{r}_b(\sigma _1,\eta _{\varphi }))\frac{\xi (\sigma -\sigma _2)\sigma _1}{\sigma _1-\sigma _2}\right\rvert ,&\quad \left\lvert \frac{\Delta _b}{\rho _b^4}(r-\mathring{r}_b(\sigma _2,\eta _{\varphi }))\frac{\xi (\sigma -\sigma _1)\sigma _1}{\sigma _2-\sigma _1}\right\rvert ,\\ \left\lvert \frac{\Delta _b}{\rho _b^4}(r-\mathring{r}_b(\sigma _1,\eta _{\varphi }))\frac{\xi (\sigma -\sigma _2)\sigma _2}{\sigma _1-\sigma _2}\right\rvert ,&\quad \left\lvert \frac{\Delta _b}{\rho _b^4}(r-\mathring{r}_b(\sigma _2,\eta _{\varphi }))\frac{\xi (\sigma -\sigma _1)\sigma _2}{\sigma _2-\sigma _1}\right\rvert , \end{aligned} \end{aligned}$$

by $\sum _{j=1}^7\mathfrak {a}_j^2$ to conclude.

The two terms in each line are handled in an identical manner (simply switching $\sigma _1$, $\sigma _2$), so without loss of generality, we will only handle one term from each line. Let us first consider the symbol given by

$$\begin{aligned} \frac{\Delta _b}{\rho _b^4}(r-\mathring{r}_b(\sigma _1, \eta _{\varphi }))\frac{\xi (\sigma -\sigma _2) \sigma }{\sigma _1-\sigma _2}. \end{aligned}$$

We can immediately write

204 $$\begin{aligned} \begin{aligned} \frac{\Delta _b}{\rho _b^4}(r-\mathring{r}_b(\sigma _1, \eta _{\varphi }))\frac{\xi (\sigma -\sigma _2) \sigma }{\sigma _1-\sigma _2} ={}&\frac{\Delta _b}{\rho _b^4}(r-\mathring{r}_b(\sigma _1, \eta _{\varphi }))\frac{\xi (\sigma -\sigma _1)(\sigma -\sigma _2)}{\sigma _1-\sigma _2}\\&+ \frac{\Delta _b}{\rho _b^4}(r-\mathring{r}_b(\sigma _1, \eta _{\varphi }))\frac{\xi (\sigma -\sigma _2)\sigma _1}{\sigma _1-\sigma _2}. \end{aligned} \end{aligned}$$

Recalling the explicit forms of $\left\{ \mathfrak {a}_j\right\} _{j=1}^{7}$, we see that applying Cauchy-Schwarz yields that

$$\begin{aligned} \frac{\Delta _b}{\rho _b^4}(r-\mathring{r}_b(\sigma _1, \eta _{\varphi }))\frac{\xi (\sigma -\sigma _2)\sigma _1}{\sigma _1-\sigma _2} \lesssim \mathfrak {a}_1^2 + \mathfrak {a}_2^2. \end{aligned}$$

To deal with the first term on the right-hand side of (204), we again apply Cauchy Schwarz,

$$\begin{aligned} \frac{\Delta _b}{\rho _b^4}(r\!-\!\mathring{r}_b(\sigma _1, \eta _{\varphi }))\frac{\xi (\sigma \!-\!\sigma _1)(\sigma \!-\!\sigma _2)}{\sigma _1\!-\!\sigma _2} \le \frac{\Delta _b^2}{2\rho _b^8}\frac{\xi ^2(\sigma \!-\!\sigma _1)^2}{(\sigma _1\!-\!\sigma _2)^2} \!+\! \frac{1}{2}(r\!-\!\mathring{r}_b(\sigma _1,\eta _{\varphi }))^2(\sigma \!-\!\sigma _2)^2. \end{aligned}$$

The first term on the right-hand side is controlled by $\mathfrak {a}_7^2$, while the second term is controlled by $\mathfrak {b}$. Now let us consider the symbol

$$\begin{aligned} \frac{\Delta _b}{\rho _b^4}(r-\mathring{r}_b(\sigma _1,\eta _{\varphi }))\frac{\xi (\sigma -\sigma _2)\sigma _1}{\sigma _1-\sigma _2}. \end{aligned}$$

As previously mentioned, this term can be controlled directly by applying Cauchy-Schwarz, using the explicit forms of $\left\{ \mathfrak {a}_j\right\} _{j=1}^7$,

$$\begin{aligned} \frac{\Delta _b}{\rho _b^4}(r-\mathring{r}_b(\sigma _1,\eta _{\varphi }))\frac{\xi (\sigma -\sigma _2)\sigma _1}{\sigma _1-\sigma _2} \lesssim \mathfrak {a}_1^2 + \mathfrak {a}_2^2. \end{aligned}$$

We now handle the final term,

$$\begin{aligned} \frac{\Delta _b}{\rho _b^4}(r-\mathring{r}_b(\sigma _1,\eta _{\varphi }))\frac{\xi (\sigma -\sigma _2)\sigma _2}{\sigma _1-\sigma _2}. \end{aligned}$$

To control this term, we use that

$$\begin{aligned} \begin{aligned} \left\lvert \frac{\Delta _b}{\rho _b^4}(r-\mathring{r}_b(\sigma _1,\eta _{\varphi }))\frac{\xi (\sigma -\sigma _2)\sigma _2}{\sigma _1-\sigma _2}\right\rvert \le {}&\left\lvert \frac{\Delta _b}{\rho _b^4}(r-\mathring{r}_b(\sigma _1,\eta _{\varphi }))\frac{\xi (\sigma -\sigma _2)\sigma _1}{\sigma _1-\sigma _2}\right\rvert \\&+ \left\lvert \frac{\Delta _b}{\rho _b^4}(r-\mathring{r}_b(\sigma _1,\eta _{\varphi }))\xi (\sigma -\sigma _2)\right\rvert . \end{aligned} \end{aligned}$$

At this point we can again use Cauchy-Schwarz to bound

$$\begin{aligned} \left\lvert \frac{\Delta _b}{\rho _b^4}(r-\mathring{r}_b(\sigma _1,\eta _{\varphi }))\frac{\xi (\sigma -\sigma _2)\sigma _1}{\sigma _1-\sigma _2}\right\rvert + \left\lvert \frac{\Delta _b}{\rho _b^4}(r-\mathring{r}_b(\sigma _1,\eta _{\varphi }))\xi (\sigma -\sigma _2)\right\rvert \lesssim \mathfrak {a}_{1}^2 + \mathfrak {a}_2^2 + \mathfrak {b}^2. \end{aligned}$$

Combining the above estimates, we see that if $\delta _0$ is chosen sufficiently small,

$$\begin{aligned} \left\lvert \overline{\textbf{s}}_b\varkappa _b\right\rvert \le \frac{1}{2}\sum _{j=1}^7 \mathfrak {a}_j^2, \end{aligned}$$

which concludes the proof of Corollary 8.13. $\square$

We are now ready to prove Lemma 8.10.

##### Proof of Lemma 8.10

We pick

$$\begin{aligned} \widetilde{X}_i = \frac{1}{2}\left( {\mathring{\chi }}\operatorname {Op}(\widetilde{\varkappa }_i){\mathring{\chi }} - ({\mathring{\chi }}\operatorname {Op}(\widetilde{\varkappa }_i){\mathring{\chi }})^*\right) , \qquad \tilde{q}_i = \frac{1}{2}\left( {\mathring{\chi }}\operatorname {Op}(\tilde{\mathfrak {q}}_i){\mathring{\chi }} + ({\mathring{\chi }}\operatorname {Op}(\tilde{\mathfrak {q}}_i){\mathring{\chi }})^{*} \right) , \end{aligned}$$

where the adjoint is taken with respect to the $L^2(\mathring{\Sigma })$ inner product.

Let us rewrite

205 $$\begin{aligned} \mathfrak {X}_{b_0} {:=} X_{b_0} + \frac{1}{2}\nabla _{g_{b}} \cdot X_{b_0},\qquad \tilde{\mathfrak {q}}_{b_0} {:=} q_{b_0} - \frac{1}{2}\nabla _{g_{b}} \cdot X_{b_0}, \end{aligned}$$

so that $\mathfrak {X}_{b_0}$ is anti-Hermitian and $\tilde{\mathfrak {q}}_{b_0}$ is Hermitian with respect to the $L^2(\mathcal {D})$ inner product. Observe that by construction,

$$\begin{aligned} \tilde{\mathfrak {q}}_{b_0} = \frac{1}{2}\left( \nabla _{g_b}-\nabla _{g_{b_0}} \right) \cdot X_{b_0} + q_1, \end{aligned}$$

where $q_1$ is as defined in (182). This directly implies that

$$\begin{aligned} \tilde{\mathfrak {q}}_{b_0} \lesssim a + \delta _r. \end{aligned}$$

Then directly by integrating by parts, we have that

206 $$\begin{aligned} 2\Re \left\langle \overline{\textbf{S}}_b h , \left( X_{b_0} \!+\! q_{b_0}\right) h\right\rangle _{L^2(\mathcal {D})} \!=\!{}&- \left\langle \left( \mathfrak {X}_{b_0}\overline{\textbf{S}}_b \!+\! \overline{\textbf{S}}_b\mathfrak {X}_{b_0}\right) h , h\right\rangle _{L^2(\mathcal {D})} \!+\! 2\Re \left\langle \overline{\textbf{S}}_bh, \tilde{\mathfrak {q}}_{b_0}h\right\rangle _{L^2(\mathcal {D})}\nonumber \\ \Re \left\langle \overline{\textbf{S}}_b h , \widetilde{X} h\right\rangle _{L^2(\mathcal {D})} \!=\!{}&\!-\! \left\langle \left( \widetilde{X}\overline{\textbf{S}}_b \!+\! \overline{\textbf{S}}_b\widetilde{X}\right) h , h\right\rangle _{L^2(\mathcal {D})} \!-\! {\Re \left\langle \overline{\textbf{S}}_0h, \widetilde{X} h\right\rangle _{L^2(\mathring{\Sigma })}}\Bigg \vert ^{t_*={T_*}}_{t_*=0}\nonumber \\&\!- \!{\Re \left\langle \overline{\textbf{S}}_bh, \widetilde{X}_0h\right\rangle _{L^2(\mathring{\Sigma })}}\Bigg \vert ^{t_*={T_*}}_{t_*=0}, \end{aligned}$$

where

$$\begin{aligned} \overline{\textbf{S}}_{b} = \overline{\textbf{S}}_0\partial _{t_*} + \overline{\textbf{S}}_1,\qquad \widetilde{X} = \widetilde{X}_0\partial _{t_*} + \widetilde{X}_1, \end{aligned}$$

and $\widetilde{X}_i, \overline{\textbf{S}}_i\in \operatorname {Op}S^i$. Then Corollary 8.13 and Theorem 7.6 show that

$$\begin{aligned} \int _{\mathcal {D}}K^{X_{b_0}, q_{b_0}, 0}[h] + a\mathbb {K}^{\widetilde{X}, \tilde{q}}[h] + \Re \left\langle \left( \mathfrak {X}_{b_0}\overline{\textbf{S}}_b + \overline{\textbf{S}}_b\mathfrak {X}_{b_0}\right) h , h\right\rangle _{L^2(\mathcal {D})} \gtrsim \left\Vert h\right\Vert _{Mor_b(\mathcal {D})}^2. \end{aligned}$$

The conclusion of the lemma then follows from the definition of $\tilde{\mathfrak {q}}_{b_0}$ and (206). $\square$

##### Remark 36

At first glance, the pseudo-differential operators $\widetilde{X}$ and $\tilde{q}$ are only well-defined away from the singularities of the $(\theta ,\phi )$ coordinates on the sphere, namely, the poles. However, we can smoothly extend both $\widetilde{X}$ and $\tilde{q}$ to the poles by repeating their constructions in charts covering the poles and gluing the resulting operators together since their construction only relies on $\sigma _1$, $\sigma _2$, which are smooth at the poles.

**Lower-order bulk terms**

Next, we show that the lower-order bulk terms can be appropriately controlled by the (degenerate) ellipticity of the principal symbol. We start with some auxiliary lemmas that will be useful in controlling the lower-order terms that appear in the divergence theorem argument.

The easiest terms to control will be the lowest-order bulk terms that contain no $D_{t_*}$ derivatives.

##### Lemma 8.14

Let $\mathcal {D}$ be as defined in (176). Fix $\delta >0$ and $\textbf{V}\in \Psi _{{\operatorname {tan}}}^0(\Sigma )$ such that $\textbf{V}$ has Schwartz kernel compactly supported on $\mathring{\Sigma }$. Then, for $\delta _r$ sufficiently small and *h* compactly supported on $\mathcal {D}$,

$$\begin{aligned} \left\lvert \Re \left\langle \textbf{V}h, h\right\rangle _{L^2(\mathcal {D})}\right\rvert \lesssim \delta \left( \left\Vert h\right\Vert _{L^2(\mathcal {D})}^2 + \left\Vert \partial _rh\right\Vert _{L^2(\mathcal {D})}^2\right) , \end{aligned}$$

and in particular,

$$\begin{aligned} \left\Vert h\right\Vert _{L^2(\mathcal {D})}^2 \lesssim \delta \left( \left\Vert h\right\Vert _{L^2(\mathcal {D})}^2 + \left\Vert \partial _rh\right\Vert _{L^2(\mathcal {D})}^2\right) . \end{aligned}$$

Similarly, if $h(t_*, \cdot )$ is compactly supported on $\mathring{\Sigma }$ for all $t_*\ge 0$, then for $\delta _r$ sufficiently small,

$$\begin{aligned} \left\lvert \Re \left\langle \textbf{V}h, h\right\rangle _{L^2(\mathring{\Sigma })}\right\rvert \lesssim \delta \left( \left\Vert h\right\Vert _{L^2(\mathring{\Sigma })}^2 + \left\Vert \partial _rh\right\Vert _{L^2(\mathring{\Sigma })}^2\right) . \end{aligned}$$

In particular,

$$\begin{aligned} \left\Vert h\right\Vert _{L^2(\mathring{\Sigma })}^2 \lesssim \delta \left( \left\Vert h\right\Vert _{L^2(\mathring{\Sigma })}^2 + \left\Vert \partial _rh\right\Vert _{L^2(\mathring{\Sigma })}^2\right) . \end{aligned}$$

##### Proof

Using the fact that $\partial _r(r-3M)=1$, we use integration by parts to see that

$$\begin{aligned} -\Re \left\langle \textbf{V}h, h\right\rangle _{L^2(\mathring{\Sigma })} = \left\langle (r-3M)\textbf{V}\partial _rh,h\right\rangle _{L^2(\mathcal {D})} + \left\langle (r-3M)\textbf{V}h,\partial _rh\right\rangle _{L^2(\mathcal {D})} + Err[h], \end{aligned}$$

where $\left\lvert Err[h]\right\rvert \lesssim \delta _r\left\Vert h\right\Vert _{L^2(\mathcal {D})}^2$. The first then follows by Cauchy-Schwarz and taking $\delta _r$ sufficiently small. Observe that since the argument only involved integration by parts in $\partial _r$, we can repeat the argument over $\mathring{\Sigma }$ instead of over $\mathcal {D}$ to achieve the second conclusion. $\square$

Throughout the proof, we will also accumulate lower-order terms of the form $\left\langle D_x\right\rangle ^{-1}D_{t_*}$ which need to be dealt with. Fortunately, this can be done with a simple symbol decomposition.

##### Lemma 8.15

Let $\mathcal {D}$ be as defined in (176). Fix $\delta _0>0$, and some $\textbf{V}= \textbf{V}_0 + \textbf{V}_{-1}D_{t_*} \in \Psi _{{\operatorname {tan}}}^0(\Sigma ) + \Psi _{{\operatorname {tan}}}^{-1}(\Sigma )D_{t_*}$ with Schwartz kernel compactly supported on $\mathring{\Sigma }$, where $\textbf{V}_0\in \Psi _{{\operatorname {tan}}}^0(\Sigma )$ and $\textbf{V}_{-1}\in \Psi _{{\operatorname {tan}}}^{-1}(\Sigma )$ are Hermitian with respect to the $L^2(\mathring{\Sigma })$ Hermitian inner product^20^. Then for $\delta _r$ sufficiently small and *h* such that $h(t_*, \cdot )$ is supported on $\mathring{\Sigma }$ for all $t_*\ge 0$, we have that

207 $$\begin{aligned} \left\lvert \Re \left\langle \textbf{V}h, h\right\rangle _{L^2(\mathcal {D})}\right\rvert < \delta _0 \left\Vert h\right\Vert _{Mor_b(\mathcal {D})}^2 + \left\Vert \mathbb {L}_{g_b}h\right\Vert _{L^2(\mathcal {D})}^2 + {\int _{\mathring{\Sigma }_{t_*}}\widetilde{J}_0[h]}\Bigg \vert ^{t_*= {T_*}}_{t_*=0}, \end{aligned}$$

where

$$\begin{aligned} \left\lvert \int _{\mathring{\Sigma }_{t_*}}\widetilde{J}_0[h]\right\rvert \lesssim \left\Vert D_{t_*}h\right\Vert ^2_{\underline{H}^{-1}(\mathring{\Sigma }_{t_*})} + \delta _0\left\Vert h\right\Vert ^2_{Mor_b(\mathring{\Sigma }_{t_*})}. \end{aligned}$$

Moreover, if $h=e^{-\mathbbm {i}\sigma t_*}u$ for $\left\lvert \Im \sigma \right\rvert \le \textbf{M}$ for some $\textbf{M}>0$, and where $u:\Sigma \rightarrow \mathbb {C}^D$ is independent of $t_*$, then

208 $$\begin{aligned} \left\lvert \Re \left\langle \textbf{V}h, h\right\rangle _{L^2(\mathring{\Sigma })}\right\rvert < \delta _0 \left\Vert h\right\Vert _{\underline{Mor}_b(\mathring{\Sigma })}^2 + \left\Vert \mathbb {L}_{g_b}h\right\Vert _{\underline{L}^2(\mathring{\Sigma })}^2. \end{aligned}$$

##### Proof

Fix some auxiliary $\delta >0$. For $\textbf{V}\in \Psi _{{\operatorname {tan}}}^0$ we can conclude directly using Lemma 8.14, so it suffices to just consider $\textbf{V}= \textbf{V}_{-1}D_{t_*}\in \Psi _{{\operatorname {tan}}}^{-1}D_{t_*}$ with symbol $\textbf{v}_{-1}\sigma$. We now choose some auxiliary stationary Hermitian elliptic operator $\textbf{E}=\textbf{E}_{-1}\in \Psi _{{\operatorname {tan}}}^{-1}$ with symbol $e_{-1}$, and observe that

209 $$\begin{aligned} \begin{aligned} 2\Re \left\langle \Box _{g_{b}}h,A^{-1}\textbf{E}^2h\right\rangle _{L^2(\mathcal {D})} ={}&2\Re \left\langle \mathbb {L}_{g_b}h, A^{-1}\textbf{E}^2h\right\rangle _{L^2(\mathcal {D})} - 2\Re \left\langle \textbf{S}_bh,A^{-1}\textbf{E}^2h\right\rangle _{L^2(\mathcal {D})}\\&- 2\Re \left\langle \textbf{V}_bh, A^{-1}\textbf{E}^2h\right\rangle _{L^2(\mathcal {D})}. \end{aligned} \end{aligned}$$

Integrating by parts in $t_*$, we then have that

210 $$\begin{aligned} \begin{aligned} \Re \left\langle \Box _{g_{b}}h,A^{-1}\textbf{E}^2h\right\rangle _{L^2(\mathcal {D})} ={}&\left\Vert \textbf{E}D_{t_*}h\right\Vert ^2_{L^2(\mathcal {D})} + {\int _{\Sigma _{t_*}}\widetilde{J}_0[h]}\Bigg \vert ^{t_*={T_*}}_{t_*=0}\\&+ O\left( \left\Vert \textbf{E}D_{t_*}h\right\Vert _{L^2(\mathcal {D})}\left\Vert h\right\Vert _{L^2(\mathcal {D})} +\left\Vert h\right\Vert _{L^2(\mathcal {D})}^2\right) , \end{aligned} \end{aligned}$$

where

$$\begin{aligned} \int _{\Sigma _{t_*}}\widetilde{J}_0[h] \lesssim \left\Vert \textbf{E} D_{t_*}h\right\Vert ^2_{\underline{L}^2(\Sigma _{t_*})} + \left\Vert h\right\Vert ^2_{\underline{L}^2(\Sigma _{t_*})}. \end{aligned}$$

Then, we observe that

211 $$\begin{aligned} \begin{aligned}&\left\lvert \left\langle \mathbb {L}_{g_b}h, A^{-1}\textbf{E}^2h\right\rangle _{L^2(\mathcal {D})}\right\rvert + \left\lvert \left\langle \textbf{S}_bh,A^{-1}\textbf{E}^2h\right\rangle _{L^2(\mathcal {D})}\right\rvert + \left\lvert \left\langle \textbf{V}_bh, A^{-1}\textbf{E}^2h\right\rangle _{L^2(\mathcal {D})}\right\rvert \\ \le {}&\left\Vert \mathbb {L}_{g_b}h\right\Vert _{L^2(\mathcal {D})}^2 + O\left( \left\Vert \textbf{E}D_{t_*}h\right\Vert _{L^2(\mathcal {D})}\left\Vert h\right\Vert _{L^2(\mathcal {D})} +\left\Vert h\right\Vert _{L^2(\mathcal {D})}^2\right) . \end{aligned} \end{aligned}$$

As a result, by applying Cauchy-Schwarz, we can combine (209), (210), and (211) to see that

$$\begin{aligned} \left\Vert \textbf{E}D_{t_*}h\right\Vert _{L^2(\mathcal {D})}^2 \lesssim \left\Vert h\right\Vert _{L^2(\mathcal {D})}^2 + \left\Vert \mathbb {L}_{g_b}h\right\Vert _{L^2(\mathcal {D})}^2 + {\int _{\Sigma _{t_*}}\widetilde{J}_0[h]}\Bigg \vert ^{t_*={T_*}}_{t_*=0}. \end{aligned}$$

Since $\textbf{E}$ was an arbitrary elliptic operator, (207) follows from Lemma 8.14.

The proof of (208) is almost identical to the proof of (207), where we observe that $D_{t_*}(e^{-\mathbbm {i}\sigma t_*}u) = \sigma e^{-\mathbbm {i}\sigma t_*}u$. Repeating the proof of (207), we have that

$$\begin{aligned} \left\Vert \textbf{E}D_{t_*}h\right\Vert _{\underline{L}^2(\mathring{\Sigma })}^2 \lesssim \left\Vert h\right\Vert _{\underline{L}^2(\mathring{\Sigma })}^2 + \left\Vert \mathbb {L}_{g_b}h\right\Vert _{\underline{L}^2(\mathring{\Sigma })}^2 + \left\lvert 2\Im \sigma \left\langle \textbf{E}D_{t_*}h, \textbf{E}h\right\rangle _{\underline{L}^2(\mathring{\Sigma })}\right\rvert . \end{aligned}$$

Then, using Cauchy Schwarz (since we assumed $\left\lvert \Im \sigma \right\rvert \le \textbf{M}$, the Cauchy Schwarz constants can be chosen independently of the specific value of $\sigma$) and Lemma 8.14 yields the result. $\square$

At the level of first-order bulk terms that arise in the application of the divergence theorem, a skew-Hermitian operator can easily be handled via an integration by parts argument.

##### Lemma 8.16

Fix $\delta >0$. Let $\textbf{S}= \textbf{S}_0D_{t_*} + \textbf{S}_1$ be a first-order pseudo-differential operator with Schwartz kernel compactly supported on $\mathring{\Sigma }$ such that $\textbf{S}_i \in \Psi _{{\operatorname {tan}}}^i(\Sigma )$ are skew-Hermitian with respect to the $\underline{L}^2(\Sigma )$ inner product respectively.

Then, for *h* such that $h(t_*,\cdot )$ is compactly supported in $\mathring{\Sigma }$ for all $t_*\ge 0$, with $\mathring{\Sigma }$ defined as in (176),

212 $$\begin{aligned} \Re \left\langle \textbf{S}h, h\right\rangle _{L^2(\mathcal {D})} = {\Re \left\langle \textbf{S}_0h, h\right\rangle _{\underline{L}^2(\mathring{\Sigma }_{t_*})}}\Bigg \vert ^{t_*=T}_{t_*=0}. \end{aligned}$$

Similarly, if there exist $\sigma$ and *u* such that $h = e^{-\mathbbm {i}\sigma t_*}u$ where $\left\lvert \Im \sigma \right\rvert <\textbf{M}$ for some $\textbf{M}>0$, and $\textbf{S}$ is as specified above, then we also have that

213 $$\begin{aligned} \Re \left\langle \textbf{S}h, h\right\rangle _{L^2(\mathring{\Sigma })} = \Re \sigma \left\langle \textbf{S}_0h,h\right\rangle _{L^2(\mathring{\Sigma })} \lesssim \delta _0\left\Vert h\right\Vert _{Mor(\mathring{\Sigma })}^2 . \end{aligned}$$

##### Proof

The proof for (212) is a simple integration by parts exercise. The proof for (213) is also a simple integration by parts exercise, applying Lemma 8.14, where we note that $D_{t_*}h = \sigma h$. $\square$

Unfortunately, not all of the first-order bulk terms that we pick up in the application of the divergence theorem respect the symmetry assumptions of Lemma 8.16. For these first-order terms, we will need to use a more delicate argument relying on exchanging the lower-order nature of the bulk terms for some degeneracy at the trapped set. Since this argument essentially promotes these lower-order terms to become principal level errors, to apply the control effectively will require some additional smallness parameter.

##### Lemma 8.17

Let $\mathcal {D}$ be as defined in (176), and fix some $\delta _0>0$. Let $\textbf{S}\in \Psi _{{\operatorname {tan}}}^1(\Sigma ) + \Psi _{{\operatorname {tan}}}^{0}(\Sigma )D_{t_*}$ be a first order pseudo-differential operator with Schwartz kernel compactly supported on $\mathring{\Sigma }$ such that

$$\begin{aligned} \textbf{S}= \textbf{S}_1 + \textbf{S}_0D_{t_*}, \end{aligned}$$

where $\textbf{S}_1$ and $\textbf{S}_0$ are Hermitian with respect to the $L^2(\mathring{\Sigma })$ Hermitian inner product, such that the symbol $\textbf{s}$ of $\textbf{S}$ satisfies

$$\begin{aligned} \left\lvert \textbf{s}_i\right\rvert \le \varepsilon _{\textbf{S}}\left\lvert \zeta \right\rvert ^i. \end{aligned}$$

Then for $\varepsilon _{\textbf{S}}$ and $\delta _r$ sufficiently small,

214 $$\begin{aligned} \Re \left\langle \textbf{S}h, h\right\rangle _{L^2(\mathcal {D})} \le \delta _0 \left\Vert h\right\Vert _{Mor_b(\mathcal {D})}^2 + \left\Vert \mathbb {L}_{g_b}h\right\Vert _{L^2(\mathcal {D})}^2 + {\int _{\mathring{\Sigma }_{t_*}}\widetilde{J}[h]}\Bigg \vert _{t_*=0}^{t_*={T_*}}, \end{aligned}$$

where *h* is as specified in Theorem 8.9, and

215 $$\begin{aligned} \left\lvert \int _{\mathring{\Sigma }_{t_*}}\widetilde{J}[h]\right\rvert \lesssim \left\Vert D_{t_*}h\right\Vert ^2_{\underline{H}^{-1}(\mathring{\Sigma }_{t_*})} + \delta _0\left\Vert h\right\Vert _{\underline{Mor}_b(\mathring{\Sigma }_{t_*})}^2. \end{aligned}$$

##### Proof

Fix some arbitrary $\delta >0$. Observe that for *a* sufficiently small with respect to *M* and $\Lambda$, we have that $\sigma _1-\sigma _2$ is an elliptic operator. We now define

$$\begin{aligned} \mathring{r}_i {:=} \mathring{r}_b(\sigma _i, \eta _{\varphi }), \end{aligned}$$

and write

$$\begin{aligned} \textbf{s}= \textbf{s}_0\sigma + \textbf{s}_1,\qquad \textbf{s}_i\in S^i. \end{aligned}$$

We will handle the two terms separately. We first handle $\textbf{s}_0\sigma$. For this term, we define

$$\begin{aligned} \textbf{s}_0^{(2)} {:=} -\frac{\sigma _2(\sigma -\sigma _1)}{\sigma _1-\sigma _2}\textbf{s}_0,\qquad \textbf{s}_0^{(1)} {:=} \frac{\sigma _1(\sigma -\sigma _2)}{\sigma _1-\sigma _2}\textbf{s}_0, \end{aligned}$$

so that

$$\begin{aligned} \textbf{s}_0\sigma = \textbf{s}_0^{(1)} + \textbf{s}_0^{(2)}. \end{aligned}$$

Now, integrating by parts, we have that

216 $$\begin{aligned}&-\left\langle \textbf{S}_0\partial _{t_*} h, h\right\rangle _{L^2(\mathcal {D})}\nonumber \\ ={}&\sum _{i=1,2}\left\langle (r-\mathring{r}_i)\operatorname {Op}\left( \textbf{s}_0^{(i)}\right) \circ \partial _r h, h\right\rangle _{L^2(\mathcal {D})} + \sum _{i=1,2}\left\langle (r-\mathring{r}_i)\operatorname {Op}\left( \textbf{s}_0^{(i)}\right) h, \partial _r h\right\rangle _{L^2(\mathcal {D})}\nonumber \\&+ \sum _{i=1,2} \left\langle (r-\mathring{r}_i)\left[ \partial _r, \operatorname {Op}\left( \textbf{s}_0^{(i)}\right) \right] h, h\right\rangle _{L^2(\mathcal {D})} + Err[h], \end{aligned}$$

where the error terms are lower-order terms respecting the degeneracy in the Morawetz norm, which for $\delta _r$ sufficiently small, using Lemma 8.14, satisfies

$$\begin{aligned} \left\lvert Err[h]\right\rvert \lesssim \delta \left\Vert h\right\Vert _{Mor_b(\mathcal {D})}^2. \end{aligned}$$

We treat each of the terms on the right-hand side of (222) individually. First, we use that up to lower-order terms,

$$\begin{aligned} \left( (r-\mathring{r}_i)\operatorname {Op}\left( \textbf{s}_0^{(i)})\right) \right) ^* = (r-\mathring{r}_i)\operatorname {Op}\left( \textbf{s}_0^{(i)}\right) - \left[ (r-\mathring{r}_i), \operatorname {Op}\left( \textbf{s}_0^{(i)}\right) \right] , \end{aligned}$$

so that using integrating by parts, taking advantage of the fact that

217 $$\begin{aligned} \partial _r(r-\mathring{r}_i) = 1 + \Psi _{{\operatorname {tan}}}^{-1}, \end{aligned}$$

and using Lemma 8.15 and Cauchy-Schwarz,

$$\begin{aligned}&\left\lvert \Re \left\langle (r-\mathring{r}_i)\operatorname {Op}\left( \textbf{s}_0^{(i)}\right) \circ \partial _r h, h\right\rangle _{L^2(\mathcal {D})}\right\rvert \\ <{}&\left\lvert \Re \left\langle \partial _r h, (r-\mathring{r}_i)\operatorname {Op}\left( \textbf{s}_0^{(i)}\right) h\right\rangle _{L^2(\mathcal {D})}\right\rvert + Err_{\mathcal {D}}[h] + {Err_{\mathring{\Sigma }}[h]}\Bigg \vert _{t_*=0}^{t_*={T_*}}, \end{aligned}$$

where

$$\begin{aligned} \left\lvert Err_{\mathcal {D}}[h]\right\rvert \!\lesssim \! \delta \left\Vert h\right\Vert _{Mor_b(\mathcal {D})}^2 \!+\! \left\Vert \mathbb {L}_{g_b}h\right\Vert _{L^2(\mathcal {D})}^2, \quad \left\lvert Err_{\mathring{\Sigma }}\right\rvert \!\lesssim \! \left\Vert D_{t_*}h\right\Vert ^2_{\underline{H}^{-1}(\Sigma _{t_*})} \!+\!\delta \left\Vert h\right\Vert _{\underline{Mor}_b(\Sigma _{\mathring{\Sigma }})}^2. \end{aligned}$$

Then, since both $\partial _r$ and $(r-\mathring{r}_i)$ have symbols that vanish at the trapped set, we have that

$$\begin{aligned} \left\lvert \Re \left\langle \partial _r h, (r-\mathring{r}_i)\operatorname {Op}\left( \textbf{s}_0^{(i)}\right) h\right\rangle _{L^2(\mathcal {D})}\right\rvert \lesssim \varepsilon _{\textbf{S}}\left\Vert h\right\Vert _{Mor_b(\mathcal {D})}^2, \end{aligned}$$

which is controlled for $\varepsilon _{\textbf{S}}$ sufficiently small. Similarly, we have that

$$\begin{aligned} \left\Vert (r-\mathring{r}_i)\operatorname {Op}\left( \textbf{s}_0^{(i)}\right) h\right\Vert _{L^2(\mathcal {D})} \lesssim \varepsilon _{\textbf{S}}\left\Vert h\right\Vert _{Mor_b(\mathcal {D})}, \end{aligned}$$

which is controlled for $\varepsilon _{\textbf{S}}$ sufficiently small. Then using Cauchy-Schwarz and Lemma 8.21, we have that for $\delta _r$ sufficiently small,

$$\begin{aligned} \sum _{i=1,2}\left\lvert \left\langle (r-\mathring{r}_2)(r-\mathring{r}_1)\operatorname {Op}\left( \textbf{s}_{0}^{(i)}\right) h,h\right\rangle _{L^2(\mathcal {D})}\right\rvert \lesssim \delta \left\Vert h\right\Vert _{Mor_b(\mathcal {D})}^2. \end{aligned}$$

To handle the commutator term in (216), we first observe that

$$\begin{aligned} \left\{ \xi , \textbf{s}_0^{(2)}\right\}&= -(\sigma -\sigma _1)\partial _r\left( \frac{\sigma _2\textbf{s}_0}{\sigma _1-\sigma _2}\right) +\frac{ \textbf{s}_0\sigma _2\partial _r\sigma _1}{\sigma _1-\sigma _2},\\ \left\{ \xi , \textbf{s}_0^{(1)}\right\}&= (\sigma -\sigma _2)\partial _r\left( \frac{\sigma _1\textbf{s}_0}{\sigma _1-\sigma _2}\right) -\frac{ \textbf{s}_0\sigma _1\partial _r\sigma _2}{\sigma _1-\sigma _2}, \end{aligned}$$

Thus, we can decompose

218 $$\begin{aligned}&\sum _{i=1,2} \left\langle (r-\mathring{r}_i)\left[ \partial _r, \operatorname {Op}\left( \textbf{s}_0^{(i)}\right) \right] h, h\right\rangle _{L^2(\mathcal {D})}\nonumber \\ ={}&\left\langle (r-\mathring{r}_1)\operatorname {Op}\left( (\sigma -\sigma _2)\partial _r\left( \frac{\sigma _1\textbf{s}_0}{\sigma _1-\sigma _2}\right) \right) h,h\right\rangle _{L^2(\mathcal {D})} \nonumber \\&- \left\langle (r-\mathring{r}_1)\operatorname {Op}\left( \frac{ \textbf{s}_0\sigma _1\partial _r\sigma _2}{\sigma _1-\sigma _2} \right) h,h\right\rangle _{L^2(\mathcal {D})}\nonumber \\&- \left\langle (r-\mathring{r}_2)\operatorname {Op}\left( (\sigma -\sigma _1)\partial _r\left( \frac{\sigma _2\textbf{s}_0}{\sigma _1-\sigma _2}\right) \right) h,h\right\rangle _{L^2(\mathcal {D})} \nonumber \\&+ \left\langle (r-\mathring{r}_2)\operatorname {Op}\left( \frac{ \textbf{s}_0\sigma _2\partial _r\sigma _1}{\sigma _1-\sigma _2} \right) h,h\right\rangle _{L^2(\mathcal {D})}. \end{aligned}$$

We see that the first terms in each line of the right-hand side of (218) are controlled by the Morawetz norm. Thus, using Cauchy-Schwarz and Lemma 8.14, we have that for $\delta _r$ sufficiently small,

$$\begin{aligned} \delta \left\Vert h\right\Vert _{Mor_b(\mathcal {D})}^2\gtrsim {}&\left\lvert \left\langle (r-\mathring{r}_1)\operatorname {Op}\left( (\sigma -\sigma _2)\partial _r\left( \frac{\sigma _1\textbf{s}_0}{\sigma _1-\sigma _2}\right) \right) h,h\right\rangle _{L^2(\mathcal {D})}\right\rvert \\&+ \left\lvert \left\langle (r-\mathring{r}_2)\operatorname {Op}\left( (\sigma -\sigma _1)\partial _r\left( \frac{\sigma _2\textbf{s}_0}{\sigma _1-\sigma _2}\right) \right) h,h\right\rangle _{L^2(\mathcal {D})}\right\rvert . \end{aligned}$$

It remains to handle the second terms in each line of the right-hand side of (218). Without loss of generality, we handle $(\sigma _1-\sigma _2)^{-1}{\textbf{s}_0\sigma _2\partial _r\sigma _1}$ since $(\sigma _1-\sigma _2)^{-1}{\textbf{s}_0\sigma _1\partial _r\sigma _2}$ is handled identically. To this end, define

$$\begin{aligned} \tilde{\textbf{s}}_{0,2} {:=} \frac{\textbf{s}_0\sigma _2\partial _r\sigma _1}{\sigma _1-\sigma _2}\in S^1,\qquad \tilde{\textbf{s}}_{0,2}^{(2)} {:=} -\frac{\sigma -\sigma _1}{\sigma _1-\sigma _2} \tilde{\textbf{s}}_{0,2},\qquad \tilde{\textbf{s}}_{0,2}^{(1)} {:=} \frac{\sigma -\sigma _2}{\sigma _1-\sigma _2} \tilde{\textbf{s}}_{0,2}, \end{aligned}$$

so that

$$\begin{aligned} \sum _{i=1,2}\tilde{\textbf{s}}_{0,2}^{(i)} = \tilde{\textbf{s}}_{0,2}. \end{aligned}$$

Then we have that

$$\begin{aligned} -\left\langle (r-\mathring{r}_2)\operatorname {Op}\left( \tilde{\textbf{s}}_{0,2}\right) h,h\right\rangle _{L^2(\mathcal {D})} = -\sum _{i=1,2}\left\langle (r-\mathring{r}_2) \operatorname {Op}\left( \tilde{\textbf{s}}_{0,2}^{(i)}\right) h,h\right\rangle _{L^2(\mathcal {D})}. \end{aligned}$$

Then observe that by construction,

$$\begin{aligned} \left\Vert (r-\mathring{r}_2)\operatorname {Op}\left( \tilde{\textbf{s}}_{0,2}^{(2)}\right) h\right\Vert _{L^2(\mathcal {D})} \lesssim \left\Vert h\right\Vert _{Mor_b(\mathcal {D})}. \end{aligned}$$

Thus, it suffices to control $\left\langle (r-\mathring{r}_2)\operatorname {Op}\left( \tilde{\textbf{s}}_{0,2}^{(1)}\right) h,h\right\rangle _{L^2(\mathcal {D})}$. To this end, we use integration by parts, recalling (217), to write that,

219 $$\begin{aligned} \begin{aligned}&\!-\!\left\langle (r\!-\!\mathring{r}_2)\operatorname {Op}\left( \tilde{\textbf{s}}_{0,2}^{(1)}\right) h,h\right\rangle _{L^2(\mathcal {D})}\\ \!=\!{}&\left\langle (r\!-\!\mathring{r}_1)(r\!-\!\mathring{r}_2)\operatorname {Op}\left( \tilde{\textbf{s}}_{0,2}^{(1)}\right) \circ \partial _r h,h\right\rangle _{L^2(\mathcal {D})} \!+\! \left\langle (r\!-\!\mathring{r}_1)(r\!-\!\mathring{r}_2)\operatorname {Op}\left( \tilde{\textbf{s}}_{0,2}^{(1)}\right) h,\partial _rh\right\rangle _{L^2(\mathcal {D})}\\&\!+ \!\left\langle (r\!-\!\mathring{r}_1)\operatorname {Op}\left( \tilde{\textbf{s}}_{0,2}^{(1)}\right) h,h\right\rangle _{L^2(\mathcal {D})} \!+\! \left\langle (r\!-\!\mathring{r}_1)(r\!-\!\mathring{r}_2)\left[ \partial _r,\operatorname {Op}\left( \tilde{\textbf{s}}_{0,2}^{(1)}\right) \right] h,h\right\rangle _{L^2(\mathcal {D})}. \end{aligned} \end{aligned}$$

Observing again that $(r-\mathring{r}_1)(r-\mathring{r}_2)\operatorname {Op}\left( \tilde{\textbf{s}}_{0,2}^{(1)}\right)$ is Hermitian up to a $\Psi _{{\operatorname {tan}}}^0 + \Psi _{{\operatorname {tan}}}^{-1}D_{t_*}$ term, we control the first three terms on the right-hand side of (219) by a combination of integration by parts, Cauchy-Schwarz, and Lemma 8.15 after taking $\varepsilon _{\textbf{S}}$ and $\delta _r$ sufficiently small. To handle the final commutator term, we again observe that

$$\begin{aligned} \left\{ \xi , \tilde{\textbf{s}}_{0,2}^{(1)}\right\} = (\sigma -\sigma _2)\partial _r\left( \frac{\tilde{\textbf{s}}_{0,2}}{\sigma _1-\sigma _2}\right) - \tilde{\textbf{s}}_{0,2}\frac{\partial _r\sigma _2}{\sigma _1-\sigma _2}. \end{aligned}$$

Again we can thus decompose

220 $$\begin{aligned}&\left\langle (r-\mathring{r}_1)(r-\mathring{r}_2)\left[ \partial _r,\operatorname {Op}\left( \tilde{\textbf{s}}_{0,2}^{(1)}\right) \right] h,h\right\rangle _{L^2(\mathcal {D})}\nonumber \\ ={}&\left\langle (r-\mathring{r}_1)(r-\mathring{r}_2)\operatorname {Op}\left( (\sigma -\sigma _2)\partial _r\left( \frac{\tilde{\textbf{s}}_{0,2}}{\sigma _1-\sigma _2}\right) \right) h,h\right\rangle _{L^2(\mathcal {D})}\nonumber \\&- \left\langle (r-\mathring{r}_1)(r-\mathring{r}_2)\operatorname {Op}\left( \tilde{\textbf{s}}_{0,2}\frac{\partial _r\sigma _2}{\sigma _1-\sigma _2}\right) h,h\right\rangle _{L^2(\mathcal {D})}. \end{aligned}$$

Once again, we see that the first term on the right-hand side of (220) is controlled directly by the Morawetz norm, using Lemma 8.14 and taking $\delta _r$ sufficiently small. To control the second term on the right-hand side of (220), we partition one final time to define

$$\begin{aligned} \tilde{\textbf{s}}_{0,2,1}:\!=\! \tilde{\textbf{s}}_{0,2}\frac{\partial _r\sigma _2}{\sigma _1\!-\!\sigma _2}\in S^1,\quad \tilde{\textbf{s}}_{0,2,1}^{(2)}:\!=\! -\!\frac{\sigma -\sigma _1}{\sigma _1\!-\!\sigma _2}\tilde{\textbf{s}}_{0,2,1},\quad \tilde{\textbf{s}}_{0,2,1}^{(1)}:\!=\! \frac{\sigma -\sigma _2}{\sigma _1\!-\!\sigma _2}\tilde{\textbf{s}}_{0,2,1}, \end{aligned}$$

so that

$$\begin{aligned} \tilde{\textbf{s}}_{0,2,1}\!=\!\tilde{\textbf{s}}_{0,2,1}^{(2)}\!+\!\tilde{\textbf{s}}_{0,2,1}^{(1)}. \end{aligned}$$

Then we have that

$$\begin{aligned} \left\langle (r\!-\!\mathring{r}_2)(r\!-\!\mathring{r}_1)\operatorname {Op}\left( \tilde{\textbf{s}}_{0,2,1}\right) h,h\right\rangle _{L^2(\mathcal {D})} \!=\! \sum _{i=1,2}\left\langle (r\!-\!\mathring{r}_2)(r\!-\!\mathring{r}_1)\operatorname {Op}\left( \tilde{\textbf{s}}_{0,2,1}^{(i)}\right) h,h\right\rangle _{L^2(\mathcal {D})}. \end{aligned}$$

But by construction,

$$\begin{aligned}&\left\Vert (r-\mathring{r}_2)(r-\mathring{r}_1)\operatorname {Op}\left( \tilde{\textbf{s}}_{0,2,1}^{(1)}\right) h\right\Vert _{L^2(\mathcal {D})} + \left\Vert (r-\mathring{r}_2)(r-\mathring{r}_1)\operatorname {Op}\left( \tilde{\textbf{s}}_{0,2,1}^{(2)}\right) h\right\Vert _{L^2(\mathcal {D})} \\ \lesssim {}&\left\Vert h\right\Vert _{Mor_b(\mathcal {D})}. \end{aligned}$$

As a result, using Cauchy-Schwarz, we have that

$$\begin{aligned} \sum _{i=1,2}\left\lvert \left\langle (r-\mathring{r}_2)(r-\mathring{r}_1)\operatorname {Op}\left( \tilde{\textbf{s}}_{0,2,1}^{(i)}\right) h,h\right\rangle _{L^2(\mathcal {D})}\right\rvert < \delta \left\Vert h\right\Vert _{Mor_b(\mathcal {D})}^2. \end{aligned}$$

Since $\delta$ was arbitrary, we can choose it sufficiently small so that

221 $$\begin{aligned} \left\lvert \Re \left\langle \textbf{S}_0D_{t_*} h, h\right\rangle _{L^2(\mathcal {D})}\right\rvert < \frac{\delta _0}{2} \left\Vert h\right\Vert _{Mor_b(\mathcal {D})}^2. \end{aligned}$$

We now move on to handling $\Re \left\langle \textbf{S}_1 h, h\right\rangle _{L^2(\mathcal {D})}$. The main idea will be the same as when handling $\textbf{S}_0\partial _{t_*}$. To this end, we define

$$\begin{aligned} \textbf{s}_1^{(2)}{:=} -\frac{\sigma -\sigma _1}{\sigma _1-\sigma _2}\textbf{s},\qquad \textbf{s}_1^{(1)}{:=} \frac{\sigma -\sigma _2}{\sigma _1-\sigma _2}\textbf{s}, \end{aligned}$$

so that

$$\begin{aligned} \textbf{s}= \textbf{s}_1^{(1)}+\textbf{s}_1^{(2)}. \end{aligned}$$

Integrating by parts, we have that

222 $$\begin{aligned}&-\left\langle \operatorname {Op}\left( \textbf{s}_1^{(i)}\right) h, h\right\rangle _{L^2(\mathcal {D})} \nonumber \\ ={}&\left\langle (r-\mathring{r}_i)\operatorname {Op}\left( \textbf{s}_1^{(i)}\right) \circ \partial _r h, h\right\rangle _{L^2(\mathcal {D})} + \left\langle (r-\mathring{r}_i)\operatorname {Op}\left( \textbf{s}_{1}^{(i)}\right) h, \partial _r h\right\rangle _{L^2(\mathcal {D})}\nonumber \\&+ \left\langle (r-\mathring{r}_i)\left[ \partial _r, \operatorname {Op}\left( \textbf{s}_1^{(i)}\right) \right] h, h\right\rangle _{L^2(\mathcal {D})} + Err[h], \end{aligned}$$

where for $\delta _r$ sufficiently small,

$$\begin{aligned} \left\lvert Err[h]\right\rvert \lesssim \delta \left\Vert h\right\Vert _{Mor_b(\mathcal {D})}^2. \end{aligned}$$

We treat each of the terms on the right-hand side of (222) individually. Observing that

$$\begin{aligned} \left( (r-\mathring{r}_i)\operatorname {Op}\left( \textbf{s}_1^{(i)}\right) \right) ^* = (r-\mathring{r}_i)\operatorname {Op}\left( \textbf{s}_1^{(i)}\right) + \Psi _{{\operatorname {tan}}}^0(\Sigma ) + \Psi _{{\operatorname {tan}}}^{-1}(\Sigma )\partial _{t_*}, \end{aligned}$$

we have by integration by parts that

$$\begin{aligned}&\left\langle (r-\mathring{r}_i)\operatorname {Op}\left( \textbf{s}_1^{(i)}\right) \circ \partial _r h, h\right\rangle _{L^2(\mathcal {D})}\\ ={}&\left\langle \partial _r h, (r-\mathring{r}_i)\operatorname {Op}\left( \textbf{s}_1^{(i)}\right) h\right\rangle _{L^2(\mathcal {D})} + Err_{\mathcal {D}}[h] + {Err_{\mathring{\Sigma }}[h]}\Bigg \vert _{t_*=0}^{t_*={T_*}}, \end{aligned}$$

where by Cauchy-Schwarz, and Lemma 8.15 that for sufficiently small $\delta _r$,

$$\begin{aligned} \left\lvert Err_{\mathcal {D}}[h]\right\rvert \lesssim \delta \left\Vert h\right\Vert ^2_{Mor_b(\mathcal {D})} \!+\! \left\Vert \mathbb {L}_{g_b}h\right\Vert _{L^2(\mathcal {D})}^2, \quad \left\lvert Err_{\mathring{\Sigma }}\right\rvert \lesssim \left\Vert D_{t_*}h\right\Vert ^2_{\underline{H}^{-1}(\Sigma _{t_*})} \!+\!\delta \left\Vert h\right\Vert ^2_{\underline{Mor}_b(\mathring{\Sigma })}. \end{aligned}$$

Then from Lemma 8.11, we have that

$$\begin{aligned} \left\lvert \left\langle \partial _r h, (r-\mathring{r}_i)\operatorname {Op}\left( \textbf{s}_1^{(i)}\right) h\right\rangle _{L^2(\mathcal {D})}\right\rvert \lesssim \varepsilon _{\textbf{S}}\left\Vert h\right\Vert _{Mor_b(\mathcal {D})}^2, \end{aligned}$$

which is clearly controlled for $\varepsilon _{\textbf{S}}$ sufficiently small.

To handle the commutator term in (222), we proceed as previously and observe that (without loss of generality, we will just consider the $i=1$ case)

$$\begin{aligned} \left\langle (r\!-\!\mathring{r}_1)\left[ \partial _r, \operatorname {Op}\left( \textbf{s}_1^{(1)}\right) \right] h, h\right\rangle _{L^2(\mathcal {D})} \!=\!{}&\left\langle (r\!-\!\mathring{r}_1)\operatorname {Op}\left( (\sigma \!-\!\sigma _2)\partial _r\left( \frac{\textbf{s}_1}{\sigma _1\!-\!\sigma _2}\right) \right) h, h\right\rangle _{L^2(\mathcal {D})}\\&\!- \!\left\langle (r\!-\!\mathring{r}_1)\operatorname {Op}\left( \frac{\textbf{s}_1\partial _r\sigma _2}{\sigma _1\!-\!\sigma _2}\right) h, h\right\rangle _{L^2(\mathcal {D})}, \end{aligned}$$

where it is clear that the first term on the right-hand side is well-controlled using Lemma 8.14, Cauchy-Schwarz, and choosing $\delta _r$ sufficiently small as before. To handle the second term, we can define

$$\begin{aligned} \tilde{\textbf{s}}_{1,1}{:=} \frac{\textbf{s}_1\partial _r\sigma _2}{\sigma _1-\sigma _2}\in S^1,\qquad \tilde{\textbf{s}}_{1,1}^{(2)} {:=} -\frac{\sigma -\sigma _1}{\sigma _1-\sigma _2}\tilde{\textbf{s}}_{1,1},\qquad \tilde{\textbf{s}}_{1,1}^{(1)} {:=} \frac{\sigma -\sigma _2}{\sigma _1-\sigma _2}\tilde{\textbf{s}}_{1,1} \end{aligned}$$

so that

$$\begin{aligned} \tilde{\textbf{s}}_{1,1} = \sum _{i=1,2}\tilde{\textbf{s}}_{1,1}^{(i)}. \end{aligned}$$

and

$$\begin{aligned} \left\Vert (r-\mathring{r}_i)\left[ \partial _r, \operatorname {Op}\left( \textbf{s}_1^{(i)}\right) \right] h\right\Vert _{L^2(\mathcal {D})} \lesssim \left\Vert h\right\Vert _{Mor_b(\mathcal {D})}. \end{aligned}$$

Then, we see that

$$\begin{aligned}&- \left\langle (r-\mathring{r}_1)\operatorname {Op}\left( \tilde{\textbf{s}}_{1,1}\right) h, h\right\rangle _{L^2(\mathcal {D})}\\ ={}&- \left\langle (r-\mathring{r}_1)\operatorname {Op}\left( \tilde{\textbf{s}}_{1,1}^{(1)}\right) h, h\right\rangle _{L^2(\mathcal {D})} - \left\langle (r-\mathring{r}_1)\operatorname {Op}\left( \tilde{\textbf{s}}_{1,1}^{(2)}\right) h, h\right\rangle _{L^2(\mathcal {D})}, \end{aligned}$$

where it is clear by construction that the first term on the right-hand side respects the degeneracy in the Morawetz norm and thus can be controlled by the Morawetz norm using Cauchy-Schwarz. To control the second term on the right-hand side, we repeat the integration by parts argument above to see that

223 $$\begin{aligned} \begin{aligned}&-\left\langle (r-\mathring{r}_1)\operatorname {Op}\left( \tilde{\textbf{s}}_{1,1}^{(2)}\right) h,h\right\rangle _{L^2(\mathcal {D})}\\ ={}&\left\langle (r-\mathring{r}_2)(r-\mathring{r}_1)\operatorname {Op}\left( \tilde{\textbf{s}}_{1,1}^{(2)}\right) \circ \partial _r h,h\right\rangle _{L^2(\mathcal {D})} \\&+ \left\langle (r-\mathring{r}_2)(r-\mathring{r}_1)\operatorname {Op}\left( \tilde{\textbf{s}}_{1,1}^{(2)}\right) h,\partial _rh\right\rangle _{L^2(\mathcal {D})}\\&+ \left\langle (r-\mathring{r}_2)\operatorname {Op}\left( \tilde{\textbf{s}}_{1,1}^{(2)}\right) h,h\right\rangle _{L^2(\mathcal {D})} \\&+ \left\langle (r-\mathring{r}_2)(r-\mathring{r}_1)\left[ \partial _r,\operatorname {Op}\left( \tilde{\textbf{s}}_{1,1}^{(2)}\right) \right] h,h\right\rangle _{L^2(\mathcal {D})}. \end{aligned} \end{aligned}$$

Again, using that

$$\begin{aligned} \left( (r-\mathring{r}_2)(r-\mathring{r}_1)\operatorname {Op}\left( \tilde{\textbf{s}}_{1,1}^{(2)}\right) \right) ^* = (r-\mathring{r}_2)(r-\mathring{r}_1)\operatorname {Op}\left( \tilde{\textbf{s}}_{1,1}^{(2)}\right) + \Psi _{{\operatorname {tan}}}^0(\Sigma ) + \Psi _{{\operatorname {tan}}}^{-1}(\Sigma )D_{t_*}, \end{aligned}$$

we have from integrating by parts, Cauchy-Schwarz, Lemma 8.15, that the first three terms are controlled, taking $\delta _r$ to be sufficiently small. To handle the last term on the right-hand side of (223), we see that

$$\begin{aligned}&\left\langle (r-\mathring{r}_2)(r-\mathring{r}_1)\left[ \partial _r,\operatorname {Op}\left( \tilde{\textbf{s}}_{1,1}^{(2)}\right) \right] h,h\right\rangle _{L^2(\mathcal {D})}\\ = {}&-\left\langle (r-\mathring{r}_2)(r-\mathring{r}_1)\operatorname {Op}\left( (\sigma -\sigma _1)\partial _r\left( \frac{\tilde{\textbf{s}}_{1,1}}{\sigma _1-\sigma _2}\right) \right) h,h\right\rangle _{L^2(\mathcal {D})}\\&+ \left\langle (r-\mathring{r}_2)(r-\mathring{r}_1)\operatorname {Op}\left( \frac{\tilde{\textbf{s}}_{1,1}\partial _r\sigma _1}{\sigma _1-\sigma _2} \right) h,h\right\rangle _{L^2(\mathcal {D})}, \end{aligned}$$

where the first term on the right-hand side is handled directly as before using Cauchy-Schwarz, and Lemma 8.14, taking $\delta _r$ to be sufficiently small. The second term on the right-hand side is handled by a final decomposition of

$$\begin{aligned} \tilde{\textbf{s}}_{1,1,2}{:=} \tilde{\textbf{s}}_{1,1}\frac{\partial _r\sigma _1}{\sigma _1-\sigma _2}\in S^1,\quad \tilde{\textbf{s}}_{1,1,2}^{(2)}{:=} -\frac{\sigma -\sigma _1}{\sigma _1-\sigma _2}\tilde{\textbf{s}}_{1,1,2},\quad \tilde{\textbf{s}}_{1,1,2}^{(1)}{:=} \frac{\sigma -\sigma _2}{\sigma _1-\sigma _2}\tilde{\textbf{s}}_{1,1,2}. \end{aligned}$$

Then it is apparent that

$$\begin{aligned} \sum _{i=1,2}\left\Vert (r-\mathring{r}_1)(r-\mathring{r}_2)\operatorname {Op}\left( \tilde{\textbf{s}}_{1,1,2}^{(i)}\right) h\right\Vert _{Mor_b(\mathcal {D})} \lesssim \left\Vert h\right\Vert _{Mor_b(\mathcal {D})}, \end{aligned}$$

and we conclude the proof of Lemma 8.17 by using Cauchy-Schwarz and taking $\delta _r$ and $\delta$ sufficiently small. $\square$

We can achieve a similar control for the lower-order boundary terms that come out of the integration by parts argument.

##### Lemma 8.18

Let $\mathring{\Sigma }$ be as defined in (176), and fix some $\delta _0>0$. Let $\textbf{S}\in \Psi _{{\operatorname {tan}}}^1(\Sigma ) + \Psi _{{\operatorname {tan}}}^{0}(\Sigma )D_{t_*}$ be a first order pseudo-differential operator with Schwartz kernel compactly supported on $\mathring{\Sigma }$ such that

$$\begin{aligned} \textbf{S}= \textbf{S}_1 + \textbf{S}_0D_{t_*}, \end{aligned}$$

where $\textbf{S}_1$ and $\textbf{S}_0$ are Hermitian with respect to the $L^2(\mathring{\Sigma })$ norm respectively, such that the symbols $\textbf{s}_i$ of $\textbf{S}_i$ satisfy

$$\begin{aligned} \left\lvert \textbf{s}_i\right\rvert \le \varepsilon _{\textbf{S}}\left\lvert \zeta \right\rvert ^i. \end{aligned}$$

Then for $\varepsilon _{\textbf{S}}$ and $\delta _r$ sufficiently small, and for $h=e^{-\mathbbm {i}\sigma t_*}u$ where $\left\lvert \Im \sigma \right\rvert <\textbf{M}$ for some $\textbf{M}>0$, and where *u* is independent of $t_*$,

224 $$\begin{aligned} \left\lvert \Re \left\langle \textbf{S}h, h\right\rangle _{L^2(\mathring{\Sigma })}\right\rvert \le \delta _0 \left\Vert h\right\Vert _{\underline{Mor}_b(\mathring{\Sigma })}^2 + C \left\Vert \mathbb {L}_{g_b}h\right\Vert _{\underline{L}^2(\mathring{\Sigma })}^2 , \end{aligned}$$

where *h* is as specified in Theorem 8.9.

##### Proof

The proof follows exactly as the proof of Lemma 8.17, observing that $D_{t_*}e^{-\mathbbm {i}\sigma t_*}u = \sigma u$. $\square$

Using Lemma 8.18, we can actually apply the control in Lemma 8.17 to operators in $\Psi _{{\operatorname {tan}}}^{-1}(\Sigma )D_{t_*}^2 + \Psi _{{\operatorname {tan}}}^0(\Sigma )D_{t_*} + \Psi _{{\operatorname {tan}}}^1(\Sigma )$ with the aid of some lower-order Lagrangian correction.

##### Lemma 8.19

Fix $\delta _0>0$ and let

$$\begin{aligned} \textbf{S}= \textbf{S}_{-1}D_{t_*}^2 + \textbf{S}_{0}D_{t_*} + \textbf{S}_{1}, \end{aligned}$$

with Schwartz kernel compactly supported on $\mathring{\Sigma }$ and where $\textbf{S}_{i} \in \Psi _{{\operatorname {tan}}}^i(\Sigma )$ and

$$\begin{aligned} \left\lvert \textbf{s}_{i}\right\rvert \le \varepsilon _{\textbf{S}}\left( \left\lvert \xi \right\rvert ^i + \left\lvert \eta \right\rvert ^i\right) ,\qquad i=-1,0,1, \end{aligned}$$

where $\textbf{s}_i$ is the symbol of $\textbf{S}_i$. Then, there exists some Hermitian $\tilde{r}\in \Psi _{{\operatorname {tan}}}^{-1}(\Sigma )$ with principal symbol $\tilde{\mathfrak {r}} \in S_{{\operatorname {tan}}}^{-1}(\Sigma )$ and sufficiently small *a*, and $\delta _r$, so that for $\varepsilon _{\textbf{S}}$ sufficiently small,

$$\begin{aligned} \left\lvert \Re \left\langle \textbf{S}h, h\right\rangle _{L^2(\mathcal {D})} \!-\! 2\Re \left\langle \Box _{g_{b}}h, \tilde{r} h\right\rangle _{L^2(\mathcal {D})}\right\rvert \!\le \! \delta _0 \left\Vert h\right\Vert _{Mor_b(\mathcal {D})}^2 \!+\! \left\Vert \mathbb {L}_{g_b}h\right\Vert _{L^2(\mathcal {D})}^2 \!+\! {\int _{\mathring{\Sigma }_{t_*}}\tilde{J}[h]}\Bigg \vert ^{t_*={T_*}}_{t_*=0}, \end{aligned}$$

where

$$\begin{aligned} \left\lvert \int _{\mathring{\Sigma }_{t_*}}\tilde{J}[h]\right\rvert \lesssim \left\Vert D_{t_*}h\right\Vert ^2_{\underline{H}^{-1}(\Sigma _{t_*})} + \delta _0 \left\Vert h\right\Vert _{\underline{Mor}_b(\Sigma _{t_*})}^2. \end{aligned}$$

##### Proof

In view of Lemma 8.17, it suffices to consider the case where $\textbf{S}= \textbf{S}^{-1}\partial _{t_*}^2$. Denoting $\textbf{s}_{-1}$ as the symbol of $\textbf{S}_{-1}$,

$$\begin{aligned} \textbf{s}_{-1}\sigma ^2 = \textbf{s}_{-1}(\sigma -\sigma _1)(\sigma -\sigma _2) - \textbf{s}_{-1}\sigma _1\sigma _2 + \textbf{s}_{-1}\sigma (\sigma _1+\sigma _2). \end{aligned}$$

Letting $\tilde{r} = -G_b(dt_*,dt_*)\textbf{s}_{-1}$, we have that

$$\begin{aligned} 2\Re \left\langle \Box _{g_{b}}h, \tilde{r}h\right\rangle _{L^2(\mathcal {D})} = \left\langle \left( \Box _{g_{b}}\tilde{r} + \tilde{r}\Box _{g_{b}}\right) h, h\right\rangle _{L^2(\mathcal {D})} + {2\Re \left\langle n_{\Sigma }h, \tilde{r} h\right\rangle _{L^2(\mathring{\Sigma }_{t_*})}}\Bigg \vert ^{t_*={T_*}}_{t_*=0}, \end{aligned}$$

so that up to lower order terms,

225 $$\begin{aligned} \begin{aligned}&\Re \left\langle \textbf{S}h, h\right\rangle _{L^2(\mathcal {D})} - 2\Re \left\langle \Box _{g_{b}}h, \tilde{r} h\right\rangle _{L^2(\mathcal {D})}\\ ={}&\left\langle \operatorname {Op}\left( \textbf{s}_{-1}\sigma (\sigma _1+\sigma _2) - \textbf{s}_{-1}\sigma _1\sigma _2\right) h,h\right\rangle _{L^2(\mathcal {D})} + {2\Re \left\langle n_{\Sigma }h, \tilde{r} h\right\rangle _{L^2(\mathring{\Sigma }_{t_*})}}\Bigg \vert ^{t_*={T_*}}_{t_*=0}. \end{aligned} \end{aligned}$$

Using Lemma 8.18, we see that for $\varepsilon _{\textbf{S}}$ sufficiently small and $\delta _r$ sufficiently small,

$$\begin{aligned} \Re \left\langle n_{\Sigma }h, \tilde{r} h\right\rangle _{L^2(\mathring{\Sigma }_{t_*})}< \frac{1}{4}\delta _0\left\Vert h\right\Vert _{\underline{Mor}_b(\Sigma _{t_*})}^2. \end{aligned}$$

Moreover, directly using Lemma 8.17, we have that for *a*, $\delta _r$, and $\varepsilon _{\textbf{S}}$ sufficiently small,

$$\begin{aligned} \left\lvert \left\langle \operatorname {Op}\left( \textbf{s}_{-1}\sigma (\sigma _1+\sigma _2) - \textbf{s}_{-1}\sigma _1\sigma _2\right) h,h\right\rangle _{L^2(\mathcal {D})}\right\rvert \lesssim \delta _0\left\Vert h\right\Vert _{Mor_b(\mathcal {D})}^2 + \left\Vert \mathbb {L}_{g_b}h\right\Vert _{L^2(\mathcal {D})}^2 + {\int _{\mathring{\Sigma }_{t_*}}\tilde{J}_1[h]}\Bigg \vert _{t_*=0}^{t_*={T_*}}, \end{aligned}$$

where

$$\begin{aligned} \left\lvert \int _{\mathring{\Sigma }_{t_*}}\tilde{J}_1[h]\right\rvert \lesssim \left\Vert D_{t_*}h\right\Vert _{\underline{H}^{-1}(\mathring{\Sigma })}^2 + \delta _0\left\Vert h\right\Vert _{\underline{Mor}_b(\mathring{\Sigma })}^2. \end{aligned}$$

This concludes the proof of Lemma 8.19. $\square$

The following corollary follows immediately from Lemma 8.19 and will be more useful in the context of proving a high-frequency Morawetz estimate for $\mathbb {L}_{g_b}$.

##### Corollary 8.20

Fix $\delta _0>0$ and let

$$\begin{aligned} \textbf{S}= \textbf{S}_{-1}D_{t_*}^2 + \textbf{S}_0D_{t_*} + \textbf{S}_1, \end{aligned}$$

with Schwartz kernel compactly supported on $\mathring{\Sigma }$ and where $\textbf{S}_i \in \varepsilon _{\textbf{S}}\Psi _{{\operatorname {tan}}}^i(\Sigma )$, where $\textbf{s}_{i}$ is the symbol of $\textbf{S}_{i}$, moreover, $\textbf{S}_{-1}$, $\textbf{S}_0$, and $\textbf{S}_{1}$ are Hermitian with respect to the $L^2(\mathring{\Sigma })$ Hermitian inner product. Then, there exists some Hermitian $\tilde{r} \in S^{-1}$ and sufficiently small *a*, and $\delta _r$, so that for $\varepsilon _{\textbf{S}}$ sufficiently small,

$$\begin{aligned} \left\lvert \Re \left\langle \textbf{S}h, h\right\rangle _{L^2(\mathcal {D}) } - 2\Re \left\langle \overline{\mathbb {L}}_{g_b}h, \tilde{r} h\right\rangle _{L^2(\mathcal {D})}\right\rvert < \delta _0 \left\Vert h\right\Vert _{Mor_b(\mathcal {D})}^2 + \left\Vert \mathbb {L}_{g_b}h\right\Vert _{L^2(\mathcal {D})}^2 + {\int _{\mathring{\Sigma }_{t_*}}\tilde{J}[h]}\Bigg \vert ^{t_*={T_*}}_{t_*=0}, \end{aligned}$$

where

$$\begin{aligned} \left\lvert \int _{\mathring{\Sigma }_{t_*}}\tilde{J}[h]\right\rvert \lesssim \left\Vert D_{t_*}h\right\Vert ^2_{\underline{H}^{-1}(\Sigma _{t_*})} + \delta _0 \left\Vert h\right\Vert _{\underline{Mor}_b(\Sigma _{t_*})}^2. \end{aligned}$$

and if there exists some $\sigma \in \mathbb {C}$ such that $h=e^{-\mathbbm {i}\sigma t_*}u$ where $\left\lvert \Im \sigma \right\rvert <\textbf{M}$ for some $\textbf{M}>0$, then

$$\begin{aligned} \left\lvert \int _{\mathring{\Sigma }_{t_*}}\tilde{J}[h]\right\rvert \lesssim \delta _0 \left\Vert h\right\Vert _{\underline{Mor}_b(\Sigma _{t_*})}^2 + \left\Vert \mathbb {L}_{g_b}h\right\Vert _{\underline{L}^2(\Sigma _{t_*})}^2. \end{aligned}$$

##### Proof

With Lemma 8.19 already in hand, it suffices to show that for $\textbf{S}\in \textbf{S}^{-1}D_{t_*}^2$ and for the same $\tilde{r}$ constructed in Lemma 8.19,

$$\begin{aligned} 2\left\lvert \Re \left\langle \overline{\textbf{S}}_bh,\tilde{r} h\right\rangle _{L^2(\mathcal {D})}\right\rvert \lesssim \varepsilon _{\textbf{S}}\left\Vert h\right\Vert _{Mor_b(\mathcal {D})}^2. \end{aligned}$$

To this end, we write

$$\begin{aligned} \overline{\textbf{S}}_{b} = \overline{\textbf{S}}_{1} + \overline{\textbf{S}}_{0}D_{t_*} \end{aligned}$$

where $\overline{\textbf{S}}_{i} \in \Psi _{{\operatorname {tan}}}^i(\Sigma )$. Then, observe that since $\tilde{r}$ is Hermitian, we have that

$$\begin{aligned} 2\Re \left\langle \overline{\textbf{S}}_{b}h,\tilde{r} h\right\rangle _{L^2(\mathcal {D})} = \left\langle \left( \tilde{r}\overline{\textbf{S}}_{b} + \overline{\textbf{S}}_{b}\tilde{r} \right) h, h\right\rangle _{L^2(\mathcal {D})}. \end{aligned}$$

But since $\tilde{\mathfrak {r}}\overline{\textbf{s}}_b \in S^0 + \sigma S^{-1}$, we can directly apply Lemma 8.18 to conclude. $\square$

We are now ready to control the lower-order terms that arise in the divergence theorem argument.

##### Lemma 8.21

Fix $\delta _0>0$, and let $X_b$, $q_b$ be those constructed in Lemma 8.11. Then there exists a choice of *a*, $\delta _r$, $\delta _\zeta$, and $\varepsilon _{{\Gamma }_{b_0}}$ such that for *h* as specified in Theorem 8.9, there exists a choice of $Q$ such that for $C(\delta )\in \mathbb {R}$, we have the following inequality

$$\begin{aligned}&\delta _0 \left\Vert h \right\Vert _{Mor_b(\mathcal {D})}^2 + C(\delta _0)\left\Vert \overline{\mathbb {L}}_{g_b}h\right\Vert _{L^2(\mathcal {D})}^2 \\ >{}&\frac{1}{2}\Re \left\langle \left[ \overline{\textbf{S}}_{b, s}, \mathfrak {X}_{b_0}\right] h , h \right\rangle _{L^2(\mathcal {D})} + \Re \left\langle \overline{\textbf{S}}_b[h ], \tilde{\mathfrak {q}}_{b_0} h \right\rangle _{L^2(\mathcal {D})} + \Re \left\langle \overline{\textbf{V}}_bh , (X_{b_0}+q_{b_0})h \right\rangle _{L^2(\mathcal {D})}\\&+ a\Re \mathbb {K}^{\widetilde{X}, \tilde{q}}_{(1)}[h ] + a\Re \mathbb {K}^{\widetilde{X}, \tilde{q}}_{(0)}[h ] + \left. \int _{\mathring{\Sigma }_{t_*}}\tilde{J}[{h}]\right| _{t_*= 0 }^{t_*= {T_*}}, \end{aligned}$$

where $\mathfrak {X}_{b_0}$ and $\tilde{\mathfrak {q}}_{b_0}$ are as defined in (205), and

226 $$\begin{aligned} \left\lvert \int _{\mathring{\Sigma }_{t_*}}\tilde{J}[h]\right\rvert \lesssim \left\Vert D_{t_*}h\right\Vert ^2_{\underline{H}^{-1}(\Sigma _{t_*})} + \delta _0 \left\Vert h\right\Vert _{\underline{Mor}_b(\Sigma _{t_*})}^2. \end{aligned}$$

Moreover, if there exists some $\sigma \in \mathbb {C}$ such that $h=e^{-\mathbbm {i}\sigma t_*}u$ where $\left\lvert \Im \sigma \right\rvert <\textbf{M}$ for some $\textbf{M}>0$, then

227 $$\begin{aligned} \left\lvert \int _{\mathring{\Sigma }_{t_*}}\tilde{J}[h]\right\rvert \lesssim \delta _0 \left\Vert h\right\Vert _{\underline{Mor}_b(\Sigma _{t_*})}^2 + \left\Vert \mathbb {L}_{g_b}h\right\Vert _{\underline{L}^2(\Sigma _{t_*})}^2 . \end{aligned}$$

##### Remark 37

The specific choice of the smallness constants *a*, $\delta _r$, $\delta _\zeta$, and $\varepsilon _{{\Gamma }}$ does depend here on the choice of $\delta _0>0$. In particular, these smallness constants could degenerate as $\delta _0\rightarrow 0$. However, in application, we will only need to take $\delta _0$ sufficiently small so that

$$\begin{aligned} \delta _0\left\Vert h\right\Vert _{Mor_b(\mathcal {D})}^2 \le \frac{1}{2} \left( \int _{\mathcal {D}} K^{X_{b_0}, q_{b_0}, 0}[h] + a\mathbb {K}^{\widetilde{X}, \tilde{q}}[h]\right) , \end{aligned}$$

and do not need to take $\delta _0$ arbitrarily small.

##### Proof

Let us first split the lower-order terms we wish to control into the first-order terms

228 $$\begin{aligned} \frac{1}{2}\Re \left\langle \left[ \overline{\textbf{S}}_{b,s}, \mathfrak {X}_{b_0}\right] {h}, {h}\right\rangle _{L^2(\mathcal {D})} + \Re \left\langle \overline{\textbf{S}}_b[{h}], \tilde{\mathfrak {q}}_{b_0}{h}\right\rangle _{L^2(\mathcal {D})} + \Re \left\langle \overline{\textbf{V}}_b{h}, X_{b_0}{h}\right\rangle _{L^2(\mathcal {D})} + a\Re \mathbb {K}^{\widetilde{X}, \tilde{q}}_{(1)}[{h}]; \end{aligned}$$

and the zero-order terms

229 $$\begin{aligned} \Re \left\langle \overline{\textbf{V}}_b{h}, q_{b_0}{h}\right\rangle _{L^2(\mathcal {D})} + a\Re \mathbb {K}^{\widetilde{X}, \tilde{q}}_{(0)}[{h}]. \end{aligned}$$

Let us briefly recall that in the non-trapping regimes dealt with previously, the lower-order terms are dealt with by a high-frequency argument, using the principal bulk.

In the trapped regime however, this is not possible because of the degeneration of the principal ellipticity at the trapped set. As an illustration, consider the term $\Re \left\langle \overline{\textbf{S}}_b{h}, \tilde{\mathfrak {q}}_{b_0}{h}\right\rangle _{L^2(\mathcal {D})}$. If we were to naively apply Cauchy-Schwarz, we would have

$$\begin{aligned} \left\lvert \left\langle \overline{\textbf{S}}_b{h}, \tilde{\mathfrak {q}}_{b_0}{h}\right\rangle _{L^2(\mathcal {D})}\right\rvert \lesssim \varepsilon _{{\Gamma }_{b_0}}\left( \left\Vert {h}\right\Vert _{H^1(\mathcal {D})}^2 + \left\Vert \textbf{T}{h}\right\Vert _{L^2(\mathcal {D})}^2 \right) . \end{aligned}$$

The apparent problem is that we have no way of controlling $\left\lvert \left\langle \overline{\textbf{S}}_b{h}, \tilde{\mathfrak {q}}_{b_0}{h}\right\rangle _{L^2(\mathcal {D})}\right\rvert$ by $\left\Vert {h}\right\Vert _{Mor_b(\mathcal {D})}^2$ directly using Cauchy-Schwarz because neither $\overline{\textbf{S}}_b$ nor $\tilde{\mathfrak {q}}_{b_0}$ degenerate at the trapped set in phase space, while the norm $\left\Vert {h}\right\Vert _{Mor_b(\mathcal {D})}$ was specifically engineered to vanish at the trapped set.

The way around this difficulty is to invoke Lemma 8.17, which trades the subprincipal nature of the first-order terms for a degeneracy at trapping.

**Step 1: The first-order terms not O(a)**. We first deal with the first order terms that are not *O*(*a*),

$$\begin{aligned} \frac{1}{2}\Re \left\langle \left[ \overline{\textbf{S}}_{b,s}, \mathfrak {X}_{b_0}\right] {h}, {h}\right\rangle _{L^2(\mathcal {D})} +\Re \left\langle \overline{\textbf{S}}_b[{h}], \tilde{\mathfrak {q}}_{b_0}{h}\right\rangle _{L^2(\mathcal {D})} + \Re \left\langle \overline{\textbf{V}}_b{h}, X_{b_0}{h}\right\rangle _{L^2(\mathcal {D})}. \end{aligned}$$

We show explicitly how to use the idea of exchanging degeneracy and derivatives discussed above with the terms in (228). Fix some $\delta >0$. We begin by controlling $\Re \left\langle \overline{\textbf{S}}_{b}h, \tilde{\mathfrak {q}}_{b_0}h\right\rangle _{L^2(\mathcal {D})}$ by using the smallness we gain from the conjugation by $Q$.

Observe that we can split

$$\begin{aligned} \Re \left\langle \overline{\textbf{S}}_{b}h, \tilde{\mathfrak {q}}_{b_0}h\right\rangle _{L^2(\mathcal {D})} = \Re \left\langle \overline{\textbf{S}}_{b,s}h, \tilde{\mathfrak {q}}_{b_0}h\right\rangle _{L^2(\mathcal {D})} + \Re \left\langle \overline{\textbf{S}}_{b,a}h, \tilde{\mathfrak {q}}_{b_0}h\right\rangle _{L^2(\mathcal {D})}, \end{aligned}$$

where $\overline{\textbf{S}}_{b,s}$ and $\overline{\textbf{S}}_{b,a}$ are as defined in (131). Using Lemma 8.16, the latter term reduces to

$$\begin{aligned} \Re \left\langle \overline{\textbf{S}}_{b,a}h, \tilde{\mathfrak {q}}_{b_0}h\right\rangle _{L^2(\mathcal {D})} = {\Re \left\langle \tilde{\mathfrak {q}}_{b_0}\overline{\textbf{S}}_{b,a}^{(0)}h, h\right\rangle _{\underline{L}^2(\Sigma _{t_*})}}\Bigg \vert _{t_*=0}^{t_*={T_*}}, \end{aligned}$$

where

$$\begin{aligned} \overline{\textbf{S}}_{b,a} = \overline{\textbf{S}}_{b,a}^{(0)}D_{t_*} + \overline{\textbf{S}}_{b,a}^{(1)},\qquad \overline{\textbf{S}}_{b,a}^{(i)} \in \Psi _{{\operatorname {tan}}}^i(\Sigma ). \end{aligned}$$

Moreover, from Lemma 7.18, we have that for *a*, $\delta _r$, $\delta _\zeta$, and $\varepsilon _{{\Gamma }_{b_0}}$ sufficiently small we have that $\left\Vert \overline{\textbf{S}}_{b,a}^{(0)}\right\Vert _{L^2\rightarrow L^2}\lesssim \delta$.

To control $\Re \left\langle \overline{\textbf{S}}_{b,s}[h], \tilde{\mathfrak {q}}_{b_0}h\right\rangle _{L^2(\mathcal {D})}$, we observe from its construction in Lemma 8.10 that for *a*, $\delta _r$ sufficiently small,

$$\begin{aligned} \sup _{\mathring{\Sigma }}\left\lvert \tilde{\mathfrak {q}}_{b_0}\right\rvert < \delta . \end{aligned}$$

Then using Lemma 8.17, we have that for *a*, $\delta _r$, $\delta _\zeta$, and $\varepsilon _{{\Gamma }}$ sufficiently small, we have that

$$\begin{aligned} \left\lvert \left\langle \tilde{\mathfrak {q}}_{b_0}\overline{\textbf{S}}_{b,s}[h], h\right\rangle _{L^2(\mathcal {D})}\right\rvert \le \delta \left\Vert h\right\Vert _{Mor_b(\mathcal {D})}^2 + \left\Vert \mathbb {L}_{g_b}h\right\Vert _{L^2(\mathcal {D})}^2 + {Err_{\mathring{\Sigma }}[h]}\Bigg \vert _{t_*=0}^{t_*={T_*}}, \end{aligned}$$

where

$$\begin{aligned} \left\lvert Err_{\mathring{\Sigma }}[h]\right\rvert \lesssim \delta \left\Vert h\right\Vert _{\underline{Mor}_b(\mathring{\Sigma })}^2 + \left\Vert D_{t_*}h\right\Vert _{\underline{H}^{-1}(\mathring{\Sigma })}^2 \end{aligned}$$

for $\delta _r$, $\varepsilon _{{\Gamma }}$, and *a* sufficiently small. We now consider the term

$$\begin{aligned} \Re \left\langle \left[ \overline{\textbf{S}}_{b,s}, \mathfrak {X}_{b_0}\right] h, h\right\rangle _{L^2(\mathcal {D})}. \end{aligned}$$

Recall from its definition in (205) that

$$\begin{aligned} \mathfrak {X}_{b_0} = X_{b_0} + \frac{1}{2}\nabla _{g_b}\cdot X_{b_0}. \end{aligned}$$

Since $\nabla _{g_b}\cdot X_{b_0}$ is just a smooth function in *r* on $\mathring{\Sigma }$, we have that

$$\begin{aligned} {}\left[ \overline{\textbf{S}}_{b,s}, \nabla _{g_b}\cdot X_{b_0}\right] \in \Psi _{{\operatorname {tan}}}^0(\Sigma ). \end{aligned}$$

As a result, using Lemma 8.14, we have that for sufficiently small $\delta _r$,

$$\begin{aligned} \left\lvert \left\langle \left[ \overline{\textbf{S}}_{b,s}, \nabla _{g_b}\cdot X_{b_0}\right] h, h\right\rangle _{L^2(\mathcal {D})}\right\rvert < \delta \left\Vert h\right\Vert _{Mor_b(\mathcal {D})}^2. \end{aligned}$$

Furthermore, using the explicit form of $X_{b_0}$ in (179), we can write that

$$\begin{aligned} {}\left[ \overline{\textbf{S}}_{b,s}, X_{b_0}\right]&= \left[ \overline{\textbf{S}}_{b,s}, f_{b_0}(r)\right] \partial _r + f_{b_0}(r)\left[ \overline{\textbf{S}}_{b,s}, \partial _r\right] . \end{aligned}$$

Since $f_{b_0}(r)$ is a smooth function we can use Lemma 8.15 to see that for $\delta _r$ sufficiently small,

230 $$\begin{aligned} \left\lvert \left\langle \left[ \overline{\textbf{S}}_{b,s}, f_{b_0}(r)\right] \partial _rh, h\right\rangle _{L^2(\mathcal {D})}\right\rvert < \delta \left\Vert h\right\Vert _{Mor_b(\mathcal {D})}^2 + \left\Vert \mathbb {L}_{g_b}h\right\Vert _{L^2(\mathcal {D})}^2 + {\int _{\mathring{\Sigma }_{t_*}}\widetilde{J}_0[h]}\Bigg \vert ^{t_*={T_*}}_{t_*=0}, \end{aligned}$$

where

$$\begin{aligned} \left\lvert \int _{\mathring{\Sigma }_{t_*}}\widetilde{J}_0[h]\right\rvert \lesssim \left\Vert D_{t_*}h\right\Vert ^2_{\underline{H}^{-1}(\Sigma _{t_*})} + \delta _0\left\Vert h\right\Vert ^2_{Mor_b(\Sigma _{t_*})}. \end{aligned}$$

The last term, $\left\langle \overline{\textbf{V}}_b{h}, X_{b_0}{h}\right\rangle _{L^2(\mathcal {D})}$, can be handled directly using Cauchy-Schwarz from Lemma 8.14.

**Step 2: First-order terms that are O(a)**. We handle the remaining first order term, $a\mathbb {K}^{\widetilde{X}, \tilde{q}}_{(1)}[{h}]$ in a similar fashion. The main difference to the previous case is that instead of relying on the smallness we gain by considering the symbol decomposition of $\overline{\textbf{S}}_b$, we rely on the inherent smallness present in *a*.

First, we recall that

$$\begin{aligned} \mathbb {K}^{\widetilde{X}, \tilde{q}}_{(1)}[{h}] = \frac{1}{2}\left\langle \left[ \overline{\textbf{S}}_{b,s}, \widetilde{X}\right] {h},{h}\right\rangle _{L^2(\mathcal {D})} + \left\langle \overline{\textbf{S}}_b {h}, \tilde{q} {h}\right\rangle _{L^2(\mathcal {D})} + \left\langle \overline{\textbf{V}}_b {h}, \widetilde{X} {h}\right\rangle _{L^2(\mathcal {D})}. \end{aligned}$$

Then observe that directly using Lemma 8.16 and Lemma 8.14 we have that for $\delta _r$ sufficiently small,

$$\begin{aligned} \left\lvert \left\langle \overline{\textbf{S}}_{b,a}h, \tilde{q}h \right\rangle _{L^2(\mathcal {D})}\right\rvert + \left\lvert \left\langle \overline{\textbf{V}}_{b,a}h, \tilde{q}h \right\rangle _{L^2(\mathcal {D})}\right\rvert < \delta \left\Vert h\right\Vert _{\underline{Mor}_b(\mathring{\Sigma })}^2. \end{aligned}$$

We now move on to handling

$$\begin{aligned} \mathbb {K}^{\widetilde{X}, \tilde{q}}_{(1,s)}[{h}] {:=} \frac{1}{2}\left\langle \left[ \overline{\textbf{S}}_{b,s}, \widetilde{X}\right] {h},{h}\right\rangle _{L^2(\mathcal {D})} + \left\langle \overline{\textbf{S}}_{b,s} {h}, \tilde{q} {h}\right\rangle _{L^2(\mathcal {D})} + \left\langle \overline{\textbf{V}}_{b,a} {h}, \widetilde{X} {h}\right\rangle _{L^2(\mathcal {D})}. \end{aligned}$$

Observe that

$$\begin{aligned} \mathbb {K}^{\widetilde{X}, \tilde{q}}_{(1,s)}[{h}] = \left\langle \textbf{k}^{\widetilde{X}, \tilde{q}}_{(1)}{h}, {h}\right\rangle _{L^2(\mathcal {D})} +\left. \left\langle \overline{\textbf{V}}_{b,a}{h}, \widetilde{X}_0{h}\right\rangle _{L^2(\mathring{\Sigma }_{t_*})}\right| _{t_*=0}^{t_*={T_*}} +\left. \left\langle \overline{\textbf{S}}_{b,s}{h}, \tilde{q}_{-1}{h}\right\rangle _{L^2(\mathring{\Sigma }_{t_*})}\right| _{t_*=0}^{t_*={T_*}}, \end{aligned}$$

where

$$\begin{aligned} \textbf{k}^{\widetilde{X}, \tilde{q}}_{(1)} = \left[ \overline{\textbf{S}}_{b,s}, \widetilde{X}\right] + \tilde{q}\overline{\textbf{S}}_b -\widetilde{X}\overline{\textbf{V}}_b \in \Psi _{{\operatorname {tan}}}^{-1}(\Sigma )D_{t_*}^2 + \Psi _{{\operatorname {tan}}}^0(\Sigma )D_{t_*} + \Psi _{{\operatorname {tan}}}^1(\Sigma ). \end{aligned}$$

It is clear that the accumulated boundary terms satisfy

$$\begin{aligned} \left\lvert \left\langle \overline{\textbf{V}}_b{h}, \widetilde{X}_0{h}\right\rangle _{L^2(\mathring{\Sigma }_{t_*})}\right\rvert +\left\lvert \left\langle \overline{\textbf{S}}_{b}{h}, \tilde{q}_{-1}{h}\right\rangle _{L^2(\mathring{\Sigma }_{t_*})}\right\rvert \lesssim \left\Vert h\right\Vert _{\underline{L}^2(\mathring{\Sigma }_{t_*})}^2 + \left\Vert \textbf{T}h\right\Vert _{\underline{H}^{-1}(\mathring{\Sigma }_{t_*})}^2. \end{aligned}$$

The bound in (226) then follows from Lemma 8.14. Moreover, using Lemma 8.18, for *a* sufficiently small, these auxiliary boundary terms can be controlled by $\delta \left\Vert {h}\right\Vert _{\underline{Mor}_b(\mathring{\Sigma }_{t_*})}^2 + \left\Vert \mathbb {L}_{g_b}h\right\Vert ^2_{\underline{L}^2(\mathring{\Sigma })}$. We can now use Lemma 8.14 to directly see that for *a* and $\delta _r$ sufficiently small, we have that

$$\begin{aligned} \left\lvert \left\langle a\textbf{k}^{\widetilde{X}, \tilde{q}}_{(1)}{h}, {h}\right\rangle _{L^2(\mathcal {D})}\right\rvert < \delta \left\Vert h\right\Vert _{Mor_b(\mathcal {D})}^2. \end{aligned}$$

**Step 3: Zero-order terms.** The zero-order terms can be directly controlled by Lemma 8.14.

We conclude the proof of Lemma 8.21 by picking a suitable $\tilde{J}[{h}]$ to cancel out all the auxiliary boundary terms we picked up in the process of integrating by parts. $\square$

We have now generated a principally positive bulk term up to some error consisting entirely of boundary integrals.

**Controlling the boundary terms**

The last step in proving a resolvent estimate for $\left\lvert \Im \sigma \right\rvert \le {\boldsymbol{\alpha }}$ will be to show that we can absorb the boundary terms into the positive bulk term as in the proof Proposition 8.7.

##### Lemma 8.22

Fix some $0<\delta _0\ll 1$. Then, there exists a choice of *a*, $\delta _r$, $\delta _\zeta$, $\varepsilon _{{\Gamma }_{b_0}}$, and ${\boldsymbol{\alpha }}$ sufficiently small such that for ${h}=e^{-\mathbbm {i}\sigma t}u$ where $\left\lvert \Im \sigma \right\rvert < {\boldsymbol{\alpha }}$,

$$\begin{aligned} e^{2\Im \sigma t}\left\lvert \Im \sigma \right\rvert \left\lvert \int _{\mathring{\Sigma }}J^{X_{b_0}, q_{b_0},0}[{h}]\cdot n_{\mathring{\Sigma }} + a\Re \mathbb {J}^{\widetilde{X}, \tilde{q}}[{h}]\right\rvert < \delta _0 \left\Vert u\right\Vert _{\underline{Mor}_b(\mathring{\Sigma })}^2. \end{aligned}$$

##### Proof

The main idea in this proof will be to recycle the methods we have already used to handle the bulk terms to analyze the boundary terms. Let us first fix some auxiliary $\delta >0$.

**Step 1: Principal boundary term.** We begin by applying the methods used to handle the principal bulk term to handle the principal boundary term, showing that for ${h} = e^{-\mathbbm {i}\sigma t_*}u$,

231 $$\begin{aligned} e^{-2\Im \sigma t_*}\left\lvert \Re \left\langle n_{\mathring{\Sigma }}{h}, X_{b_0}{h}\right\rangle _{L^2(\mathring{\Sigma })} + a\Re \mathbb {J}^{\widetilde{X}, \tilde{q}}_{(2)}[h]\right\rvert < \delta \left\Vert u\right\Vert _{\underline{Mor}_b(\mathring{\Sigma })}^2. \end{aligned}$$

Observe that on $\mathring{\Sigma }$, $n_{\mathring{\Sigma }}= \frac{1}{\sqrt{A}}dt_*$, so for ${h} = e^{-\mathbbm {i}\sigma t_*}u$,

$$\begin{aligned} e^{-2\Im \sigma t_*}\left( \Re \left\langle n_{\mathring{\Sigma }}{h}, X_{b_0}{h}\right\rangle _{L^2(\mathring{\Sigma })} + a\Re \left\langle n_{\mathring{\Sigma }}{h}, \widetilde{X}{h}\right\rangle _{L^2(\mathring{\Sigma })} \right) = -\Re \left\langle \sigma u, X_b u\right\rangle _{L^2(\mathring{\Sigma })}. \end{aligned}$$

As a result, choosing $\left\lvert \Im \sigma \right\rvert$ sufficiently small,

$$\begin{aligned} \left\lvert \Im \sigma \right\rvert \left\lvert \left\langle {h}, n_{\mathring{\Sigma }}X_b{h}\right\rangle _{L^2(\mathring{\Sigma })}\right\rvert < \delta \left\Vert {u}\right\Vert _{\underline{Mor}_b(\mathring{\Sigma })}^2. \end{aligned}$$

We remark that since we use the smallness $\Im \sigma$ to conclude, this step necessarily restricts the size of the spectral gap.

**Step 2: Lower-order boundary terms.** We now handle the lower-order boundary terms, showing that for ${h}=e^{-\mathbbm {i}\sigma t_*}u$,

232 $$\begin{aligned} \delta \left\Vert h\right\Vert _{\underline{Mor}_b(\mathring{\Sigma })}^2>&\left\lvert \Im \sigma \right\rvert \left( \left\lvert \left\langle q_{b_0}{h}, n_{\mathring{\Sigma }}{h}\right\rangle _{L^2(\mathring{\Sigma })}\right\rvert + \left\lvert \left\langle g_b(\textbf{T}, n_{\mathring{\Sigma }})\overline{\textbf{S}}_{0,a}h, X_{b_0}h\right\rangle _{L^2(\mathring{\Sigma })}\right\rvert + a\left\lvert \mathbb {J}^{\widetilde{X}, \tilde{q}}_{(1)}[{h}]\right\rvert \right) . \end{aligned}$$

To handle these lower-order boundary terms, we appeal to Lemma 8.18, mirroring the approach taken in proving Lemma 8.21. Using Lemma 8.18, we see that for sufficiently small^21^$\delta _r$, $\Im \sigma$,

233 $$\begin{aligned} \Im \sigma \left( \left\lvert \left\langle q_{b_0}{u}, \sigma {u}\right\rangle _{L^2(\mathring{\Sigma })}\right\rvert + \left\lvert \left\langle g_b(\textbf{T}, n_{\mathring{\Sigma }})\overline{\textbf{S}}_{0,a}u, X_{b_0}u\right\rangle _{L^2(\mathring{\Sigma })}\right\rvert \right) < \delta \left\Vert u\right\Vert _{\underline{Mor}_b(\mathring{\Sigma })}^2. \end{aligned}$$

To handle $a\left\lvert \mathbb {J}^{\widetilde{X}, \tilde{q}}_{(1)}[{h}]\right\rvert$, we can again use Lemma 8.18 to see that for sufficiently small^22^*a*, $\delta _r$,

234 $$\begin{aligned} \left\lvert a e^{-2\Im \sigma t_*}\Re \mathbb {J}^{\widetilde{X}, \tilde{q}}_{(1)}[h]\right\rvert <\delta \left\Vert u\right\Vert _{\underline{Mor}_b(\mathring{\Sigma })}^2. \end{aligned}$$

Combining (233) and (234) concludes the proof of Lemma 8.22. $\square$

**Resolvent estimate for** $\Im \sigma >\frac{{\boldsymbol{\alpha }}}{2}$ Let us first begin then by showing that a naive Gronwall-type energy estimate allows us to reduce the problem to the region where $\left\lvert \Im \sigma \right\rvert \le {\boldsymbol{\alpha }}$.

##### Lemma 8.23

Let *u* be as specified in the assumptions of Theorem 8.9. Then, for any $\delta >0$, there exists some constant $C>0$ such that for $\Im \sigma > \delta$, $\left\lvert \sigma \right\rvert >C$,

235 $$\begin{aligned} \left\Vert u\right\Vert _{\underline{L}^2(\mathring{\Sigma })} \lesssim \left\Vert \widehat{\overline{\mathbb {L}}}_{g_b}(\sigma )u\right\Vert _{\underline{L}^2(\mathring{\Sigma })}. \end{aligned}$$

##### Proof

The proof of this lemma will proceed in the same way as Corollary 6.4, we will take advantage of the smallness of $\overline{\textbf{s}}_b$ by microlocalizing to a neighborhood of ${\Gamma }_b$.

Recall that $\textbf{T}$ is uniformly timelike and Killing on $\mathring{\Sigma }$. Thus, on $\mathring{\Sigma }$, $E(t_*)[{h}]$ is coercive. Moreover, using the divergence theorem we have that

$$\begin{aligned} E({T_*})[{h}] \!-\! E(0)[{h}] \!=\!{}&\!-\! \Re \left\langle \overline{\mathbb {L}}_{g_b} {h}, \textbf{T}{h}\right\rangle _{L^2(\mathcal {D})} \!+\! \Re \left\langle \overline{\textbf{S}}_b[{h}],\textbf{T}{h}\right\rangle _{L^2(\mathcal {D})} \!+\! \Re \left\langle \overline{\textbf{V}}_b{h}, \textbf{T}{h}\right\rangle _{L^2(\mathcal {D})}. \end{aligned}$$

Taking a $\partial _{t_*}$ derivative, we have that

$$\begin{aligned} \partial _{t_*}E(t_*)[{h}] ={}&- \Re \left\langle \overline{\mathbb {L}}_{g_b} {h}, \textbf{T}{h}\right\rangle _{\underline{L}^2(\mathring{\Sigma })} + \Re \left\langle \overline{\textbf{S}}_b[{h}],\textbf{T}{h}\right\rangle _{\underline{L}^2(\mathring{\Sigma })} + \Re \left\langle \overline{\textbf{V}}_b{h}, \textbf{T}{h}\right\rangle _{\underline{L}^2(\mathring{\Sigma })}. \end{aligned}$$

Using Lemma 8.16, we have that

$$\begin{aligned} \partial _{t_*}E(t_*)[{h}] ={}&- \Re \left\langle \overline{\mathbb {L}}_{g_b} {h}, \textbf{T}{h}\right\rangle _{\underline{L}^2(\mathring{\Sigma })} + \Re \left\langle \overline{\textbf{S}}_{b,a}[{h}],\textbf{T}{h}\right\rangle _{\underline{L}^2(\mathring{\Sigma })} + \Re \left\langle \overline{\textbf{V}}_b{h}, \textbf{T}{h}\right\rangle _{\underline{L}^2(\mathring{\Sigma })}. \end{aligned}$$

Using Lemma 7.18, we have that for any fixed $\epsilon >0$, there exists a choice of *a*, $\varepsilon _{{\Gamma }}$, $\delta _r$, and $\delta _\zeta$ sufficiently small such that $\left\lvert \overline{\textbf{s}}_b\right\rvert \le \delta _0\left\lvert \zeta \right\rvert$. As a result, large,

$$\begin{aligned} \left\lvert \left\langle \overline{\textbf{S}}_{b,a}{h}, \textbf{T}{h}\right\rangle _{\underline{L}^2(\mathring{\Sigma })}\right\rvert&< \epsilon \left\Vert h\right\Vert _{\overline{H}^{1}(\mathring{\Sigma })}^2,\\ \left\lvert \left\langle \overline{\textbf{V}}_b{h}, \textbf{T}{h}\right\rangle _{\underline{L}^2(\mathring{\Sigma })}\right\rvert&< \epsilon \left\Vert h\right\Vert _{\underline{L}^2(\mathring{\Sigma })}^2 + C(\epsilon )\left\Vert h\right\Vert _{\underline{L}^2(\mathring{\Sigma })}^2. \end{aligned}$$

Recall that $\mathring{\Sigma }$ does not intersect the ergoregion of $g_b$ and that therefore $E(t_*)[h]$ is strictly positive and coercive for *h* such that $h(t_*, \cdot )$ is supported on $\mathring{\Sigma }$ for all $t_*$. As a result, for ${h}=e^{-\mathbbm {i}\sigma t_*}u$,

$$\begin{aligned} \partial _{t_*}E(t_*)[{h}] \gtrsim \Im \sigma \left\Vert h\right\Vert _{\overline{H}^{1}(\mathring{\Sigma })}^2. \end{aligned}$$

Since we are only considering $\Im \sigma >\delta$, the right-hand side is positive, and now we can now multiply through by $e^{-2\Im \sigma t_*}$ to remove any $t_*$ dependency to see that

$$\begin{aligned} \left\Vert u\right\Vert _{\underline{H}^{1}(\mathring{\Sigma })}^2 + \left\Vert \sigma u\right\Vert _{\underline{L}^2(\mathring{\Sigma })}^2 \lesssim \left\Vert \widehat{\overline{\mathbb {L}}}_{g_b}(\sigma )u\right\Vert _{\underline{L}^2(\mathring{\Sigma })}^2 +\left\Vert u\right\Vert _{\underline{L}^2(\mathring{\Sigma })}^2. \end{aligned}$$

We conclude by observing as before that the lower-order term on the right-hand side can be absorbed into the left-hand side for $\left\lvert \sigma \right\rvert$ sufficiently large. $\square$

**Reduction to conjugated operator**

All of the estimates thus far in the section have been proven by multiplying against $\overline{\mathbb {L}}_{g_b}$ instead of $\mathbb {L}_{g_b}$. In this section, we show that this is in fact sufficient to prove Theorem 8.9.

##### Lemma 8.24

Let $\mathcal {D}$ and $\mathring{\Sigma }$ be as defined in (176), and *u* be a sufficiently smooth function supported in $\mathring{\Sigma }$ such that $\hat{u}(\xi , \eta )$ is supported on the region $\frac{\left\lvert \xi \right\rvert ^2}{\left\lvert \eta \right\rvert ^2} \le 2\delta _\zeta$. Then if there exists ${\boldsymbol{\alpha }}>0$, $C_0>0$, such that

236 $$\begin{aligned} \left\Vert u\right\Vert _{Mor_b(\mathring{\Sigma })} \lesssim \left\Vert \widehat{\overline{\mathbb {L}}}_{g_b}(\sigma )u\right\Vert _{\underline{L}^2_\sigma (\mathring{\Sigma })},\qquad {\text {if }}\Im \sigma > -{\boldsymbol{\alpha }},{\text { and }} \left\lvert \sigma \right\rvert \ge C_0; {\text { or }} \Im \sigma = -{\boldsymbol{\alpha }}, \end{aligned}$$

Then Theorem 8.9 holds.

##### Proof

Observe that there exists some $$C_{ell}>0$$ such that if $$\left\lvert \sigma \right\rvert > C_{ell}(\left\lvert \xi \right\rvert + \left\lvert \eta \right\rvert )$$, then $$p_b > 0 $$ is elliptic. Then we first show that it is sufficient to prove Theorem 8.9 for $$\hat{u}(\xi , \eta )$$ supported on^23^$$\left\lvert \xi , \eta \right\rvert > \frac{1}{C_{ell}}\left\lvert \sigma \right\rvert $$.

To this end, let $\hat{u}(\xi , \eta )$ be supported on $\left\lvert \sigma \right\rvert > C_{ell}(\xi + \left\lvert \eta \right\rvert )$. Then $p_b$ is elliptic, and we have directly via (24) that

$$\begin{aligned} \!-\!\left\langle \mathbb {L}_{g_b}h, h\right\rangle _{L^2(\mathring{\Sigma })} \!=\! \int _{\mathring{\Sigma }}K^{0, 1, 0}[h]\,\sqrt{A} \!-\! \left\langle \textbf{S}_bh, h\right\rangle _{L^2(\mathring{\Sigma })} \!-\! \left\langle \textbf{V}_bh, h\right\rangle _{L^2(\mathring{\Sigma })} \!+\! \partial _{t_*}\int _{\mathring{\Sigma }} J^{0, 1, 0}[h] , \end{aligned}$$

where

$$\begin{aligned} K^{0,1,0}[h] \gtrsim \left\lvert \nabla h\right\rvert ^2 - C\left\lvert h\right\rvert ^2, \qquad \left\lvert J^{0,1,0}[h]\right\rvert < \epsilon \left\lvert \nabla h\right\rvert ^2 + C(\epsilon )\left\lvert h\right\rvert ^2. \end{aligned}$$

Directly from Cauchy-Schwarz, we have then that

$$\begin{aligned} \int _{\mathring{\Sigma }}K^{0, 1, 0}[h]\,\sqrt{A} - \left\langle \textbf{S}_bh, h\right\rangle _{L^2(\mathring{\Sigma })} - \left\langle \textbf{V}_bh, h\right\rangle _{L^2(\mathring{\Sigma })} \gtrsim \left\Vert h\right\Vert _{\overline{H}^{1}(\mathring{\Sigma })}^2 - \left\Vert h\right\Vert _{\underline{L}^2(\mathring{\Sigma })}^2. \end{aligned}$$

Then, considering $h=e^{-\mathbbm {i}\sigma t_*}u(x)$, and multiplying both sides by $e^{-2\Im \sigma t_*}$ and using the fact that $\left\lvert \Im \sigma \right\rvert \le \textbf{M}$ as usual, we have that

$$\begin{aligned} \left\Vert u\right\Vert _{\underline{H}^{1}(\mathring{\Sigma })}^2 + \left\Vert \sigma u\right\Vert _{\underline{L}^2(\mathring{\Sigma })}^2 \lesssim \left\Vert \widehat{\mathbb {L}}_{g_b}(\sigma )u\right\Vert _{\underline{L}^2(\mathring{\Sigma })}^2 + \left\Vert u\right\Vert _{\underline{L}^2(\mathring{\Sigma })}^2. \end{aligned}$$

The desired resolvent estimate in (189) follows directly by taking $\left\lvert \sigma \right\rvert$ sufficiently large and absorbing the lower-order term on the right-hand side into the left-hand side.

We now show that return to proving the lemma itself. Since it suffices to consider $\hat{u}(\xi , \eta )$ supported on $\left\lvert \xi , \eta \right\rvert > \frac{1}{C_{ell}}\left\lvert \sigma \right\rvert$, and we are can choose $\left\lvert \sigma \right\rvert$ as large as we like, we can in particular choose $\left\lvert \sigma \right\rvert$ large enough so that $\hat{u}(\xi , \eta )$ is supported on the region where

$$\begin{aligned} \left\Vert Qu\right\Vert _{\underline{L}^2(\mathring{\Sigma })} \lesssim \left\Vert u\right\Vert _{\underline{L}^2(\mathring{\Sigma })}, \qquad \left\Vert Q^- u\right\Vert _{\underline{L}^2(\mathring{\Sigma })} \lesssim \left\Vert u\right\Vert _{\underline{L}^2(\mathring{\Sigma })}. \end{aligned}$$

Then, we have that

$$\begin{aligned} \left\Vert u\right\Vert _{\underline{Mor}_b(\mathring{\Sigma })}&\lesssim \left\Vert \widehat{\overline{\mathbb {L}}}_{g_b}(\sigma )u\right\Vert _{\underline{L}^2(\mathring{\Sigma })}\nonumber \\&\lesssim \left\Vert \widehat{\mathbb {L}}_{g_b}(\sigma ) u\right\Vert _{\underline{L}^2(\mathring{\Sigma })} + \left\Vert \mathfrak {R}u\right\Vert _{\underline{L}^2(\mathring{\Sigma })}\nonumber \\&\lesssim \left\Vert \widehat{\mathbb {L}}_{g_b}(\sigma )u\right\Vert _{\underline{L}^2(\mathring{\Sigma })} + \left\Vert \left[ \widehat{\mathbb {L}}_{g_b}(\sigma ), \mathfrak {r}\right] u\right\Vert _{\underline{L}^2(\mathring{\Sigma })}, \end{aligned}$$

where $\mathfrak {r} = [\widehat{\mathbb {L}}_{g_b}(\sigma ), Q]Q^- + [\widehat{\mathbb {L}}_{g_b}(\sigma ), 1- QQ^-]\in \sigma \operatorname {Op}S^{-1}$. Then, we can directly use Lemma 8.15 and choose $\delta _0$ sufficiently small so that

$$\begin{aligned} \left\Vert u\right\Vert _{\underline{Mor}_b(\mathring{\Sigma })} \lesssim \left\Vert \widehat{\mathbb {L}}_{g_b}(\sigma )u\right\Vert _{\underline{L}^2(\mathring{\Sigma })}, \end{aligned}$$

as desired. $$\square $$

**Proving Theorem** 8.9

We are now ready to prove Theorem 8.9. We first show how to combine the above results to conclude the $k=1$ case of Theorem 8.9.

***Proof of Theorem*** 8.9***for*** k=1 Using Lemma 8.11 and Corollary 8.13 we can write

$$\begin{aligned} \int _{\mathcal {D}}K^{X_{b_0},q_{b_0},0}[{h}] + a\Re \mathbb {K}^{\widetilde{X}, \tilde{q}}_{(2)}[{h}] - \Re \left\langle \overline{\textbf{S}}_b[{h}], X_{b_0}{h}\right\rangle _{L^2(\mathcal {D})} \ge {}&\frac{1}{2}\int _{\mathcal {D}}\sum _{j=1}^7\left\lvert \mathfrak {A}_j{h}\right\rvert ^2 + \int _{\mathcal {D}}\nabla ^\alpha \partial _\alpha q_{b_0}\left\lvert {h}\right\rvert ^2. \end{aligned}$$

Choosing *a*, and $\delta$, sufficiently small we can use Lemma 8.21 to control the lower order bulk terms as well and see that

$$\begin{aligned} \int _{\mathcal {D}}\sum _{j=1}^7\left\lvert \mathfrak {A}_j{h}\right\rvert ^2 + \left\Vert {h}\right\Vert _{L^2(\mathcal {D})}^2 \lesssim {}&\int _{\mathcal {D}}K^{X_{b_0},q_{b_0},0}[{h}] - \Re \left\langle \overline{\textbf{S}}_b[{h}], (X_{b_0}+q_{b_0}){h}\right\rangle _{L^2(\mathcal {D})}\\&- \Re \left\langle \overline{\textbf{V}}_b{h},(X_{b_0}+q_{b_0}){h} \right\rangle _{L^2(\mathcal {D})} + a\Re \mathbb {K}^{\widetilde{X}, \tilde{q}}[{h}] + \left. \tilde{J}(t_*)[{h}]\right| _{t_*=0}^{t_*= {T_*}}. \end{aligned}$$

Defining

$$\begin{aligned} \mathring{\mathfrak {E}}(t_*)[{h}] = \int _{\Sigma _{t_*}}J^{X_{b_0}, q_{b_0},0}[{h}]\cdot n_{\mathring{\Sigma }} + a \Re \mathbb {J}^{\widetilde{X}, \tilde{q}}(t_*)[{h}] + \tilde{J}(t_*)[{h}], \end{aligned}$$

it is clear that

237 $$\begin{aligned} \left. \mathring{\mathfrak {E}}(t_*)[{h}]\right| _{t_*= 0}^{t_*= {T_*}} + \left\Vert {h}\right\Vert _{Mor_b(\mathcal {D})}^2 \lesssim \left\Vert \mathbb {L}_{g_b}{h}\right\Vert _{L^2(\mathcal {D})}^2. \end{aligned}$$

We now prove the resolvent estimate in (189) for $k=1$. As before, we consider $h= e^{-\mathbbm {i}\sigma t_*}u(x)$, where *u* satisfies the assumptions made in the statement of the theorem.

Differentiating both sides of (237) by $\partial _{t_*}$ and multiplying by $e^{-2\Im \sigma t_*}$ to remove any $t_*$-dependency, we see that

$$\begin{aligned} \begin{aligned} \left\Vert u\right\Vert _{Mor_b(\mathring{\Sigma })}^2 \lesssim {}&\left\Vert \widehat{\mathbb {L}}_{g_b}(\sigma )u\right\Vert _{L^2(\mathring{\Sigma })}^2\\&\!+\! e^{\!-\!2\Im \sigma t_*}\left\lvert \partial _{t_*}\left( \int _{\Sigma _{t_*}}J^{X_{b_0}, q_{b_0},0}[{h}]\cdot n_{\mathring{\Sigma }} \!+\! a \Re \mathbb {J}^{\widetilde{X}, \tilde{q}}(t_*)[{h}] \!+\! \tilde{J}(t_*)[{h}] \right) \right\rvert . \end{aligned} \end{aligned}$$

We now show that for ${\boldsymbol{\alpha }}$ sufficiently small, and $\left\lvert \sigma \right\rvert$ sufficiently large, the boundary terms on the right-hand side can be absorbed into the bulk norm on the left-hand side. Since ${h}=e^{-\mathbbm {i}\sigma t_*}u$, we can now apply Lemma 8.22, and the bound on $\tilde{J}(t_*)[{h}]$ from Lemma 8.21 to choose $\varepsilon _{{\Gamma }_{b_0}}$, *a*, ${\boldsymbol{\alpha }}$ sufficiently small so that for $\left\lvert \Im \sigma \right\rvert \le {\boldsymbol{\alpha }}$,

$$\begin{aligned} \begin{aligned} e^{2\Im \sigma t_*}\left\lvert \partial _{t_*}\left( \int _{\Sigma _{t_*}}J^{X_{b_0}, q_{b_0},0}[{h}]\cdot n_{\mathring{\Sigma }} +1 a \Re \mathbb {J}^{\widetilde{X}, \tilde{q}}(t_*)[{h}] + \tilde{J}(t_*)[{h}] \right) \right\rvert \lesssim \delta \left\Vert u\right\Vert _{\underline{Mor}_b(\mathring{\Sigma })}^2. \end{aligned} \end{aligned}$$

Choosing $\delta$ sufficiently small, we conclude via Lemma 8.11 and Proposition 7.11 that for $\left\lvert \sigma \right\rvert$ sufficiently large,

$$\begin{aligned} \left\Vert u\right\Vert _{\underline{Mor}_b(\mathring{\Sigma })} \lesssim \left\Vert \widehat{\mathbb {L}}_{g_b}(\sigma )u\right\Vert _{\underline{L}^2(\mathring{\Sigma })}, \end{aligned}$$

as desired. $$\square $$

We now show how to commute derivatives through the relevant estimates to obtain higher order resolvent estimates. The main idea here is that $\textbf{T}$ is both Killing and time-like on $\mathring{\Sigma }$. As a result, we can use a combination of commuting with $\textbf{T}$ and elliptic estimates to prove via induction higher-order estimates.

***Proof of Theorem*** 8.9***for*** k>1 We begin with the observation that since $\textbf{T}$ is Killing, it commutes with $\mathbb {L}_{g_b}$. As a result,

$$\begin{aligned} \left\Vert \sigma u\right\Vert _{\underline{Mor}_b(\mathring{\Sigma })} \lesssim \left\Vert \sigma \widehat{\mathbb {L}}_{g_b}(\sigma )u\right\Vert _{\underline{L}^2(\mathring{\Sigma })}. \end{aligned}$$

To control the rest of the derivatives, we recall that we can write

$$\begin{aligned} \mathbb {L}_{g_b} = A^{-1} D_{t_*}^2 + P_1D_{t_*} + P_2, \end{aligned}$$

where $P_i\in \Psi _{{\operatorname {tan}}}^i(\mathring{\Sigma })$. We rewrite the main equation as

$$\begin{aligned} P_2{h} = \mathbb {L}_{g_b}{h} - A^{-1} D_{t_*}^2 {h} - P_1D_{t_*}{h}. \end{aligned}$$

We conclude by recalling that $P_2$ is elliptic on $\mathring{\Sigma }$ and using standard elliptic estimates. $\square$

### Proof of Theorem 8.1

At this point, we have proven resolvent estimates for *h* supported on the redshift region, the non-trapping region, and the trapping region. In this section, the goal will be to glue these estimates together. To this end, recall the relation between the constants

$$\begin{aligned} r_{\mathcal {H}^+}< \breve{r}_{-}< r_{\bullet ,\mathcal {H}^+}<r_{0}< \mathring{r}_{-}< \breve{r}_{+}<3M<\breve{R}_{-}<\mathring{R}_{ +}< R_{0}< R_{\bullet ,\mathcal {H}^+}< \breve{R}_{+} < r_{\overline{\mathcal {H}}^+}. \end{aligned}$$

In practice, it will be useful to consider

$$\begin{aligned}&\breve{r}_{-} = r_{\mathcal {H}^+}+\delta _{\mathcal {H}}, \quad r_{\bullet , \mathcal {H}^+} = r_{\mathcal {H}^+} + 2\delta _{\mathcal {H}} ,\quad \mathring{r}_{ -} = 3M- 4\delta _r, \\&\quad \breve{r}_{ +} = 3M - 2\delta _r, \breve{R}_{-} = 3M + 2\delta _r,\quad \mathring{R}_{ +} = 3M + 4\delta _r,\\&\quad R_{\bullet , \overline{\mathcal {H}}^+} = r_{\overline{\mathcal {H}}^+} - 2\delta _{\mathcal {H}} ,\quad \breve{R}_+ = r_{\overline{\mathcal {H}}^+} - \delta _{\mathcal {H}}. \end{aligned}$$

One would wish to directly apply Theorems 8.2, 8.3, 8.5, and 8.9, to prove Theorem 8.1. However, we cannot actually do this, as from directly applying Theorems 8.2, 8.3, 8.5, and 8.9, we only have that for $k>{k_0}$

$$\begin{aligned} \begin{aligned} \left\Vert u\right\Vert _{\underline{LE}^{k}(\Sigma )} \lesssim {}&\left\Vert \widehat{\mathbb {L}}_{g_b}(\sigma )(\dot{\chi }u)\right\Vert _{\underline{H}^{k-1}_\sigma (\Sigma )} + \left\Vert \widehat{\mathbb {L}}_{g_b}(\sigma )(\breve{\chi }u)\right\Vert _{\underline{H}^{k-1}_\sigma (\Sigma )}\\&+ \left\Vert \widehat{\mathbb {L}}_{g_b}(\sigma )(\mathring{\chi } u)\right\Vert _{\underline{H}^{k-1}_\sigma (\Sigma )} +\left\Vert u\right\Vert _{\underline{LE}^{k-1}(\Sigma )}, \end{aligned} \end{aligned}$$

where we recall the definition of the $LE^k$ norm defined in Definition 36. We see here that this is not enough to conclude, since we have to commute the cutoff functions with $\widehat{\mathbb {L}}_{g_b}(\sigma )$, producing commutation error terms in $\underline{H}^{k}_\sigma (\Sigma )$ that can not necessarily be controlled by the left-hand side. To achieve the desired estimate on the entire exterior region then, we will rely on using the multipliers constructed in the proofs of Theorems 8.2, 8.3, 8.5, and 8.9, along with carefully chosen cutoffs, so that by gluing all the multipliers (multiplied by the appropriately chosen cutoffs), we can prove Theorem 8.1. The main difficulty will come from carefully considering the regions where the various cutoffs are non-constant, where $\widehat{\mathbb {L}}_{g_b}(\sigma )$ does not commute with the cutoffs, and showing that for appropriately chosen largeness and smallness constants, we can glue together the multipliers to prove Theorem 8.1.

Recall that in the slowly-rotating regime, the redshift regions and the trapping regions are physically disjoint from one another. We then divide our analysis into two components: one analyzing the intersection of the trapping and non-trapping regions, and one analyzing the intersection of the non-trapping and redshift regions.

To glue the multipliers together, consider that

238 $$\begin{aligned}&\left\langle \chi \mathbb {L}_{g_b} {h},(X+q)(\chi {h})\right\rangle _{L^2(\mathcal {D})} \nonumber \\ ={}&\left\langle \left[ \chi , \mathbb {L}_{g_b}\right] {h}, (X+q)(\chi {h})\right\rangle _{L^2(\mathcal {D})} + \left\langle \mathbb {L}_{g_b}(\chi {h}),(X+q)(\chi {h})\right\rangle _{L^2(\mathcal {D})}. \end{aligned}$$

Letting $\chi$ be one of the cutoff functions defined at the beginning of the section, and $X, q$ be a pair of the multipliers constructed in the earlier sections, we see that the second term on the right-hand side of equation (238) can already be controlled by one of Theorems 8.2, 8.3, 8.5, 8.9. It remains to choose the $\chi$ in such a way such that the commutator term is also controlled and does not disrupt the ellipticity generated by the previously proven Morawetz estimates.

#### Remark 38

Recalling from Lemma 3.4 that $\mathbb {L}_{g_b}$ is strongly hyperbolic, we observe that

$$\begin{aligned} [\chi , \mathbb {L}_{g_b}] = [\chi , \Box _{g_{b}}] + [\chi , \textbf{S}_b] + [\chi , \textbf{V}_b]. \end{aligned}$$

For any smooth, compactly supported $\chi =\chi (r)$, such that the support of $\partial _r\chi$ is supported away from the trapped set, $[\chi , \textbf{S}_b] + [\chi , \textbf{V}_b]$ is bounded as an operator on $L^2(\mathcal {D})\rightarrow L^2(\mathcal {D})$, they can be controlled by a high-frequency argument in the combined Morawetz estimate. Thus, it will suffice in what follows to consider the error term produced by $[\chi , \Box _{g_{b}}]$. The same result is true when considering the commutation $[\chi , \overline{\mathbb {L}}_{g_b}]$.

We will first show that we can combine the Morawetz estimates in the non-trapping region and the trapping region.

#### Proposition 8.25

Let $g_b$ be a fixed slowly-rotating Kerr-de Sitter background, and letThen for *u* compactly supported on $\Sigma$ and $k\ge 1$, there exist constants ${\boldsymbol{\alpha }}, C_0>0$ such that we have the following resolvent estimate for $\textbf{M}\ge \Im \sigma \ge -{\boldsymbol{\alpha }}, \left\lvert \sigma \right\rvert > C_0$,239

As mentioned previously, we will prove this proposition by combining the vectorfields and Lagrangian correctors used to prove Theorems 8.3, 8.5, and 8.9. Recall that in handling the Morawetz estimates in a neighborhood of $r=3M$, we needed to localize to a neighborhood of ${\Gamma }_{b}$ in both physical space and frequency space. Now that we are considering *h* without compact support in a neighborhood of ${\Gamma }_{b}$, we will need to add both physical cutoffs and frequency cutoffs and show that we can combine the resolvent estimates in the trapping and non-trapping regimes.

Recalling that on $\mathbb {R}^+\times (\mathring{r}_{ -},\mathring{R}_{ +}) \times \mathbb {S}^2$, the $(t_*, r, \theta ,{\varphi _*})$ coordinates agree with the Boyer-Lindquist coordinates, we work in the $(t,r,\theta ,\varphi ;\sigma ,\xi ,\eta _{\theta }, \eta _{\varphi })$ Boyer-Lindquist coordinates in what follows.

Observe that the cutoff functions $\chi \in \Psi ^0(\mathcal {M})$ will not affect our integration-by-parts arguments. As such, we can repeat the proofs of Theorems 8.3, 8.5, 8.9 with $\breve{\chi }{h}$, $\mathring{\chi }\breve{\chi }_{\zeta }{h}$, and $\mathring{\chi }\mathring{\chi }_{\zeta }{h}$ in place of *h* respectively. The resulting commutation error is handled in the following lemma.

#### Lemma 8.26

There exists a choice of constants $\breve{c}, \check{c}, \mathring{c}$, such that for vectorfields $\breve{X}, \check{X}, X_b$, and Lagrangian correctors $\breve{q}, \check{q}, q_{b}$ as defined above such that240on $r\in (\breve{r}_{-}, \breve{R}_{+})$, where

$$\begin{aligned} \mathfrak {k}^{\varkappa ,\mathfrak {q}} {:=} \frac{1}{2\mathbbm {i}}H_{p_b}\varkappa + p_b\mathfrak {q}- \overline{\textbf{s}}_{b,a}\varkappa . \end{aligned}$$

andand

#### Proof

Recall that from Theorems 8.3 , 8.5, and 8.9, we have already proven the lemma on the regions where the cutoff functions are all constant. The main difficulty is to account for the error terms that arise from using these cutoffs.

These terms are of the form $H_{p_b}\chi$, where $\chi$ is a cutoff function (see Remark 38). Throughout the proof, we will normalize the constants $\breve{c}$, $\check{c}$, and $\mathring{c}$ so that $\breve{c}=1$. The cutoff errors fall into three cases.

1. The errors arising from physically gluing together the resolvent estimates in a frequency neighborhood of ${\Gamma }_b$, i.e. on the intersection of the supports of $\breve{\chi }$, $\mathring{\chi }$, and $\mathring{\chi }_\zeta$. To handle this case, we will choose $\breve{C}\gg \mathring{c}, \delta _r^{-1}$.
2. The errors arising from physically gluing together the resolvent estimates outside of a frequency neighborhood of ${\Gamma }_b$, i.e. on the intersection of the supports of $\mathring{\chi }, \breve{\chi }$, and $\breve{\chi }_\zeta$. To handle this case, we will choose $\breve{C}\gg \check{c}, \delta _\zeta ^{-1}$.
3. The errors arising from gluing together the resolvent estimates in a frequency space in a neighborhood of $r=3M$, i.e. on the intersection of the supports of $\mathring{\chi }_\zeta$, $\breve{\chi }_\zeta$, and $\mathring{\chi }$. To handle this case, we will choose $\mathring{c} \ll \frac{1}{\delta _\zeta }$ and $a, \delta _r \ll \frac{\mathring{c}}{\delta _\zeta }$.

It suffices to address each of these cases separately.

**Step 1: Physical gluing in a frequency neighborhood of** ${\Gamma }$. DefineThen we will show that we can find $\mathring{c}$ and $\breve{C}_\circ$, such thatUsing the constructions of $\breve{\chi }$, $\mathring{\chi }$, it suffices to prove that for any $\delta >0$, there exists a choice of $\mathring{c}, \breve{C}$ such that for $\frac{\xi ^2}{\left\lvert \eta \right\rvert ^2}\le 2\delta _\zeta ^2$,

241 $$\begin{aligned} \delta \mathring{c} \mathfrak {k}^{\varkappa _b, \mathfrak {q}_b} \ge -\breve{\varkappa }H_{p_b}(\breve{\chi }^{2}),&\qquad r\in (\breve{r}_{ +}, 3M-\delta _r)\bigcup (3M+\delta _r,\breve{R}_{-}), \end{aligned}$$

242 $$\begin{aligned} \delta \mathfrak {k}^{\breve{\varkappa }, \breve{\mathfrak {q}}} \ge \mathring{c}\left\lvert \varkappa _b H_{p_b}(\mathring{\chi }^{2})\right\rvert ,&\qquad r\in (\mathring{r}_{ -}-\delta _r, \mathring{r}_{ -})\bigcup (\mathring{R}_{ +}, \mathring{R}_{ +}+\delta _r). \end{aligned}$$

Observe that

243 $$\begin{aligned} \breve{\varkappa }H_{p}\breve{\chi } = e^{\breve{C}(r-3M)^2}(r-3M)\partial _r\breve{\chi } (H_{p_b}r)^2, \end{aligned}$$

and that moreover, $(r-3M)\partial _r\breve{\chi }\ge 0$ since $\partial _r\breve{\chi }$ is supported away from $r=3M$. As a result, $\breve{\varkappa }H_{p}\breve{\chi }\ge 0$, and (241) follows immediately.

It remains to show (242). To this end, observe that $\breve{\chi }=1$ on ${\text {supp}}\,\partial _r\mathring{\chi }$, and that $\left\lvert \varkappa _b H_{p_b}(\mathring{\chi }^2)\right\rvert$ is a bounded second order symbol. Then, using Lemma 8.4, we can pick $\breve{C}$ sufficiently large so that (242) holds.

**Step 2: Physical gluing in a frequency neighborhood away from** ${\Gamma }$. Next we handle the error rising from physically gluing together the resolvent estimates microlocalized away from ${\Gamma }_b$. To make this more precise, considerThen we will show that we can find $\check{c}$, $\check{C}$ such that for $\frac{\xi ^2}{\left\lvert \eta \right\rvert ^2}\ge \delta _\zeta ^2$.

244 $$\begin{aligned} \delta \check{c} \mathfrak {k}^{\check{\varkappa }, \check{\mathfrak {q}}} \ge -\breve{\varkappa }H_{p_b}(\breve{\chi }^{2}),&\qquad r\in (\breve{r}_{ +}, 3M-\delta _r)\bigcup (3M+\delta _r,\breve{R}_{-}), \end{aligned}$$

245 $$\begin{aligned} \delta \mathfrak {k}^{\breve{\varkappa }, \breve{\mathfrak {q}}} \ge \check{c}\left\lvert \check{\varkappa }H_{p_b}(\mathring{\chi }^{2})\right\rvert ,&\qquad r\in (\mathring{r}_{ -}-\delta _r, \mathring{r}_{ -})\bigcup (\mathring{R}_{ +}, \mathring{R}_{ +}+\delta _r). \end{aligned}$$

Using (243), we see that

$$\begin{aligned} \breve{\varkappa }H_{p_b}(\breve{\chi }^2) \ge 0, \end{aligned}$$

so on the region of interest, (244) is immediately satisfied.

To prove (245), we observe that $H_{p_b}(\mathring{\chi }^2)$ is a bounded operator, then with the choice $\check{C}= \breve{C}$,

$$\begin{aligned} \left\lvert \check{\varkappa }H_{p_b}(\mathring{\chi }^2)\right\rvert = \left\lvert e^{\breve{C}(r-3M)^2}(r-3M)(H_{p_b}r)H_{p_b}(\mathring{\chi }^2)\right\rvert . \end{aligned}$$

Then we see that since we are only considering $\frac{\xi ^2}{\left\lvert \eta \right\rvert ^2}\ge \delta _\zeta ^2$, we can pick some $\breve{C}$ sufficiently large depending on $\delta _{\zeta }$ and $\delta$ such that

$$\begin{aligned} \left\lvert (r-3M)(H_{p_b}r)H_{p_b}(\mathring{\chi }^2)\right\rvert < \delta \left( 2\breve{C}(r-3M)^2+1\right) \left\lvert H_{p_b}r\right\rvert ^2, \end{aligned}$$

As a result, we can take $\check{c}=1$ and $\check{C}= \breve{C}$, and see that (245) is satisfied.

**Step 3: Gluing together the frequency cutoffs.** Having dealt with the errors arising from the spatial cutoff functions, it remains to deal with the errors from the frequency cutoffs. Because on $\breve{\Sigma }$ we do not divide the analysis into two frequency cases, we only have to deal with the error arising from using the frequency cutoff on $\mathring{\Sigma }$. That is, defineThen, we will show that there exists a choice of constants such thatUsing the construction of the cutoffs, it suffices to show that

246 $$\begin{aligned} \delta \mathring{c}\textbf{k}^{\varkappa _b, \mathfrak {q}_b} >&-\check{c}\check{\varkappa }H_{{\rho _b^2 p}_b}(\breve{\chi }_{\zeta }^2), \qquad {\text {supp}}\,\mathring{\chi }_{\zeta }, \end{aligned}$$

247 $$\begin{aligned} \delta \check{c}\textbf{k}^{\check{\varkappa }, \check{\mathfrak {q}}} >&\left\lvert \mathring{c}\varkappa _b H_{p_b} (\mathring{\chi }_{\zeta }^2)\right\rvert , \qquad {\text {supp}}\,\breve{\chi }_{\zeta }. \end{aligned}$$

We first ensure that (247) holds simply by choosing some $\mathring{c}$ sufficiently small.

To show that (246) also holds, we will show that in fact

$$\begin{aligned} \check{\varkappa }H_{{\rho _b^2 p}_b}(\breve{\chi }_{\zeta }^2) > - O(a)\left\lvert \zeta \right\rvert ^2 - O(\delta _r)\xi ^2, \end{aligned}$$

so that for *a* and $\delta _r$ sufficiently small, (246) is always satisfied. To this end, it will be convenient to observe that

248 $$\begin{aligned} H_{{\rho _b^2 p}_b}\breve{\chi }_{\zeta } = \partial _\xi \breve{\chi }_{\zeta }\frac{\xi H_{{\rho _b^2 p}_b}\xi }{\left\lvert \eta \right\rvert ^2} + \partial _{\eta }\breve{\chi }_{\zeta } \xi ^2 S^{-1}(r,\theta ,\varphi ;\eta _{\theta },\eta _{\varphi }). \end{aligned}$$

Now observe that

$$\begin{aligned} \check{\varkappa }H_{{\rho _b^2 p}_b}(\breve{\chi }_\zeta ^2) = 2\breve{\chi }_{\zeta }e^{\check{C}(r-3M)^2}\frac{r-3M}{\rho _b^2}\Delta _b\xi \left( \partial _\xi \breve{\chi }_{\zeta }\frac{\xi H_{{\rho _b^2 p}_b}\xi }{\left\lvert \eta \right\rvert ^2} + \partial _{\eta }\xi ^2 S^{-1}(r,\theta ,\varphi ;\eta _{\theta },\eta _{\varphi })\right) . \end{aligned}$$

$$\begin{aligned} H_{{\rho _b^2 p}_b}\xi&= -\partial _r\Delta _{b}\xi ^2 + \partial _r\left( \frac{(1+\lambda _b)^2}{\Delta _b}\left( (r^2+a^2)\sigma + a\eta _{\varphi }\right) ^2\right) , \end{aligned}$$

where from Lemma 7.8 we know that

$$\begin{aligned} (r-\mathring{r}_b)\partial _r\left( \frac{(1+\lambda _b)^2}{\Delta _b}\left( (r^2+a^2)\sigma + a\eta _{\varphi }\right) ^2\right) \ge 0, \end{aligned}$$

with vanishing exactly at ${\Gamma }_b$. Observing that

$$\begin{aligned} \left\lvert \mathring{r}_b - 3M\right\rvert \lesssim a, \end{aligned}$$

we then have that

$$\begin{aligned} \check{\varkappa }H_{{\rho _b^2 p}_b}(\breve{\chi }_\zeta ^2)&= 2\frac{\breve{\chi }_\zeta \Delta _b}{\rho _b^2}e^{\check{C}(r-3M)^2}\left( \frac{\partial _\xi \breve{\chi }_\zeta \xi ^2}{\left\lvert \eta \right\rvert ^2}(r-\mathring{r}_b)\partial _r\left( \frac{(1+\lambda _b)^2}{\Delta _b}\left( (r^2+a^2)\sigma + a\eta _{\varphi }\right) ^2\right) \right) \\&+ O(\delta _r)\xi ^2 + aS^2, \end{aligned}$$

where on the domain of interest,

$$\begin{aligned} \frac{\partial _\xi \breve{\chi }_\zeta \xi ^2}{\left\lvert \eta \right\rvert ^2}(r-\mathring{r}_b)\partial _r\left( \frac{(1+\lambda _b)^2}{\Delta _b}\left( (r^2+a^2)\sigma + a\eta _{\varphi }\right) ^2\right) \ge 0,\\ \frac{\breve{\chi }_\zeta \Delta _b}{\rho _b^2}e^{\check{C}(r-3M)^2} > 0. \end{aligned}$$

This concludes the proof of Lemma 8.26. $\square$

Since we are proving a resolvent estimate on a region including the trapped set, we need two integration-by-parts arguments to prove a resolvent estimate on $\textbf{M}\ge \Im \sigma \ge -{\boldsymbol{\alpha }}$. We need one integration-by-parts argument using $(X_b, q_b, 0)$ as multipliers in a neighborhood of the trapped set to prove the resolvent estimate for $\left\lvert \Im \sigma \right\rvert \le {\boldsymbol{\alpha }}$, and one integration-by-parts argument using $(\textbf{T}, 0, 0)$ as multipliers in a neighborhood of the trapped set to prove the resolvent estimate for $\Im \sigma > \frac{{\boldsymbol{\alpha }}}{2}$.

Thus we also need the following analogue of Lemma 8.26, which allows us to glue together the nontrapping Morawetz estimate and the trapping Morawetz estimate when $\Im \sigma \ge \frac{{\boldsymbol{\alpha }}}{2}$.

#### Lemma 8.27

Fix $\delta _0$. Then for $\varepsilon _{{\Gamma }}$ sufficiently small, there exists a choice of constants $\breve{c}, \check{c}, \mathring{c}$, such that for the vectorfields $\breve{X}$ and $\check{X}$, and Lagrangian correctors $\breve{q}$ and $\check{q}$ as defined above,249on $r\in (\breve{r}_{-}, \breve{R}_{+})$, whereand

The proof of Lemma 8.27 is identical to that of 8.26. Since the gluing procedure is also identical for the two cases, we only explicitly work out the procedure for the case where we use $(X_b, q_b, 0)$ to prove a resolvent estimate for $\left\lvert \Im \sigma \right\rvert \le {\boldsymbol{\alpha }}$.

We are now ready to handle the proof of Proposition 8.25.

#### Proof of Proposition 8.25

Definewhere

$$\begin{aligned} \breve{\mathfrak {E}}(t_*)[\breve{\chi }h]&= \int _{\Sigma _{t_*}} J^{\breve{X}, \breve{q}, 0}[\breve{\chi }h]\cdot n_{\Sigma _{t_*}},\\ \check{\mathfrak {E}}(t_*)[\mathring{\chi }\breve{\chi }_\zeta h]&= \int _{\Sigma _{t_*}} J^{\check{X}, \check{q}, 0}[\mathring{\chi }\breve{\chi }_\zeta h] \cdot n_{\Sigma _{t_*}},\\ \mathring{\mathfrak {E}}(t_*)[\mathring{\chi }\mathring{\chi }_\zeta h]&= \int _{\Sigma _{t_*}} J^{X_b, q_b, 0}[\mathring{\chi }\mathring{\chi }_\zeta h] \cdot n_{\Sigma _{t_*}}. \end{aligned}$$

Then, using the pseudo-differential modification of the divergence theorem in (135), we have then that up to lower-order terms,250We will discuss neither the treatment of the boundary terms nor of the lower-order terms in detail here, having already provided a treatment of them in the preceding sections^24^. The terms on the left-hand side generate positive coercive (and degenerate at ${\Gamma }_b$) terms. It suffices then to show that it is possible to choose the vectorfield multipliers, the Lagrangian correctors, and the real weights $\breve{c}, \check{c}, \mathring{c}$ so that the commutation errors arising from commuting the cutoffs with $\mathbb {L}_{g_b}$ on the right-hand side are controlled by the ellipticity of the left-hand side. Recall that since we are only interested in obtaining a high-frequency Morawetz estimate and $\mathbb {L}_{g_b}$ is strongly hyperbolic, it is sufficient to consider the commutation of the cutoffs with $\Box _{g_{b}}$ (see Remark 38). To be more precise, we only need to show that there exists constants $\breve{c}, \check{c}, \mathring{c}$ such that for *h* spatially supported in ,

$$\begin{aligned}&\left\langle \left[ \Box _{g_{b}}, \breve{c}\breve{\chi }\right] {h}, \breve{X}(\breve{\chi }{h})\right\rangle _{L^2(\mathcal {D})} + \left\langle \left[ \rho _b^2\Box _{g_{b}}, \check{c}\mathring{\chi }\breve{\chi }_{\zeta }\right] {h}, \rho _b^{-2}\check{X}(\mathring{\chi }\breve{\chi }_{\zeta }{h})\right\rangle _{L^2(\mathcal {D})}\\&+\left\langle \left[ \Box _{g_{b}}, \mathring{c}\mathring{\chi }\mathring{\chi }_{\zeta }\right] {h}, X_b(\mathring{c}\mathring{\chi }\mathring{\chi }_{\zeta }{h})\right\rangle _{L^2(\mathcal {D})}\\ \le {}&\delta \left( \int _{\mathcal {D}} \breve{c} a\Re K^{\breve{X}, \breve{q}, 0}[\breve{\chi }{h}] + \check{c}K^{\check{X}, \check{q}, 0}[\mathring{\chi }\breve{\chi }_{\zeta }{h}] + \mathring{c} K^{X_{b_0}, q_{b_0}, 0}[\mathring{\chi }\mathring{\chi }_{\zeta }{h}] + \mathring{c}\mathbb {K}^{\widetilde{X}, \tilde{q}}[\mathring{\chi }\mathring{\chi }_{\zeta }{h}]\right) \\&+\delta \left( \breve{c}\Re \left\langle \breve{X}(\breve{\chi }h), \textbf{S}_b[\breve{\chi }h]\right\rangle _{L^2(\mathcal {D})} + \check{c}\Re \left\langle \check{X}(\mathring{\chi }\breve{\chi }_\zeta h), \textbf{S}_b[\mathring{\chi }\breve{\chi }_\zeta h]\right\rangle _{L^2(\mathcal {D})}\right) \end{aligned}$$

for some $0<\delta <1$, but this is exactly the content of Lemma 8.26, so we conclude after a simple application of Cauchy-Schwarz. $\square$

Next, we move onto handling the intersection of the redshift and the non-trapping regions.

#### Proposition 8.28

Let $g_b$ be a fixed slowly-rotating Kerr-de Sitter background, and letThen for $k>k_0$, where $k_0$ is the threshold regularity level in (79), there exist constants ${\boldsymbol{\alpha }}, C_0>0$ such that for *u* compactly supported in ,251

Gluing together the non-trapping Morawetz estimate and the redshift estimate is less nuanced than gluing together the trapping Morawetz estimate and the non-trapping Morawetz estimate. We no longer have frequency-dependent multipliers and thus can glue the two estimates together using physical space methods. In addition, we only have one cutoff function to handle instead of two. The main difficulty with gluing the non-trapping Morawetz estimate and the redshift estimate together then is that the Morawetz estimate degenerates in derivatives transverse to the horizons at the horizons, but this is exactly in the region where the redshift estimate is coercive and positive definite. We first let

252 $$\begin{aligned} \mathbb {L}_{g_b}^{(k)} {:=} \Box _{g_{b}} + \textbf{S}_{b}^{(k)} + \textbf{V}_{b}^{(k)} \end{aligned}$$

denote the linear operator that results from commuting *k* times $\mathbb {L}_{g_b}$ with $\{\mathcal {K}_i\}$ as in Theorem 6.6.

To proceed with the resolvent estimate, we first prove the desired positivity of the bulk terms.

#### Lemma 8.29

Let *g* be a fixed slowly-rotating Kerr-de Sitter background, let $\breve{X}, \breve{q}$ be as constructed in (156), (162), and fix some $\dot{c}>0$. Then for *h* supported outside $\mathring{\Sigma }$, there exists $\breve{C}_\star >0$ such that for $\breve{C}>\breve{C}_\star$, and $\varepsilon _{\mathcal {M}}$ sufficiently small, and $k>k_0$, where $k_0$ is the threshold regularity level defined in (79), such that on $\mathcal {M}\bigcap \{r<r_0, r>R_0\}$,

253 $$\begin{aligned} K^{\breve{X}, \breve{q}, 0}[{h}] + \dot{c}K^{\textbf{N}, 0, 0}[{h}] -\Re \left[ \textbf{S}_b^{(k)}[{h}]\cdot (\breve{X}+ \dot{c}\textbf{N})\overline{{h}}\right] - \Re \left[ \dot{c}\left[ \Box _{g_{b}}, \dot{\chi }\right] h\cdot \dot{\chi }\textbf{N}\overline{h}\right] \gtrsim \left\lvert \nabla h\right\rvert ^2 - C\left\lvert h\right\rvert ^2, \end{aligned}$$

where $\textbf{S}_b^{(k)}$ is the subprincipal operator of $\mathbb {L}_{g_b}^{(k)}$, as in (252).

#### Proof

We decompose

$$\begin{aligned} \textbf{S}_b^{(k)} = \textbf{S}_{b, \mathcal {H}}^{(k)}\widehat{R}+ \widetilde{\textbf{S}}_b^{(k)}, \end{aligned}$$

where $\widetilde{\textbf{S}}_b^{(k)}$ is tangent to both $\mathcal {H}^+$ and $\overline{\mathcal {H}}^+$, and we know that for $k>k_0$, $\overline{\xi }\cdot \textbf{S}_{b, \mathcal {H}}^{(k)}\xi >0$.

Similarly, we decompose for $\mathcal {H}= \mathcal {H}^+, \overline{\mathcal {H}}^+$,

$$\begin{aligned} {\textbf{N}}\big \vert _{\mathcal {H}} = -\widehat{R}+ \widetilde{\textbf{N}}, \end{aligned}$$

where $\widetilde{\textbf{N}}$ is tangent to both $\mathcal {H}^+$ and $\overline{\mathcal {H}}^+$.

Observe that for $k>k_0$, we have by the choice of $\textbf{N}$ in Proposition 2.7 that there exists some $\varepsilon _{\mathcal {H}}>0$ such that

$$\begin{aligned} K^{\textbf{N}, 0, 0}[h] - \Re \left[ \textbf{S}_b^{(k)}[h]\cdot \textbf{N}\overline{h}\right] > \varepsilon _{\mathcal {H}}\left\lvert \nabla h\right\rvert ^2. \end{aligned}$$

We also assume that we have already chosen $\breve{C}$ large enough that

$$\begin{aligned} K^{\breve{X},\breve{q}, 0}[h] > 4\dot{c} \left\lvert \partial _r\dot{\chi }\Delta \widehat{R}h \cdot \widetilde{\textbf{N}} \overline{h}\right\rvert + 4 \left\lvert \widetilde{\textbf{S}}_{b}^{(k)}[h]\cdot \breve{X}\overline{h}\right\rvert . \end{aligned}$$

We see that it then suffices to choose $\breve{C}$, $\dot{c}$, and $\delta _{\mathcal {H}}$ such that

254 $$\begin{aligned} K^{\breve{X}, \breve{q}, 0}[h] > - 4 \dot{c}\partial _r\dot{\chi }\Delta _b\left\lvert \widehat{R}h\right\rvert ^2, \qquad&{\text {supp}}\,\partial _r\dot{\chi }, \end{aligned}$$

255 $$\begin{aligned} K^{\breve{X}, \breve{q}, 0}[h] + \dot{c}\varepsilon _{\mathcal {H}}\left\lvert \nabla h\right\rvert ^2 > 4 e^{\breve{C}(r-3M)^2}\left\lvert \Delta _b (r-3M)\textbf{S}_{b,\mathcal {H}}^{(k)}\right\rvert \left\lvert \widehat{R}h\right\rvert ^2, \qquad&\{r:\dot{\chi }=1\}. \end{aligned}$$

We first consider (255). If $\Delta _b > \frac{4}{\breve{C}}$, then (255) follows directly from the form of $K^{\breve{X}, \breve{q}, 0}[h]$. On the other hand, if $\Delta _b < \frac{4}{\breve{C}}$, then we choose $\dot{c}$ such that

256 $$\begin{aligned} \dot{c} > e^{\breve{C}(r-3M)^2}\left\lvert \frac{(r-3M)\textbf{S}_{b,\mathcal {H}}^{(k)}}{\varepsilon _{\mathcal {H}} \breve{C}}\right\rvert , \end{aligned}$$

so that (255) is verified.

We now turn our attention to (254). Recalling the form of $K^{\breve{X}, \breve{q}, 0}[h]$, and using the fact that our choice of $\dot{c}$ satisfies (256), we have that (254) is satisfied if

$$\begin{aligned} \Delta _b > 16\left\lvert \frac{\textbf{S}_{b,\mathcal {H}}^{(k)} \partial _r\dot{\chi } }{\varepsilon _{\mathcal {H}}\breve{C}^2(r-3M)}\right\rvert ,\qquad {\text {supp}}\,\partial _r\dot{\chi }. \end{aligned}$$

But from the definition of $\dot{\chi }$ in (145), we see that we have the bound

$$\begin{aligned} \left\lvert \partial _r\dot{\chi }\right\rvert < \delta _{\mathcal {H}}^{-2}. \end{aligned}$$

For $\delta _{\mathcal {H}}$ sufficiently small, we have that

$$\begin{aligned} \sup _{{\text {supp}}\,\partial _r{\dot{\chi }}}\Delta _b > \delta _{\mathcal {H}}^2. \end{aligned}$$

Then we take $\breve{C}$ sufficiently large so that

$$\begin{aligned} \breve{C}^2 > 16\left\lvert \frac{\textbf{S}_{b,\mathcal {H}}^{(k)}}{\varepsilon _{\mathcal {H}}(r-3M)\delta _{\mathcal {H}}^4}\right\rvert , \end{aligned}$$

so that (254) is verified. $\square$

We can now use the positivity in Lemma 8.29 to glue together the red-shift and nontrapping Morawetz estimates for the rescaled problem.

#### Lemma 8.30

For $\breve{X}$ and $\breve{q}$ as constructed in Lemma 8.4, there exists an auxiliary one-form $\breve{m}$ and a $\dot{c}>0$ such that the following properties hold.

1. On the hypersurfaces $\mathcal {H}^+_-, \overline{\mathcal {H}}^+_+$, the boundary flux has the following properties:
  257 $$\begin{aligned} \begin{aligned} \dot{c}\int _{\mathcal {H}^+_-}J^{\textbf{N}, 0, 0}[h]\cdot n_{\mathcal {H}^+_-} + \int _{\mathcal {H}^+_-}J^{\breve{X}, \breve{q}, \breve{m}}[h]\cdot n_{\mathcal {H}^+_-}&\ge 0,\\ \dot{c}\int _{\overline{\mathcal {H}}^+_+}J^{\textbf{N}, 0, 0}[h]\cdot n_{\overline{\mathcal {H}}^+_+} +\int _{\overline{\mathcal {H}}^+_+}J^{\breve{X}, \breve{q}, \breve{m}}[h]\cdot n_{\overline{\mathcal {H}}^+_+}&\ge 0. \end{aligned} \end{aligned}$$
2. For *h* supported away from $r=3M$, the inequality in (159) continues to hold with $K^{\breve{X}, \breve{q}, \breve{m}}[h]$ in place of $K^{\breve{X}, \breve{q}, 0}[h]$. For $\varepsilon _{\mathcal {M}}$ sufficiently small, we have moreover that on all of $\Sigma$ as defined in (8), the following relation holds up to zero-order terms
  258 $$\begin{aligned} \left\lvert \nabla h\right\rvert ^2\lesssim {}&\dot{c}K^{\textbf{N}, 0, 0}[\dot{\chi }h] + K^{\breve{X}, \breve{q},\breve{m}}[h]\nonumber \\&- \Re \left[ \textbf{S}_b^{(k)}h\cdot \left( \dot{c}\textbf{N}\circ \dot{\chi }+ \breve{X}\right) \overline{h}\right] + \Re \left[ \dot{c}\left[ \Box _{g_{b}}, \dot{\chi }\right] h\cdot \textbf{N}(\dot{\chi }\overline{h})\right] , \end{aligned}$$
  where $\textbf{S}_b^{(k)}$ is the subprincipal operator of $\mathbb {L}_{g_b}^{(k)}$ as defined in (252), $k>k_0$ the threshold regularity level as defined in (79), and $\dot{\chi }$ is the cutoff localizing to the redshift region as defined in (146).

#### Proof

We first prove (258). First, observe that it suffices to show (258) with the choice $\breve{m}=0$. Observe that

259 $$\begin{aligned} K^{\breve{X}, \breve{q},\breve{m}}[h] ={} K^{\breve{X}, \breve{q},0}[h] + \Re \left[ \breve{m}_\alpha h\cdot \partial ^\alpha \overline{h}\right] + \frac{1}{2}\left( \nabla \cdot \breve{m}\right) \left\lvert h\right\rvert ^2. \end{aligned}$$

As a result, we have that for any choice of one-form $\breve{m}$ and $\epsilon >0$, there exists some $C(\epsilon )>0$ such that

$$\begin{aligned} K^{\breve{X}, \breve{q},\breve{m}}[h] \le K^{\breve{X}, \breve{q}, 0}[h] + \epsilon \left\lvert \nabla h\right\rvert ^2 + C(\epsilon )\left\lvert h\right\rvert ^2. \end{aligned}$$

But then (258) with the choice choice $\breve{m}=0$ is exactly the statement of Lemma 8.29.

We emphasize that the proof of (258) and in particular the choice of $\dot{c}$ is independent of $\breve{m}$. We now consider $\dot{c}$ fixed and show that there exists some choice of $\breve{m}$ such that (257) holds. We will just prove the statement on $\mathcal {H}^+_-$. A similar argument will suffice to show the conclusion on $\overline{\mathcal {H}}^+_+$.

Recall that $\textbf{N}$ is uniformly timelike on $\mathcal {M}$. Since $n_{\mathcal {H}^+_-}$ is also timelike, we have that on $\mathcal {H}^+_-$,

$$\begin{aligned} J^{\textbf{N}, 0, 0}[h]\cdot n_{\mathcal {H}^+_-} \gtrsim \left\lvert \nabla h\right\rvert ^2. \end{aligned}$$

Now observe that

$$\begin{aligned} \int _{\mathcal {H}^+_-}J^{\breve{X}, \breve{q}, \breve{m}}[h]\cdot n_{\mathcal {H}^+_-} \!=\! \int _{\mathcal {H}^+_-}J^{\breve{X},0, 0}[h]\cdot n_{\mathcal {H}^+_-} \!+\!\int _{\mathcal {H}^+_-}J^{0, \breve{q}, 0}[h]\cdot n_{\mathcal {H}^+_-} \!+\!\int _{\mathcal {H}^+_-}J^{0, 0, \breve{m}}[h]\cdot n_{\mathcal {H}^+_-}. \end{aligned}$$

Since $\Delta _b\widehat{R}$ is timelike beyond the horizons (and vanishes at the horizons), we have that

$$\begin{aligned} \int _{\mathcal {H}^+_-}J^{\breve{X},0, 0}[h]\cdot n_{\mathcal {H}^+_-} \ge 0. \end{aligned}$$

Recall from the definition of $J^{X,q,m}[h]$ in (23) that

260 $$\begin{aligned} J^{0, \breve{q}, \breve{m}}[h]\cdot n_{\mathcal {H}^+_-} = \Re \left[ \breve{q}h\cdot n_{\mathcal {H}^+_-}\overline{h}\right] - \frac{1}{2}n_{\mathcal {H}^+_-} \breve{q}\left\lvert h\right\rvert ^2 + \frac{1}{2}g(n_{\mathcal {H}^+_-}, \breve{m}) \left\lvert h\right\rvert ^2. \end{aligned}$$

If we pick for instance $\breve{m}_\alpha = -\breve{C}'\textbf{N}_\alpha$ then

$$\begin{aligned} {g(n_{\mathcal {H}^+_-}, \breve{m})}\Big \vert _{\mathcal {H}^+_-} > 0. \end{aligned}$$

Picking $\breve{C}'$ sufficiently large, it is clear that the second term on the right-hand side of (260) will be controlled by the last term on the right-hand side of (260). Moreover, for $\breve{C}'$ sufficiently large, we have, using Cauchy-Schwarz, that in fact the first term on the right-hand side of (260) can be controlled

$$\begin{aligned} \left\lvert \breve{q}h \cdot n_{\mathcal {H}^+_-}\overline{h} \right\rvert \lesssim \frac{1}{4}g(n_{\mathcal {H}^+_-}, \breve{m})\left\lvert h\right\rvert ^2 + J^{\breve{X}, 0, 0}[h]\cdot n_{\mathcal {H}^+_-} + \dot{c}J^{\textbf{N}, 0, 0 }[h]\cdot n_{\mathcal {H}^+_-} \end{aligned}$$

on $\mathcal {H}^+_-$. This concludes the proof of Lemma 8.30. $\square$

We are now ready to prove Proposition 8.28.

#### Proof of Proposition 8.28

DefineThen, applying the divergence theorem in (26)

and using the control of the boundary terms along $\mathcal {H}^+_-$, $\overline{\mathcal {H}}^+_+$ present in (257), we have that up to lower order terms261To prove the resolvent estimate in (251), it suffices to differentiate (261) in $\partial _{t_*}$ and multiply both sides by $e^{-2\Im \sigma t_*}$. Higher-order estimates then follow as before by commuting through with $\mathcal {K}_i, \textbf{T}$, and using elliptic estimates. $\square$

We are now ready to prove Theorem 8.9 for $\left\lvert \Im \sigma \right\rvert \le {\boldsymbol{\alpha }}$.

#### Proof of Theorem 8.9

for $\left\lvert \Im \sigma \right\rvert \le {\boldsymbol{\alpha }}$ The theorem follows directly from (261) and (250), using the bulk positivity in Lemmas 8.26 and 8.30, and controlling boundary terms and lower-order terms as previously done. $\square$

The proof of Theorem 8.9 for $\left\lvert \Im \sigma \right\rvert > \frac{{\boldsymbol{\alpha }}}{2}$ follows exactly like the proof of Theorem 8.9 for $\left\lvert \Im \sigma \right\rvert \le {\boldsymbol{\alpha }}$, so we omit it here.

## Exponential Decay Up to Finite-Dimensional Perturbation

The main goal of this section will be to prove Theorems 5.3 and 5.4. Together, these two theorems imply that there are only finitely many non-decaying $\textbf{H}^{k}$-quasinormal mode solutions for $\mathbb {L}=\mathbb {L}_{g_b}$, giving exponential decay up to finite-dimensional perturbation, and in particular, the asymptotic expansion in Corollary 5.5.

### Fredholm Alternative for $\mathcal {A}$ (Proof of Theorem 5.3)

In this section, we will prove Theorem 5.3. We do so by analyzing the invertibility of the Laplace-transformed operator $\widehat{\mathbb {L}}(\sigma )$. By using the Killing energy estimate, the redshift energy estimate, and commuting with the redshift vectorfield, we will be able to show that for sufficiently slowly-rotating Kerr-de Sitter metrics, there exists some $\gamma$ such that $(\widehat{\mathbb {L}}(\sigma ) - \gamma )^{-1}$ is a well-defined, compact operator in the half-plane

262 $$\begin{aligned} \left\{ \sigma \in \mathbb {C}:\Im \sigma > \frac{1}{2}\max _{\mathcal {H}=\mathcal {H}^+, \overline{\mathcal {H}}^+}\left( \textbf{s}_{\mathbb {L}}[\mathcal {H}] -\left( 2k + \frac{1}{2}\right) \kappa _\mathcal {H}\right) \right\} . \end{aligned}$$

An appeal to the analytic Fredholm theorem then allows us to derive the equivalent of Theorem 5.3 for the Laplace-transformed operator, which directly implies Theorem 5.3, despite lacking compactness for $(\mathcal {A}-\sigma )^{-1}$. This follows closely the approach taken by Warnick in proving an equivalent result on asymptotically Schwarzschild anti-de Sitter spacetimes in [62].

In what follows, we first prove estimates for $\widehat{\mathbb {L}}(\sigma )$ with domain $D^1(\widehat{\mathbb {L}}(\sigma ))$, and then for higher regularity domains.

#### Injectivity

We first show that $(\widehat{\mathbb {L}}(\sigma ) - \gamma )^{-1}$ with domain $D^1(\widehat{\mathbb {L}}(\sigma ))$ is injective.

##### Theorem 9.1

For all sufficiently slowly-rotating Kerr-de Sitter metrics $g_b$ where $b=(M, a)$ the following holds. For a fixed compact domain

$$\begin{aligned} \Omega \subset \left\{ \sigma \in \mathbb {C}: \Im \sigma > \frac{1}{2} \max _{\mathcal {H}=\mathcal {H}^+, \overline{\mathcal {H}}^+}\left( \textbf{s}_{\mathbb {L}}[\mathcal {H}] -\frac{1}{2}\kappa _{\mathcal {H}} \right) \right\} , \end{aligned}$$

there exists some $\gamma _1$ such that $\widehat{\mathbb {L}}(\sigma ) - \gamma$ is injective for any $\gamma >\gamma _1$ and $D^1(\widehat{\mathbb {L}}(\sigma ))\subset \underline{H}^{1}(\Sigma )$, for any $\sigma \in \Omega$, and that furthermore we have the estimate

263 $$\begin{aligned} \left\Vert u\right\Vert _{\underline{H}^{1}(\Sigma )} \lesssim \left\Vert (\widehat{\mathbb {L}}(\sigma ) - \gamma )u\right\Vert _{\underline{L}^2(\Sigma )}. \end{aligned}$$

##### Proof

It suffices to prove (263) for smooth *u*. The rest of the conclusions then follow by a density argument.

We first define $P_2, P_1$ as in (56) such that $P_i$ is a bounded differential operators of order *i* such that

$$\begin{aligned} \mathbb {L}u = \frac{1}{A} D_{t_*}^2 u + P_1D_{t_*} u + P_2 u. \end{aligned}$$

We first apply the $\widetilde{\textbf{T}}$-energy estimate in part 2 of Corollary 6.2 to the function $e^{-\mathbbm {i}(\sigma + c)t_*} u(x)$ and multiply both sides by $e^{2\Im c t_*}$ to see that

$$\begin{aligned}&2\Im (c+\sigma )E_\gamma (t_*)[e^{-\mathbbm {i}\sigma t_*}u]\\ \le {}&\epsilon \left( \left\Vert e^{-\mathbbm {i}\sigma t_*} u\right\Vert ^2_{\underline{H}^{1}(\widetilde{\Sigma })} + \left\Vert (\mathbb {L}- \gamma ) (e^{-\mathbbm {i}\sigma t_*} u)\right\Vert _{\underline{L}^2(\widetilde{\Sigma })}^2\right) \\&+ \epsilon \left\Vert \left( cP_1+2cA^{-1}+ c^2A^{-1}\right) e^{-\mathbbm {i}\sigma t_*} u\right\Vert _{\underline{L}^2(\widetilde{\Sigma })}^2 + C(\epsilon ) \left\lvert c+\sigma \right\rvert ^2\left\Vert e^{-\mathbbm {i}\sigma t_*}u\right\Vert _{\underline{L}^2(\widetilde{\Sigma })}^2\\&+ a C(\epsilon )\left\Vert e^{-\mathbbm {i}\sigma t_*}u\right\Vert _{\underline{H}^{1}(\widetilde{\Sigma })}^2 + a\gamma C\left\Vert e^{-\mathbbm {i}\sigma t_*}u\right\Vert _{\underline{L}^2(\widetilde{\Sigma })}^2, \end{aligned}$$

where we recall $\widetilde{\Sigma }$ is as constructed in (4). Now, we choose *c* such that $\Im (c+\sigma )>0$ for all $\sigma \in \Omega$

so that the left-hand side of the inequality is positive. We emphasize that this choice of *c* very much depends on $\Omega$.

Next, recall that $P_1: \underline{H}^{1}(\Sigma )\rightarrow \underline{L}^2(\Sigma )$ is a bounded operator. Thus, given $\delta >0$, there exist constants $C_{\widetilde{\textbf{T}}}(\delta ), C_{\widetilde{\textbf{T}}}$ depending on *c*, $\Omega$ and $P_1$, but independent of $\gamma$ such that for all $\sigma \in \Omega$,

264 $$\begin{aligned}&E_\gamma (t_*)[e^{-\mathbbm {i}\sigma t_*}u]\nonumber \\ \le {}&\delta \left( \left\Vert e^{-\mathbbm {i}\sigma t_*}u\right\Vert ^2_{\underline{H}^{1}(\widetilde{\Sigma })} + \left\Vert (\mathbb {L}- \gamma )(e^{-\mathbbm {i}\sigma t_*} u)\right\Vert ^2_{\underline{L}^2(\widetilde{\Sigma })}\right) \nonumber \\&+ C_{\widetilde{\textbf{T}}}(\delta )\left\Vert e^{-\mathbbm {i}\sigma t_*} u\right\Vert _{\underline{L}^2(\widetilde{\Sigma })}^2 + aC_{\widetilde{\textbf{T}}}(\delta )\left\Vert e^{-\mathbbm {i}\sigma t_*}u\right\Vert _{\underline{H}^{1}(\widetilde{\Sigma })}^2 + aC_{\widetilde{\textbf{T}}}\gamma \left\Vert e^{-\mathbbm {i}\sigma t_*} u\right\Vert ^2_{\underline{L}^2(\widetilde{\Sigma })} . \end{aligned}$$

We now apply the redshift estimate in part 2 of Corollary 6.5 with

$$\begin{aligned} \varepsilon _{\textbf{N}} < \frac{1}{4}\max _{\mathcal {H}= \mathcal {H}^+, \overline{\mathcal {H}}^+}\kappa _{\mathcal {H}} \end{aligned}$$

to $e^{-\mathbbm {i}\sigma t_*}u$ to deduce that

265 $$\begin{aligned}&\left( 2\Im \sigma - \max _{\mathcal {H}=\mathcal {H}^+, \overline{\mathcal {H}}^+}\left( \textbf{s}_{\mathbb {L}}[\mathcal {H}] - \kappa _{\mathcal {H}} + \varepsilon _{\textbf{N}} + \epsilon _0\right) \right) \mathcal {E}_\gamma (t_*)[e^{-\mathbbm {i}\sigma t_*}u] \nonumber \\ \le {}&C^\textbf{N}(\epsilon _0)\left( \left\Vert (\mathbb {L}- \gamma )(e^{-\mathbbm {i}\sigma t_*}u)\right\Vert _{\underline{L}^2(\Sigma )}^2 + \left\Vert e^{-\mathbbm {i}\sigma t_*}u\right\Vert _{\underline{L}^2(\Sigma )}^2 + E_\gamma (t_*)[e^{-\mathbbm {i}\sigma t_*} u]\right) , \end{aligned}$$

where $\epsilon _0 = \frac{1}{4}\max _{\mathcal {H}= \mathcal {H}^+, \overline{\mathcal {H}}^+}\kappa _{\mathcal {H}}$ is chosen so that no matter our choice of $\Omega$,

$$\begin{aligned} 2\Im \sigma - \max _{\mathcal {H}=\mathcal {H}^+, \overline{\mathcal {H}}^+}\left( \textbf{s}_{\mathbb {L}}[\mathcal {H}] - \kappa _{\mathcal {H}}\right) - \varepsilon _{\textbf{N}} - \epsilon _0 > 0. \end{aligned}$$

We will now apply (264) to control the $\widetilde{\textbf{T}}$-energy norm on the right-hand side of (265). To do this, we realize that there exists $\delta _0$ sufficiently small, and independent of our choice of $\Omega$ such that for any $\delta \le \delta _0$,

$$\begin{aligned} \delta C_{\textbf{N}}(\epsilon _0) \left\Vert e^{-\mathbbm {i}\sigma t_*}u\right\Vert _{\underline{H}^{1}(\Sigma )}^2 <\epsilon _0 \mathcal {E}(t_*)[e^{-\mathbbm {i}\sigma t_*}u]. \end{aligned}$$

Now let $C_{\widetilde{\textbf{T}}}(\delta _0)$ be the large constant such that (264) holds with the choice $\delta =\delta _0$. We can then find $a_0$ sufficiently small such that for all $a<a_0$,

266 $$\begin{aligned} aC_{\textbf{N}}(\epsilon _0)C_{\widetilde{\textbf{T}}}(\delta _0)\left\Vert e^{-\mathbbm {i}\sigma t_*}u\right\Vert _{\underline{H}^{1}(\Sigma )}^2< \epsilon _0 \mathcal {E}(t_*)[e^{-\mathbbm {i}\sigma t_*}u],\qquad a C_{\textbf{N}}(\epsilon _0) C_{\widetilde{\textbf{T}}} < \frac{1}{2}. \end{aligned}$$

We observe that this is possible precisely because both $\epsilon _0=\frac{1}{4}\max _{\mathcal {H}= \mathcal {H}^+, \overline{\mathcal {H}}^+}\kappa _{\mathcal {H}}$ and $\delta _0$ were chosen in a manner such that so that they are positive for all *a*, independent of the choice of $\Omega$. As a result, $C_{\textbf{N}}(\epsilon _0), C_{\widetilde{\textbf{T}}}(\delta _0)$ are finite, and we can pick $a_0$ such that for all $a<a_0$, (266) is satisfied by continuity.

Thus, using (264)

to control the $\widetilde{\textbf{T}}$-energy in (265), we have that there exists some $C(\epsilon ,\delta )$ such that

$$\begin{aligned} \mathcal {E}_\gamma (t_*)[e^{-\mathbbm {i}\sigma t_*}u] \le {}&C(\epsilon _0,\delta _0) \left( \left\Vert (\mathbb {L}- \gamma )\left( e^{-\mathbbm {i}\sigma t_*}u\right) \right\Vert _{\underline{L}^2(\Sigma )}^2 + \left\Vert e^{-\mathbbm {i}\sigma t_*}u\right\Vert _{\underline{L}^2(\Sigma )}^2 \right) \\&+ \frac{\gamma }{2}\left\Vert e^{-\mathbbm {i}\sigma t_*}u\right\Vert _{\underline{L}^2(\Sigma )}^2. \end{aligned}$$

Picking $\gamma$ such that $\gamma >2 C(\epsilon _0,\delta _0)$, and observing that

$$\begin{aligned} \mathcal {E}_\gamma (t_*)[{h}] = \mathcal {E}_{\gamma '}(t_*)[{h}] + (\gamma -\gamma ')\left\Vert {h}\right\Vert _{L^2(\Sigma )}^2, \end{aligned}$$

we have in fact that

$$\begin{aligned} \mathcal {E}_{\gamma /2}(t_*)[e^{-\mathbbm {i}\sigma t_*}u] \le C(\epsilon _0, \delta _0) \left\Vert (\mathbb {L}- \gamma )e^{-\mathbbm {i}\sigma t_*}u\right\Vert _{\underline{L}^2(\Sigma )}^2. \end{aligned}$$

Multiplying by $e^{-2\Im \sigma t_*}$, both sides of the inequality become independent of time, and using the formula for the Laplace-transformed operator and (112), we have that

$$\begin{aligned} \left\Vert u\right\Vert _{\underline{H}^{1}_\sigma (\Sigma )}\le C(\epsilon ,\delta ) \left\Vert (\widehat{\mathbb {L}}(\sigma ) - \gamma )u\right\Vert _{\underline{L}^2(\Sigma )}, \end{aligned}$$

as desired, concluding the proof of Theorem 9.1. $\square$

#### Surjectivity

In the previous subsection, we established injectivity of $\widehat{\mathbb {L}}(\sigma ) - \gamma : D^1(\widehat{\mathbb {L}}(\sigma ))\rightarrow L^2(\Sigma )$ for a certain range of $\sigma$. To complete the proof of invertibility, we need to verify surjectivity, which will follow from injectivity of the adjoint operator.

##### Lemma 9.2

Let *A* be a closed, densely defined operator on a Hilbert space *H* with closed range. Then *A* is surjective if and only if the adjoint $A^*$ is injective.

We now move onto the injectivity of $\widehat{L}^*(\sigma ) - \gamma$.

##### Theorem 9.3

For all sufficiently slowly-rotating Kerr-de Sitter metrics $g_b$ where $b=(M, a)$ the following holds. For a fixed compact domain

$$\begin{aligned} \Omega \subset \left\{ \sigma \in \mathbb {C}:\Im \sigma > \frac{1}{2}\max _{\mathcal {H}=\mathcal {H}^+,\overline{\mathcal {H}}^+} \left( \textbf{s}_{\mathbb {L}}[\mathcal {H}] + \frac{3}{2}\kappa _{\mathcal {H}} \right) \right\} , \end{aligned}$$

there exists $\gamma _1$ depending on $\Omega , g_b$, such that $\widehat{\mathbb {L}}^*(\sigma )$ is injective for $\gamma >\gamma _1$ and $D(\widehat{\mathbb {L}}^*(\sigma ))\subset H^1(\Sigma )$ for all $\sigma \in \Omega$. In addition, the following estimate holds:

$$\begin{aligned} \left\Vert u\right\Vert _{\underline{H}^{1}(\Sigma )} \lesssim \left\Vert (\widehat{\mathbb {L}}^*(\sigma ) - \gamma )u\right\Vert _{\underline{L}^2(\Sigma )}. \end{aligned}$$

##### Proof

The outline of the proof closely follows the proof of Theorem 9.1. The main difference lies in that the domain of $\mathbb {L}^*$ consists of functions vanishing along the horizons. Thus instead of applying the estimates in part 2 of Corollary 6.5, we apply the estimates in part 3 of Corollary 6.5.

We apply Corollary 6.2 part 3 to the function $e^{-\mathbbm {i}(\overline{\sigma } + c)t_*}\chi _{\bullet }u$, where $\chi _\bullet = \chi _\bullet (r)$ is that from Part 3 of Theorem 6.3 and *c* is some constant which we will determine later, with $\mathbb {L}^*$ in place of $\mathbb {L}$. Then,

$$\begin{aligned} \begin{aligned}&2\Im (\sigma +c)E_\gamma (t_*)[e^{-\mathbbm {i}\overline{\sigma }t_*} \chi _{\bullet }u] \\ \le {}&\epsilon \left( \left\Vert e^{-\mathbbm {i}\overline{\sigma }t_*} u\right\Vert _{\underline{H}^{1}(\widetilde{\Sigma })}^2 + \left\Vert (\mathbb {L}^* - \gamma )e^{-\mathbbm {i}\overline{\sigma }t_*} u\right\Vert _{\underline{L}^2(\widetilde{\Sigma })}^2\right) \\&+ \epsilon \left( \left\Vert \left[ \mathbb {L}^*, \chi _{\bullet } \right] e^{-\mathbbm {i}\overline{\sigma }t_*}u\right\Vert _{\underline{L}^2(\widetilde{\Sigma })}^2 + \left\Vert \left( cP_1^* + 2cA^{-1} + c^2A^{-1}\right) e^{-\mathbbm {i}\overline{\sigma }t_*} u\right\Vert _{\underline{L}^2(\widetilde{\Sigma })}^2 \right) \\&+C(\epsilon )\left\lvert c+\overline{\sigma }\right\rvert ^2\left\Vert e^{-\mathbbm {i}\overline{\sigma }t_*} u\right\Vert _{\underline{L}^2(\widetilde{\Sigma })}^2 + aC(\epsilon )\left\Vert e^{-\mathbbm {i}\overline{\sigma }t_*} u\right\Vert _{\underline{H}^{1}(\widetilde{\Sigma })}^2 + a\gamma C\left\Vert e^{-\mathbbm {i}\overline{\sigma }t_*}u\right\Vert _{\underline{L}^2(\widetilde{\Sigma })}^2. \end{aligned} \end{aligned}$$

We choose *c* such that $\Im (\sigma +c)>0$ so that the left-hand side is positive. Like in the proof of Theorem 9.1, we use the fact that $P_1^*$ is a bounded map from $\underline{H}^{1}(\Sigma )\rightarrow L^2(\Sigma )$, and that $\epsilon$ can be made arbitrarily small to conclude that given $\delta >0$, there exists $C(\delta )$ such that for any $\sigma \in \Omega$,

267 $$\begin{aligned} E_\gamma (t_*)[e^{-\mathbbm {i}\overline{\sigma }t_*}\chi _{\bullet }(r) u] \le {}&\delta \left( \left\Vert e^{-\mathbbm {i}\overline{\sigma }t_*} u\right\Vert _{\underline{H}^{1}(\widetilde{\Sigma })}^2 + \left\Vert (\mathbb {L}^* - \gamma )e^{-\mathbbm {i}\overline{\sigma }t_*} u\right\Vert _{\underline{L}^2(\widetilde{\Sigma })}^2\right) \nonumber \\&+ a C_{\widetilde{\textbf{T}}}(\delta )\left\Vert e^{-\mathbbm {i}\overline{\sigma }t_*} u\right\Vert _{\underline{H}^{1}(\widetilde{\Sigma })}^2 + a C_{\widetilde{\textbf{T}}}\gamma \left\Vert e^{-\mathbbm {i}\overline{\sigma }t_*} u\right\Vert _{\underline{L}^2(\widetilde{\Sigma })}^2 . \end{aligned}$$

Next, we apply the redshift estimate in part 3 of Corollary 6.5, with $\mathbb {L}^*$ in place of $\mathbb {L}$, and with $\varepsilon _{\textbf{N}}<\frac{1}{4}\max _{\mathcal {H}=\mathcal {H}^+,\overline{\mathcal {H}}^+}\kappa _{\mathcal {H}}$. Recalling that $\textbf{s}_{\mathbb {L}^*}^* = -\textbf{s}_{\mathbb {L}}$, we have that

268 $$\begin{aligned}&\left( 2\Im \sigma - \max _{\mathcal {H}=\mathcal {H}^+, \overline{\mathcal {H}}^+}(\textbf{s}_{\mathbb {L}}[\mathcal {H}] - \kappa _{\mathcal {H}}) - \varepsilon _{\textbf{N}}-\epsilon _0 \right) \mathcal {E}_\gamma (t_*)[e^{-\mathbbm {i}\overline{\sigma }t_*}u] \nonumber \\ \le {}&C_{\textbf{N}}(\epsilon _0)\left( \left\Vert (\mathbb {L}^* - \gamma )e^{-\mathbbm {i}\overline{\sigma }t_*}u\right\Vert _{\underline{L}^2(\Sigma )}^2 + \left\Vert e^{-\mathbbm {i}\overline{\sigma }t_*}u\right\Vert _{\underline{L}^2(\Sigma )}^2 + E_\gamma (t_*)[e^{-\mathbbm {i}\overline{\sigma }t_*}\chi _\bullet (r)u] \right) , \end{aligned}$$

where we choose $\epsilon _0 = \frac{1}{4}\max _{\mathcal {H}=\mathcal {H}^+,\overline{\mathcal {H}}^+}\kappa _{\mathcal {H}}$, so that no matter our choice of $\Omega$,

$$\begin{aligned} 2\Im \sigma -\max _{\mathcal {H}=\mathcal {H}^+,\overline{\mathcal {H}}^+}(\textbf{s}_{\mathbb {L}}[\mathcal {H}]+\kappa _{\mathcal {H}}) - \varepsilon _{\textbf{N}}-\epsilon _0 > 0. \end{aligned}$$

We now use (267) to control the $C(\epsilon )E_\gamma (t_*)[e^{-\mathbbm {i}\overline{\sigma }t_*}u]$ term on the right-hand side of (268). We first recognize that there exists a $\delta _0$ such that for all $\delta <\delta _0$,

$$\begin{aligned} \delta C_{\textbf{N}}(\epsilon _0)\left\Vert e^{-\mathbbm {i}\overline{\sigma }t_*}u\right\Vert _{\underline{H}^{1}(\Sigma )}^2 \le \epsilon _0 \mathcal {E}_\gamma (t_*)[e^{-\mathbbm {i}\overline{\sigma }t_*}u]. \end{aligned}$$

We then see that there exists some $a_0$ sufficiently small so that for all $a<a_0$,

269 $$\begin{aligned} aC_{\textbf{N}}(\epsilon _0)C_{\widetilde{\textbf{T}}}(\delta _0)\left\Vert e^{-\mathbbm {i}\sigma t_*}u\right\Vert _{\underline{H}^{1}(\Sigma )}^2< \epsilon _0 \mathcal {E}(t_*)[e^{-\mathbbm {i}\sigma t_*}u],\qquad a C_{\textbf{N}}(\epsilon _0) C_{\widetilde{\textbf{T}}} < \frac{1}{2}. \end{aligned}$$

As in the proof of Theorem 9.1, we remark that the existence of such an $a_0$ comes from the fact that $\epsilon _0$ is uniformly positive and bounded away from 0 for all *a* sufficiently small. Then we have that

$$\begin{aligned} \mathcal {E}_\gamma (t_*)[e^{-\mathbbm {i}\overline{\sigma }t_*}u] \le {}&C(\epsilon _0, \delta _0)\left( \left\Vert (\mathbb {L}^* - \gamma )e^{-\mathbbm {i}\overline{\sigma }t_*}u\right\Vert _{\underline{L}^2(\Sigma )}^2 +\left\Vert e^{-\mathbbm {i}\overline{\sigma }t_*}u\right\Vert _{\underline{L}^2(\Sigma )}^2\right) \\&+ \frac{\gamma }{2}\left\Vert e^{-\mathbbm {i}\overline{\sigma }t_*}u\right\Vert _{\underline{L}^2(\Sigma )}^2. \end{aligned}$$

Again, we recall that $\mathcal {E}_{\gamma }(t_*)[h] = \mathcal {E}_{\gamma }(t_*)[h] + (\gamma -\gamma ')\left\Vert h\right\Vert _{\underline{L}^2(\Sigma )}$. We then choose $\gamma _0$ such that $\frac{\gamma _0}{2} >C(\epsilon _0,\delta _0)$. The $\underline{L}^2(\Sigma )$ norm on the right-hand side of (267) can then be absorbed to obtain

$$\begin{aligned} \mathcal {E}_{\gamma /2}(t_*)[e^{-\mathbbm {i}\overline{\sigma }t_*}u] \le C(\epsilon ,\delta )\left\Vert (\mathbb {L}^* - \gamma )e^{-\mathbbm {i}\overline{\sigma }t_*}u\right\Vert _{\underline{L}^2(\Sigma )}^2. \end{aligned}$$

for any $\gamma >\gamma _0$. Finally, multiplying both sides by $e^{-2\Im \sigma t_*}$ and using the definition of the Laplace-transformed operator and (112) concludes the proof of Theorem 9.3. $\square$

Given the proofs of injectivity for $\widehat{\mathbb {L}}(\sigma )$ and its adjoint on the region

$$\begin{aligned} \Im \sigma >\max _{\mathcal {H}=\mathcal {H}^+, \overline{\mathcal {H}}^+}\frac{1}{2}\left( \textbf{s}_{\mathbb {L}}[\mathcal {H}] + \frac{3}{2}\kappa _{\mathcal {H}} \right) , \end{aligned}$$

we can now prove invertibility.

##### Theorem 9.4

Let $\mathbb {L}$ be the gauge-fixed Einstein operator linearized around a slowly-rotating Kerr-de Sitter black hole. Fix a compact domain

$$\begin{aligned} \Omega \subset \left\{ \sigma \in \mathbb {C}: \Im \sigma >\frac{1}{2}\max _{\mathcal {H}=\mathcal {H}^+, \overline{\mathcal {H}}^+}\left( \textbf{s}_{\mathbb {L}}[\mathcal {H}] + \frac{3}{2}\kappa _{\mathcal {H}} \right) \right\} . \end{aligned}$$

Then there exists some $\gamma _1$ depending only on $\Omega$, the black-hole parameters (*M*, *a*) such that for $\gamma >\gamma _1$,

$$\begin{aligned} \widehat{\mathbb {L}}(\sigma ) - \gamma : D^1(\widehat{\mathbb {L}}(\sigma ))\rightarrow \underline{L}^2(\Sigma ) \end{aligned}$$

is invertible, and

$$\begin{aligned} (\widehat{\mathbb {L}}(\sigma ) - \gamma )^{-1}: \underline{L}^2(\Sigma )\mapsto \underline{H}^{1}(\Sigma ) \end{aligned}$$

is well-defined.

##### Proof

Theorem 9.1 gives us that for sufficiently large $\gamma$, $(\widehat{\mathbb {L}}(\sigma )-\gamma ):D^1(\widehat{\mathbb {L}}(\sigma ))\rightarrow \underline{L}^2(\Sigma )$ is injective. Then by Theorem 9.3 we also have surjectivity (after potentially increasing $\gamma$ if necessary). This proves the existence of the resolvent $(\widehat{\mathbb {L}}(\sigma ) -\gamma )^{-1}$. The desired estimate is simply a consequence of equation (263). $\square$

##### Remark 39

From Theorem 9.1 we have that $(\widehat{\mathbb {L}}(\sigma )-\gamma ):D^1(\widehat{\mathbb {L}}(\sigma ))\rightarrow \underline{L}^2(\Sigma )$ is injective on $\Im \sigma >\frac{1}{2} \max _{\mathcal {H}=\mathcal {H}^+,\overline{\mathcal {H}}^+} \left( \textbf{s}_{\mathbb {L}}[\mathcal {H}] - \frac{1}{2}\kappa _{\mathcal {H}} \right)$. On the other hand, Theorem 9.3 only gives surjectivity on the region $\Im \sigma > \frac{1}{2} \max _{\mathcal {H}=\mathcal {H}^+,\overline{\mathcal {H}}^+} (\textbf{s}_{\mathbb {L}}[\mathcal {H}] + \frac{3}{2}\kappa _{\mathcal {H}})$. While this appears to be an obstacle to proving invertibility on the entirety of the region $\Im \sigma >\frac{1}{2}\max _{\mathcal {H}=\mathcal {H}^+,\overline{\mathcal {H}}^+}\left( \textbf{s}_{\mathbb {L}}[\mathcal {H}] - \frac{1}{2}\kappa _{\mathcal {H}}\right)$, as we will see in the next section, it is possible to extend the range of invertibility to the full range on which we have shown that $\widehat{\mathbb {L}}(\sigma )-\gamma$ is injective.

#### Extending the Inverse

Recall that in commuting the equation $\mathbb {L}{h}=f$ with the vectorfields $\mathcal {K}_i$ constructed in Lemma 2.8, we were able to recover a strongly hyperbolic operator $\textbf{L}$ satisfying $\textbf{L}{h}= \textbf{f}$ with $\textbf{s}_{\textbf{L}}[\mathcal {H}]$ improved by $2\kappa _{\mathcal {H}}$ with each commutation. This allowed us to prove higher-regularity energy inequalities for $\mathbb {L}{h}=0$. We will now apply these principles to the Laplace-transformed operator to prove that for *f* sufficiently regular, $\widehat{\mathbb {L}}(\sigma )-\gamma$ is invertible on a larger set.

##### Theorem 9.5

For *g* a sufficiently slowly-rotating Kerr-de Sitter metric, let $\mathbb {L}$ denote the gauge-fixed linearized Einstein operator. Moreover, let *k* be a positive integer and fix a compact domain

270 $$\begin{aligned} \Omega \subset \left\{ \sigma \in \mathbb {C}:\Im \sigma >\frac{1}{2}\max _{\mathcal {H}=\mathcal {H}^+,\overline{\mathcal {H}}^+}\left( \textbf{s}_{\mathbb {L}}[\mathcal {H}] - \left( 2k + \frac{1}{2} \right) \kappa _{\mathcal {H}} \right) \right\} . \end{aligned}$$

Then there exists $\gamma _k$ depending on $\Omega , b, k$, such that for $\gamma >\gamma _k$, the equation

271 $$\begin{aligned} (\widehat{\mathbb {L}}(\sigma )-\gamma )u=f \end{aligned}$$

admits a unique solution $u\in \underline{H}^{k}(\Sigma )$ for any $f\in \underline{H}^{k-1}(\Sigma )$. Furthermore, *u* satisfies the estimate

272 $$\begin{aligned} \left\Vert u\right\Vert _{\underline{H}^{k}(\Sigma )} \lesssim \left\Vert f\right\Vert _{\underline{H}^{k-1}(\Sigma )}. \end{aligned}$$

##### Proof

Consider the Laplace-transformed commutators $\widehat{\mathcal {K}}_i: \underline{H}^{k}(\Sigma ) \rightarrow \underline{H}^{k-1}(\Sigma )$, defined by

$$\begin{aligned} \widehat{\mathcal {K}}_i(\sigma )u = \left. e^{\mathbbm {i}\sigma t_*}\mathcal {K}_ie^{-\mathbbm {i}\sigma t_*}u\right| _{\Sigma _{t_*}}. \end{aligned}$$

The theorem is proven in two steps.

The first step to proving the theorem will then be to inductively commute with $\widehat{\mathcal {K}}_i$ to prove that (272) holds for $\sigma$ in a compact domain

273 $$\begin{aligned} \Omega \subset \left\{ \sigma \in \mathbb {C}: \Im \sigma >\frac{1}{2}\max _{\mathcal {H}=\mathcal {H}^+,\overline{\mathcal {H}}^+} \left( \textbf{s}_{L}[\mathcal {H}] - \left( 2k-\frac{3}{2}\right) \kappa _{\mathcal {H}} \right) \right\} . \end{aligned}$$

We will then extend this to the full region in the theorem by taking the higher-order estimates, re-applying them to the lower-order estimates via an approximation argument, and then repeating the induction argument to achieve the desired results.

The $k=0$ case of our induction is the content of Theorem 9.4. Fix $k>0$, and a complex subset of the complex plane $\Omega \subset \bigg \{ \sigma \in \mathbb {C}: \Im \sigma >\max _{\mathcal {H}=\mathcal {H}^+, \overline{\mathcal {H}}^+}(\frac{1}{2}\textbf{s}_{\mathbb {L}}[\mathcal {H}] -\big (k -\frac{3}{4}\big )\kappa _{\mathcal {H}}) \bigg \}$. Assume for the sake of induction that the theorem holds for $k-1$, with

$$\begin{aligned} \Omega \subset \left\{ \sigma \in \mathbb {C}: \Im \sigma >\frac{1}{2}\max _{\mathcal {H}=\mathcal {H}^+, \overline{\mathcal {H}}^+} \left( \textbf{s}_{\mathbb {L}}[\mathcal {H}] -\left( 2k - \frac{7}{2}\right) \kappa _{\mathcal {H}} \right) \right\} . \end{aligned}$$

Commuting (271) with $\widehat{\mathcal {K}}_i$ and applying Theorem 6.6, we can conclude that a solution *u* to (271) must induce a solution $\textbf{u}{:=}(u, \widehat{\mathcal {K}}_i u)$ such that

274 $$\begin{aligned} (\widehat{\textbf{L}}(\sigma )-\gamma )\textbf{u}= \textbf{f}. \end{aligned}$$

Crucially, observe that

$$\begin{aligned} \textbf{s}_{\textbf{L}}[\mathcal {H}] = \textbf{s}_{\mathbb {L}}[\mathcal {H}] -2\kappa _{\mathcal {H}}. \end{aligned}$$

Since $\Omega \subset \left\{ \sigma \in \mathbb {C}:\Im \sigma > \frac{1}{2}\max _{\mathcal {H}=\mathcal {H}^+, \overline{\mathcal {H}}^+}\left( \textbf{s}_{\textbf{L}}[\mathcal {H}] - \left( 2k - \frac{7}{2} \right) \kappa _{\mathcal {H}} \right) \right\}$ and $\textbf{f}\in \underline{H}^{k-2}(\Sigma )$, we now apply the induction assumption on the commuted equation in (274). This yields that for sufficiently large $\gamma$, there exists a unique solution $\textbf{u}= (u, u_i)$ to (274), and moreover, that $\textbf{u}\in \underline{H}^{k-1}(\Sigma )$. The second part of Theorem 6.6 then shows that

$$\begin{aligned} (\widehat{\textbf{L}}'(\sigma )-\gamma )\tilde{u}=0,\qquad \tilde{u} = u_i-\widehat{\mathcal {K}}_i(\sigma )u. \end{aligned}$$

Thus, we have that $\tilde{u}$ solves (271). The estimate from the inductive assumption,

$$\begin{aligned} \left\Vert \textbf{u}\right\Vert _{\underline{H}^{k-1}(\Sigma )} \le C \left\Vert \textbf{f}\right\Vert _{\underline{H}^{k-2}(\Sigma )}, \end{aligned}$$

then implies (272). We can then relax the assumption that *f* is smooth to an assumption that $f\in \underline{H}^{k-1}(\Sigma )$.

Now we move on to extending the domain from that in (273) to that in (270). To do so, we reconsider the $k=0$ case. Let us assume that $f\in \underline{H}^{1}(\Sigma )$. Then for sufficiently large $\gamma$, we have already shown that (271) has a $\underline{H}^{2}(\Sigma )$ solution if

$$\begin{aligned} \sigma \in \Omega \subset \left\{ \sigma \in \mathbb {C}:\Im \sigma > \frac{1}{2}\max _{\mathcal {H}=\mathcal {H}^+, \overline{\mathcal {H}}^+}\left( \textbf{s}_{\mathbb {L}}[\mathcal {H}] -\frac{1}{2}\kappa _{\mathcal {H}} \right) \right\} . \end{aligned}$$

Theorem 9.1 then shows that this solution is unique in $\underline{H}^{1}(\Sigma )$, and moreover satisfies the estimate

$$\begin{aligned} \left\Vert u\right\Vert _{\underline{H}^{1}(\Sigma )} \le C\left\Vert f\right\Vert _{\underline{L}^2(\Sigma )}. \end{aligned}$$

Then, the fact that $\underline{H}^{1}(\Sigma )$ is dense in $\underline{L}^2(\Sigma )$ allows us to deduce that for any $f\in \underline{L}^2(\Sigma )$, (271) has a unique solution for $s\in \Omega$. We have thus extended the possible range of $\Omega$ to the desired range for $k=1$. Doing this inductively yields the result for arbitrary *k*. $\square$

Using Theorem 9.5, we can prove the following corollary.

##### Corollary 9.6

Let $\mathbb {L}$ denote the gauge-fixed linearized Einstein operator around a sufficiently slowly-rotating Kerr-de Sitter metric. Moreover, let $\Omega$ be a fixed compact connected set such that

$$\begin{aligned} \Omega \subset \left\{ \sigma :\Im \sigma > \frac{1}{2}\max _{\mathcal {H}=\mathcal {H}^+,\overline{\mathcal {H}}^+} \left( \textbf{s}_{\mathbb {L}}[\mathcal {H}] -\left( 2k + \frac{1}{2}\right) \kappa _{\mathcal {H}} \right) \right\} . \end{aligned}$$

and let $u \in \underline{H}^{k}(\Sigma )$ be a weak solution of

275 $$\begin{aligned} \widehat{\mathbb {L}}(\sigma )u = 0, \end{aligned}$$

where $$\sigma \in \Omega $$. Then $$u\in C^{\infty }_0(\Sigma )$$.

##### Proof

We first show that it is possible to prove that $u\in \underline{H}^{k+1}(\Sigma )$ from the fact that $u\in \underline{H}^{k}(\Sigma )$. Afterwards, one can iteratively prove that $u\in \underline{H}^{k+s}(\Sigma )$ for any *s* to prove the result.

First observe that if $\widehat{\mathbb {L}}(\sigma )u = 0$, then we trivially have that

$$\begin{aligned} \left( \widehat{\mathbb {L}}(\sigma )-\gamma \right) u = -\gamma u \end{aligned}$$

for any $\gamma \in \mathbb {R}$. Moreover, from Theorem 6.6, we have that there exists some $\gamma$ sufficiently large so that

$$\begin{aligned} \widehat{\mathbb {L}}(\sigma )-\gamma : \underline{H}^{k+1}(\Sigma )\rightarrow \underline{H}^{k}(\Sigma ) \end{aligned}$$

is invertible, and in particular, so that

$$\begin{aligned} \left( \widehat{\mathbb {L}}(\sigma )-\gamma \right) ^{-1}: \underline{H}^{k}(\Sigma )\rightarrow \underline{H}^{k+1}(\Sigma ) \end{aligned}$$

is well-defined.

Thus, writing that

$$\begin{aligned} u = \frac{1}{\gamma }\left( \widehat{\mathbb {L}}(\sigma )-\gamma \right) ^{-1}u, \end{aligned}$$

we find that $u\in \underline{H}^{k+1}(\Sigma )$. Moreover, using Theorem 6.6 again, we find that

$$\begin{aligned} \left\Vert u\right\Vert _{\underline{H}^{k+1}(\Sigma )} \lesssim \frac{1}{\gamma }\left\Vert u\right\Vert _{\underline{H}^{k}(\Sigma )}. \end{aligned}$$

As mentioned earlier, iterating this result then yields the desired conclusion. $\square$

We can now prove a Fredholm alternative for $\widehat{\mathbb {L}}(\sigma )$.

##### Theorem 9.7

For *g* a sufficiently slowly-rotating Kerr-de Sitter metric, let $\mathbb {L}$ be the gauge-fixed linearized Einstein operator on *g*. Then for any $k\in \mathbb {N}$, and $\sigma \in \mathbb {C}$ such that

$$\begin{aligned} \Im \sigma >\frac{1}{2}\max _{\mathcal {H}=\mathcal {H}^+, \overline{\mathcal {H}}^+}\left( \textbf{s}_{\mathbb {L}}[\mathcal {H}] -\left( 2k+\frac{1}{2}\right) \kappa _{\mathcal {H}}\right) , \end{aligned}$$

one of the following holds: either

1. $\widehat{\mathbb {L}}(\sigma )^{-1}$ exists as a bounded map from $\underline{H}^{k-1}(\Sigma )$ to $D^{k}(\widehat{\mathbb {L}}(\sigma ))$, or
2. there exists a finite-dimensional family of solutions to $\widehat{\mathbb {L}}(\sigma )u=0$.

Moreover, the latter occurs only when $\sigma \in \Lambda _{\operatorname {QNF}}^{k}(\mathbb {L})$, where $\Lambda _{\operatorname {QNF}}^{k}(\mathbb {L})$ is a discrete set of points with no accumulation point except at infinity, satisfying that

$$\begin{aligned} \Lambda _{\operatorname {QNF}}^{k}(\mathbb {L})\subset \Lambda _{\operatorname {QNF}}^{k+1}(\mathbb {L}), \end{aligned}$$

and in addition, $\widehat{\mathbb {L}}(\sigma )^{-1}$ is meromorphic in the half-plane

$$\begin{aligned} \Im \sigma >\frac{1}{2}\max _{\mathcal {H}=\mathcal {H}^+, \overline{\mathcal {H}}^+}\left( \textbf{s}_{\mathbb {L}}[\mathcal {H}] -\left( 2k+\frac{1}{2}\right) \kappa _{\mathcal {H}}\right) , \end{aligned}$$

with poles in $\Lambda _{\operatorname {QNF}}^{k}$ where the residues are finite rank operators.

##### Proof

Let $\Omega$ be a fixed compact connected set such that

$$\begin{aligned} \Omega \subset \left\{ \sigma :\Im \sigma > \frac{1}{2}\max _{\mathcal {H}=\mathcal {H}^+,\overline{\mathcal {H}}^+} \left( \textbf{s}_{\mathbb {L}}[\mathcal {H}] -\left( 2k + \frac{1}{2}\right) \kappa _{\mathcal {H}} \right) \right\} . \end{aligned}$$

Recall that from Theorem 9.5, we have shown that there exists a $\lambda$ sufficiently large such that

$$\begin{aligned} (\widehat{\mathbb {L}}(\sigma )-\lambda )^{-1}: \underline{H}^{k}(\Sigma )\rightarrow D^k(\widehat{\mathbb {L}}(\sigma ))\subset \underline{H}^{k+1}(\Sigma ) \end{aligned}$$

is a well-defined, bounded operator for all $\sigma \in \Omega$. Then for the same $\lambda$, we can define the operator $A(\sigma ):\underline{H}^{k}(\Sigma )\rightarrow \underline{H}^{k+1}(\Sigma )$ by

$$\begin{aligned} A(\sigma ){:=}-\lambda (\widehat{\mathbb {L}}(\sigma )-\lambda )^{-1}, \end{aligned}$$

exists as a bounded operator on the entirety of $\Omega$. We will prove the main theorem by using the analytic Fredholm theorem on $A(\sigma )$. We first verify the conditions for the application of the analytic Fredholm theorem (Theorem A.1):

1. As previously mentioned, using Theorem 9.5, there exists some $\lambda$ such that $A(\sigma )$ is a bounded operator on the entirety of $\Omega$.
2. Also by Theorem 9.5, we know that for $\sigma \in \Omega$, $A(\sigma )$ maps $\underline{H}^{k}(\Sigma )\mapsto \underline{H}^{k+1}(\Sigma )$. Thus, by Rellich-Kondrachov, $A(\sigma )$ is a compact operator.
3. $A(\sigma )$ is analytic on $\Omega$, and we can calculate directly that
  $$\begin{aligned} \lim _{\sigma \rightarrow \sigma _0} \frac{A(\sigma )-A(\sigma _0)}{\sigma -\sigma _0} = -\lambda (\widehat{\mathbb {L}}(\sigma _0)-\lambda )^{-1} \left( P_1 + 2\sigma _0A^{-1}\right) (\widehat{\mathbb {L}}(\sigma _0)-\lambda )^{-1}, \end{aligned}$$
  which is a bounded operator on $\underline{H}^{k}(\Sigma )$, where we define $P_1$ by writing
  $$\begin{aligned} \mathbb {L}_{g_b} = A^{-1}D_{t_*}^2 + P_1D_{t_*} + P_2. \end{aligned}$$

We have thus confirmed that $A(\sigma )$ verifies the conditions to apply the analytic Fredholm theorem. We now observe that

$$\begin{aligned} \widehat{\mathbb {L}}(\sigma )u=f \quad \iff \quad (1-A(\sigma ))u = (\widehat{\mathbb {L}}(\sigma )-\lambda )^{-1}f, \end{aligned}$$

and that moreover, by Proposition 4.3, $\widehat{\mathbb {L}}(\sigma )$ always exists for some $\sigma \in \Omega$ after potentially extending $\Omega$. We can now apply the analytic Fredholm theorem to draw the conclusion that either

1. $\widehat{\mathbb {L}}(\sigma )^{-1}$ exists as a bounded map from $\underline{H}^{k}(\Sigma )$ to $\underline{H}^{k+1}(\Sigma )$, or
2. There exists a finite-dimensional family of solutions to $\widehat{\mathbb {L}}(\sigma )u=0$.

From Corollary 9.6, we see that solutions of $\widehat{\mathbb {L}}(\sigma )u=0$ are smooth. Thus, $\Lambda _{\operatorname {QNF}}^{k}(\mathbb {L})\subset \Lambda _{\operatorname {QNF}}^{k+1}(\mathbb {L})$. Finally, $1-A(\sigma )$ is a finite-dimensional perturbation of the identity, and thus is Fredholm of index 0. Consequently, the dimension of the kernel and the co-kernel agree. $\square$

### Identifying the Spectral Gap (Proof of Theorem 5.4)

As was the case for the proof of Theorem 5.3, we prove Theorem 5.4 by analyzing $\widehat{\mathbb {L}}(\sigma )$ in place of $\mathcal {A}- \sigma$. Recall from Lemma 4.6 that $(\mathcal {A}- \sigma )^{-1}(\Sigma ):\textbf{H}^{k}(\Sigma ) \rightarrow D^k(\mathcal {A})$ exists and is a bounded linear transformation if and only if $\widehat{\mathbb {L}}(\sigma )^{-1}: \underline{H}^{k-1}(\Sigma ) \rightarrow D^k(\widehat{\mathbb {L}}(\sigma ))$ exists and is a bounded linear transformation. But from the Morawetz estimate and the corresponding resolvent estimates in Theorem 8.1, we know that this is the case for $\Im \sigma \ge -{\boldsymbol{\alpha }}, \left\lvert \sigma \right\rvert >C_0$, as desired.

#### Remark 40

We briefly remark that what we have essentially shown in this section, is that Assumption 4.8 could instead be reduced to the following two assumptions.

1. $L$ is a strongly hyperbolic linear operator on a slowly-rotating Kerr-de Sitter background, $g_b$.
2. For any $\varepsilon >0$, there exists an elliptic stationary zero-order pseudo-differential operator $Q$ such that
  $$\begin{aligned} {\frac{1}{\left\lvert \eta \right\rvert }\sigma _1\left( QLQ^- - (QLQ^-)^* \right) }\Bigg \vert _{{\Gamma }_{b}} <\varepsilon \operatorname {Id}, \end{aligned}$$
  where $Q^-$ denotes the parametrix of $Q$, and $\sigma _1(P)$ denotes the principal symbol of *P*, and ${\Gamma }_b$ denotes the trapped set of the Kerr-de Sitter metric $g_b$ (recall the definitions in Sect. 7).

## Mode Stability

Having shown that $\mathbb {L}_{g_b}$ satisfies Theorem 5.3 and Theorem 5.4, the only remaining obstacle to exponential decay are the finitely many residual non-decaying resonances. To this end, we show that these resonances are non-physical. At the level of the gauge-fixed linearized Einstein equation, this can mean one of two things.

1. First, it is possible that the quasinormal mode solution does not itself satisfy the linearized constraint equations. As such, it is not a valid solution to the linearized Einstein vacuum equations.
2. Second, even if the quasinormal mode solution does satisfy the linearized constraint equations, if the quasinormal mode in question is an infinitesimal diffeomorphism of the zero solution, it is not truly a distinct solution.

Our goal then will be to show that all of the non-decaying $\textbf{H}^{k}$-quasinormal mode solutions fall into one of these two categories, and are thus unphysical.

### Geometric Mode Stability of Schwarzschild-de Sitter

We begin by considering the case of the linearized non-gauge-fixed Einstein’s equations linearized around a fixed Schwarzschild-de Sitter background, $g_{b_0}$. On a fixed Schwarzschild-de Sitter background, a strong geometric mode stability statement (GMS) exists, having first been proven by Kodama and Ishibashi, [42], but presented below in the slightly modified form derived by Hintz and Vasy [31].

#### Theorem 10.1

(Geometric mode stability (GMS). Referred to as Ungauged Einstein Mode Stability (UEMS) in [31]) Let $b_0$ be the black hole parameters of a Schwarzschild-de Sitter black hole. Then,

1. Let $\sigma \in \mathbb {C}, \Im \sigma \ge 0$, $\sigma \ne 0$ and suppose that ${h}(t_*, x)=e^{-\mathbbm {i}\sigma t_*}u(x)$, $u\in C^\infty (\Sigma )$ is a mode solution of the linearized Einstein equation
  $$\begin{aligned} D_{g_{b_0}}({\text {Ric}}- \Lambda )({h}) = 0. \end{aligned}$$
  Then there exists a 1-form $\omega (t_*, x)=e^{-\mathbbm {i}\sigma t_*}\omega _\sigma (x)$ with $\omega _\sigma \in C^\infty (\Sigma )$ such that
  $$\begin{aligned} {h} =\nabla _{g_{b_0}}\otimes \omega . \end{aligned}$$
2. For all $k\in \mathbb {N}$, and all generalized mode solutions
  $$\begin{aligned} {h}(t_*, x) = \sum _{j=0}^k t_*^j u_j(x),\quad u_j\in C^\infty (\Sigma ),\quad 0\le j\le k, \end{aligned}$$
  of the linearized Einstein equation, there exist $b'\in T_{b_0}B$ and $\omega \in C^\infty (M)$, such that
  $$\begin{aligned} {h} = g'_{b_0}(b') +\nabla _{g_{b_0}}\otimes \omega . \end{aligned}$$

GMS states that any mode solution to the non-gaugefixed linearized vacuum Einstein equations (linearized around a fixed Schwarzschild-de Sitter background) that is non-decaying is a pure gauge solution, arising either from an infinitesimal diffeomorphism ${h} = \mathcal {L}_{\omega ^\sharp } g_{b_0}$, or a linearized Kerr-de Sitter metric (or a combination of the two). In particular, being a mode stability statement at the level of the non-gauge-fixed system of equations, GMS also implies a mode stability statement at the level of the gauge-fixed equations. That is, applying the linearized Bianchi equation, we immediately have the following corollary:

#### Corollary 10.2

Let $b_0$ be the black hole parameters of a Schwarzschild-de Sitter black hole. Let $\sigma \in \mathbb {C}, \Im \sigma \ge 0$, $\sigma \ne 0$ and suppose that ${h}(t_*, x)=e^{-\mathbbm {i}\sigma t_*}u(x)$, $u\in C^\infty (\Sigma )$ is a mode solution of the gauge-fixed linearized Einstein operator,

276 $$\begin{aligned} \mathbb {L}_{g_{b_0}}{h}=0. \end{aligned}$$

Then one of the following must be true:

1. either $${h} = \nabla _{g_{b_0}} \otimes \omega $$, or
2. $\psi {:=} \mathcal {C}_{g_{b_0}}({h}) = e^{-\mathbbm {i}\sigma t_*}v(x)$ is a non-zero mode solution to the constraint propagation equation
  $$\begin{aligned} \Box ^{CP}_{g_{b_0}}\psi = (\Box ^{(1)}_{g_{b_{0}}}-\Lambda )\psi = 0. \end{aligned}$$

A similar result holds for the quasinormal mode at 0. Indeed, let

$$\begin{aligned} {h}(t_*,x) = \sum _{j=0}^k t_*^ju_{jk}(x),\quad u_{jk}\in C^\infty (\Sigma ),\quad 0\le j\le k, \end{aligned}$$

be a generalized mode solution of the gauge-fixed linearized Einstein equation (276). Then, one of the following must hold true:

1. there exist $b'\in T_{b_0}B$ and $\omega \in C^\infty (T^*\mathcal {M})$, such that
  $$\begin{aligned} {h} = g'_{b_0}(b') +\nabla _{g_{b_0}}\otimes \omega , \end{aligned}$$
  and moreover, *h* satisfies the linearized gauge constraint
  $$\begin{aligned} \mathcal {C}_{g_{b_0}}{h} = 0 \end{aligned}$$
  uniformly in $$\mathcal {M}$$;
2. $\psi {:=} \mathcal {C}_{g_{b_0}}({h}) = \sum _{j=0}^k t_*^jv_j(x)$ is a non-zero mode solution to the constraint propagation equation
  $$\begin{aligned} \Box ^{CP}_{g_{b_0}}\psi = (\Box ^{(1)}_{g_{b_{0}}}-\Lambda ) \psi = 0. \end{aligned}$$

#### Proof

Recall from Sect. 3.2 that a solution *h* to the gauge-fixed linearized Einstein equation $\mathbb {L}_{g_{b_0}}{h} = 0$ also satisfies

$$\begin{aligned} \Box ^{CP}_{g_{b_0}}\mathcal {C}_{g_{b_0}}({h}) = 0. \end{aligned}$$

Thus, either $\mathcal {C}_{g_{b_0}}({h})$ is a non-zero mode solution to the constraint propagation equation

$$\begin{aligned} \Box ^{CP}_{g_{b_0}}\psi = 0, \end{aligned}$$

or $\mathcal {C}_{g_{b_0}}({h})=0$. But if $\mathcal {C}_{g_{b_0}}({h})=0$, then *h* must actually be a mode solution of the non-gauge-fixed linearized Einstein equation. An application of Theorem 10.1 allows us to conclude. $\square$

We see from Theorem 10.1 and Corollary 10.2 that there are two types of unphysical modes: those that violate the constraint conditions, and those that are infinitesimal diffeomorphisms of a nearby linearized Kerr-de Sitter metric. This leads us to define the following two categories of unphysical $\textbf{H}^{k}$-quasinormal mode solutions.

#### Definition 39

Let ${h}=e^{-\mathbbm {i}\sigma t_*}u$ be an $\textbf{H}^{k}$-quasinormal mode solution $\mathbb {L}_{g_b}{h}=0$. Then we say that *h* is a $\textbf{H}^{k}$-*geometric quasinormal mode solution* for $\mathbb {L}_{g_b}$ if there exists a linearized Kerr-de Sitter metric $g_b'(b')$ and a one-form $\omega$ such that

$$\begin{aligned} {h} = g_{b}'(b') + \nabla _{g_{b}}\otimes \omega , \end{aligned}$$

and moreover, *h* satisfies the linearized gauge condition

$$\begin{aligned} \mathcal {C}_{g_{b}}({h}) = 0 \end{aligned}$$

uniformly on $\mathcal {M}$. On the other hand, let us call any quasinormal mode solution *h* such that

$$\begin{aligned} \mathcal {C}_{g_b}({h}) \ne 0 \end{aligned}$$

a $\textbf{H}^{k}$-*constraint quasinormal mode solution* for $\mathbb {L}_{g_b}$.

#### Definition 40

For any $\sigma \in \mathbb {C}$, if there exists an $\textbf{H}^{k}$-geometric quasinormal mode solution for $\mathbb {L}_{g_b}$ of the form $e^{-\mathbbm {i}\sigma t_*}u$, then we call $\sigma$ a $\textbf{H}^{k}$-*geometric quasinormal frequency* of $\mathbb {L}_{g_b}$. Similarly, if for $\sigma \in \mathbb {C}$, there exists an $\textbf{H}^{k}$-constraint quasinormal mode solution $e^{-\mathbbm {i}\sigma t_*}u$ of $\mathbb {L}_{g_b}$, then we call $\sigma$ a $\textbf{H}^{k}$-*constraint quasinormal frequency* of $\mathbb {L}_{g_b}$. Given an open subset $\Xi \subset \mathbb {C}$ that contains only finitely many $\textbf{H}^{k}$-quasinormal frequencies of $\mathbb {L}_{g_b}$, we denote by $\Lambda _{\operatorname {QNF}, \Upsilon }^{k}(\mathbb {L}_{g_b}, \Xi )$ and $\Lambda _{\operatorname {QNF}, \mathcal {C}}^{k}(\mathbb {L}_{g_b}, \Xi )$ the set of $\textbf{H}^{k}$-geometric quasinormal frequencies and $\textbf{H}^{k}$-constraint quasinormal frequencies respectively in $\Xi$.

#### Definition 41

Given an open subset $\Xi \subset \mathbb {C}$ that contains only finitely many $\textbf{H}^{k}$-quasinormal frequencies of $\mathbb {L}_{g_b}$, we denote by $\Lambda _{\operatorname {QNM}, \Upsilon }^{k}(\mathbb {L}_{g_b}, \Xi )$ the set of all $\textbf{H}^{k}$-geometric quasinormal mode solutions with frequency $\sigma \in \Xi$, and $\Lambda _{\operatorname {QNM}, \mathcal {C}}^{k}(\mathbb {L}_{g_b}, \Xi )$ the set of all $\textbf{H}^{k}$-constraint quasinormal mode solutions with frequency $\sigma \in \Xi$.

#### Remark 41

Note that $\sigma$ can be both a geometric and a constraint quasinormal frequency for $\mathbb {L}_{g_b}$ and *a priori* it is not clear that all $\textbf{H}^{k}$-quasinormal frequencies of $\mathbb {L}_{g_b}$ are either geometric or constraint frequencies.

The nomenclature reflects that $\textbf{H}^{k}$-geometric quasinormal mode solutions will be handled via a gauge choice, while $\textbf{H}^{k}$-constraint quasinormal mode solutions violate the constraint conditions and thus both geometric and constraint quasinormal modes represent unphysical quasinormal mode solutions to the gauge-fixed linearized Einstein equations.

With this new nomenclature, we see that Corollary 10.2 is a precise statement that in the case $g_b=g_{b_0}$ is a fixed Schwarzschild-de Sitter background, any non-decaying $\textbf{H}^{k}$-quasinormal mode solution ${h} = e^{-\mathbbm {i}\sigma t_*}u$ of $\mathbb {L}_{g_{b_0}}$ are either an $\textbf{H}^{k}$-geometric quasinormal mode or an $\textbf{H}^{k}$-constraint quasinormal mode solution of $\mathbb {L}_{g_{b_0}}$, and therefore, all non-decaying $\textbf{H}^{k}$-quasinormal mode solutions of $\mathbb {L}_{g_{b_0}}$ are unphysical.

### Linearized Stability of Schwarzschild-de Sitter

Given the GMS statement in Theorem 10.1 and the gauge-fixed mode stability statement in Corollary 10.2 for Schwarzschild-de Sitter, we can now prove the linearized stability statement for the linearized Einstein’s equations around $g_{b_0}$.

#### Theorem 10.3

(Stability of the linearized gauge-fixed Einstein vacuum equations linearized around $g_{b_0}$) Fix $k>k_0$, and let $(\underline{g}', k')\in H^{k+1}(\Sigma _0, S^2T^*\Sigma _0)\oplus H^{k}(\Sigma _0;S^2T^*\Sigma _0)$ be solutions of the linearized constraint equations, linearized around the initial data $(\underline{g}_{b_0}, k_{b_0})$ of the Schwarzschild-de Sitter space $(\mathcal {M}, g_{b_0})$. Let *h* be a solution to the initial value problem

$$\begin{aligned} {\left\{ \begin{array}{ll} \mathbb {L}_{g_{b_0}}{h} = 0 & {\text {in }}\mathcal {M},\\ \gamma _0({h}) = D_{(\underline{g}_{b_0}, k_{b_0})}i_{b_0, \operatorname {Id}}(\underline{g}', k')& {\text {on }}\Sigma _0, \end{array}\right. } \end{aligned}$$

where $i_{b, \operatorname {Id}}$ is defined in Proposition 3.6.

Then, there exist $b'\in T_{b_0}B$ and a 1-form $\omega \in C^\infty (\mathcal {M}, T^*\mathcal {M})$ such that

$$\begin{aligned} h= g_{b_0}'(b') +\nabla _{g_{b_0}}\otimes \omega +\tilde{h}, \end{aligned}$$

where $\tilde{h}$ satisfies the pointwise bounds

$$\begin{aligned} \begin{aligned} \left\Vert \tilde{h}\right\Vert _{\overline{H}^{k}(\Sigma _{t_*})}&\lesssim e^{-{\boldsymbol{\alpha }}t_*}\left( \left\Vert \underline{g}'\right\Vert _{H^{k+1}(\Sigma _0)} + \left\Vert k'\right\Vert _{H^{k}(\Sigma _0)}\right) ,\\ \left\lvert b'\right\rvert + e^{-\textbf{M}t_*}\left\Vert \omega \right\Vert _{\overline{H}^{k}(\Sigma _{t_*})}&\lesssim \left( \left\Vert \underline{g}'\right\Vert _{H^{k+1}(\Sigma _0)} + \left\Vert k'\right\Vert _{H^{k}(\Sigma _0)}\right) . \end{aligned} \end{aligned}$$

#### Proof

Let $\Xi = \Lambda _{\operatorname {QNF}}^{k}(\mathbb {L}_{g_{b_0}}, \mathbb {H}^+)$. At the cost of reducing ${\boldsymbol{\alpha }}$, we can ensure that in fact, $\Xi$ consists of all $\textbf{H}^{k}$-quasinormal frequencies such that $\Im \sigma >-{\boldsymbol{\alpha }}$. We index $\Xi = \{\sigma _{j}\}, 1\le j \le N_{\mathbb {L}_{g_{b_0}}}$. Applying Corollary 5.5, we see that *h* can be written as

277 $$\begin{aligned} {h} = \tilde{{h}} + \sum _{1\le j \le N_{\mathbb {L}_{g_{b_0}}}}\sum _{\ell = 1}^{d_j}\sum _{k=0}^{n_{j\ell }} e^{-\mathbbm {i}\sigma _jt_*}t_*^ku_{j\ell }, \end{aligned}$$

where each $\sum _{\ell = 1}^{d_j}\sum _{k=0}^{n_{j\ell }} e^{-\mathbbm {i}\sigma _jt_*}t_*^ku_{j\ell }\in \Lambda _{\operatorname {QNM}}^{k}(\mathbb {L}_{g_{b_0}}, \sigma _j)$, and moreover,

$$\begin{aligned} \begin{aligned} \left\Vert \tilde{{h}}\right\Vert _{\overline{H}^{k}(\Sigma _{t_*})}&\lesssim e^{-{\boldsymbol{\alpha }}t_*}\left( \left\Vert \underline{g}'\right\Vert _{H^{k+1}(\Sigma _0)} + \left\Vert k'\right\Vert _{H^{k}(\Sigma _0)}\right) ,\\ \left\Vert \sum _{\ell = 1}^{d_j}\sum _{k=0}^{n_{j\ell }} e^{-\mathbbm {i}\sigma _jt_*}t_*^ku_{j\ell }\right\Vert _{\overline{H}^{k}(\Sigma _{t_*})}&\lesssim e^{\textbf{M}t_*}\left( \left\Vert \underline{g}'\right\Vert _{H^{k+1}(\Sigma _0)} + \left\Vert k'\right\Vert _{H^{k}(\Sigma _0)}\right) . \end{aligned} \end{aligned}$$

Recall from the construction of the map $i_{b, \operatorname {Id}}$ in Proposition 3.6 that for *h* inducing on $\Sigma _0$ the initial data $i_{b,\operatorname {Id}}(\underline{g}', k') = \gamma _0(h)$, *h* satisfies the initial gauge constraint

$$\begin{aligned} \mathcal {C}_{g_{b_0}}{h}\vert _{\Sigma _0} =\mathcal {L}_{\textbf{T}} \mathcal {C}_{g_{b_0}}{h}\vert _{\Sigma _0} = 0 . \end{aligned}$$

Then, recalling from the discussion in Sect. 3, $\mathcal {C}_{g_{b_0}}{h}$ itself satisfies a wave equation, and as a result, the initial data $(\underline{g}', k')$ launches a solution *h* to the gauge-fixed linearized EVE such that

$$\begin{aligned} \mathcal {C}_{g_{b_0}}({h})=0 \end{aligned}$$

for all time, and thus, *h* solves not only the gauge-fixed linearized EVE, but also the non-gauge-fixed linearized EVE, $D_{g_{b_0}}({\text {Ric}}-\Lambda ){h} = 0$. This implies that the $\textbf{H}^{k}$-quasinormal mode solutions in the finite sum in equation (277) also satisfy the linearized constraint equations

$$\begin{aligned} \mathcal {C}_{g_{b_0}}\left( \sum _{1\le j \le N_{\mathbb {L}_{g_{b_0}}}}\sum _{\ell = 1}^{d_j}\sum _{k=0}^{n_{j\ell }} e^{-\mathbbm {i}\sigma _jt_*}t_*^ku_{j\ell }\right) =0. \end{aligned}$$

As a result, using Corollary 10.2, we see that $\sum _{1\le j \le N_{\mathbb {L}_{g_{b_0}}}}\sum _{\ell = 1}^{d_j}\sum _{k=0}^{n_{j\ell }} e^{-\mathbbm {i}\sigma _jt_*}t_*^ku_{j\ell }$ must in fact be a linear combination of $\textbf{H}^{k}$-geometric quasinormal mode solutions, and thus there exists some $b'$, $\omega$ such that

$$\begin{aligned} \sum _{1\le j \le N_{\mathbb {L}_{g_{b_0}}}}\sum _{\ell = 1}^{d_j}\sum _{k=0}^{n_{j\ell }} e^{-\mathbbm {i}\sigma _jt_*}t_*^ku_{j\ell } = g_{b_0}'(b') +\nabla _{g_{b_0}}\otimes \omega , \end{aligned}$$

as desired. $$\square $$

This method of proving stability of the linearized EVE around $g_{b_0}$ relies only on having the GMS statement in Theorem 10.1 and an appropriate high-frequency resolvent estimate like in Theorem 5.4. When trying to generalize the result to $g_b$, $b\in \mathcal {B}$, we see that while we do still have a good high-frequency resolvent estimate in Theorem 5.4, we do not have a version of GMS which holds for the non-gauge-fixed linearized EVE around $g_b$ for $b=(M, \textbf{a})$ where $\textbf{a}\ne 0$. Instead of proving a version of GMS for $g_b$ directly, which is in and of itself a difficult problem, we will take advantage of the fact that we are only considering $b\in \mathcal {B}$, where $\mathcal {B}$ is a small neighborhood of black hole parameters of $b_0$, to prove a mode stability statement for $\mathbb {L}_{g_b}$ perturbatively.

To this end, we consider an alternative proof for the linear stability of the gauge-fixed Einstein equations linearized around $g_{b_0}$ by showing how to use the $\textbf{H}^{k}$-quasinormal modes to generate an appropriate linearized generalized harmonic gauge for the equation, in which the contribution of the non-decaying $\textbf{H}^{k}$-quasinormal modes disappears.

#### Proposition 10.4

For $b'\in T_{b_0}B$, let $\omega _{b_0}^\Upsilon (b')$ denote the solution of the Cauchy problem

$$\begin{aligned} \begin{aligned} {\left\{ \begin{array}{ll} \mathcal {C}_{g_{b_0}}\circ \nabla _{g_{b_0}}\otimes \omega _{b_0}^\Upsilon (b') = -\mathcal {C}_{g_{b_0}}(g_{b_0}'(b')) &  {\text {in }}\mathcal {M}\\ \gamma _0(\omega _{b_0}^\Upsilon (b'))=(0,0)& {\text {on }}\Sigma _0. \end{array}\right. } \end{aligned} \end{aligned}$$

Define

$$\begin{aligned} (g_{b_0}')^\Upsilon (b') {:=} g_{b_0}'(b') +\nabla _{g_{b_0}}\otimes \omega _{b_0}^\Upsilon (b'), \end{aligned}$$

which solves the linearized gauge-fixed Einstein equation $\mathbb {L}_{g_{b_0}}((g_{b_0}')^\Upsilon (b'))=0$. Fix $k>k_0$, and let ${\boldsymbol{\alpha }}>0$ be sufficiently small.

Then there exists finite-dimensional linear subspaces

$$\begin{aligned} \Theta \subset C^\infty _0 (\mathcal {M}, T^*\mathcal {M}), \qquad \Upsilon \subset \textbf{H}^{k}(\Sigma _0) \end{aligned}$$

such that the following holds: for any $(f, {h}_0, {h}_1)\in D^{k+1,{\boldsymbol{\alpha }}}(\Omega )$ there exist unique $b'\in T_{b_0}B$, $\vartheta \in \Theta$, and $\upsilon \in \Upsilon$, such that the solution $\tilde{{h}}$ of the forward problem

$$\begin{aligned} {\left\{ \begin{array}{ll} \mathbb {L}_{g_{b_0}}\tilde{{h}} = f, &  {\text {in }}\mathcal {M},\\ \gamma _0(\tilde{{h}})=({h}_0, {h}_1) - \gamma _0((g_{b_0}')^\Upsilon (b') +\nabla _{g_{b_0}}\otimes \vartheta ) - \upsilon , &  {\text {on }}\Sigma _0 \end{array}\right. } \end{aligned}$$

satisfies the pointwise bound

$$\begin{aligned} \left\Vert \tilde{{h}}\right\Vert _{\overline{H}^{k}(\Sigma _{t_*})} \lesssim e^{-{\boldsymbol{\alpha }}t_*}\left\Vert (f, {h}_0, {h}_1)\right\Vert _{D^{k+1,{\boldsymbol{\alpha }}}(\mathcal {M})}, \end{aligned}$$

and moreover, the map $(f, {h}_0, {h}_1)\mapsto (b', \vartheta , \upsilon )$ is linear and continuous. Finally, if $({h}_0, {h}_1)$ satisfy the linearized gauge conditions, then in fact $\upsilon =0$.

#### Remark 42

The analogous result to Proposition 10.4 in [31] is Theorem 10.1. The first proof we gave at the beginning of the section should be thought of as analogous to the proof of Theorem 10.1 in [31]. In particular, both proofs rely on a geometric mode stability statement (referred to as UEMS in [31]) that is not robust with respect to perturbations of the angular momentum. In [31], constraint damping is used to then show the existence of a stable constraint propagation equation to ensure that all non-decaying modes of the linearized gauge-fixed Einstein operator are pure gauge modes. On the other hand, we have introduced the space $\Lambda _{\operatorname {QNF}, \Upsilon }^{k}(\mathbb {L}_{g_b},\Xi )$ in order to track these modes precisely.

#### Proof

Consider the set of $\textbf{H}^{k}$-quasinormal mode solutions of $\mathbb {L}_{g_{b_0}}$ with non-negative imaginary part $\Lambda _{\operatorname {QNM}}^{k}(\mathbb {L}_{g_{b_0}}, \mathbb {H}^+)$. Recall from Theorem 5.3 and Theorem 5.4 that there are only finitely many such $\textbf{H}^{k}$-quasinormal modes. Let us first recall that all $\textbf{H}^{k}$-geometric quasinormal mode solutions $\psi$ satisfy the linearized gauge condition

$$\begin{aligned} \mathcal {C}_{g_{b_0}}\psi = 0. \end{aligned}$$

Moreover, since all $\textbf{H}^{k}$- quasinormal mode solutions of $\mathbb {L}_{g_{b_0}}$ satisfying the linearized gauge constraint are also mode solutions of the non-gauge-fixed linearized Einstein equation, we know from Theorem 10.1 that all $\textbf{H}^{k}$-quasinormal mode solutions of $\mathbb {L}_{g_{b_0}}$ satisfying the linearized gauge constraint are in fact $\textbf{H}^{k}$-geometric quasinormal modes. On the other hand, since the constraint modes of $\mathbb {L}_{g_{b_0}}$ are exactly those modes of $\mathbb {L}_{g_{b_0}}$ which do not satisfy the linearized gauge condition, all the quasinormal modes of $\mathbb {L}_{g_{b_0}}$ must either be an $\textbf{H}^{k}$-quasinormal geometric mode solution or an $\textbf{H}^{k}$-quasinormal constraint mode solution. We can then characterize the $\textbf{H}^{k}$-geometric quasinormal mode solutions and the $\textbf{H}^{k}$-constraint quasinormal mode solutions by:

$$\begin{aligned} \begin{aligned} \Lambda _{\operatorname {QNM}, \Upsilon }^{k}(\mathbb {L}_{g_{b_0}},\mathbb {H}^+)&= \left\{ {h}\in \Lambda _{\operatorname {QNM}}^{k}(\mathbb {L}_{g_{b_0}}, \mathbb {H}^+):\mathcal {C}_{g_{b_0}}({h}) = 0\right\} ,\\ \Lambda _{\operatorname {QNM}, \mathcal {C}}^{k}(\mathbb {L}_{g_{b_0}},\mathbb {H}^+)&= \left\{ {h}\in \Lambda _{\operatorname {QNM}}^{k}(\mathbb {L}_{g_{b_0}}, \mathbb {H}^+):\mathcal {C}_{g_{b_0}}({h}) \ne 0\right\} . \end{aligned} \end{aligned}$$

Since all $\textbf{H}^{k}$-quasinormal mode solutions are either $\textbf{H}^{k}$-geometric quasinormal solutions or $\textbf{H}^{k}$-constraint quasinormal mode solutions, and linear combination of $\textbf{H}^{k}$-quasinormal mode solutions are $\textbf{H}^{k}$-quasinormal mode solutions, it is clear from the preceding discussion that

$$\begin{aligned} \Lambda _{\operatorname {QNM}}^{k}(\mathbb {L}_{g_{b_0}},\mathbb {H}^+) = \Lambda _{\operatorname {QNM}, \Upsilon }^{k}(\mathbb {L}_{g_{b_0}}, \mathbb {H}^+) + \Lambda _{\operatorname {QNM}, \mathcal {C}}^{k}(\mathbb {L}_{g_{b_0}}, \mathbb {H}^+). \end{aligned}$$

We remark that $\Lambda _{\operatorname {QNM}, \mathcal {C}}^{k}$ is not itself a vector space and in general, the sum of a $\textbf{H}^{k}$-constraint quasinormal mode solution and a $\textbf{H}^{k}$-geometric quasinormal mode solution is a $\textbf{H}^{k}$-constraint quasinormal mode solution. We index the $\textbf{H}^{k}$-geometric quasinormal frequencies, $\left\{ \sigma _{j}^\Upsilon \right\} _{j=1}^{N_{\Upsilon }}$ such that $\sigma _1=0$. For each $\sigma _j^\Upsilon$, $j\ge 2$, we fix a basis $\left\{ \nabla _{g_{b_0}}\otimes \vartheta _{j\ell }\right\} _{\ell =1}^{N^\Upsilon _j}$ of $\Lambda _{\operatorname {QNM}, \Upsilon }^{k}(\mathbb {L}_{g_{b_0}}, \sigma _j^\Upsilon )$, and define

$$\begin{aligned} \Theta _{\ne 0}{:=} {\text {Span}}\,\left\{ \vartheta _{j\ell }:2\le j\le N_{b_0}^\Upsilon , 1\le \ell \le N^{\Upsilon }_j\right\} . \end{aligned}$$

Next, index the constraint $\textbf{H}^{k}$-quasinormal frequencies $\{\sigma _j^\mathcal {C}\}_{i=1}^{N_{\mathcal {C}}}$. Then for each $\sigma _j^{\mathcal {C}}$, we fix a basis $\left\{ \upsilon _{j\ell }\right\} _{\ell =1}^{N^{\mathcal {C}}_j}$, and define

$$\begin{aligned} \Upsilon {:=}{\text {Span}}\,\left\{ \upsilon _{j\ell }: 1\le j\le N^{\mathcal {C}}_{b_0}, 1\le \ell \le N_j^{\mathcal {C}}\right\} . \end{aligned}$$

It remains to deal with the geometric $\textbf{H}^{k}$-quasinormal mode solutions at 0. At the zero quasinormal mode, we need to separate the gauge modes coming from a linearized Kerr-de Sitter metric and the remaining modes arising from an infinitesimal diffeomorphism.

To do so, recall that we defined $\omega _{b_0}^\Upsilon (b')$ as the solution of a Cauchy problem for

$$\begin{aligned} \Box ^{\Upsilon }_{g_{b_0}} = \mathcal {C}_{g_{b_0}}\circ \nabla _{g_{b_0}}\otimes . \end{aligned}$$

We can calculate using the definition of $\mathcal {C},\nabla _{g_{b_0}}\otimes$, that $\Box ^{\Upsilon }_{g_{b_0}}$ is principally $\Box _{g_{b_{0}}}$ and is equal to $\Box ^{(1)}_{g_{b_{0}}}$ up to an order-zero perturbation^25^. As a result, $\Box ^{\Upsilon }_{g_{b_0}}$ is a strongly hyperbolic operator with a well-defined $\textbf{H}^{k}$-quasinormal spectrum just like $\mathbb {L}_{g_{b_0}}$. In fact, from Lemma 7.17, we see that it also has the desired pseudo-differential smallness at ${\Gamma }_{b_0}$ that would allow us to prove the existence of a spectral gap for $\Box ^{\Upsilon }_{g_{b_0}}$. As a result,

$$\begin{aligned} \omega _{b_0}^\Upsilon = \omega _0 + \omega _{-} + \omega _{+}, \end{aligned}$$

where $\omega _{-}$ decays exponentially, $\omega _{+}\in \Lambda _{\operatorname {QNM}}^{k}(\Box ^{\Upsilon }_{g_{b_0}}, \mathbb {H}^+\backslash \{0\})$, and

$\omega _0$ is the contribution of the zero-mode. Then

$$\begin{aligned} (g_{b_0}')^\Upsilon (b')^{(0)} = g_{b_0}'(b') +\nabla _{g_{b_0}}\otimes \omega _0 \end{aligned}$$

satisfies $$\mathbb {L}_{g_{b_0}}((g_{b_0}')^\Upsilon (b')^{(0)}) = 0$$, and $$(g_{b_0}')^\Upsilon (b')^{(0)} \in \Lambda _{\operatorname {QNM}, \Upsilon }^{k}(\mathbb {L}_{g_{b_0}}, 0)$$.

We now define

$$\begin{aligned} K {:=} \left\{ (g_{b_0}')^\Upsilon (b')^{(0)}:b'\in T_{b_0}B\right\} \subset \Lambda _{\operatorname {QNM}, \Upsilon }^{k}(\mathbb {L}_{g_{b_0}}, 0), \end{aligned}$$

which has dimension $d_0= 4$. Then $\Lambda _{\operatorname {QNM}, \Upsilon }^{k}(\mathbb {L}_{g_{b_0}}, 0)\backslash K$ has a basis of the form $\left\{ \nabla _{g_{b_0}}\otimes \vartheta _\ell :1\le \ell \le d_1\right\}$, where $d_1=\dim \Lambda _{\operatorname {QNM}, \Upsilon }^{k}(\mathbb {L}_{g_{b_0}}, 0)-d_0$. Define

$$\begin{aligned} \Theta _0 {:=} {\text {Span}}\,\left\{ \vartheta _\ell :1\le \ell \le d_1\right\} ,\quad \Theta = \Theta _0\oplus \Theta _{\ne 0}. \end{aligned}$$

We then observe that

$$\begin{aligned} \mathbb {L}_{g_{b_0}}\left( (g_{b_0}')^\Upsilon (b')-(g_{b_0}')^\Upsilon (b')^{(0)}\right) = 0. \end{aligned}$$

As a result,

$$\begin{aligned} (g_{b_0}')^\Upsilon (b')-(g_{b_0}')^\Upsilon (b')^{(0)} \in \nabla _{g_{b_0}}\otimes \Theta + O(e^{-{\boldsymbol{\alpha }}t_*}). \end{aligned}$$

We can now define the space of initial value problem modifications,

$$\begin{aligned} \mathcal {Z}{:=} (0, \gamma _0((g_{b_0}')^\Upsilon (T_{b_0}B))) +(0, \gamma _0(\nabla _{g_{b_0}}\otimes \Theta )) + (0, \gamma _0(\Upsilon )) \subset D^{\infty ,{\boldsymbol{\alpha }}}(\mathcal {M}, S^2T^*\mathcal {M}). \end{aligned}$$

The map $\lambda _{\mathcal {Z}}$ as defined in Corollary 4.13 is then bijective by construction. $\lambda _{\mathcal {Z}}$ is surjective since any non-decaying asymptotic behavior in a solution of $\mathbb {L}_{g_{b_0}}{h} = (f,{h}_0,{h}_1)$ can be removed by modifying $(f', {h}_0', {h}_1') = (f,{h}_0,{h}_1) + z$ for some $z\in \mathcal {Z}$. By construction $\lambda _{\mathcal {Z}}$ is also injective as the dimension of $\mathcal {Z}$ is at most as large as the space of $\textbf{H}^{k}$-quasinormal mode solutions $\Lambda _{\operatorname {QNM}}^{k}(\mathbb {L}_{g_{b_0}}, \mathbb {H}^+)$. We can also observe directly that in the case that $({h}_0, {h}_1)$ solve the linearized constraint equation and the linearized harmonic coordinate condition, we in fact have that $\mathcal {C}_{g_{b_0}}({h}) = 0$, and thus, there are no contributions from the $\textbf{H}^{k}$-quasinormal constraint modes, turning the family of initial value problem modifications simply into a family of gauge modifications, as desired. The desired estimates follow from the estimates in Corollary 4.13. $\square$

With Proposition 10.4, we can now offer an alternative proof of Theorem 10.3.

#### Proof

(Alternative proof of Theorem 10.3) Using Proposition 10.4, we know that there exists some $b'\in T_{b_0}B$, $\vartheta \in \Theta$ such that the solution $\tilde{h}$ of the Cauchy problem

$$\begin{aligned} \begin{aligned} \mathbb {L}_{g_{b_0}}{h}&= 0\\ \gamma _0({h})&= i_{b_0, \operatorname {Id}}(\underline{g}', k') - \gamma _0((g_{b_0}')^{\Upsilon }(b') +\nabla _{g_{b_0}}\otimes \vartheta ) \end{aligned} \end{aligned}$$

satisfies the decay bound

$$\begin{aligned} \left\Vert \tilde{h}\right\Vert _{\overline{H}^{k}(\Sigma _{t_*})} \lesssim e^{-{\boldsymbol{\alpha }}t_*}\left( \left\Vert g'\right\Vert _{H^{k+1}(\Sigma _0)} + \left\Vert k'\right\Vert _{H^{k}(\Sigma _0)} \right) . \end{aligned}$$

Moreover, by construction,

$$\begin{aligned} \mathbb {L}_{g_{b_0}}\left( (g_{b_0}')^{\Upsilon }(b') +\nabla _{g_{b_0}}\otimes \vartheta \right) = 0. \end{aligned}$$

As a result,

$$\begin{aligned} h = (g_{b_0}')^{\Upsilon }(b') +\nabla _{g_{b_0}}\otimes \vartheta + \tilde{h} \end{aligned}$$

solves

$$\begin{aligned} \begin{aligned} \mathbb {L}_{g_{b_0}}h&= 0,\\ \gamma _0(h)&= i_{b_0, \operatorname {Id}}(\underline{g}', k'), \end{aligned} \end{aligned}$$

and moreover since $\mathcal {C}_{g_{b_0}}(h) = 0$ uniformly on $\mathcal {M}$, we must have that actually, *h* also solves the non-gauge-fixed linearized Einstein equation and has the desired form in Theorem 10.3. $\square$

### Perturbation of the $\textbf{H}^{k}$-Quasinormal Spectrum to Kerr-de Sitter

We now wish to show that Theorem 10.3 holds not only for $g_{b_0}$, but also for $g_b$ sufficiently slowly-rotating. Unfortunately, in the Kerr-de Sitter case, we do not have a statement of geometric mode stability like we did in the Schwarzschild-de Sitter case. Therefore, as previously mentioned, the direct proof of linear stability of Schwarzschild-de Sitter using Theorem 10.1 and Theorems 5.3 and 5.4 cannot be adjusted to extend to the slowly-rotating Kerr-de Sitter case. What saves us is the alternative method of proving the linear stability of Schwarzschild-de Sitter in Proposition 10.4 which uses only the fact that all non-decaying $\textbf{H}^{k}$-quasinormal modes of $\mathbb {L}_{g_{b_0}}$ are either $\textbf{H}^{k}$-geometric or $\textbf{H}^{k}$-constraint quasinormal modes. The main goal of this section will be to utilize perturbative properties of the spectrum of strongly hyperbolic linear operators to deduce the that all non-decaying $\textbf{H}^{k}$-quasinormal modes of $\mathbb {L}_{g_b}$ are also either geometric or constraint modes. From there, it will follow that we can repeat the proof of Proposition 10.4, and as a result, attain a proof for the linear stability of the gauge-fixed Einstein’s equations linearized around $g_b$.

#### Proposition 10.5

(Mode stability of $\mathbb {L}_{g_b}$, version 2) The set of $\textbf{H}^{k}$-geometric quasinormal frequencies of $\mathbb {L}_{g_b}$, and $\textbf{H}^{k}$-constraint quasinormal frequencies of $\mathbb {L}_{g_b}$ are continuous in *b* in the Hausdorff distance sense, and in particular, for a sufficiently small neighborhood $\mathcal {B}$ of $b_0$,

$$\begin{aligned} \begin{aligned} \dim \Lambda _{\operatorname {QNM}, \Upsilon }^{k}(\mathbb {L}_{g_{b_0}}, \mathbb {H}^+)&= \dim \Lambda _{\operatorname {QNM}, \Upsilon }^{k}(\mathbb {L}_{g_b}, \mathbb {H}^+),\\ \dim \Lambda _{\operatorname {QNM}, \mathcal {C}}^{k}(\mathbb {L}_{g_{b_0}}, \mathbb {H}^+)&= \dim \Lambda _{\operatorname {QNM}, \mathcal {C}}^{k}(\mathbb {L}_{g_b}, \mathbb {H}^+), \end{aligned} \end{aligned}$$

for all $b\in \mathcal {B}$. As a result, for $b\in \mathcal {B}$, all non-decaying $\textbf{H}^{k}$-quasinormal mode solutions of $\mathbb {L}_{g_b}$ are either $\textbf{H}^{k}$-geometric or $\textbf{H}^{k}$-constraint quasinormal mode solutions.

#### Proof

The main difficulty in applying perturbation theory to $\mathbb {L}_{g_b}$ directly is that while $\mathbb {L}_{g_b}$ is strongly hyperbolic, the perturbation theory established in Proposition 4.14 does not distinguish between geometric and constraint modes. Neither does it prevent the introduction or destruction of geometric or constraint modes. The main tool that allows us to circumvent this in the proof of the current proposition is the observation that we can identify the $\textbf{H}^{k}$-geometric quasinormal modes of $\mathbb {L}_{g_b}$ with the $\textbf{H}^{k}$-quasinormal modes of the constraint propagation operator $\Box ^{CP}_{g_{b}}$. Since $\Box ^{CP}_{g_b}$ is a strongly hyperbolic operator, we can apply perturbation theory results to $\Box ^{CP}_{g_b}$, which gives us a perturbative way of treating the $\textbf{H}^{k}$-geometric quasinormal modes of $\mathbb {L}_{g_b}$.

We begin by relating the non-zero $\textbf{H}^{k}$-quasinormal modes of $\mathbb {L}_{g_b}$ with the non-zero $\textbf{H}^{k}$-quasinormal modes of $\Box ^{CP}_{g_b}$. Define

$$\begin{aligned} \mathbb {H}^+_\epsilon {:=} \left\{ \sigma \in \mathbb {C}:\left\lvert \sigma \right\rvert>\epsilon , \Im \sigma > -\epsilon \right\} ,\qquad \mathbb {B}_\epsilon {:=} \left\{ \sigma \in \mathbb {C}: \left\lvert \sigma \right\rvert <\epsilon \right\} , \end{aligned}$$

where $\epsilon$ is chosen so that

$$\begin{aligned} \partial \mathbb {H}^+_{\epsilon }\bigcap \Lambda _{\operatorname {QNM}}^{k}(\mathbb {L}_{g_b}) = \emptyset ,\qquad \mathbb {B}_{\epsilon } \bigcap \Lambda _{\operatorname {QNM}}^{k}(\mathbb {L}_{g_b}) = \{0\}. \end{aligned}$$

For any $\textbf{H}^{k}$-quasinormal mode $\omega = e^{-\mathbbm {i}\sigma t_*}\omega _\sigma$ of $\Box ^{CP}_{g_b}$,Recall that . As a result, for ${h} = \nabla _{g_b}\otimes \omega$, we have that

$$\begin{aligned} \mathbb {L}_{g_b}{h} = 0, \end{aligned}$$

and *h* is a geometric mode of $\mathbb {L}_{g_b}$. Thus, we can consider the mapping

$$\begin{aligned} \begin{aligned} \Lambda _{\operatorname {QNM}}^{k}(\Box ^{CP}_{g_b}, \mathbb {H}^+_\epsilon )&\mapsto \Lambda _{\operatorname {QNM}, \Upsilon }^{k}(\mathbb {L}_{g_b}, \mathbb {H}^+_\epsilon ),\\ \omega&\mapsto \nabla _{g_b}\otimes \omega . \end{aligned} \end{aligned}$$

This map is clearly injective, as if $\omega _1, \omega _2\in \Lambda _{\operatorname {QNM}}^{k}(\Box ^{CP}_{g_b}, \mathbb {H}^+_\epsilon ) \subset C^{\infty }(\mathcal {M}, T^*\mathcal {M})$ satisfy

$$\begin{aligned} \nabla _{g_b}\otimes (\omega _1-\omega _2) = 0, \end{aligned}$$

then $\omega _1-\omega _2$ is Killing, but all the Killing vectors on Kerr-de Sitter are stationary, and we assumed that $\omega _1,\omega _2\in \Lambda _{\operatorname {QNM}}^{k}(\Box ^{CP}_{g_b}, \mathbb {H}^+_\epsilon )$, so in particular, $\omega _1,\omega _2\not \in \Lambda _{\operatorname {QNM}, \Upsilon }^{k}(\Box ^{CP}_{g_b}, 0)$. Now consider some ${h} = \nabla _{g_b}\otimes \omega \in \Lambda _{\operatorname {QNM}, \Upsilon }^{k}(\mathbb {L}_{g_b}, \mathbb {H}^+_\epsilon )$. Then by definition, we have that $\nabla _{g_b}\otimes \omega$ satisfies the linearized gauge constraint, and in fact, $\omega \in \Lambda _{\operatorname {QNM}}^{k}(\Box ^{CP}_{g_b}, \mathbb {H}^+_\epsilon )$. As a result, we can define the injective map

$$\begin{aligned} \begin{aligned} \Lambda _{\operatorname {QNM}, \Upsilon }^{k}(\mathbb {L}_{g_b}, \mathbb {H}^+_\epsilon )&\mapsto \Lambda _{\operatorname {QNM}}^{k}(\Box ^{CP}_{g_b}, \mathbb {H}^+_\epsilon ),\\ \nabla _{g_b}\otimes \omega&\mapsto \omega . \end{aligned} \end{aligned}$$

As a result, we have a bijection between $\Lambda _{\operatorname {QNM}}^{k}(\Box ^{CP}_{g_b}, \mathbb {H}^+_\epsilon )$ and $\Lambda _{\operatorname {QNM}, \Upsilon }^{k}(\mathbb {L}_{g_b}, \mathbb {H}^+_\epsilon )$. This crucially gives a method for counting the non-zero $\textbf{H}^{k}$-geometric quasinormal modes of $\mathbb {L}_{g_b}$, and we have that

278 $$\begin{aligned} \dim \Lambda _{\operatorname {QNM}}^{k}(\Box ^{CP}_{g_b}, \mathbb {H}^+_\epsilon ) = \dim \Lambda _{\operatorname {QNM}, \Upsilon }^{k}(\mathbb {L}_{g_b}, \mathbb {H}^+_\epsilon ). \end{aligned}$$

We now treat the zero-frequency $\textbf{H}^{k}$-geometric quasinormal mode solutions. Observe that for $b = (M, a)$ the mapping

$$\begin{aligned} \begin{aligned} \Lambda _{\operatorname {QNM}}^{k}(\Box ^{CP}_{g_b}, \mathbb {B}_\epsilon )&\rightarrow \Lambda _{\operatorname {QNM}, \Upsilon }^{k}(\mathbb {L}_{g_b}, \mathbb {B}_\epsilon ), \\ \omega&\mapsto \nabla _{g_b}\otimes \omega \end{aligned} \end{aligned}$$

is a linear map with kernel spanned by the Killing vectorfields of $g_b$. Recall that the Killing vectorfields of $g_b$ have basis $\textbf{T}, {\Phi }$ if $b=(M, a), a\ne 0$, and basis $\textbf{T}, \Omega _{ij}$ if $b=(M, 0)$, where $\Omega _{ij}$ are the rotation vectorfields.

Applying the linearized second Bianchi identity, the map

$$\begin{aligned} \begin{aligned}&\left\{ {h} \in \Lambda _{\operatorname {QNM}, \Upsilon }^{k}(\mathbb {L}_{g_b}, \mathbb {B}_\epsilon ): {h} = \nabla _{g_{b}}\otimes \omega , \omega \in C^\infty (\mathcal {M}, T^*\mathcal {M})\right\} \\&\rightarrow \Lambda _{\operatorname {QNM}}^{k}(\Box ^{CP}_{g_b}, \mathbb {B}_\epsilon ) \backslash \left( \operatorname {Ker}(\nabla _{g_b}\otimes )\backslash \textbf{0}\right) ,\\&\nabla _{g_b}\otimes \omega \mapsto \omega , \end{aligned} \end{aligned}$$

is injective. Thus the family of maps

$$\begin{aligned} \begin{aligned}&\Lambda _{\operatorname {QNM}}^{k}(\Box ^{CP}_{g_b}, \mathbb {B}_\epsilon ) \backslash \left( \operatorname {Ker}(\nabla _{g_b}\otimes )\backslash \textbf{0}\right) \\&\rightarrow \left\{ {h} \in \Lambda _{\operatorname {QNM}, \Upsilon }^{k}(\mathbb {L}_{g_b}, \mathbb {B}_\epsilon ): {h} = \nabla _{g_{b}}\otimes \omega , \omega \in C^\infty (\mathcal {M}, T^*\mathcal {M})\right\} , \\&\omega \mapsto \nabla _{g_b}\otimes \omega \end{aligned} \end{aligned}$$

are bijective for all $b\in \mathcal {B}$. Furthermore, defining

$$\begin{aligned} d_b = \dim \frac{g_b'(T_bB)}{(\operatorname {range}\, \nabla _{g_b}\otimes )\bigcap g_b'(T_bB)}, \end{aligned}$$

we have that

$$\begin{aligned} d_b = {\left\{ \begin{array}{ll} 4, &  a = 0,\\ 2, &  a \ne 0. \end{array}\right. } \end{aligned}$$

As a result,

$$\begin{aligned} \dim \Lambda _{\operatorname {QNM}, \Upsilon }^{k}(\mathbb {L}_{g_b}, 0) = \dim \Lambda _{\operatorname {QNM}}^{k}(\Box ^{CP}_{g_b}, 0) - \dim \operatorname {Ker}\, \nabla _{g_b}\otimes + d_b = \dim \Lambda _{\operatorname {QNM}}^{k}(\Box ^{CP}_{g_b}, 0). \end{aligned}$$

We now apply Proposition 4.14 to see that for $b\in \mathcal {B}$, $\mathcal {B}$ sufficiently small,

$$\begin{aligned} \dim \Lambda _{\operatorname {QNM}}^{k}(\Box ^{CP}_{g_{b_0}}, 0) = \dim \Lambda _{\operatorname {QNM}}^{k}(\Box ^{CP}_{g_b}, 0). \end{aligned}$$

As a result, we in fact have that

$$\begin{aligned} \dim \Lambda _{\operatorname {QNM}, \Upsilon }^{k}(\mathbb {L}_{g_b}, 0) = \dim \Lambda _{\operatorname {QNM}, \Upsilon }^{k}(\mathbb {L}_{g_{b_0}}, 0). \end{aligned}$$

Combined with (278), we have that

$$\begin{aligned} \dim \Lambda _{\operatorname {QNM}, \Upsilon }^{k}(\mathbb {L}_{g_b}, \mathbb {H}^+) = \dim \Lambda _{\operatorname {QNM}, \Upsilon }^{k}(\mathbb {L}_{g_{b_0}}, \mathbb {H}^+). \end{aligned}$$

We now move on to consider the constraint modes. To begin, observe that both $\mathcal {C}_{g_b}$ and $\Lambda _{\operatorname {QNM}}^{k}(\mathbb {L}_{g_b}, \mathbb {H}^+)$ are continuous in *b*. Then, we can use the lower-semicontinuity of rank to see that for a sufficiently small neighborhood of black hole parameters $\mathcal {B}\ni b_0$,

$$\begin{aligned} \dim \mathcal {C}_{g_b}\left( \Lambda _{\operatorname {QNM}}^{k}(\mathbb {L}_{g_b},\mathbb {H}^+)\right) \ge \dim \mathcal {C}_{g_{b_0}}\left( \Lambda _{\operatorname {QNM}}^{k}(\mathbb {L}_{g_{b_0}},\mathbb {H}^+)\right) . \end{aligned}$$

We can now conclude that all $\textbf{H}^{k}$-quasinormal mode solutions of $\mathbb {L}_{g_b}$ are either $\textbf{H}^{k}$-geometric or $\textbf{H}^{k}$-constraint quasinormal mode solutions. The continuity statement holds directly by applying Proposition 4.14 to $\Box ^{CP}_{g_b}$ and $\mathbb {L}_{g_b}$. $\square$

Having shown that all $\textbf{H}^{k}$-quasinormal modes of $\mathbb {L}_{g_b}$ are either $\textbf{H}^{k}$-geometric quasinormal modes or $\textbf{H}^{k}$-constraint quasinormal modes, we can now directly repeat the proof of Proposition 10.4 with $g_b$ in place of $g_{b_0}$.

#### Proposition 10.6

Fix $b\in \mathcal {B}$. Then for $b'\in T_{b}B$, let $\omega _{b}^\Upsilon (b')$ denote the solution of the Cauchy problem

$$\begin{aligned} {\left\{ \begin{array}{ll} \left( \mathcal {C}_{g_{b}}\circ \nabla _{g_{b}}\otimes \right) \omega _{b}^\Upsilon (b') = -\mathcal {C}_{g_{b}}(g_{b}'(b')) &  {\text {in }}\mathcal {M},\\ \gamma _0(\omega _{b}^\Upsilon (b'))=(0,0)& {\text {on }}\Sigma _0. \end{array}\right. } \end{aligned}$$

Define

$$\begin{aligned} (g_b')^\Upsilon (b') {:=} g_b'(b') + \nabla _{g_b}\otimes \omega _{b}^\Upsilon (b'), \end{aligned}$$

which solves the linearized gauge-fixed Einstein equation

$$\begin{aligned} \mathbb {L}_{g_b}((g_b')^\Upsilon (b')) = 0. \end{aligned}$$

Now fix some $k>k_0$. Then there exists some small ${\boldsymbol{\alpha }}>0$ such that there exist finite dimensional linear subspaces

$$\begin{aligned} \Theta \subset H^{k,-\textbf{M}}(\mathcal {M}, T^*\mathcal {M}), \qquad \Upsilon \subset \textbf{H}^{k}(\Sigma _0), \end{aligned}$$

where $-\textbf{M}$ is as constructed in 4.11 and represents the maximal exponential rate at which a solution to $\mathbb {L}_{g_b}$ can grow, such that the following holds: for any $(f, h_0, h_1)\in D^{k+1,{\boldsymbol{\alpha }}}(\mathcal {M})$ there exist unique $b'\in T_{b}\mathcal {B}$, $\vartheta \in \Theta$, and $\upsilon \in \Upsilon$, such that the solution of the Cauchy problem

$$\begin{aligned} \begin{aligned} \mathbb {L}_{g_b}\tilde{h}&= f,\\ \gamma _0(\tilde{h})&=(h_0, h_1) - \upsilon - \gamma _0\left( (g_{b}')^\Upsilon (b') + \nabla _{g_b}\otimes \vartheta \right) . \end{aligned} \end{aligned}$$

satisfies $\tilde{h}\in H^{k,{\boldsymbol{\alpha }}}(\mathcal {M})$, the map $(f, h_0, h_1)\mapsto (b', \vartheta , \upsilon )$ is linear and continuous, and $\tilde{h}$ satisfies the pointwise bound

$$\begin{aligned} \left\Vert \tilde{h}\right\Vert _{\overline{H}^{k}(\Sigma _{t_*})} \lesssim e^{-{\boldsymbol{\alpha }}t_*}\left\Vert (f, h_0, h_1)\right\Vert _{D^{k+1,{\boldsymbol{\alpha }}}(\mathcal {M})}. \end{aligned}$$

Finally, if $f=0$, and moreover, $(h_0, h_1)$ satisfy the linearized gauge constraint, then in fact $\upsilon =0$.

#### Proof

From Proposition 10.5, we know that all $\textbf{H}^{k}$-quasinormal modes of $\mathbb {L}_{g_b}$ are either geometric or constraint modes, we see that we can characterize the $\textbf{H}^{k}$-geometric quasinormal modes of $\mathbb {L}_{g_b}$ and $\textbf{H}^{k}$-constraint quasinormal modes of $\mathbb {L}_{g_b}$ by

$$\begin{aligned} \begin{aligned} \Lambda _{\operatorname {QNM}, \Upsilon }^{k}(\mathbb {L}_{g_b}, \mathbb {H}^+)&= \left\{ h\in \Lambda _{\operatorname {QNM}}^{k}(\mathbb {L}_{g_b}, \mathbb {H}^+): \mathcal {C}_b({h})=0\right\} ,\\ \Lambda _{\operatorname {QNM}, \mathcal {C}}^{k}(\mathbb {L}_{g_b}, \mathbb {H}^+)&= \left\{ h \in \Lambda _{\operatorname {QNM}}^{k}(\mathbb {L}_{g_b}, \mathbb {H}^+): \mathcal {C}_b({h})\ne 0\right\} . \end{aligned} \end{aligned}$$

This allows us to effectively repeat the proof of Proposition 10.4, using the $\textbf{H}^{k}$-quasinormal modes of $\mathbb {L}_{g_b}$ in place of those of $\mathbb {L}_{g_{b_0}}$. We construct the span of all gauge choices that correspond to the non-zero $\textbf{H}^{k}$-quasinormal geometric mode solutions,

$$\begin{aligned} \Theta _{\ne 0} {:=} {\text {Span}}\,\left\{ \vartheta _{j\ell }: \nabla _{g_b}\otimes \vartheta _{j\ell } \in \Lambda _{\operatorname {QNM}, \Upsilon }^{k}(\mathbb {L}_{g_b}, \sigma _j), \sigma _j\in \Lambda _{\operatorname {QNF}, \Upsilon }^{k}(\mathbb {L}_{g_b}, \mathbb {H}^+_\epsilon )\right\} ; \end{aligned}$$

the span of the zero-frequency gauge choices that correspond to the non-linearized Kerr-de Sitter zero-frequency $\textbf{H}^{k}$-geometric quasinormal mode solutions,

$$\begin{aligned} \Theta _0{:=} {\text {Span}}\,\left\{ \vartheta _{\ell }: \nabla _{g_b}\otimes \vartheta _\ell \in \Lambda _{\operatorname {QNM}, \Upsilon }^{k}(\mathbb {L}_{g_b}, 0)\backslash \left\{ (g_b')^\Upsilon (b')^{(0)}\right\} \right\} ; \end{aligned}$$

and the space of possible initial-data modifications corresponding to the $\textbf{H}^{k}$-constraint quasinormal mode solutions,

$$\begin{aligned} \Upsilon _b {:=} {\text {Span}}\,\left\{ \gamma _0(\psi _{j\ell }): \psi _{j\ell }\in \Lambda _{\operatorname {QNM}, \mathcal {C}}^{k}(\mathbb {L}_{g_b}, \mathbb {H}^+)\right\} . \end{aligned}$$

Just as was the case for Proposition 10.4, we now have that defining $\Theta = \Theta _0\oplus \Theta _{\ne 0}$, we have the space of initial value problem modifications,

$$\begin{aligned} \mathcal {Z}{:=} (0, \gamma _0((g_{b}')^\Upsilon (b')) +(0, \gamma _0(\nabla _{g_b}\otimes \Theta )) + (0, \Upsilon _b) \subset D^{\infty ,{\boldsymbol{\alpha }}}(\mathcal {M}, S^2T^*\mathcal {M}), \end{aligned}$$

for which the map $\lambda _{\mathcal {Z}}$ as defined in Corollary 4.13 is bijective. The conclusions of the proposition then follow from Corollary 4.13. It is also clear from the construction that if $({h}_0, {h}_1)$ satisfy the linearized gauge constraint, then $\upsilon =0$. We also remark that applying the perturbation theory in Proposition 4.14 to $\Box ^{CP}_{g_b}$, it is clear that $\Theta$ can be constructed to depend continuously on the black hole parameters *b*. $\square$

Observe that as constructed in Proposition 10.6, the space $\Theta$ from which we can construct $\textbf{H}^{k}$-geometric quasinormal mode solutions is dependent on the Kerr-de Sitter metric $g_b$ around which we linearize. While this is perfectly fine for the linearized stability statement, it will be desirable in the nonlinear theory to show that in fact, we can choose $\Theta$ independent of our choice of $g_b$.

#### Corollary 10.7

Let $(g'_b)^\Upsilon (b')$ be as defined in Proposition 10.6, and fix some $k>k_0$, and ${\boldsymbol{\alpha }}>0$. Let $\Upsilon _b$ denote the $\Upsilon$ constructed for the choice of Kerr-de Sitter black hole parameters *b*, as in Proposition 10.6.

Then there exists a fixed finite dimensional linear subspace $\Theta \subset H^{k,-\textbf{M}}(\mathcal {M}, T^*\mathcal {M})$ with $-\textbf{M}$ as constructed in 4.11, and a sufficiently small neighborhood $\mathcal {B}$ of $b_0$ such that for any $b\in \mathcal {B}$, for any $(f, {h}_0, {h}_1)\in D^{k+1,{\boldsymbol{\alpha }}}(\mathcal {M})$ there exist unique $b'\in T_{b}B$, $\vartheta \in \Theta$, and $\upsilon \in \Upsilon _b$, such that the solution of the Cauchy problem

$$\begin{aligned} \begin{aligned} \mathbb {L}_{g_b}\tilde{{h}}&= f,\\ \gamma _0(\tilde{{h}})&=({h}_0, {h}_1) - \upsilon - \gamma _0\left( g_{b}'(b') + \nabla _{g_b}\otimes \vartheta \right) . \end{aligned} \end{aligned}$$

satisfies the pointwise bound

$$\begin{aligned} \left\Vert \tilde{{h}}\right\Vert _{\overline{H}^{k}(\Sigma _{t_*})} \lesssim e^{-{\boldsymbol{\alpha }}t_*}\left\Vert (f, {h}_0, {h}_1)\right\Vert _{D^{k+1,{\boldsymbol{\alpha }}}(\mathcal {M})}, \end{aligned}$$

and moreover, the map $(f, {h}_0, {h}_1)\mapsto (b', \vartheta , \upsilon )$ is linear and continuous. In addition, if $({h}_0, {h}_1)$ satisfy the linearized gauge constraint, then in fact $\upsilon =0$.

#### Proof

Consider $\tilde{\mathcal {Z}}_b\subset D^{k,{\boldsymbol{\alpha }}}(\mathcal {M})$ defined by

$$\begin{aligned} \tilde{\mathcal {Z}}_b = T_bB \times \Theta _{b_0}\times \Upsilon _b. \end{aligned}$$

We will prove the corollary by showing that

$$\begin{aligned} \lambda _{\tilde{\mathcal {Z}}_b}{:=}\lambda _{IVP}\vert _{\tilde{\mathcal {Z}}_b}: \tilde{\mathcal {Z}}_b\rightarrow \Lambda _{\operatorname {QNF}}^{k*}(\mathbb {L}_{g_{b_0}}, \mathbb {H}^+) \end{aligned}$$

is an isomorphism.

We begin by parameterizing the space $\tilde{\mathcal {Z}}_b$ by picking an isomorphism

$$\begin{aligned} \varTheta :\mathbb {R}^{N_\Theta } \rightarrow \Theta _{b_0},\qquad N_{\Theta } = \dim \Theta _{b_0}, \end{aligned}$$

which induces an isomorphism

$$\begin{aligned} \tilde{z}_b: \mathbb {R}^{4 + N_{\Theta } + N_{\Upsilon }}&\rightarrow \tilde{\mathcal {Z}}_b\\ (b', w_\Theta , \upsilon )&\mapsto \left( 0, \gamma _0\left( (g_b')^\Upsilon (b') + \nabla _{g_b}\otimes \varTheta (w_\Theta )\right) + \upsilon \right) , \end{aligned}$$

and is clearly continuous in *b*. Then, we see that the mapping

$$\begin{aligned} \lambda _{\tilde{\mathcal {Z}}_{b}}\circ \tilde{z}_b^{-1} \end{aligned}$$

depends continuously on *b*. Moreover, from Proposition 10.4, we know that $\lambda _{\tilde{\mathcal {Z}}_{b_0}}\circ \tilde{z}_{b_0}^{-1}$ is bijective, and as a result, for $b\in \mathcal {B}$ sufficiently small, $\lambda _{\tilde{\mathcal {Z}}_{b}}\circ \tilde{z}_b^{-1}$ is bijective as well by semicontinuity of rank^26^. The rest of the conclusions follow as before. $\square$

## Proof of the main theorem

We can now prove Theorem 5.1.

### Proof of Theorem 5.1

Let us denote $({h}_0, {h}_1)=D_{(\underline{g}_b, k_b)}i_{b,\operatorname {Id}}(\underline{g}', k')$ the initial data for the gauge-fixed linearized Einstein equations in harmonic gauge. By the construction of $D_{(\underline{g}_b, k_b)}i_{b,\operatorname {Id}}$ in Corollary 3.7, we have that $(h_0, h_1)$ satisfies the linearized harmonic gauge constraint on $\Sigma _0$. Thus, using Proposition 10.6, there exists some $b'\in T_b\mathcal {B}, \vartheta \in \Theta$ satisfying the control in (77) such that the solution *h* of the Cauchy problem

$$\begin{aligned} \begin{aligned} \mathbb {L}_{g_b}\tilde{h}&= 0,\\ \gamma _0(\tilde{h})&= ({h}_0, {h}_1) - \gamma _0\left( g_{b}'(b') + \nabla _{g_b}\otimes \vartheta \right) , \end{aligned} \end{aligned}$$

satisfies the decay estimate

$$\begin{aligned} \begin{aligned} \sup _{t_*}e^{{\boldsymbol{\alpha }}t_*}\left\Vert \tilde{h}\right\Vert _{\overline{H}^{k}(\Sigma _{t_*})}&\lesssim \left\Vert ({h}_0, {h}_1)\right\Vert _{\textbf{H}^{k+1}(\Sigma _0)}. \end{aligned} \end{aligned}$$

Then,

$$\begin{aligned} h = g_{b}'(b') + \nabla _{g_b}\otimes \vartheta + \tilde{h} \end{aligned}$$

solves

$$\begin{aligned} \begin{aligned} \mathbb {L}_{g_b}h&= 0,\\ \gamma _0(h)&= (h_0, h_1), \end{aligned} \end{aligned}$$

and has the desired form. $\square$

## Appendix A: Background Functional Analysis

### Theorem A.1

(Analytic Fredholm Theorem) Let $\Omega \subset \mathbb {C}$ be a domain in the complex plane. Let *H* be a Hilbert space, and $\mathcal {L}(H)$ the space of bounded linear operators from *H* to itself. Also let $\iota$ denote the identity operator. Then, for $A:\Omega \rightarrow \mathcal {L}(H)$ a mapping such that

1. *A* is analytic on $\Omega$ in the following sense:
  $$\begin{aligned} \lim _{\sigma \rightarrow \sigma _0}\frac{A(\sigma )-A(\sigma _0)}{\sigma -\sigma _0} \end{aligned}$$
  exists for all $\sigma _0\in \Omega$; and
2. the operator $A(\sigma )$ is a compact operator for every $\sigma \in \Omega$.

Then we can conclude that either

1. $(\iota -A(\sigma ))^{-1}$ does not exist for any $\sigma \in \Omega$; or
2. There exists a discrete subset $\Sigma \subset \Omega$ (in particular, $\Sigma$ has no limit points in $\Omega$), such that $(\iota -A(\sigma ))^{-1}$ exists for every $\sigma \in \Omega \backslash \Sigma$, and the function $\sigma \rightarrow (\iota -A(\sigma ))^{-1}$ is analytic on $\Omega \backslash \Sigma$ in the sense above and the equation
  $$\begin{aligned} A(\sigma )\psi =\psi \end{aligned}$$
  has a finite-dimensional family of solutions for any $\sigma \in \Sigma$. Moreover $(\iota -A(\sigma ))^{-1}$ is meromorphic in $\Omega$ with poles at $\Sigma$ where the residues at the poles are finite rank operators.

### Lemma A.2

(Lemma 1.3 [21]) Let $(D^k(\mathcal {A}), \mathcal {A})$ be the infinitesimal generator for the $C^0$-solution semigroup $\mathcal {S}(t_*)$. Then for $\sigma \in \mathbb {C}, t_*>0$, we have that for all $F\in \textbf{H}^{k}(\Sigma )$,

$$\begin{aligned} \mathbbm {i}\left( e^{\mathbbm {i}\sigma t_*}\mathcal {S}(t_*)F - F\right) = (\mathcal {A}-\sigma )\int _0^{t_*} e^{\mathbbm {i}\sigma {s_*}}\mathcal {S}({s_*})F\,d{s_*}; \end{aligned}$$

and for all ${h}\in D^k(\mathcal {A})$,

$$\begin{aligned} \mathbbm {i}\left( e^{\mathbbm {i}\sigma t_*}\mathcal {S}(t_*){h} - {h}\right) = \int _0^{t_*} e^{\mathbbm {i}\sigma {s_*}}\mathcal {S}({s_*})(\mathcal {A}- \sigma ){h}\,d{s_*}. \end{aligned}$$

The Mather Division Theorem introduced below is also referred to as the Malgrange Preparation Theorem.

### Theorem A.3

(Mather Division Theorem [47]) If *f*(*t*, *x*) is a smooth complex function of $t\in \mathbb {R}$, and $x\in \mathbb {R}^n$ near the origin, and *k* is the smallest integer such that

$$\begin{aligned} \frac{d^k f}{d t^k}(\textbf{0}) \ne 0, \end{aligned}$$

and moreover, *g* is a smooth function near the origin, then we can write

$$\begin{aligned} g = qf + r, \end{aligned}$$

where *q* and *r* are smooth, and

$$\begin{aligned} r= \sum _{0\le j<k }t^jr_j(x) \end{aligned}$$

for some family of smooth functions $r_j(x)$.

## Appendix B: Appendix to Section 2

### B.1 Proof of Lemma 2.1

Define the cutoff functions $\chi _{\mathcal {I}_{b_0},-}, \chi _{\mathcal {I}_{b_0},+}\in C_0^\infty (r_{\mathcal {H}^+}-\varepsilon _{\mathcal {M}}, r_{\overline{\mathcal {H}}^+}+\varepsilon _{\mathcal {M}})$ such that

$$\begin{aligned} \chi _{\mathcal {I}_{b_0}, -}(r) = {\left\{ \begin{array}{ll} 1 &  {\text {near }}\mathcal {H}^+\\ 0 &  r>\inf \mathcal {I}_{b_0} \end{array}\right. },\qquad \chi _{\mathcal {I}_{b_0}, +}(r) = {\left\{ \begin{array}{ll} 1 &  {\text {near }}\overline{\mathcal {H}}^+\\ 0 &  r<\sup \mathcal {I}_{b_0} \end{array}\right. }. \end{aligned}$$

Now we choose

B1 $$\begin{aligned} F_{b_0}'(r) = \left( \chi _{\mathcal {I}_{b_0},+}-\chi _{\mathcal {I}_{b_0}, -}\right) \mu _{b_0}^{-1}\sqrt{1-c_{t_*}^2\mu _{b_0}(r)}, \end{aligned}$$

where $c_{t_*} = \left( 1 - \root 3 \of {9\Lambda M^2}\right) ^{-\frac{1}{2}}$. $F_{b_0}'$ clearly vanishes on $\mathcal {I}_{b_0}$. To check that the constant-$t_*$ hypersurfaces are uniformly spacelike, we can compute that

$$\begin{aligned} G_{b_0}(dt_*, dt_*) = \frac{1}{\mu _{b_0}}\left( -1 + \left( \chi _{\mathcal {I}_{b_0},+}-\chi _{\mathcal {I}_{b_0}, -}\right) ^2\right) -\left( \chi _{\mathcal {I}_{b_0},+}-\chi _{\mathcal {I}_{b_0}, -}\right) ^2c_{t_*}^2, \end{aligned}$$

which is uniformly negative.

### B.2 Proof of Lemma 2.2

We will construct an explicit coordinate system that satisfies the conditions in the lemma. To define $F'_b(r), \Phi _b'(r)$, fix $\mathcal {I}_{b}=r_1, r_2$ such that $r_{b_0, \mathcal {H}^+}+\epsilon _{\mathcal {M}}<r_1<r_2<r_{b_0,\overline{\mathcal {H}}^+} - \epsilon _{\mathcal {M}}$, and define smooth cutoff functions $\chi _{\mathcal {I}_b,\mathcal {H}^+}, \chi _{\mathcal {I}_b,\overline{\mathcal {H}}^+}$ such that

$$\begin{aligned} \chi _{\mathcal {I}_b,\mathcal {H}^+}(r) {:=} {\left\{ \begin{array}{ll} 1& {\text {near }} \mathcal {H}^+\\ 0& r > r_1 \end{array}\right. },\qquad \chi _{\mathcal {I}_b,\mathcal {H}^+}(r) {:=} {\left\{ \begin{array}{ll} 1& {\text {near }} \overline{\mathcal {H}}^+\\ 0& r < r_2 \end{array}\right. }. \end{aligned}$$

Then we define $F_b'(r), \Phi _b'(r)$ so that

$$\begin{aligned} F_b'(r)&= F_{b_0}'(r) + (\chi _{\mathcal {I}_b,\mathcal {H}^+} - \chi _{\mathcal {I}_b,\overline{\mathcal {H}}^+})\left( \mu _{b_0}^{-1} - \frac{(1+\lambda _b)(r^2+a^2)}{\Delta _b} \right) ,\\ \Phi _b'(r)&= (\chi _{\mathcal {I}_b,\mathcal {H}^+} - \chi _{\mathcal {I}_b,\overline{\mathcal {H}}^+})\left( \frac{(1+\lambda _b)a}{\Delta _b} \right) . \end{aligned}$$

This definition ensures that $F_b$, $\Phi _b$ are well-defined up to an additive constant. Furthermore, it is immediately clear that $F_b(r) = {\Phi }_b(r) = 0$ on $\mathcal {I}_b$, and that $F_{b}', \Phi _{b}'$ coincide with $F_{b_0}', \Phi _{b_0}'$ when $b=b_0$. Finally, to check that the $t_*$-constant hypersurfaces are space-like, it suffices to observe that

$$\begin{aligned} G_b(dt_*,dt_*) = G_{b_0}(dt_*, dt_*) + O(a), \end{aligned}$$

where the perturbation in particular follows from the fact that *r* is bounded. Since we have that $G_{b_0}(dt_*, dt_*)<0$ uniformly, $t_*$-constant hypersurfaces remain space-like in $g_b$ for sufficiently small *a*.

Near the poles, we can use the smooth coordinates on $\mathbb {S}^2$ given by $x = \sin \theta \cos {\varphi _*}$ and $y=\sin \theta \sin {\varphi _*}$ to extend the metric smoothly to the poles.

### B.3 Proof of Proposition 2.7

We define the redshift vectorfield $\textbf{Y}$ such that on $\mathcal {H}=\mathcal {H}^+, \overline{\mathcal {H}}^+$,

1. $\textbf{Y}$ is future-directed null, with $g(\textbf{Y}, \textbf{K}_{\mathcal {H}}) = -2$,
2. $$\nabla _\textbf{Y}\textbf{Y}= -C_\textbf{Y}(\textbf{Y}+\textbf{K}_{\mathcal {H}})$$,
3. $$\mathcal {L}_\textbf{T}\textbf{Y}= \mathcal {L}_{\Phi }\textbf{Y}= 0$$.

Define a local frame $\omega _1$, $\omega _2$ on the spheres. Then at $\mathcal {H}^+$ for some vectorfield $a^A$ and two-tensor ,

B2

We can then calculate that at $\mathcal {H}^+$,

Then, at $\mathcal {H}^+$, we have that

$$\begin{aligned} K^{\textbf{Y}, 0, 0}[{h}] \ge {}&\frac{1}{2}\kappa _{\mathcal {H}^+} \mathbb {T}(\textbf{Y},\textbf{Y}) + \frac{1}{4}C_\textbf{Y}\mathbb {T}(\textbf{K}_{\mathcal {H}^+}, \textbf{Y}+ \textbf{K}_{\mathcal {H}^+})\\&- c\mathbb {T}(\textbf{K}_{\mathcal {H}^+}, \textbf{Y}+ \textbf{K}_{\mathcal {H}^+}) - c\sqrt{\mathbb {T}(\textbf{K}_{\mathcal {H}^+}, \textbf{Y}+ \textbf{K}_{\mathcal {H}^+})\mathbb {T}(\textbf{Y}, \textbf{Y})}. \end{aligned}$$

Thus, for any $\epsilon >0$, for $C_\textbf{Y}$ sufficiently large, we have that on $\mathcal {H}^+$,

B3 $$\begin{aligned} K^{\textbf{Y}, 0, 0}[{h}] \ge \frac{1}{2}(\kappa _{\mathcal {H}^+} - \epsilon )\mathbb {T}(\textbf{Y},\textbf{Y}) + c(\epsilon )\mathbb {T}(\textbf{K}_{\mathcal {H}^+}, \textbf{Y}+ \textbf{K}_{\mathcal {H}^+}) \end{aligned}$$

for some $c(\epsilon )>0$. In particular, for any fixed $C>0$, we can choose $C_\textbf{Y}$ sufficiently large so that along $\mathcal {H}^+$,

B4 $$\begin{aligned} K^{\textbf{Y}, 0, 0}[{h}] \ge \frac{1}{4}\kappa _{\overline{\mathcal {H}}^+}\mathbb {T}(\textbf{Y},\textbf{Y}) + C\mathbb {T}(\textbf{K}_{\overline{\mathcal {H}}^+}, \textbf{Y}+ \textbf{K}_{\overline{\mathcal {H}}^+}). \end{aligned}$$

A similar argument shows that $C_{\textbf{Y}}$ can be chosen sufficiently large so that in fact (B4) also holds at $\overline{\mathcal {H}}^+$. We then define

$$\begin{aligned} \textbf{N}= {\left\{ \begin{array}{ll} \textbf{K}_{\mathcal {H}^+}+\textbf{Y}&  r\le r_0\\ \textbf{T}&  r\in (r_1, R_1)\\ \textbf{K}_{\overline{\mathcal {H}}^+}+\textbf{Y}&  r\ge R_0, \end{array}\right. } \end{aligned}$$

where on the regions $(r_0, r_1)$, $(R_1, R_0)$ we smoothly extend $\textbf{N}$ as a $\textbf{T}$, ${\Phi }$-independent timelike vectorfield. Then we have (29), (30), and (31) by construction.

We now move onto proving (28). Recalling the definition of $\widetilde{\textbf{T}}$ in (33), and (B4), we have that on $\mathcal {H}^+$,

B5 $$\begin{aligned} K^{\textbf{Y}, 0, 0}[h] \ge \frac{1}{2}\left( \kappa _{\mathcal {H}^+}-\varepsilon _{\textbf{N}}\right) \mathbb {T}(\textbf{Y},\textbf{Y}) + C(\varepsilon _{\textbf{N}})\mathbb {T}(\widetilde{\textbf{T}}, \textbf{N}), \end{aligned}$$

where we observe that $C(\varepsilon _{\textbf{N}})$ can be made arbitrarily large by increasing $C_\textbf{Y}$ as desired in the construction of $\textbf{Y}$.

Now observe that at the horizons, the unit normal $n_{\Sigma _{t_*}}$ can be decomposed as

$$\begin{aligned} n_{\Sigma _{t_*}} = \frac{1}{2\sqrt{A}}\textbf{Y}+ \tilde{n}, \end{aligned}$$

where $\tilde{n}$ is tangent to $\mathcal {H}^+$ and $\overline{\mathcal {H}}^+$.

As a result, we have that on a neighborhood of the horizons, in particular, for $r<r_0$, $r>R_0$,

$$\begin{aligned} K^{\textbf{Y}, 0, 0}[h] \ge \frac{1}{\sqrt{A}}\left( \kappa _{\mathcal {H}^+} - \varepsilon _{\textbf{N}}\right) J^{\textbf{N}, 0, 0}[h]\cdot n_{\Sigma } + C(\varepsilon _{\textbf{N}})\mathbb {T}(\widetilde{\textbf{T}}, \textbf{N}). \end{aligned}$$

Now recall that $\widetilde{\textbf{T}}$ is timelike on the interior of the stationary region, only becoming null exactly at $\mathcal {H}^+$, and that $\textbf{N}$ is globally timelike. Then, $\mathbb {T}(\widetilde{\textbf{T}}, \textbf{N})$ controls all tangential derivatives of *h* along the horizons, and all derivatives of *h* away from the horizons. In particular, since *X* is tangential to both $\mathcal {H}^+$ and $\overline{\mathcal {H}}^+$,

$$\begin{aligned} \left\lvert X h \right\rvert ^2 \lesssim \mathbb {T}(\widetilde{\textbf{T}}, \textbf{N}). \end{aligned}$$

Choosing $C_{\textbf{Y}}$ sufficiently large so that $C(\varepsilon _{\textbf{N}})\mathbb {T}(\widetilde{\textbf{T}}, \textbf{N})$ is large enough to control *Xh* then allows us to conclude.

The fact that the divergence of $\textbf{N}$ is negative follows directly from the computation of the components of  above.

### B.4 Proof of Lemma 2.8

We first pick stationary, smooth $\mathcal {K}_1$ such that $g(\mathcal {K}_1,\textbf{K}_{\mathcal {H}})=-1$ near $\mathcal {H}$, for $\mathcal {H}=\mathcal {H}^+, \overline{\mathcal {H}}^+$ and vanishes on a neighborhood away from the horizons. Now consider some $\Sigma =\Sigma _{t_*}$. Any coordinate chart $(U, \Psi )$ on $\Sigma$ can be pushed forward to a tubular coordinate patch on $\mathcal {M}$ by the map

$$\begin{aligned} \mathbb {R}_+\times U&\rightarrow \mathcal {M}\\ (t_*, x)&\mapsto \varphi _{t_*}\circ \Psi (x). \end{aligned}$$

In such a coordinate chart, the metric coefficients are independent of $t_*$. Now, pick an arbitrary point $\textbf{x}=(t_*,x)\in \Sigma$. We now split into two cases, a case where we are not on the horizons, and a case where we are on one of $\mathcal {H}^+$ and $\overline{\mathcal {H}}^+$.

1. $\textbf{x}\in \Sigma \backslash (\mathcal {H}^+\bigcup \overline{\mathcal {H}}^+)$: In this case, we pick a coordinate chart $(U,\Psi )$ such that $\textbf{x}\in \Psi (U)\Subset \Sigma \backslash (\mathcal {H}^+\bigcup \overline{\mathcal {H}}^+)$. Now define $\chi$ a cut-off function such that $\chi =1$ in a neighborhood of *p* and ${\text {supp}}\,\chi \subset U$. Then define the vectorfields
  $$\begin{aligned} \mathcal {K}^{(\textbf{x})}_\mu {:=} \chi \partial _\mu \end{aligned}$$
  on the tubular patch $\mathbb {R}_+\times U$, where $\partial _0=\textbf{K}_{\mathcal {H}^+}+\textbf{K}_{\overline{\mathcal {H}}^+}, \partial _i=\partial _{x^i}$. Each of the $\mathcal {K}^{\textbf{x}}_\mu$ are stationary vectorfields since the coordinate chart is stationary, and they are also trivially tangent to both $\mathcal {H}^+$ and to $\overline{\mathcal {H}}^+$. Finally, since all the vectorfields themselves vanish near the two horizons, we also have that
  near both $\mathcal {H}^+$ and $\overline{\mathcal {H}}^+$.
2. $\textbf{x}\in \mathcal {H}^+\bigcup \overline{\mathcal {H}}^+$. The argument for both cases are similar, so we only give details in the case of $\textbf{x}\in \overline{\mathcal {H}}^+$: We can pick a chart $(U, \Psi )$ on $\Sigma$ such that $\textbf{x}\in \Psi (U)$, and moreover, $\overline{\mathcal {H}}^+$ is given locally by $x_1=0$, and $\partial _{x^1} = \textbf{Y}$ is normal to $\overline{\mathcal {H}}^+$. Like in the previous case, define a cut-off function $\chi$ such that $\chi =1$ near $\textbf{x}$ and ${\text {supp}}\,\chi \in U$. Then, in the tubular neighborhood $\mathbb {R}_+\times U$, define
  $$\begin{aligned} \mathcal {K}^{(\textbf{x})}_0 = \chi \textbf{K}_{\overline{\mathcal {H}}^+}, \qquad \mathcal {K}^{(\textbf{x})}_1 = \chi x_1\partial _{x^1}, \qquad \mathcal {K}^{(\textbf{x})}_i = \chi \partial _{x^i}\quad 2\le i \end{aligned}$$
  By construction, all of these vectors are smooth and tangent to $\overline{\mathcal {H}}^+$. Moreover, they vanish on and thus are tangent to $\mathcal {H}^+$.

At this point, we have constructed a tubular neighborhood $V_{(\textbf{x})}$ around each point $\textbf{x}\in \Sigma$ and a set of smooth vectorfields which are stationary and tangent to both $\overline{\mathcal {H}}^+$ and $\mathcal {H}^+$. Moreover, for $\mathcal {K}$ a smooth vectorfield tangent to both $\overline{\mathcal {H}}^+$ and $\mathcal {H}^+$, supported in $V_{(\textbf{x})}$, there exist stationary $(x^{(\textbf{x})})^\mu \in C^\infty (\mathcal {M})$ such that

$$\begin{aligned} \mathcal {K}= (x^{(\textbf{x})})^\mu \mathcal {K}^{(\textbf{x})}_\mu . \end{aligned}$$

Now, crucially, we realize that $\Sigma$ is compact in Kerr-de Sitter. This allows us to choose a finite set of points $\{\textbf{x}_i\in \Sigma \}_{i=1}^N$ such that

$$\begin{aligned} \mathcal {M}\subset \bigcup _{i=1}^N V_{(\textbf{x}_i)}. \end{aligned}$$

Now re-index $\{\mathcal {K}_i\}_{i=2}^N$ to be the union of the $\{\mathcal {K}^{(p_i)}_\mu \}_{i=1}^N$. This is now a finite set of vectorfields such that for any $X\in C^\infty (\mathcal {M})$ there exist functions $x^i\in C^\infty (\mathcal {M})$ such that

$$\begin{aligned} X = \sum _i x^i\mathcal {K}_i. \end{aligned}$$

It remains to confirm the properties of the deformation tensors. Directly from our construction of $\textbf{Y}$, we have that

$$\begin{aligned} f_{1}^{11} = \kappa _{\mathcal {H}} \qquad {\text {near }}\mathcal {H}, \end{aligned}$$

where $$\mathcal {H} = \mathcal {H}^+,\overline{\mathcal {H}}^+$$. Finally,

$$\begin{aligned} f_i^{11} = 0,\quad i\ne 1 \end{aligned}$$

follows directly from that $\mathcal {K}_i,i\ne 1$ is tangent to $\mathcal {H}^+$ and $\overline{\mathcal {H}}^+$, and that

### B.5 Proof of Lemma 2.9

We define $\tilde{\chi }$ by

$$\begin{aligned} a\tilde{\chi }(r) {:=} {\left\{ \begin{array}{ll} \frac{a}{r^2+a^2}&  r\in [r_{\mathcal {H}^+}, \sup _{\mathcal {E}_{\mathcal {H}^+}}r],\\ \frac{a}{r^2+a^2}&  [\inf _{\mathcal {E}_{\overline{\mathcal {H}}^+}}r, r_{\overline{\mathcal {H}}^+}],\\ 0&  r\in [r_-,r_+], \end{array}\right. } \end{aligned}$$

where

B6 $$\begin{aligned} \sup _{\mathcal {E}_{\mathcal {H}^+}}r< r_-< r_0,\qquad R_0< r_+ < \inf _{\mathcal {E}_{\overline{\mathcal {H}}^+}}r, \end{aligned}$$

so $\tilde{\chi }$ clearly satisfies the desired support assumptions, and (34) holds. To verify that $\widetilde{\textbf{T}}$ is timelike on the interior of the stationary region and becomes null on the horizons, we observe that ${\widetilde{\textbf{T}}}\big \vert _{r_{\mathcal {H}}} = \textbf{K}_{\mathcal {H}}$ and ${\widetilde{\textbf{T}}}\big \vert _{r_{\overline{\mathcal {H}}}} = \textbf{K}_{\overline{\mathcal {H}}}$ so $\widetilde{\textbf{T}}$ is clearly null on both $\mathcal {H}$ and $\overline{\mathcal {H}}$. To show that $\widetilde{\textbf{T}}$ is timelike on $\mathcal {M}^{\circ }\backslash (\mathcal {H}\bigcup \overline{\mathcal {H}})$, we can compute that

$$\begin{aligned} g_b\left( \textbf{T}+ \frac{a}{r^2+a^2}{\Phi }, \textbf{T}+ \frac{a}{r^2+a^2}{\Phi }\right) ={} - \frac{\Delta \left\lvert q\right\rvert ^2}{(1+\gamma )^2(r^2+a^2)^2}, \end{aligned}$$

so we see that $\textbf{T}+ \frac{a}{r^2+a^2}{\Phi }$ is timelike on $\mathcal {M}^{\circ }\backslash (\mathcal {H}\bigcup \overline{\mathcal {H}})$. Since $\textbf{T}$ is also timelike outside of the ergoregion, we see that $\widetilde{\textbf{T}}= \textbf{T}+ a\tilde{\chi }{\Phi }$ is timelike in the ergoregions of Kerr-de Sitter, and the interpolation of two timelike vectors, and therefore, also itself timelike outside the ergoregions.

## Appendix C: Appendix to Section 3

### C.1 Proof of Proposition 3.6

Let us define $\psi = \phi ^{-1}$. Then, denote

$$\begin{aligned} \tilde{\underline{g}}_0 = \psi ^*\underline{g}_0, \qquad \tilde{k}_0 = \psi ^*k_0. \end{aligned}$$

Fix $N_b$ and $X_b$ the lapse function and the shift vectorfield respectively of $g_b$, so that

$$\begin{aligned} g_b = -N_b^2\,dt_*^2 + (\underline{g}_b)_{ij}(d x^i + X_b^i\,dt_*)\otimes (dx^j + X_b^j\,dt_*). \end{aligned}$$

Then define *g* so that

$$\begin{aligned} g = -N_b^2\,dt_*^2 + (\tilde{\underline{g}}_0)_{ij}(d x^i + X_b^i\,dt_*)\otimes (dx^j + X_b^j\,dt_*). \end{aligned}$$

It remains to define $\partial _{t_*}g_{\mu \nu }$. We first define

$$\begin{aligned} \partial _{t_*}g_{ij} = \mathcal {L}_{X_b}\tilde{\underline{g}}_0 + 2N_{b} (\tilde{k}_0)_{ij}. \end{aligned}$$

It remains to determine $\partial _{t_*}g(\textbf{T}, \cdot )$. We do so exactly by using the gauge condition. Recall that in local coordinates, the gauge condition can be written as

$$\begin{aligned} 0 = g^{\alpha \beta }\partial _\alpha g_{\mu \beta } - \frac{1}{2}g^{\alpha \beta }\partial _\mu g_{\alpha \beta } - g_{\mu \gamma }g^{\alpha \beta }\Gamma (g_b)^{\gamma }_{\alpha \beta } . \end{aligned}$$

Contracting this with $\textbf{T}$, we have then that

C7 $$\begin{aligned} \frac{1}{2}g^{t_*t_*}\partial _{t_*}g_{t_*t_*} = - g^{i\beta }\partial _i g_{t_*\beta } + \frac{1}{2}g^{i j}\partial _{t_*}g_{ij} + g_{t_*\gamma }g^{\alpha \beta }\Gamma (g_b)^{\gamma }_{\alpha \beta }, \end{aligned}$$

which uniquely determines $\partial _{t_*}g_{t_*t_*}$. On the other hand, contracting with $\partial _i$, we have that

$$\begin{aligned} g^{t_*t_*}\partial _{t_*}g_{it_*} = \frac{1}{2}g^{\alpha \beta }\partial _ig_{\alpha \beta } - g^{\alpha j}\partial _{j}g_{i\alpha } + g_{i\gamma }g^{\alpha \beta }\Gamma (g_b)^{\gamma }_{\alpha \beta }, \end{aligned}$$

which uniquely determines $\partial _{t_*}g_{t_*i}$. We can then define

$$\begin{aligned} i_{b,\phi }(\underline{g}, k) = \left( (g - g_b)\vert _{\Sigma _0}, \mathcal {L}_{\textbf{T}}g\vert _{\Sigma _0}\right) . \end{aligned}$$

Denoting $(\underline{g}, k)$ the induced metric and second fundamental form of *g* on $\phi (\Sigma _0)$, it is clear that $(\phi ^*\underline{g}, \phi ^*k)=(g_0, k_0)$, and moreover, we have constructed $(g\vert _{\Sigma _0}, \mathcal {L}_\textbf{T}g\vert _{\Sigma _0})$ satisfying the gauge constraint. The smoothness and mapping properties follow by construction.

### C.2 Proof of Lemma 3.5

Throughout this section, we work with the Eddington-Finkelstein coordinates on Schwarzschild-de Sitter defined in Sect. 2.2. We will then denote covectors in this section by

$$\begin{aligned} -\sigma dt_0+ \xi dr + \eta _{\theta }d\theta + \eta _{\varphi }d\varphi _0. \end{aligned}$$

Then we can write the metric $g_{b_0}$ and the inverse metric $G_{b_0}$ as

C8

where $\pm (r-r_{{\text {crit}}})>0$. Recall that in this system of coordinates, we have that $\partial _{t_0}$ is Killing, and in particular is tangential to the two Killing horizons. Let us now define

$$\begin{aligned} e_0{:=}\sqrt{\mu _{b_0}}^{-1}\partial _t, \quad e^0{:=} \sqrt{\mu _{b_0}}dt, \quad e_1{:=} \sqrt{\mu _{b_0}}\partial _r, \quad e^1{:=} \sqrt{\mu _{b_0}}^{-1} dr. \end{aligned}$$

To achieve a smooth splitting near $r_{b_0,\mathcal {H}^+}, r_{b_0,\overline{\mathcal {H}}^+}$, we split any smooth one-form *w* into

$$\begin{aligned} w = w^0_N \,dt_0 + w^0_{TN}\, dr + w^0_{TT}, \end{aligned}$$

where $w^0_N, w^0_{TN}$ are smooth, and $w^0_{TT}$ is a smooth one-form on the 2-sphere $\mathbb {S}^2$. This transforms between the splitting

$$\begin{aligned} w = w_N\,e^0 + w_{TN}\,e^1+w_{TT} \end{aligned}$$

via

$$\begin{aligned} \begin{pmatrix} w_N\\ w_{TN}\\ w_{TT} \end{pmatrix} = \mathcal {J}_{\pm }^{(1)} \begin{pmatrix} w^0_N\\ w^0_{TN} \\ w^0_{TT} \end{pmatrix}, \quad \mathcal {J}_{\pm }^{(1)} = \begin{pmatrix} \sqrt{\mu _{b_0}}^{-1} &  0 &  0\\ \mp \sqrt{\mu _{b_0}}^{-1} &  \sqrt{\mu _{b_0}} &  0 \\ 0 &  0 &  1 \end{pmatrix}. \end{aligned}$$

This induces a splitting on two tensors *u* as

C9 $$\begin{aligned} u = u^0_{NN}\,dt_0^2 + 2u^0_{NTN}\,dt_0dr + 2\,dt_0 u^0_{NTT} + u^0_{TNN}\,dr^2 + 2\,dr u^0_{TNT} + u^0_{TTT}, \end{aligned}$$

where $u^0_{NN}, u^0_{NTN}, u^0_{TNN}$ are functions on $T^*\mathcal {M}$, $u^0_{NTT}$ and $u^0_{TNT}$ are one forms on the sphere, and $u^0_{TTT}$ is a two-tensor on the sphere. This then transforms between the splitting in (C9) via

$$\begin{aligned} \begin{pmatrix} u_{NN} \\ u_{NTN} \\ u_{NTT} \\ u_{TNN} \\ u_{TNT} \\ u_{TTT} \end{pmatrix} = \mathcal {J}_{\pm }^{(2)} \begin{pmatrix} u^0_{NN} \\ u^0_{NTN} \\ u^0_{NTT} \\ u^0_{TNN} \\ u^0_{TNT} \\ u^0_{TTT} \end{pmatrix}, \quad \mathcal {J}_{\pm }^{(2)} = \begin{pmatrix} \mu _{b_0}^{-1} &  0 &  0 &  0 &  0 &  0\\ \mp \mu _{b_0}^{-1} &  1 &  0 &  0 &  0 &  0\\ 0 &  0 &  \sqrt{\mu _{b_0}}^{-1} &  0 &  0 &  0\\ \mu _{b_0}^{-1} &  \mp 2 &  0 &  \mu _{b_0} &  0 &  0\\ 0 &  0 &  \mp \sqrt{\mu _{b_0}}^{-1} &  0 &  \sqrt{\mu _{b_0}} &  0 \\ 0 &  0 &  0 &  0 &  0 &  1 \end{pmatrix}. \end{aligned}$$

The threshold regularity will depend on the component of $\textbf{S}_{b_0}$ transversal to the horizons. We first consider $\textbf{S}_{b_0}$ at the cosmological horizon before considering the event horizon. In the coordinates of (C8), we can calculate that the non-zero Christoffel symbols are

Using the normalized coordinates on the 2-sphere

$$\begin{aligned} ({\underline{\theta }}, {\underline{\varphi }}) {:=} \left( \theta , \frac{\varphi }{\sin \theta }\right) , \end{aligned}$$

the subprincipal operator for the vector-wave operator is given by:

We can similarly use the Christoffel symbols to calculate the subprincipal operator for the linearized Einstein operator, which is exactly the subprincipal operator for the 2-wave operator:

Let us now consider the case where $\mathcal {H}=\overline{\mathcal {H}}^+$. We observe that in order to calculate $\textbf{s}_{\mathbb {L}_{g_{b_0}}}[\overline{\mathcal {H}}^+]$, we only need to consider the components of $\textbf{S}_{b_0}\Big \vert _{\overline{\mathcal {H}}^+}$ in $\partial _r$ since

Then recalling that

$$\begin{aligned} 2\kappa _{b_0, \overline{\mathcal {H}}^+} = - \partial _{r}\mu _{b_0} (r_{b_0, \overline{\mathcal {H}}^+}), \end{aligned}$$

we calculate that at $\overline{\mathcal {H}}^+$, in the splitting of (C9), we can write

$$\begin{aligned} \textbf{S}_{b_0}\Bigg \vert _{\overline{\mathcal {H}}^+} = 2\kappa _{b_0,\overline{\mathcal {H}}^+} \begin{pmatrix} -2& 0& 0& 0& 0& 0\\ 0& 0& 0& 0& 0& 0\\ 0& 0& -1& 0& 0& 0\\ 0& 0& 0& 2& 0& 0\\ 0& 0& 0& 0& 1& 0\\ 0& 0& 0& 0& 0& 0 \end{pmatrix}\partial _r + \widetilde{\textbf{S}}_{b_0, \overline{\mathcal {H}}^+}, \end{aligned}$$

where $\widetilde{\textbf{S}}_{b_0, \overline{\mathcal {H}}^+}$ is a matrix-valued vectorfield tangent to $\overline{\mathcal {H}}^+$. As a result, we have that

$$\begin{aligned} \sup _{\overline{\mathcal {H}}^+}g \left( \textbf{K}_{b_0, \overline{\mathcal {H}}^+}, \Re \left( \overline{\xi }\cdot \textbf{S}_{b_0}\xi \right) \right) \Bigg \vert _{\overline{\mathcal {H}}^+}&= 4\kappa _{b_0,\overline{\mathcal {H}}^+} ,\\ \inf _{\mathcal {H}} g \left( \textbf{K}_{b_0, \overline{\mathcal {H}}^+}, \Re \left( \overline{\xi }\cdot \textbf{S}_{b_0}\xi \right) \right) \Bigg \vert _{\overline{\mathcal {H}}^+}&= -4\kappa _{b_0,\overline{\mathcal {H}}^+}. \end{aligned}$$

Similar calculations at $\mathcal {H}^+$ yield that

$$\begin{aligned} \textbf{S}_{b_0}\Bigg \vert _{\mathcal {H}^+} =-2\kappa _{b_0,\mathcal {H}^+} \begin{pmatrix} -2& 0& 0& 0& 0& 0\\ 0& 0& 0& 0& 0& 0\\ 0& 0& -1& 0& 0& 0\\ 0& 0& 0& 2& 0& 0\\ 0& 0& 0& 0& 1& 0\\ 0& 0& 0& 0& 0& 0 \end{pmatrix}\partial _r + \widetilde{\textbf{S}}_{b_0, \mathcal {H}^+}, \end{aligned}$$

where $\widetilde{\textbf{S}}_{b_0, \mathcal {H}^+}$ is a matrix-valued vectorfield tangent to $\mathcal {H}^+$, and thus

$$\begin{aligned} \sup _{\mathcal {H}^+}g \left( \textbf{K}_{b_0, \mathcal {H}^+}, \Re \left( \overline{\xi }\cdot \textbf{S}_b\xi \right) \right) \Bigg \vert _{\mathcal {H}^+}&= 4\kappa _{b_0,\mathcal {H}^+} ,\\ \inf _{\mathcal {H}^+} g \left( \textbf{K}_{b_0, \mathcal {H}^+}, \Re \left( \overline{\xi }\cdot \textbf{S}_b\xi \right) \right) \Bigg \vert _{\mathcal {H}^+}&= -4\kappa _{b_0,\mathcal {H}^+}. \end{aligned}$$

## Appendix D: Appendix to Section 4

### D.1 Proof of Proposition 4.2

From Corollary 6.8, we already know that $\mathcal {S}(t_*)$ maps $\textbf{H}^{k}(\Sigma )$ to itself. It suffices then to check that:

1. $\mathcal {S}(0) = \operatorname {Id}_{\textbf{H}^{k}(\Sigma )}$: This follows from the definition of $\mathcal {S}(0)$ and the well-posedness theorem for the relevant Cauchy problem.
2. $\mathcal {S}(t_0+t_1) = \mathcal {S}(t_0)\circ \mathcal {S}(t_1)$: This follows from the stationarity of the Kerr-de Sitter black hole spacetime. The relevant Cauchy problem is invariant under time-translations, and thus, solving the Cauchy problem from $[t_1, t_0+t_1]$ is equivalent to solving the problem on $[0, t_0]$. Thus, we can solve the problem from $[0, t_0+t_1]$ by first solving the problem from $[0, t_1]$ and then from $[t_1, t_0+t_1]$.
3. $\mathcal {S}(t_*)$ is continuous in the strong operator topology: Using Corollary 6.8, we see that for each $f\in \textbf{H}^{k}(\Sigma )$, the map $t_*\mapsto \mathcal {S}(t_*)f$ is a $C^0$ curve in $\textbf{H}^{k}(\Sigma )$.

### D.2 Proof of Proposition 4.9

Let

D10 $$\begin{aligned} \textbf{h}(t_*, x) = \sum _{\ell =0}^{n-1} e^{-\mathbbm {i}\sigma t_*}t_*^\ell \textbf{u}_{\ell }(x) \end{aligned}$$

be a $\textbf{H}^{k}$-quasinormal mode solution of $L$. Then define

$$\begin{aligned} F(t_*, \cdot ) {:=} \frac{1}{\mathbbm {i}} \delta _0(t_*)\textbf{h}(0, \cdot ), \end{aligned}$$

so that

$$\begin{aligned} \widehat{F}(\sigma , \cdot ) = \frac{1}{\mathbbm {i}} \textbf{h}(0, \cdot ). \end{aligned}$$

Applying Lemma 4.4, we have that for $\Im \sigma > \textbf{M}$,

$$\begin{aligned} (\mathcal {A}- \sigma )^{-1} \widehat{F}(\sigma ) = \int _{\mathbb {R}^+}e^{\mathbbm {i}\sigma {s_*}}\mathcal {S}({s_*})\textbf{h}(0)\,d{s_*}= \widehat{\textbf{h}}(\sigma ). \end{aligned}$$

Taking the inverse Laplace transform on both sides and applying the Cauchy integral formula over an appropriately deformed contour, we have that

$$\begin{aligned} \textbf{h}(t_*) = {\text {res}}_{\sigma =\sigma _0}(e^{-\mathbbm {i}\sigma t_*}(\mathcal {A}-\sigma )^{-1}\widehat{F}(\sigma )). \end{aligned}$$

Then, Proposition 4.9 follows from the assumption the $\textbf{H}^{k}$-quasinormal spectrum is discrete and thus all poles of $(\mathcal {A}-\sigma )^{-1}$ are of finite order.

### D.3 Proof of Proposition 4.10

Let us first consider the case where $\Xi$ consists of only one $\textbf{H}^{k}$-quasinormal frequency, $\sigma _0$, which is an $\textbf{H}^{k}$-quasinormal mode of order $\ell$. Now for some fixed $F\in H^{-1,\beta }(\mathbb {R}^+; \textbf{H}^{k-1}(\Sigma ))$, we have that $(\mathcal {A}- \sigma )^{-1}\widehat{F}(\sigma )$ is holomorphic near $\sigma _0$ if and only if $\left\langle \widehat{F}(\sigma ), (\mathcal {A}^*-\overline{\sigma })^{-1}G\right\rangle _{L^2(\Sigma )}$ is holomorphic near every $\sigma _0$ for every $G\in \textbf{H}^{1-k}(\Sigma )$. Using the definition of the Laplace transform, this is equivalent to the condition that

$$\begin{aligned} \left\langle F(t_*,x), e^{-\mathbbm {i}\overline{\sigma }t_*}(\mathcal {A}^*-\overline{\sigma })^{-1}G(x)\right\rangle _{L^2(\mathcal {M})} \end{aligned}$$

is holomorphic for every $G\in \textbf{H}^{1-k}(\Sigma )$, near each $\sigma _j\in \Xi$.

Since $(\mathcal {A}^*-\overline{\sigma })^{-1}$ is meromorphic, for a fixed $G\in \textbf{H}^{1-k}(\Sigma )$, we have that for $\sigma$ in a small neighborhood of $\sigma _j$,

$$\begin{aligned} e^{-\mathbbm {i}\overline{\sigma }t_*}(\mathcal {A}^*-\overline{\sigma })^{-1}G = \sum _{j=1}^{\ell }(\overline{\sigma } - \overline{\sigma }_0)^{-j}{G}_{j} + \widetilde{G}(\overline{\sigma }) \end{aligned}$$

with ${G}_{j}\in e^{-\Im \sigma _0t_*}\textbf{H}^{2-k}(\Sigma )$, and $\widetilde{G}$ holomorphic near $\overline{\sigma }_0$ with values in $\textbf{H}^{2-k}(\Sigma )$.

Thus, $\left\langle \widehat{F}(\sigma ), e^{-\mathbbm {i}\overline{\sigma }t_*}(\mathcal {A}^*-\overline{\sigma })G\right\rangle _{L^2(\mathcal {M})}$ is holomorphic at $\sigma _0$ if and only if

D11

for each $1\le j\le \ell$. Observe that (D11) holds for all $j>\ell$ since the poles of $e^{-\mathbbm {i}\overline{\sigma }t_*}(\mathcal {A}^*-\overline{\sigma })^{-1}G$ are all of finite order at most $\ell$. Recalling that *G* was arbitrary, we see that the condition in (D11) is equivalent to the condition that

for all polynomials $p(\sigma )$ in $\sigma$ with coefficients in $\textbf{H}^{1-k}(\Sigma )$. The case where $\Xi$ consists of more than one $\textbf{H}^{k}$-quasinormal frequency then follows directly.

### D.4 Proof of Proposition 4.14

#### Remark 43

Recall that the definition of the space $\textbf{H}^{k}(\Sigma )$, we relied on the construction of a family of vectorfields $\mathcal {K}_j$ which depend on the background metric $g_{b(w)}$. Thus, the family of operators $\mathcal {A}_w - \sigma : D^k(\mathcal {A}_w-\sigma )\rightarrow \textbf{H}^{k}(\Sigma )$ has domain and range varying in *w*. To avoid any difficulties that could rise in the ensuing perturbation theory, we observe that by construction the $\textbf{H}^{k}_b(\Sigma )$ norms for $b\in \mathcal {B}$ are all equivalent norms. Thus, the family $\mathcal {A}_w - \sigma$ is a family with differing domains, but identical range. Likewise, the same is true for the family $\widehat{L}_{b}(\sigma ):D^k(\widehat{L}_b(\sigma ))\rightarrow \underline{H}^{k-1}(\Sigma )$.

The crucial ingredient for the first two items is that the assumptions imply that

D12 $$\begin{aligned} \left\Vert \textbf{u}\right\Vert _{\underline{H}^{k}(\Sigma )} \lesssim \left\Vert (\mathcal {A}_w-\sigma )\textbf{u}\right\Vert _{\underline{H}^{k-1}(\Sigma )} + \left\Vert \textbf{u}\right\Vert _{\underline{H}^{k_0}(\Sigma )} \end{aligned}$$

where $k>k_0$, and $k_0$ is the threshold regularity level defined in (75). This is shown explicitly for the case where the family $L_{b(w)}$ is the family of linearized gauge-fixed Einstein operators $\mathbb {L}_{g_{b(w)}}$ in Sect. 6,^27^.

#### Proof of part 1

To prove the first statement we will show that if $\mathcal {A}_{w_0}-\sigma _0$ is invertible, then there exists a sufficiently small neighborhood $\widetilde{W}\times \widetilde{\Omega }\ni (w_0, \sigma _0)$ such that for all $(w, \sigma )\in \widetilde{W}\times \widetilde{\Omega }$, $\mathcal {A}_{w}-\sigma : D^{k}(\mathcal {A})\rightarrow \textbf{H}^{k}(\Sigma )$ is invertible. An application of the analytic Fredholm theorem as in Theorem 9.7 then shows the meromorphy of $(\mathcal {A}_w-\sigma )^{-1}$ in $\sigma$, and we can conclude using the relation between the Laplace-transformed operator and the infinitesimal generator in Lemma 4.6.

Assume for the sake of contradiction that there exists a sequence of $w_j\rightarrow w_0$, $\sigma _j\rightarrow \sigma$ such that $\mathcal {A}_{w_j}-\sigma _j$ is not invertible for all *j*. Then for each *j*, either the kernel or the cokernel of $\mathcal {A}_{w_j}-\sigma _j$ is nontrivial. Passing to a subsequence, we can assume without loss of generality that $\operatorname {Ker}(\mathcal {A}_{w_j}-\sigma _j)$ is nontrivial for all *j*. Now consider the sequence ${h}_j \in \textbf{H}^{k}(\Sigma )$ such that ${h}_j$ are normalized so that $\left\Vert {h}_j\right\Vert _{\textbf{H}^{k}(\Sigma )}=1$, and moreover,

$$\begin{aligned} (\mathcal {A}_{w_j}-\sigma _j){h}_j = 0 \end{aligned}$$

for all *j*. Using (D12), we see that then for all *j*,

D13 $$\begin{aligned} 1\lesssim \left\Vert {h}_j\right\Vert _{\textbf{H}^{k_0}(\Sigma )}. \end{aligned}$$

Since we constructed ${h}_j$ as a bounded sequence in $\textbf{H}^{k}(\Sigma )$, there exists a subsequence such that ${h}_j\rightharpoonup {h}_0$ weakly for some ${h}_0\in \textbf{H}^{k}(\Sigma )$. Now, using Rellich-Kondrachov, we have in fact that ${h}_j\rightarrow {h}_0$ in $\textbf{H}^{k_0}(\Sigma )$. But then (D13) implies that there exists some non-zero ${h}_0$ such that

$$\begin{aligned} (\mathcal {A}_{w_0}-\sigma _0){h}_0 = 0, \end{aligned}$$

and thus that $\operatorname {Ker}(\mathcal {A}_{w_0}-\sigma _0) \ne \emptyset$, which is a contradiction.

#### Proof of part 2

We now move onto proving the second item. We first show that the map

$$\begin{aligned} I\ni (w,\sigma )\mapsto (\mathcal {A}_w-\sigma )^{-1}\in \mathcal {L}_{weak}(\textbf{H}^{k}(\Sigma ), \textbf{H}^{k}(\Sigma )) \end{aligned}$$

is continuous for all $k>k_0$. To do so, we see from Lemma 4.6 that it is sufficient to show that the map

$$\begin{aligned} I\ni (w,\sigma )\mapsto \widehat{L}_w(\sigma )\in \mathcal {L}_{weak}(\underline{H}^{k-1}(\Sigma ), \underline{H}^{k}(\Sigma )) \end{aligned}$$

is continuous for all $k>k_0$.

Consider a sequence $f_j\in \textbf{H}^{k}(\Sigma )$, such that

$$\begin{aligned} \left\Vert f_j\right\Vert _{\textbf{H}^{k}(\Sigma )} \le 1, \end{aligned}$$

and let $f_j\rightarrow f$ in $\textbf{H}^{k}$, $w_j\rightarrow w_0$, $\sigma _j\rightarrow \sigma _0$. Assume for the sake of contradiction that $\textbf{h}_j$ defined by

$$\begin{aligned} \textbf{h}_j = (\mathcal {A}_{w_j} - \sigma _j)^{-1}f_j \end{aligned}$$

is not a bounded sequence in $\underline{H}^{k}(\Sigma )$. Defining

$$\begin{aligned} \psi _j = \frac{\textbf{h}_j}{\left\Vert \textbf{h}_j\right\Vert _{\underline{H}^{k}(\Sigma )}}, \end{aligned}$$

we see from Eq. (D12), that

$$\begin{aligned} 1\lesssim \left\Vert \textbf{h}_j\right\Vert _{\textbf{H}^{k}(\Sigma )}^{-1} + \left\Vert \psi _j\right\Vert _{\textbf{H}^{k_0}(\Sigma )}. \end{aligned}$$

Using that $\textbf{h}_j$ is not bounded, we find that for *j* sufficiently large,

$$\begin{aligned} 1\lesssim \left\Vert \psi _j\right\Vert _{\textbf{H}^{k-1}(\Sigma )}. \end{aligned}$$

Since by construction, $\psi _j$ is a bounded sequence in $\textbf{H}^{k}(\Sigma )$, there exists some $\psi _0\ne 0\in \textbf{H}^{k}(\Sigma )$ and some subsequence $\psi _{j\ell }$ of $\psi _j$ that converges weakly to $\psi _0$. We then have that

$$\begin{aligned} \frac{f_{j\ell }}{\left\Vert \textbf{h}_{j\ell }\right\Vert _{\textbf{H}^{k}(\Sigma )}} = (\mathcal {A}_{w_{j\ell }}-\sigma _{j\ell })\psi _{j\ell } \rightharpoonup (\mathcal {A}_{w_0}-\sigma _0)\psi _0 \end{aligned}$$

weakly in $\textbf{H}^{k-1}(\Sigma )$. But then since $\textbf{h}_{j\ell }$ is not bounded, we have that $\frac{f_{j\ell }}{\left\Vert \textbf{h}_{j\ell }\right\Vert _{\textbf{H}^{k}(\Sigma )}}$ converges to 0 in $\textbf{H}^{k_0}(\Sigma )$, and thus $(\mathcal {A}_{w_0}-\sigma _0)\psi _0=0$. This is a contradiction, and thus, $\left\{ \textbf{h}_j\right\}$ must be bounded in $\textbf{H}^{k}(\Sigma )$. Thus, any subsequence $\textbf{h}_{j\ell }$ converges weakly to some $\textbf{h}_0\in \textbf{H}^{k}(\Sigma )$. As a result,

$$\begin{aligned} f_{j\ell } =(\mathcal {A}_{w_{j\ell }}-\sigma _{j\ell }) \textbf{h}_{j\ell } \rightharpoonup (\mathcal {A}_{w_0}-\sigma _{0})\textbf{h}_0 \end{aligned}$$

weakly in $\textbf{H}^{k-1}(\Sigma )$ and $(\mathcal {A}_{w_{0}}-\sigma _0)\textbf{h}_0 = f$. Since $\mathcal {A}_{w_0}-\sigma _0$ is invertible, it is in particular injective. Thus every subsequence $\textbf{h}_{j\ell }$ must converge weakly to $\textbf{h}_0\in \textbf{H}^{k}(\Sigma )$, where $\textbf{h}_0$ is independent of the chosen subsequence. As a result, $\textbf{h}_j$ itself converges weakly to $\textbf{h}_0$ in $\textbf{H}^{k}(\Sigma )$.

#### Proof of part 3

For proving the remaining items, it is clear that it suffices to consider the case where $\Omega = \{\sigma \in \Omega : \left\lvert \sigma - \sigma _0\right\rvert <\delta \}$, and the only $\textbf{H}^{k}$-quasinormal frequency of $L_{w_0}(\sigma _0)$ in $\overline{\Omega }$ is exactly $\sigma _0$.

To prove the third item, it is sufficient to consider the poles of $(\mathcal {A}_w-\sigma )^{-1}$, and to prove that the total rank, which can also be expressed as

$$\begin{aligned} d{:=}\sum _{\sigma \in \Lambda _{\operatorname {QNM}}^{k}(L_w)\bigcap \Omega }{\text {rank}}_{\zeta =\sigma }(\mathcal {A}_w-\sigma )^{-1}, \end{aligned}$$

is constant for *w* near $w_0$.

Recall from Theorem 9.7 that $(\mathcal {A}_{w_0}-\sigma ): D^k(\mathcal {A}_{w_0})\rightarrow \textbf{H}^{k}(\Sigma )$ is a zero-index operator. We can thus construct the bases $\{u_j\}_{j=1}^{n}$, $\{f_j\}_{j=1}^{n}$ of the kernel and the cokernel respectively, and define the operator $R: D^k(\mathcal {A}_w)\rightarrow \textbf{H}^{k-1}(\Sigma )$

$$\begin{aligned} Ru{:=}\sum _{j=1}^n\left\langle u, u_j\right\rangle _{L^2(\Sigma )}f_j. \end{aligned}$$

Then, let us denote by $\mathcal {Y}_2 = \operatorname {coKer}(\mathcal {A}_{w_0}-\sigma _0)$, and let $\mathcal {Y}_1$ be defined such that

$$\begin{aligned} \textbf{H}^{k}(\Sigma )= \mathcal {Y}_1\oplus \mathcal {Y}_2. \end{aligned}$$

Then,

$$\begin{aligned} P_w(\sigma ) {:=} \mathcal {A}_w-\sigma + R: D^k(\mathcal {A}_w)\rightarrow \textbf{H}^{k}(\Sigma ) \end{aligned}$$

is invertible for $w=w_0$, $\sigma =\sigma _0$. Moreover, $P_w(\sigma )$ is a zero-order perturbation of $\mathcal {A}_w-\sigma$, and thus equation (D12) continues to hold with $P_w(\sigma )$ in place of $\mathcal {A}_w-\sigma$. As a result, parts 1 and 2 of the current theorem hold for $P_w(\sigma )^{-1}$ in place of $(\mathcal {A}_w-\sigma )^{-1}$ as well. As a result, by considering a sufficiently small parameter space *W*, and a sufficiently small neighborhood $\Omega$ of $\sigma _0$, we can assume that $P_w(\sigma ):D^k(\mathcal {A}_w)\rightarrow \textbf{H}^{k}(\Sigma )$ is invertible on $W\times \Omega$, with a continuous inverse. Define

$$\begin{aligned} Q_w(\sigma ) = 1 - RP_w(\sigma )^{-1}: \textbf{H}^{k}(\Sigma )\rightarrow \textbf{H}^{k}(\Sigma ), \end{aligned}$$

so that

$$\begin{aligned} \mathcal {A}_w-\sigma = Q_w(\sigma )P_w(\sigma ). \end{aligned}$$

Then $\mathcal {A}_w-\sigma$ is invertible if and only if $Q_w(\sigma )$ is invertible. Using the decomposition $\textbf{H}^{k}(\Sigma ) = \mathcal {Y}_1 \oplus \mathcal {Y}_2$, we see that

$$\begin{aligned} Q_w(\sigma ) = \begin{pmatrix} 1 &  0\\ Q_{w,1}(\sigma ) &  Q_{w,2}(\sigma ) \end{pmatrix} \end{aligned}$$

where

$$\begin{aligned} Q_{w,1}(\sigma ) \!=\! -\!RP_w(\sigma )^{-1}\vert _{\mathcal {Y}_1}\!\in \! \mathcal {L}(\mathcal {Y}_1, \mathcal {Y}_2)\quad Q_{w,2}(\sigma ) \!=\! 1 - \!RP_w(\sigma )^{-1}\vert _{\mathcal {Y}_2}\!\in \! \mathcal {L}(\mathcal {Y}_2, \mathcal {Y}_2). \end{aligned}$$

Thus we see that the invertibility of $Q_w(\sigma )$ is equivalent to the invertibility of $Q_{w,2}(\sigma )$, which is a family of linear operators depending continuously on *w* and holomorphically on $\sigma$ acting on a fixed finite-dimensional subspace $\mathcal {Y}_2$.

Recall that by assumption, we have that for any $w\in W$, $\mathcal {A}_w -\sigma$ is an analytic meromorphic family, we have that

$$\begin{aligned} {\text {rank}}_{\zeta = \sigma }\,(\mathcal {A}_w-\zeta )^{-1} = \frac{1}{2\pi \mathbbm {i}} {\text {tr}}\oint _{\partial \Omega } Q_{w,2}(\zeta )^{-1}\partial _\zeta Q_{w,2}(\zeta )\,d\zeta , \end{aligned}$$

which is integer-valued and continuous in *w*.

#### Proof of parts 4 and 5

The fourth and fifth items are proved by similar arguments, so we only provide a detailed proof for the former.

Consider polynomials $p_1(\zeta ), \cdots , p_{d}(\zeta )$ with values in $C^\infty (\Sigma )$ such that

$$\begin{aligned} {h}_j(w_0){:=} \oint _{\partial \Omega }e^{-\mathbbm {i}\zeta t_*}(\mathcal {A}_{w_0}- \sigma _0)^{-1}p_j(\zeta )\,d\zeta \in C^{\infty }(\Sigma ) \end{aligned}$$

span $\Lambda _{\operatorname {QNM}}^{k}(L_{w_0}, \sigma _0)$. Using 3 of the proposition, we see that for sufficiently small $w\in W$, $(\mathcal {A}_w-\zeta )^{-1}$ exists for $\zeta \in \partial \Omega$. Thus, the contour integral

$$\begin{aligned} {h}_j(w){:=} \oint _{\partial \Omega }e^{-\mathbbm {i}\zeta t_*}(\mathcal {A}_{w}-\zeta )^{-1}p_j(\zeta )\,d\zeta \in C^{\infty }(\Sigma ) \end{aligned}$$

is well defined. Using part 2 of the proposition, ${h}_j(w)$ depends continuously on *w* in the topology of $C^{\infty }(\Sigma )$. Thus, $\{{h}_j(w)\}_{j=1}^d$ is a *d*-dimensional set of $C^\infty (\Sigma )$ functions for $w\in W$ sufficiently small. Since $\Lambda _{\operatorname {QNM}}^{k}(L_w, \Omega )$ is also *d*-dimensional and ${h}_j(w)\in \Lambda _{\operatorname {QNM}}^{k}(L_w, \Omega )$, we have that actually

$$\begin{aligned} \Lambda _{\operatorname {QNM}}^{k}(L_w, \Omega ) = {\text {Span}}\,\left\{ {h}_j(w): 1\le j\le d\right\} . \end{aligned}$$

The statement then follows from the map $W\times \mathbb {C}^d\ni (w, (c_1,\cdots , c_d)) \rightarrow \sum c_j{h}_j(w)$.

## Appendix E: Appendix to Section 7

### E.1 Proof of Lemma 7.7

In what ensues, we will use regular coordinates on Schwarzschild-de Sitter, where we set $c_{b_0}=0$. This is equivalent to a coordinate transformation, and because we work with either coordinate independent objects, like principal symbols, or *c*-independent objects, this does not affect our symbolic calculations.

Let $g_{b_0}$ be a Schwarzschild-de Sitter metric. Then in the Boyer-Lindquist $(t, r, \theta , \varphi )$ coordinates,

We now locate the trapped null geodesics in this coordinate system. In anticipation of the more complicated nature of trapping in Kerr-de Sitter, we analyze the trapped set of $g_{b_0}$ by considering the Hamiltonian vectorfield $H_{p_{b_0}}$, where $p_b = G_b$ is the principal symbol of $\Box _{g_{b}}$.

To begin, observe that in the $(t, r, \theta , \varphi ; \sigma , \xi , \eta _{\theta }, \eta _{\varphi })$ coordinates,

E14 $$\begin{aligned} H_{r^2p_{b_0}} = 2\Delta _{b_0}\xi \partial _r - \left( \frac{r^2\partial _r\Delta _{b_0} - 2r\Delta _{b_0}}{\Delta _{b_0}^2}\sigma ^2- \partial _r\Delta _{b_0}\xi ^2\right) \partial _\xi - H_{\left\lvert \eta \right\rvert ^2}. \end{aligned}$$

We then observe that $H_{r^2p_{b_0}}r = 0$ exactly when $\xi =0$. Let us first consider the region where $\Delta _{b_0} \le 0$. Then, we have that if $\Delta _{b_0}\le 0$, and in addition $H_{r^2p_{b_0}}r=0$, then we necessarily have that

$$\begin{aligned} p_{b_0} > 0. \end{aligned}$$

This rules out trapped null-bicharacteristics on the region where $\Delta _{b_0}\le 0$, except exactly those null-bicharacteristics spanning the horizons. Thus, we are left with considering only the interior of the static region, where $\Delta _{b_0}>0$. In this region, if $H_{r^2p_{b_0}}r=0$, we can calculate that

$$\begin{aligned} H^2_{r^2p_{b_0}}r ={}&2\Delta _{b_0}H_{r^2p_{b_0}}\xi + 4\Delta _{b_0}\partial _r\Delta _{b_0}\xi ^2\\ ={}&2\Delta _{b_0}\partial _r\frac{2r^4}{\Delta _{b_0}}\sigma ^2 + 2\Delta _{b_0}\partial _r\Delta _{b_0}\xi ^2. \end{aligned}$$

Then, if $H_{r^2p_{b_0}}r=0$, we can compute that

$$\begin{aligned} H^2_{r^2p_{b_0}}r = \frac{4r^4(r-3M)}{\Delta _{b_0}^2} \end{aligned}$$

so if $H_{r^2p_{b_0}}r = 0$, $\Delta _{b_0}>0$, and $p_{b_0}=0$, then $H_{r^2p_{b_0}}^2r = 0$ only if $r=3M$. Furthermore, using the explicit calculation of $H_{r^2p_{b_0}}^2r$, we see that

$$\begin{aligned} \Delta _{b_0}>0,\, p_{b_0}=0,\, \pm (r-3M)>0,\, H_{p_{b_0}}r =0\implies \pm H_{r^2p_{b_0}}^2r > 0. \end{aligned}$$

### E.2 Proof of Lemma 7.8

The approach will follow the same general outline the approach in Appendix E.1 with Schwarzschild-de Sitter. We again use Eddington-Finkelstein coordinates since all our symbolic calculations are at the principal level, and thus coordinate independent, and using Eddington-Finkelstein significantly simplifies the calculations. We begin with the rescaled principal symbol:

$$\begin{aligned} \rho _b^2p_b = \left( \Delta _b\xi ^2 + \varkappa _b\eta _{\theta }^2\right) + \frac{(1+\lambda _b)^2}{\varkappa _b\sin ^2\theta }\left( a\sin ^2\theta \sigma + \eta _{\varphi }\right) ^2 - \frac{(1+\lambda _b)^2}{\Delta _b}\left( \left( r^2+a^2\right) \sigma + a\eta _{\varphi } \right) ^2, \end{aligned}$$

It is convenient to rewrite this as:

$$\begin{aligned} \rho _b^2p_b = \Delta _b\xi ^2 - \frac{(1+\lambda _b)^2}{\Delta _b}\left( \left( r^2+a^2\right) \sigma + a\eta _{\varphi }\right) ^2 + \rho _b^2 \tilde{p}_b, \end{aligned}$$

where

$$\begin{aligned} \rho _{b}^2\tilde{p}_b = \varkappa _b\eta _{\theta }^2 + \frac{(1+\lambda _b)^2}{\varkappa \sin ^2\theta }(a(\sin ^2\theta )\sigma + \eta _{\varphi })^2. \end{aligned}$$

In this way, we see that

$$\begin{aligned} \Delta _b\xi ^2 - \frac{(1+\lambda _b)^2}{\Delta _b}\left( \left( r^2+a^2\right) \sigma + a\eta _{\varphi }\right) ^2 \end{aligned}$$

has coefficients dependent only on *r*, while the coefficients of $\rho _b^2\tilde{p}_b$ depend only on $\theta$.

An immediate observation is that $(r^2+a^2)\sigma +a\eta _{\varphi }$ cannot vanish along the characteristic set for $\Delta _b>0$, as if it did, then at the same point, we must also necessarily have that $\sigma =\xi =\eta _{\theta }=\eta _{\varphi }=0$, which is not possible.

We now calculate the Hamiltonian vectorfield:

$$\begin{aligned} H_{\rho _b^2p_b} ={}&2(1+\lambda _b)^2\left( \frac{a}{\varkappa _b}(a\sin ^2\theta \sigma +\eta _{\varphi }) - \frac{r^2+a^2}{\Delta _b}\left( (r^2+a^2)\sigma + a\eta _{\varphi } \right) \right) \partial _t\\&- \left( \partial _\theta \varkappa _b\eta _{\theta }^2 + \frac{2(1+\lambda _b)^2\cos \theta }{\varkappa _b\sin \theta } \left( a\sin ^2\theta \sigma +\eta _{\varphi } \right) \right) \partial _{\eta _{\theta }}\\&+ (1+\lambda _b)^2\partial _\theta (\varkappa _b^{-1}\sin ^{-2}\theta )\eta _{\varphi }^2 \partial _{\eta _{\theta }} + 2\Delta _b\xi \partial _r- 2\varkappa _b\eta _{\theta }\partial _\theta \\&-\left( \partial _r\Delta _b\xi ^2 - (1+\lambda _b)^2\partial _r\left( \frac{\left( (r^2+a^2)\sigma + a\eta _{\varphi } \right) ^2}{\Delta _b}\right) ^2\right) \partial _\xi \\&+ 2(1+\lambda _b)^2\left( \frac{1}{\varkappa _b\sin ^2\theta }\left( a\sin ^2\theta \sigma + \eta _{\varphi }\right) -\frac{a}{\Delta _b} \left( (r^2+a^2)\sigma +a\eta _{\varphi } \right) \right) \partial _\varphi . \end{aligned}$$

Equivalently,

$$\begin{aligned} \begin{aligned} H_{{\rho _b^2 p}_b} ={}&-\left( \partial _r\Delta _b\xi ^2 - (1+\lambda _b)^2\partial _r\left( \frac{\left( (r^2+a^2)\sigma + a\eta _{\varphi } \right) ^2}{\Delta _b}\left( (r^2+a^2)\sigma +a\eta _{\varphi } \right) \right) ^2\right) \partial _\xi \\&- \frac{2(1+\lambda _b)^2\left( r^2+a^2 \right) }{\Delta _b}\left( (r^2+a^2)\sigma + a\eta _{\varphi } \right) \partial _t + 2\Delta _b\xi \partial _r+ H_{\rho _b^2\tilde{p}_b}. \end{aligned} \end{aligned}$$

Unlike in the Schwarzschild-de Sitter case, we no longer have integrability of the Hamiltonian flow directly from the conservation of $p$, $\sigma$, and $\eta$. However, we have that

$$\begin{aligned} H_{p_b}p_{b} = 0,\quad H_{p_b}\sigma = 0,\quad H_{p_b}\eta _{\varphi }=0,\quad H_{p_b}\tilde{p}_b =0. \end{aligned}$$

These are now the conserved quantities of motion along the Hamiltonians and show the integrability of the Hamiltonian flow. The fact that $H_{p_b}\tilde{p}_b=0$ comes from the so-called hidden symmetries of the Kerr-de Sitter family, which were first observed in the Kerr family by Carter [6].

We can thus calculate

$$\begin{aligned} H_{\rho _b^2p_b}r = 2\Delta _b\xi \end{aligned}$$

#### Remark 44

We can show that on $\Delta _b< 0$, $H_{\rho _b^2p_b}r$ cannot vanish on the characteristic set. This follows from the observation that if both $\Delta _b< 0$, and $H_{\rho _b^2p_b}r=0$, then,

$$\begin{aligned} \rho _b^2p_b = - \frac{(1+\lambda _b)^2}{\Delta _b}\left( \left( r^2+a^2\right) \sigma + a\eta _{\varphi }\right) ^2 + \rho _b^2 \tilde{p}_b \ge 0, \end{aligned}$$

with equality only if $\sigma =\xi =\eta _{\theta }=\eta _{\varphi }=0$. If instead, $\Delta _b=0$, then the only trapped null-bicharacteristic is

$$\begin{aligned} \left\{ \Delta _b=0, \sigma =\eta _{\theta }= \eta _{\varphi }=0\right\} , \end{aligned}$$

which are exactly the null geodesics spanning the event horizon and the cosmological horizon.

Applying $H_{{\rho _b^2 p}_b}r$ again, we find that

$$\begin{aligned} H^2_{{\rho _b^2 p}_b}r = 2\Delta _bH_{{\rho _b^2 p}_b}\xi + 2 \xi \partial _r\Delta _b H_{{\rho _b^2 p}_b} r. \end{aligned}$$

We can also calculate that

$$\begin{aligned} \begin{aligned} H_{{\rho _b^2 p}_b}\xi = -\partial _r\Delta _b\xi ^2 + (1+\lambda _b)^2\partial _r\left( \frac{\left( (r^2+a^2)\sigma + a\eta _{\varphi } \right) ^2}{\Delta _b}\right) ^2. \end{aligned} \end{aligned}$$

We thus have that

E15 $$\begin{aligned} H^2_{{\rho _b^2 p}_b}r =2\Delta _b(1+\lambda _b)^2\partial _r\left( \Delta _b^{-1}\left( \left( r^2+a^2\right) \sigma +a\eta _{\varphi }\right) ^2 \right) . \end{aligned}$$

In order to locate trapped null bicharacteristics, we will need to analyze the sign of $H_{{\rho _b^2 p}_b}^2r$. To this end, we consider $\mathcal {F}= \Delta _b^{-1}(a\eta _{\varphi }+ (r^2+a^2)\sigma )^2$. We will show that if $\Delta _b>0$, the critical point $\mathring{r}_b$ of $\mathcal {F}$ is unique in $(r_{\mathcal {H}^+}, r_{\overline{\mathcal {H}}^+})$ for fixed $\eta _{\varphi }$; and depends smoothly on $\eta _{\varphi }$. Moreover, $\frac{\partial \mathcal {F}}{\partial r}>0$ for $r>\mathring{r}_b$, and $\frac{\partial \mathcal {F}}{\partial r}<0$ for $r<\mathring{r}_b$. This will show then that the only trapped null bicharacteristics are

$$\begin{aligned} {\Gamma }_b = \left\{ p_b=0, H_{p_b}r = 0, r = \mathring{r}_b\right\} . \end{aligned}$$

The first step will be to calculate

E16 $$\begin{aligned} \partial _r\mathcal {F} = -\left( a\eta _{\varphi }+ \left( r^2+a^2\right) \sigma \right) \Delta _b^{-2}f,\quad f = \left( a\eta _{\varphi }+ \left( r^2+a^2\right) \sigma \right) \partial _r\Delta _b - 4r\Delta _b\sigma . \end{aligned}$$

From this point forward, all calculations will be under the assumption that we are working on the characteristic set on the exterior region without boundary (so in particular, on the region where $p_b=0$ and $\Delta _b>0$) since we are interested in locating trapped null bicharacteristics and their properties, and along null bicharacteristics, $p_b$ is necessarily zero. Recall that we showed earlier that $a\eta _{\varphi }+ (r^2+a^2)\sigma$ is non-vanishing on the characteristic set in the region where $\Delta _b>0$. Consequently, we have that

$$\begin{aligned} \partial _r\mathcal {F} = 0 \quad {\text {if and only if}}\quad f=0, \end{aligned}$$

and

$$\begin{aligned} f=0\implies \partial _r\Delta _b\ne 0. \end{aligned}$$

If now in addition, $\partial _r\mathcal {F}=0$, then

$$\begin{aligned} (r^2+a^2)\sigma -a\eta _{\varphi }= \frac{4r\Delta _b\sigma }{\partial _r\Delta _b}. \end{aligned}$$

Thus we can calculate that

$$\begin{aligned} \partial _{rr}\mathcal {F} = -\left( a\eta _{\varphi }+\left( r^2+a^2\right) \sigma \right) \Delta _b^{-2}\partial _r f = -\frac{4r\sigma }{\Delta _b(\partial _r\Delta _b)^2}\partial _r\Delta _b\partial _rf. \end{aligned}$$

To specify the trapped set, We will show that for $g_b$ a slowly-rotating Kerr-de Sitter metric, if $\Delta _b>0$, and $\partial _r\mathcal {F}= 0$, then $-\sigma \partial _r\Delta _b\partial _rf>0$. To this end, first calculate that

$$\begin{aligned} \partial _rf = \left( a\eta _{\varphi }+ \left( r^2+a^2\right) \sigma \right) \partial _{rr}\Delta _b -4 \Delta _b\sigma - 2r\sigma \partial _r\Delta _b, \end{aligned}$$

where

E17 $$\begin{aligned} \begin{aligned} \partial _r\Delta _b\partial _r f ={}&4r\Delta _b\sigma \partial _{rr}\Delta _b -2\sigma r(\partial _r \Delta _b)^2 - 4\sigma \Delta _b\partial _r\Delta _b\\ ={}&2\sigma \left( -\frac{1}{r}\left( r\partial _r\Delta _b - 4\Delta _b\right) ^2-\frac{2\Delta _b}{r}\left( 6Mr -8a^2\right) \right) . \end{aligned} \end{aligned}$$

Then, observe that $\partial _{rr}\Delta _b<0$ on the entire range where $\Delta _b>0$. As a result from the first line of (E17)

$$\begin{aligned} \partial _r\Delta _b\ge 0 \implies -\sigma \partial _r\Delta _b\partial _rf > 0. \end{aligned}$$

On the other hand, we also see from equation (E17) that

$$\begin{aligned} 6Mr - 8a^2> 0 \implies -\sigma \partial _r\Delta _b\partial _rf > 0. \end{aligned}$$

We can then evaluate that

$$\begin{aligned} \partial _r\Delta _{(M,M)}\left( {\frac{4}{3}M}\right) = \frac{158}{729}M + \frac{328}{729}M(1-9\Lambda M^2)>0. \end{aligned}$$

Thus, we see that for $a<M$, $\partial _r\Delta _{(M, a)}\left( \frac{4}{3}M\right) > 0$. As a result, for $a<M$,

$$\begin{aligned} \partial _r\Delta _{(M, a)} < 0 \implies 6Mr - 8a^2 > 0, \end{aligned}$$

and we have that $-\sigma \partial _r\Delta _b\partial _rf > 0$ for $a<M$. Moreover, if $\partial _r\mathcal {F}=0$, then

$$\begin{aligned} -\sigma \partial _r\Delta _b \partial _rf > 0. \end{aligned}$$

Thus, if $\partial _r\mathcal {F}=0$, then

$$\begin{aligned} \partial _{rr}\mathcal {F} = -\left( a\eta _{\varphi }+\left( r^2+a^2\right) \sigma \right) \Delta _b^{-2}\partial _rf = -\frac{4r}{\Delta _b(\partial _r\Delta _b)^2}\sigma \partial _r\Delta _b\partial _rf>0. \end{aligned}$$

This implies that the critical points of $\mathcal {F}$ are non-degenerate minimum. In particular, as

$$\begin{aligned} \lim _{\Delta _b\rightarrow 0}\mathcal {F} = \infty , \end{aligned}$$

$\mathring{r}_b$, the critical point of $\mathcal {F}$,

1. exists and is unique in $(r_{\mathcal {H}^+}, r_{\overline{\mathcal {H}}^+})$ for fixed $\eta _{\varphi }$;
2. depends smoothly on $\eta _{\varphi }$;
3. lies in an *O*(*a*) neighborhood of $r=3M$;

and $\partial _r\mathcal {F}$ has the same sign as $r-\mathring{r}_b$, implying that

$$\begin{aligned} \Delta _b>0, \quad p_b=0, \quad \pm (r-\mathring{r}_b)>0, \quad H_{p_b} r=0, \implies \pm H^2_{p_b} r > 0. \end{aligned}$$

#### Remark 45

We remark that our above analysis does *not* cover the full sub-extremal range of black hole parameters for Kerr-de Sitter except in the limiting $\Lambda =0$ case of the Kerr family.

This is due to the fact that except in the $\Lambda =0$ case, $a=M$ is not the extremal Kerr-de Sitter black hole, but we do not pursue this line of reasoning further here.

### E.3 Proof of Lemma 7.14

Let $e(x) = \left\{ e_i(x)\right\} _{i=1}^N$ be a local frame for $\mathcal {E}$. We can compute that

$$\begin{aligned}&S_{sub}(P)\left( \sum _{jk}q_{jk}(x,\zeta )u_k(x,\zeta )e_j(x) \right) \\ ={}&\sum _{j\ell }\left( \sum _k \sigma _{sub}(P)_{jk}q_{k\ell } - \mathbbm {i}H_p(q_{j\ell })\right) u_\ell e_j - \mathbbm {i}q_{j\ell }H_p(u_\ell )e_j - \mathbbm {i}q_{j\ell }u_{\ell }e_j H_p,\\&q S_{sub}(P)\left( \sum _{\ell }u_{\ell }(x,\zeta )e_\ell (x) \right) \\ ={}&\sum _{j\ell }\left( \sum _k q_{jk}\sigma _{sub}(P)_{k\ell } \right) u_{\ell }e_j - \mathbbm {i}q_{j\ell }H_p(u_{\ell })e_j -\mathbbm {i}q_{j\ell }u_\ell e_j H_p. \end{aligned}$$

As a result, we have that

$$\begin{aligned}&\left( S_{sub}(P)q - q S_{sub}(P)\right) \sum _{\ell }u_{\ell }(x,\zeta )e_\ell (x)\\ ={}&\sum _{j\ell }\left( \sum _k \left( \sigma _{sub}(P)_{jk}q_{k\ell } - q_{jk}\sigma _{sub}(P)_{k\ell } \right) - \mathbbm {i}H_p(q_{j\ell })\right) u_\ell e_j. \end{aligned}$$

### E.4 Proof of Lemma 7.15

Both sides of (142) are invariantly defined. As a result, it suffices to prove the equality in some local coordinate system. We first consider the left-hand side. Fix an arbitrary point $x_0\in \mathcal {M}$ and introduce normal coordinates $\left\{ y_i\right\} _{i=1}^{N}$ such that the associated Christoffel symbols vanish at $x_0$. Then

E18 $$\begin{aligned} S_{sub}(\bigtriangleup ^{(k)})(x_0, \zeta ) = -\mathbbm {i}H_{\left\lvert \zeta \right\rvert _g^2} = -2\mathbbm {i}g^{jk}\zeta _k\partial _{y^j}. \end{aligned}$$

We now evaluate the right-hand side of (142). For any multi-index *I*, we denote

$$\begin{aligned} dx^I {:=} dx^{i_1}\otimes \cdots \otimes dx^{i_k}. \end{aligned}$$

Observe then that sections of $\pi ^*T_k\mathcal {M}$ are of the form $U_{I}(x, \zeta )dx^I$, and pullbacks of sections (under $\pi$) of $T_k\mathcal {M}$ are of the form $u_I(x)dx^I$. By definition, the pullback connection $\nabla ^{\pi ^*T_k\mathcal {M}}$ on pulled back sections and extended to sections of the pullback bundle using the Leibniz rule is given by

$$\begin{aligned} \nabla ^{\pi ^*T_k\mathcal {M}}_{\partial _{x^j}}(u_i(x)dx^I) = \nabla ^{T_k\mathcal {M}}_{\partial _{x^j}}(u_i(x)dx^I),\qquad \nabla ^{\pi ^*T_k\mathcal {M}}_{\partial _{\zeta ^j}}(u_i(x)dx^I) = 0. \end{aligned}$$

As a result, we have that

$$\begin{aligned} \nabla ^{\pi ^*T_k\mathcal {M}}_{\partial _{x^j}}(u_I(x,\zeta )dx^I)&= \nabla ^{T_k\mathcal {M}}_{\partial _{x^j}}(u_i(\cdot , \zeta )dx^I)(x),\\ \nabla ^{\pi ^*T_k\mathcal {M}}_{\partial _{\zeta _k}}(u_I(x,\zeta )dx^I)&= \partial _{\zeta _k}u_i(x, \zeta )dx^I. \end{aligned}$$

Thus in normal coordinates at $x_0\in \mathcal {M}$, we simply have that

$$\begin{aligned} \nabla ^{\pi ^*T_k\mathcal {M}}_{\partial _{x^j}} = \partial _{x^j},\qquad \nabla ^{\pi ^*T_k\mathcal {M}}_{\partial _{\zeta ^j}} = \partial _{\zeta ^j}. \end{aligned}$$

As a result,

E19 $$\begin{aligned} \nabla ^{\pi ^*T_k\mathcal {M}}_{H_{\left\lvert \zeta \right\rvert _g^2}} = 2g^{jk}\zeta _k\partial _{x^j} \end{aligned}$$

at $x_0$. Then we conclude from the equality between (E18) and (E19).

### E.5 Proof of Lemma 7.16

Fix a point $x\in \mathbb {S}^2$ and consider geodesic normal coordinates $y^1$ and $y^2$ such that the Christoffel symbols vanish at *x*. Denote the dual variables on the fibers of $T^*\mathbb {S}^2$ by $\eta _1$ and $\eta _2$. Then we can calculate that in terms of the geodesic normal coordinates and their dual variables,

To see that $\nabla ^{\pi ^*_{\mathbb {S}^2}T^*\mathbb {S}^2}_{H_{\left\lvert \eta \right\rvert ^2}}$ preserves sections of *E*, observe that

On the other hand, to see that $\nabla ^{\pi ^*_{\mathbb {S}^2}T^*\mathbb {S}^2}_{H_{\left\lvert \eta \right\rvert ^2}}$ also preserves sections of *F*, observe that for sections $\phi$, $\psi$ of $\pi ^*T^*\mathbb {S}^2\rightarrow T^*\mathbb {S}^2$, we have that

Letting $\phi = \eta$, it is clear that if $\psi \cdot \eta = 0$, then $\nabla ^{\pi ^*_{\mathbb {S}^2}T^*\mathbb {S}^2}_{H_{\left\lvert \eta \right\rvert ^2}}\psi \cdot \eta =0$, which proves that $\nabla ^{\pi ^*_{\mathbb {S}^2}T^*\mathbb {S}^2}_{H_{\left\lvert \eta \right\rvert ^2}}$ also preserves the sections of *F*.

### E.6 Proof of Lemma 7.17

We include the following computations for the sake of completeness. The computations are based on the similar proof carried out in Sect. 9 of [31].

Let $(t,r,\omega )$ denote the Boyer-Lindquist coordinates (*t*, *r*) with $\omega$ coordinate on the sphere to be specified. We can then compute that the only non-vanishing Christoffel symbols are:

Recall that Greek letters are used to indicate spacetime indices, lower-case Latin letters are used to indicate spatial indices, and upper-case Latin letters are used to indicate spherical indices. We first calculate the subprincipal operator of the vector wave operator.

Let us denote by $\textbf{S}_{b_0}^{(1)}$ the subprincipal operator associated with the wave operator acting on one forms $\Box ^{(1)}_{g_{b_{0}}}$. Then, by explicit computations in local coordinates, we see that

where  denotes the angular Laplacian acting on one-forms, and  its subprincipal operator. To simplify our calculations at the trapped set, we define:

$$\begin{aligned} e_0 {:=} \sqrt{\mu _{b_0}}^{-1}\partial _t,\quad e^0{:=} \sqrt{\mu _{b_0}}dt,\quad e_1 = \sqrt{\mu _{b_0}}\partial _r,\quad e^1{:=}\sqrt{\mu _{b_0}}^{-1}dr. \end{aligned}$$

Then, let us decompose

E20 $$\begin{aligned}&T^*\mathcal {M} = \left\langle e^0\right\rangle \oplus \left\langle e^1\right\rangle \oplus T^*\mathbb {S}^2,\nonumber \\&S^2T^*\mathcal {M} = \left\langle e^0e^0\right\rangle \oplus \left( \left\langle 2e^0e^1\right\rangle \oplus \left\langle 2e^0\omega \right\rangle \right) \oplus \left( \left\langle e^1e^1\right\rangle \oplus \left\langle 2e^1\omega \right\rangle \ \oplus S^2T^*\mathbb {S}^2 \right) ,\nonumber \\&\qquad \omega \in T^*\mathbb {S}^2. \end{aligned}$$

Now recall that

$$\begin{aligned} {\Gamma }_{b_0} = \left\{ r=3M, \xi =0, \sigma ^2 = \mu _{b_0}r^{-2}\left\lvert \eta \right\rvert ^2\right\} . \end{aligned}$$

We can then write the invariant subprincipal symbol of the vector-valued-wave operator in Schwarzschild-de Sitter using the decomposition in (E20),

$$\begin{aligned} S_{sub}(\Box ^{(1)}_{g_{b_{0}}})\Bigg \vert _{{\Gamma }_{b_0}} ={}&-2\mu _{b_0}^{-1}\sigma D_{t} + \mathbbm {i}r^{-2} \begin{pmatrix} H_{\left\lvert \eta \right\rvert ^2}&  0 &  0 \\ 0 &  H_{\left\lvert \eta \right\rvert ^2} &  0 \\ 0 &  0 &  \nabla ^{\pi ^*_{\mathbb {S}^2}T^*\mathbb {S}^2}_{H_{\left\lvert \eta \right\rvert ^2}} \end{pmatrix}\\&+ \mathbbm {i}\begin{pmatrix} 0 &  -2r^{-1}\sigma &  0\\ -2r^{-1}\sigma & 0&  -2\sqrt{\mu _{b_0}}r^{-3}i_\eta \\ 0&  2r^{-1}\sqrt{\mu _{b_0}}\eta &  0 \end{pmatrix}, \end{aligned}$$

where we use $i_\eta$ to denote the interior product with respect to $\eta$. In a similar vein, we can compute using local coordinates that:

where  denotes the subprincipal symbol of the angular Laplacian acting on two-tensors on the sphere.

Now, using the splitting in (E20) we can write that

$$\begin{aligned} {S_{sub}(\Box ^{(2)}_{g_{b_{0}}})}\Bigg \vert _{{\Gamma }_{b_0}} ={}&\mathbbm {i}r^{-2} \begin{pmatrix} H_{\left\lvert \eta \right\rvert ^2} &  0 &  0 &  0 &  0 &  0 \\ 0 &  H_{\left\lvert \eta \right\rvert ^2} &  0 &  0 &  0 &  0 \\ 0 &  0 &  \nabla ^{\pi ^*_{\mathbb {S}^2}T^*\mathbb {S}^2}_{H_{\left\lvert \eta \right\rvert ^2}} &  0 &  0 &  0 \\ 0 &  0 &  0 &  H_{\left\lvert \eta \right\rvert ^2} &  0 &  0 \\ 0 &  0 &  0 &  0 &  \nabla ^{\pi ^*_{\mathbb {S}^2}T^*\mathbb {S}^2}_{H_{\left\lvert \eta \right\rvert ^2}} &  0 \\ 0 &  0 &  0 &  0 &  0 &  \nabla ^{\pi ^*_{\mathbb {S}^2}T_2\mathbb {S}^2}_{H_{\left\lvert \eta \right\rvert ^2}} \end{pmatrix}\\&-2\mu _{b_0}D_t + \textbf{s}_{b_0, {\Gamma }_{b_0}}, \end{aligned}$$

where

$$\begin{aligned} \textbf{s}_{b_0,{\Gamma }_{b_0}} = \begin{pmatrix} 0 &  -4r^{-1}\sigma &  0 &  0 &  0 &  0\\ -2r^{-1}\sigma &  0 &  -2\sqrt{\mu _{b_0}}r^{-3}i_\eta &  -2r^{-1}\sigma &  0 &  0\\ 0 &  2\sqrt{\mu _{b_0}}r^{-1} \eta &  0 &  0 &  -2r^{-1}\sigma &  0 \\ 0 &  -4 r^{-1}\sigma &  0 &  0 &  -4\sqrt{\mu _{b_0}}r^{-3}i_{\eta }& 0\\ 0 &  0 &  -2r^{-1}\sigma &  2\sqrt{\mu _{b_0}}r^{-1}\eta &  0 &  -2r^{-3}i_{\eta }\\ 0 &  0 &  0 &  0 &  2\sqrt{\mu _{b_0}}r^{-1}\eta &  0 \end{pmatrix}. \end{aligned}$$

For the sake of simplifying calculations, we split

E21 $$\begin{aligned} T^*\mathbb {S}^2 = \left\langle r \left\lvert \eta \right\rvert ^{-1}\eta \right\rangle \oplus \eta ^\perp , \end{aligned}$$

which induces the splitting

$$\begin{aligned} S^2T^*\mathbb {S}^2 = \left\langle (r \left\lvert \eta \right\rvert ^{-1}\eta )^2\right\rangle \oplus 2r \left\lvert \eta \right\rvert ^{-1}\eta \cdot \eta ^\perp \oplus S^2\eta ^\perp . \end{aligned}$$

We thus can further split the splitting (E20) to:

E22 $$\begin{aligned} \begin{aligned} S^2T^*\mathcal {M} ={}&\left\langle e^0e^0\right\rangle \oplus \left\langle 2e^0e^1\right\rangle \oplus (\left\langle 2e^0r\left\lvert \eta \right\rvert ^{-1}\eta \right\rangle \oplus 2e^0\cdot \eta ^\perp )\\&\oplus \left\langle e^1e^1\right\rangle \oplus (\left\langle 2e^1r\left\lvert \eta \right\rvert ^{-1}\eta \right\rangle \oplus 2e^1\cdot \eta ^\perp )\\&\oplus (\left\langle (r\left\lvert \eta \right\rvert ^{-1}\eta )^2\right\rangle \oplus 2r\left\lvert \eta \right\rvert ^{-1}\eta \cdot \eta ^{\perp }\oplus S^2\eta ^\perp ). \end{aligned} \end{aligned}$$

Using that on ${\Gamma }_{b_0}$, $r^2\mu _{b_0}^{-1} \sigma ^2 = \left\lvert \eta \right\rvert ^2$, we can compute that in the decomposition (E21), $\eta :\mathbb {R}\rightarrow T^*\mathbb {S}^2$, $i_\eta : T^*\mathbb {S}^2\rightarrow \mathbb {R}$ are given by:

$$\begin{aligned} \eta = \begin{pmatrix} r^{-1}\left\lvert \eta \right\rvert \\ 0 \end{pmatrix},\quad i_{\eta } = \begin{pmatrix} r\left\lvert \eta \right\rvert&0 \end{pmatrix}. \end{aligned}$$

Thus, $\eta : T^*\mathbb {S}^2\rightarrow S^2 T^*\mathbb {S}^2$, $i_\eta : S^2T^*\mathbb {S}^2\rightarrow T^*\mathbb {S}^2$ are given by

$$\begin{aligned} \eta = \begin{pmatrix} r^{-1}\left\lvert \eta \right\rvert &  0 \\ 0 &  \frac{1}{2}r^{-1}\left\lvert \eta \right\rvert \\ 0 &  0 \end{pmatrix} ,\quad i_\eta = \begin{pmatrix} r\left\lvert \eta \right\rvert &  0 &  0\\ 0 &  r\left\lvert \eta \right\rvert &  0 \end{pmatrix}. \end{aligned}$$

From Lemma 7.16, we have that the only non-diagonal component of $S_{sub}(\Box ^{(2)}_{g_{b_{0}}})$ is precisely $\textbf{s}_{g_{b_0}, {\Gamma }_{b_0}}$. Thus, it suffices to find some $Q$ such that

$$\begin{aligned} Q\textbf{s}_{g_{b_0},{\Gamma }_{b_0}} Q^{-} < \varepsilon _{{\Gamma }}. \end{aligned}$$

To this end, observe that in the splitting of (E22),

$$\begin{aligned} \textbf{s}_{b_0, {\Gamma }_{b_0}} = \mathbbm {i}r^{-2}\sqrt{\mu _{b_0}}\left\lvert \eta \right\rvert \begin{pmatrix} 0& -4& 0& 0& 0& 0& 0& 0& 0& 0\\ -2& 0& -2& 0& -2& 0& 0& 0& 0& 0\\ 0& 2& 0& 0& 0& -2& 0& 0& 0& 0\\ 0& 0& 0& 0& 0& 0& -2& 0& 0& 0\\ 0& -4& 0& 0& 0& -4& 0& 0& 0& 0\\ 0& 0& -2& 0& 2& 0& 0& -2& 0& 0\\ 0& 0& 0& -2& 0& 0& 0& 0& -2& 0\\ 0& 0& 0& 0& 0& 4& 0& 0& 0& 0\\ 0& 0& 0& 0& 0& 0& 2& 0& 0& 0\\ 0& 0& 0& 0& 0& 0& 0& 0& 0& 0 \end{pmatrix}. \end{aligned}$$

The crucial observation is then that $Q_{b_0}\in S^0$, where

E23 $$\begin{aligned} Q_{b_0} {:=} \begin{pmatrix} 0& 0& 0& 0& 0& \frac{96}{\varepsilon _{{\Gamma }_{b_0}}}& 0& 0& 0& 0\\ 0& 0& 0& 0& 0& 0& -\frac{24}{\varepsilon _{{\Gamma }_{b_0}}}& 0& 0& 0\\ -\frac{1}{3}& 0& 0& 0& 0& -\frac{96}{\varepsilon _{{\Gamma }_{b_0}}}& 0& \frac{4}{\varepsilon _{{\Gamma }_{b_0}}^2}& 0& 0\\ 0& 0& \frac{4}{\varepsilon _{{\Gamma }_{b_0}^2}}& 0& 0& 0& 0& 0& 0& 0\\ \frac{1}{3}& 0& 0& 0& 0& 0& 0& \frac{8}{\varepsilon _{{\Gamma }_{b_0}}^2}& 0& 0\\ 0& 0& 0& 0& 0& 0& \frac{24}{\varepsilon _{{\Gamma }_{b_0}}^3}& 0& -\frac{2}{\varepsilon _{{\Gamma }_{b_0}}}& 0\\ 0& 0& 0& -\frac{2}{\varepsilon _{{\Gamma }_{b_0}}}& 0& 0& 0& 0& 0& 0\\ \frac{2}{3}& 0& 0& 0& 0& \frac{96}{\varepsilon _{{\Gamma }_{b_0}}^4}& 0& -\frac{8}{\varepsilon _{{\Gamma }_{b_0}}^2}& 0& 1\\ 0& 0& -\frac{4}{\varepsilon _{{\Gamma }_{b_0}}^2}& 0& 1& 0& 0& 0& 0& 0\\ 0& 1& 0& 0& 0& 0& 0& 0& 0& 0 \end{pmatrix}, \end{aligned}$$

is well defined in a neighborhood of where the splitting in (E22) is well-defined, in particular, in a neighborhood of ${\Gamma }_{b_0}$, and furthermore, $Q$ satisfies the property that

E24 $$\begin{aligned} Q^{-1}\textbf{s}_{b_0}Q\Bigg \vert _{{\Gamma }_{b_0}} = \mathbbm {i}\varepsilon _{{\Gamma }_{b_0}}r^{-2}\sqrt{\mu _{b_0}}\left\lvert \eta \right\rvert \begin{pmatrix} 0& 0& 0& 0& 0& 0& 0& 0& 0& 0\\ 0& 0& 0& 0& 0& 0& 0& 0& 0& 0\\ 0& 0& 0& 1& 0& 0& 0& 0& 0& 0\\ 0& 0& 0& 0& 1& 0& 0& 0& 0& 0\\ 0& 0& 0& 0& 0& 0& 0& 0& 0& 0\\ 0& 0& 0& 0& 0& 0& 1& 0& 0& 0\\ 0& 0& 0& 0& 0& 0& 0& 1& 0& 0\\ 0& 0& 0& 0& 0& 0& 0& 0& 1& 0\\ 0& 0& 0& 0& 0& 0& 0& 0& 0& 1\\ 0& 0& 0& 0& 0& 0& 0& 0& 0& 0 \end{pmatrix}. \end{aligned}$$

Observe that $Q^-$ is a constant coefficient operator on the region where the splitting in (E22) is valid (in particular, in a neighborhood of the trapped set), so clearly commutes with $\Box _{g_{b_{0}}}$.

### E.7 Proof of Proposition 7.11

We remark that while we will prove the proposition for the specific operator $\overline{\mathbb {L}}_{g_b} = \Box _{g_{b}} + \overline{\textbf{S}}_{b} + \overline{\textbf{V}}_b$, the conclusions are true for the more general strongly-hyperbolic operator $L= \Box _{g_{b}} + S + V$.

Integrating by parts, we have that for *h* compactly supported on $\mathcal {D}$ as defined in (176),

E25 $$\begin{aligned} 2\Re \left\langle \overline{\mathbb {L}}_{g_b}{h},\widetilde{X}{h}\right\rangle _{L^2(\mathcal {D})} ={}&\left\langle \Box _{g_{b}}{{h}}, \widetilde{X}{h}\right\rangle _{L^2(\mathcal {D})} + \left\langle \widetilde{X}{h},\Box _{g_{b}}{{h}}\right\rangle _{L^2(\mathcal {D})} + \left\langle \overline{\textbf{S}}_{b}[{h}], \widetilde{X}{h}\right\rangle _{L^2(\mathcal {D})} \nonumber \\&+ \left\langle \widetilde{X}{h}, \overline{\textbf{S}}_{b}[{h}]\right\rangle _{L^2(\mathcal {D})} + 2\Re \left\langle \overline{\textbf{V}}_b{h}, \widetilde{X}{h}\right\rangle _{L^2(\mathcal {D})} \nonumber \\ ={}&\Re \left\langle \left[ \Box _{g_{b}},\widetilde{X}\right] {h}, {h}\right\rangle _{L^2(\mathcal {D})} - \Re \left\langle \overline{\textbf{S}}_{b,a}[\widetilde{X}{h}] + \widetilde{X} \overline{\textbf{S}}_{b,a}[{h}], {h}\right\rangle _{L^2(\mathcal {D})}\nonumber \\&+ \Re \left\langle \left[ \overline{\textbf{S}}_{b,s},\widetilde{X}\right] {h}, {h}\right\rangle _{L^2(\mathcal {D})} + 2\Re \left\langle \widetilde{X}{h}, \overline{\textbf{V}}_b{h}\right\rangle _{L^2(\mathcal {D})}. \end{aligned}$$

E26 $$\begin{aligned} 2\Re \left\langle \overline{\mathbb {L}}_{g_b}{h}, \tilde{q}{h}\right\rangle _{L^2(\mathcal {D})} ={}&\left\langle \left( \Box _{g_{b}}\tilde{q} + \tilde{q}\Box _{g_{b}}\right) {h}, {h}\right\rangle _{L^2(\mathcal {D})} + 2\Re \left\langle \overline{\textbf{S}}_b[{h}], \tilde{q}{h}\right\rangle _{L^2(\mathcal {D})}\nonumber \\&+ 2\Re \left\langle \overline{\textbf{V}}_b{h}, \tilde{q}{h}\right\rangle _{L^2(\mathcal {D})}. \end{aligned}$$

We remark that (E25) follows from the observation that for *h* compactly supported on $\mathcal {D}$,

$$\begin{aligned} 2\Re \left\langle \overline{\textbf{S}}_b[{h}], \widetilde{X}{h}\right\rangle _{L^2(\mathcal {D})} ={}&\left\langle \overline{\textbf{S}}_b[{h}], \widetilde{X}{h}\right\rangle _{L^2(\mathcal {D})} + \left\langle \widetilde{X}{h}, \overline{\textbf{S}}_b[{h}]\right\rangle _{L^2(\mathcal {D})} \\ ={}&-\left\langle \widetilde{X}\overline{\textbf{S}}_b[{h}], {h}\right\rangle _{L^2(\mathcal {D})} + \left\langle \overline{\textbf{S}}_{b,s}\widetilde{X}{h}, [{h}]\right\rangle _{L^2(\mathcal {D})}\\&- \left\langle \overline{\textbf{S}}_{b,a}\widetilde{X}{h}, [{h}]\right\rangle _{L^2(\mathcal {D})}. \end{aligned}$$

As a result,

$$\begin{aligned} \Re \left\langle \overline{\mathbb {L}}_{g_b}{h}, (\widetilde{X}+\tilde{q}){h}\right\rangle _{L^2(\mathcal {D})} = -\Re \mathbb {K}^{\widetilde{X}, \tilde{q}}[{h}], \end{aligned}$$

where

E27 $$\begin{aligned} \mathbb {K}^{\widetilde{X}, \tilde{q}}[{h}] = \mathbb {K}^{\widetilde{X}, \tilde{q}}_{(2)}[{h}] +\mathbb {K}^{\widetilde{X}, \tilde{q}}_{(1)}[{h}] +\mathbb {K}^{\widetilde{X}, \tilde{q}}_{(0)}[{h}], \end{aligned}$$

and

$$\begin{aligned} \begin{aligned} 2\mathbb {K}^{\widetilde{X}, \tilde{q}}_{(2)}[{h}] ={}&\left\langle \left[ \widetilde{X}, \Box _{g_{b}}\right] {h}, {h}\right\rangle _{L^2(\mathcal {D})} + \left\langle \overline{\textbf{S}}_{b,a}[\widetilde{X}{h}] + \widetilde{X} \overline{\textbf{S}}_{b,a}[{h}], {h}\right\rangle _{L^2(\mathcal {D})}\\&- \left\langle \left( \Box _{g_{b}}\tilde{q} + \tilde{q}\Box _{g_{b}}\right) {h}, {h}\right\rangle _{L^2(\mathcal {D})},\\ 2\mathbb {K}^{\widetilde{X}, \tilde{q}}_{(1)}[{h}] ={}&\left\langle \left[ \widetilde{X},\overline{\textbf{S}}_{b,s}\right] {h}, {h}\right\rangle _{L^2(\mathcal {D})} - 2\left\langle \widetilde{X}{h}, \overline{\textbf{V}}_b{h}\right\rangle _{L^2(\mathcal {D})} - 2\left\langle \overline{\textbf{S}}_{b}[{h}], \tilde{q}{h}\right\rangle _{L^2(\mathcal {D})},\\ 2\mathbb {K}^{\widetilde{X}, \tilde{q}}_{(0)}[{h}] ={}&- 2\left\langle \overline{\textbf{V}}_b{h}, \tilde{q}{h}\right\rangle _{L^2(\mathcal {D})}. \end{aligned} \end{aligned}$$

Unfortunately, although the geometry of the exterior domain of outer communication of Kerr-de Sitter allows us to consider only solutions *h* with compact support in *r*, we are rarely interested in *h* with compact support in $t_*$. Thus, we must be careful integrating by parts in $\partial _{t_*}$, since we will necessarily pick up boundary terms. It is because of this integration by parts in $\partial _{t_*}$ that we require the specific form of $\widetilde{X}$, $\tilde{q}$ in (134)^28^.

Integrating by parts in $\partial _{t_*}$ we then repeat the above calculations to see that

E28 $$\begin{aligned} - \Re \left\langle \mathbb {L}_{g_b} {h}, \left( \widetilde{X} +\tilde{q} \right) {h}\right\rangle _{L^2(\mathcal {D})} = \Re \mathbb {K}^{\widetilde{X}, \tilde{q}}[{h}] + \Re \mathbb {J}^{\widetilde{X}, \tilde{q}}(t_*)[{h}]\Bigg \vert _{t_*=0}^{t_*= {T_*}}, \end{aligned}$$

where,

E29 $$\begin{aligned} 2 \mathbb {K}^{\widetilde{X}, \tilde{q}}[{h}] ={}&\left\langle \left[ \widetilde{X},\Box _{g_{b}} \right] {h},{h}\right\rangle _{L^2(\mathcal {D})} +\left\langle \left[ \widetilde{X}, \overline{\textbf{S}}_{b,s} \right] h,h\right\rangle _{L^2(\mathcal {D})}\nonumber \\&+\left\langle \left( \widetilde{X} \overline{\textbf{S}}_{b,a} + \overline{\textbf{S}}_{b,a} \widetilde{X}\right) {h},{h}\right\rangle _{L^2(\mathcal {D})} - \left\langle \left( \tilde{q}\Box _{g_{b}}+ \Box _{g_{b}}\tilde{q}\right) {h}, {h}\right\rangle _{L^2(\mathcal {D})}\nonumber \\&- 2\left\langle \overline{\textbf{S}}_b{h}, \tilde{q} {h}\right\rangle _{L^2(\mathcal {D})} - 2\left\langle \overline{\textbf{V}}_b{h},\left( \widetilde{X}+\tilde{q}\right) {h}\right\rangle _{L^2(\mathcal {D})},\nonumber \\ \mathbb {J}^{\widetilde{X}, \tilde{q}}(t_*)[{h}] ={}&\left\langle n_{\mathring{\Sigma }}{h}, \widetilde{X}{h}\right\rangle _{L^2(\mathring{\Sigma }_{t_*})} + \left\langle \overline{\textbf{S}}{h}, \widetilde{X}_0 g_b(\textbf{T}, n_{\mathring{\Sigma }}){h}\right\rangle _{L^2(\mathring{\Sigma }_{t_*})} \nonumber \\&+ \left\langle g_b(\textbf{T}, n_{\mathring{\Sigma }})\overline{\textbf{S}}_0{h}, \widetilde{X}h\right\rangle _{L^2(\mathring{\Sigma }_{t_*})} + \left\langle n_{\mathring{\Sigma }}{h}, \tilde{q}{h}\right\rangle _{L^2(\mathring{\Sigma }_{t_*})} . \end{aligned}$$

Combining this with the physical divergence theorem in Proposition 2.4 then yields the conclusion.

## Footnotes

1 For black hole solutions, the question is generally stated as whether a *family* of black hole solutions is stable rather than a particular black hole solution. This is in line with the expectation that nontrivial perturbations of a black hole should alter the mass and angular momentum of the black hole.

2 In the notation used in this article, consistent with most of the literature, the lower half-space of the complex plane is the half-space corresponding to exponential decay. However, in some articles in the literature, the half-space corresponding to exponential decay is instead the left half-space.

3 This is typically the primary difficulty in the method of scattering resonances, and has often relied on the use of Melrose’s b-calculus and microlocal analysis.

4 For slowly-rotating Kerr-de Sitter spacetimes, like in slowly-rotating Kerr spacetimes, superradiance is both separated from trapping and effectively handled by redshift. Passing to the more general case, superradiance is also a frequency-dependent obstacle to stability.

5 These geometric difficulties are inherent to the geometry of black hole spacetimes rather than Einstein’s equations, and are present even for simpler problems, such as decay for scalar waves on black hole backgrounds.

6 On Schwarzschild-de Sitter, there is a timelike Killing vectorfield up to the horizons, where it becomes null. See Sect. 2.3 for more details.

7 One should think of $M, \Lambda$ as fixed, and the slowly-rotating condition as a condition on the dimensionless quantity $\frac{a}{M}$.

8 We will use $\nabla ^\alpha \nabla _\alpha$ to denote the tensorial wave operator.

9 For a complex-valued matrix $M_{ab}$, we use the convention that its adjoint is

$$\begin{aligned} M^*_{ab} = \overline{M}_{ba}. \end{aligned}$$

10 In our case, we typically take $g^0$ to be the background metric around which we linearize the equations.

11 Observe that in terms of the Lie derivative, we have that

$$\begin{aligned} \nabla _{g_b}\otimes \omega = \frac{1}{2}\mathcal {L}_{\omega ^\sharp }g_b. \end{aligned}$$

12 The time regularity in the definition of $\lambda$ can be improved beyond $H^{-1}$ to incorporate more negative order Sobolev spaces. But since these do not make an appearance in this paper, we do not optimize this further.

13 *A priori*, only the principal operator of a pseudo-differential operator is well-defined.

14 In particular, a scalar multiple of the identity when interpreted as a $N\times N$ matrix of symbols.

15 We do not restrict the signature of the metric.

16 In the construction, $Q$ will be pseudo-differential in the angular variables, but differential in the radial variable.

17 Typically, one will also have flux terms incurred over the components of the boundary of the domain of integration $\mathcal {D}$ apart from the integrals over $\Sigma _{t_*}$, but here we assume for the sake of simplicity that these flux terms have been proven to have the right signs so as to be excluded from the final inequality.

18 As was the case in the proof of Theorem 8.3, a Morawetz estimate follows directly at this stage from the observation that using Lemma 8.6, there exists a choice of $\check{C}$, such that for $\check{X}$ as defined above

$$\begin{aligned} \left\Vert {h}\right\Vert _{H^1(\mathcal {D})}^2 \lesssim \int _{\mathcal {D}}K^{\check{X}, \check{q}, 0}[{h}] - \Re \left\langle \textbf{S}[{h}], \check{X}{h}\right\rangle _{L^2(\mathcal {D})} + \left\Vert {h}\right\Vert _{L^2(\mathcal {D})}^2, \end{aligned}$$

and an application of the Cauchy-Schwarz theorem to deduce that

$$\begin{aligned} \left. \check{\mathfrak {E}}(t_*)[{h}]\right| _{t_*= 0}^{t_*= {T_*}} +\left\Vert {h}\right\Vert _{H^1(\mathcal {D})}^2 \lesssim \left\Vert \mathbb {L}h\right\Vert _{L^2(\mathcal {D})}^2 + \left\Vert {h}\right\Vert _{L^2(\mathcal {D})}^2. \end{aligned}$$

19 We have included the divergence term in the Lagrange corrector for computational convenience, since it effectively plays a similar role.

20 The restriction to Hermitian $\Psi _{{\operatorname {tan}}}^0(\Sigma )$, $\Psi _{{\operatorname {tan}}}^{-1}(\Sigma )$ is unnecessary, but is sufficient here.

21 Observe that due to the fact that we need to use $\Im \sigma$ as a smallness parameter in controlling $\left\lvert \left\langle q_{b_0}{h}, n_{\mathring{\Sigma }}{h}\right\rangle _{L^2(\mathring{\Sigma })}\right\rvert$, this also limits the size of the spectral gap.

22 We emphasize that here because of the additional smallness factor in *a*, we do not need to rely on the smallness in $\Im \sigma$.

23 In fact, it is sufficient to prove Theorem 8.9 for $\hat{u}(\xi , \eta )$ supported on $C_{ell}\left\lvert \sigma \right\rvert>\left\lvert \xi , \eta \right\rvert > \frac{1}{C_{ell}}\left\lvert \sigma \right\rvert$, but just proving the lower bound is sufficient here.

24 In particular, notice that error terms arising from commuting derivatives with cutoff functions for the boundary terms can be controlled by reducing ${\boldsymbol{\alpha }}$ if necessary, and error terms arising from commuting derivatives with lower-order terms can be controlled by increasing the high-frequency threshold $C_0$ if necessary.

25 We can actually calculate that

$$\begin{aligned} \Box ^{\Upsilon }_{g_{b_0}} = \Box ^{(1)}_{g_{b_{0}}} - \Lambda . \end{aligned}$$

26 This can be clearly seen by using Proposition 4.14, to parametrize the family of spaces $\Lambda _{\operatorname {QNM}}^{k*}(\mathbb {L}_{g_b}, \mathbb {H}^+)$ by means of a continuous map

$$\begin{aligned} \varLambda : B\times \mathbb {R}^D \rightarrow \Lambda _{\operatorname {QNM}}^{k*}(\mathbb {L}_{g_b}, \mathbb {H}^+) \end{aligned}$$

linear in the second argument such that

$$\begin{aligned} \varLambda (b_0, \cdot ) = \lambda _{\mathcal {Z}_{b_0}}\circ \tilde{z}_{b_0}. \end{aligned}$$

With the parametrization $\varLambda$,

$$\begin{aligned} \varLambda (b, \cdot )^{-1}\circ \lambda _{\tilde{\mathcal {Z}}_{b}}\circ \tilde{z}_b^{-1} \end{aligned}$$

can be represented as a complex $4+N_{\Theta }+N_{\Upsilon }$ sized square matrix.
